# Synthesis of new condensed naphthoquinone, pyran and pyrimidine furancarboxylates

**DOI:** 10.3762/bjoc.21.24

**Published:** 2025-02-12

**Authors:** Kirill A Gomonov, Vasilii V Pelipko, Igor A Litvinov, Ilya A Pilipenko, Anna M Stepanova, Nikolai A Lapatin, Ruslan I Baichurin, Sergei V Makarenko

**Affiliations:** 1 Department of organic chemistry, Herzen State Pedagogical University of Russia, nab. r. Moiki 48, 191186 St. Petersburg, Russian Federation; 2 Federal Research Center "Kazan Scientific Center of the Russian Academy of Sciences", Arbuzov Institute of Organic and Physical Chemistry, 8 ul. Akad. Arbuzova, 420088 Kazan, Russian Federation

**Keywords:** CH-acids, 1-halo-1-nitroethenes, heterocyclization, nitroacrylates, X-ray diffraction analysis

## Abstract

New representatives of dioxodihydronaphtho[2,3-*b*]furan-, furo[3,2-*c*][1]benzopyran-, furo[2,3-*d*]pyrano[4,3-*b*]pyran-, furo[2',3':4,5]pyrano[3,2-*c*]chromene-, and furo[2,3-*d*]pyrimidine carboxylates were obtained from the reactions of alkyl 3-bromo-3-nitroacrylates with representatives of carbo- and heterocyclic CH-acids under simple conditions, without the use of organocatalysts. The structures of the synthesized compounds were proven by a set of physicochemical methods, including X-ray diffraction analysis.

## Introduction

The combination of the furan ring with various carbo- and heterocyclic structures leads to the production of new types of heterocyclic compounds with practical useful properties [1–6]. Representatives of the naphthofuran series exhibit anticancer [7] and antiinfectious activity [8]. Anticancer properties have also been found in representatives of the furopyran, furocoumarin [9–10], and furopyrimidine series [11–12].

**Scheme 1:**
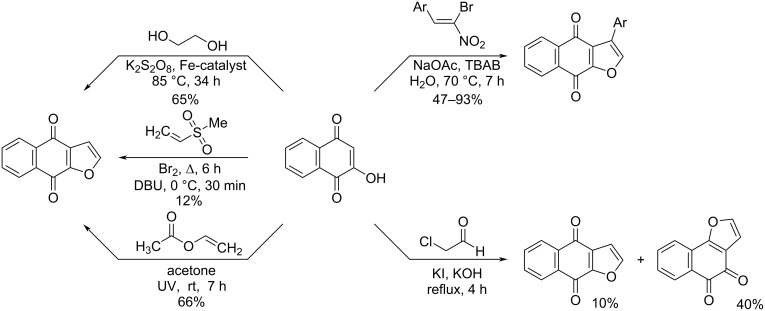
Approaches to the synthesis of naphtho[2,3-*b*]furan-4,9-diones.

It is known that the main approaches to the synthesis of naphtho[2,3-*b*]furan-4,9-diones are reactions of hydroxynaphthalene-1,4-dione with ethane-1,2-diol [13], chloroacetaldehyde [14], ethenyl methyl sulfone [15], or ethenyl acetate [16] (Scheme 1). In turn, naphtho[2,3-*b*]furan-4,9-diones containing an aromatic substituent in the third position can be obtained as a result of the interaction of *gem*-bromonitroalkenes with 2-hydroxynaphthalene-1,4-dione [17–19] (Scheme 1).

**Scheme 2:**
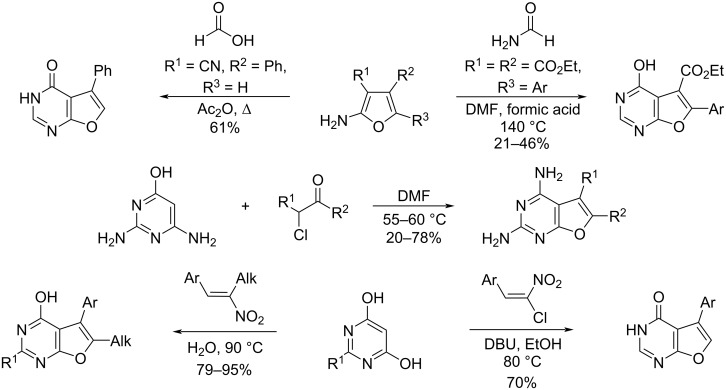
Approaches to the synthesis of furo[2,3-*d*]pyrimidin-4(3*H*)-ones.

Furo[2,3-*d*]pyrimidin-4(3*H*)-ones are obtained on the basis of furoamines [20–21], as well as by the interaction of pyrimidinols with α-halocarbonyl compounds [22], nitroalkenes [23] or *gem*-chloronitroalkenes [21,24–25] (Scheme 2).

**Scheme 3:**
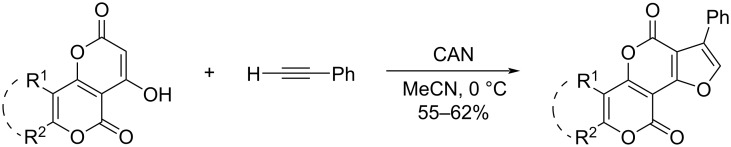
Approaches to the synthesis of furan-containing pyranopyrans and pyranochromenes.

At the same time, the construction of furan-containing pyranopyrans and pyranochromenes in the literature is represented only by the reactions of 4-hydroxy-7-methyl-2*H*,5*H*-pyrano[4,3-*b*]pyran-2,5-dione and 4-hydroxy-2*H*,5*H*-pyrano[3,2-*c*]chromene-2,5-dione with ethynylbenzene [26] (Scheme 3).

The possibility of the formation of substituted furan structures is shown by the example of the reaction of nitroalkenes with aliphatic CH acids [27]. In addition, we have previously demonstrated the effective use of alkyl 3-bromo-3-nitroacrylates in the preparation of condensed furancarboxylates using potassium acetate as a catalyst [28–30].

The present study is aimed at developing methods for the synthesis of a wide range of condensed furancarboxylates based on the interaction of alkyl 3-bromo-3-nitroacrylates [31] with carbo- and heterocyclic CH-acids of the naphthoquinone, pyran, and pyrimidine series.

## Results and Discussion

**Scheme 4:**
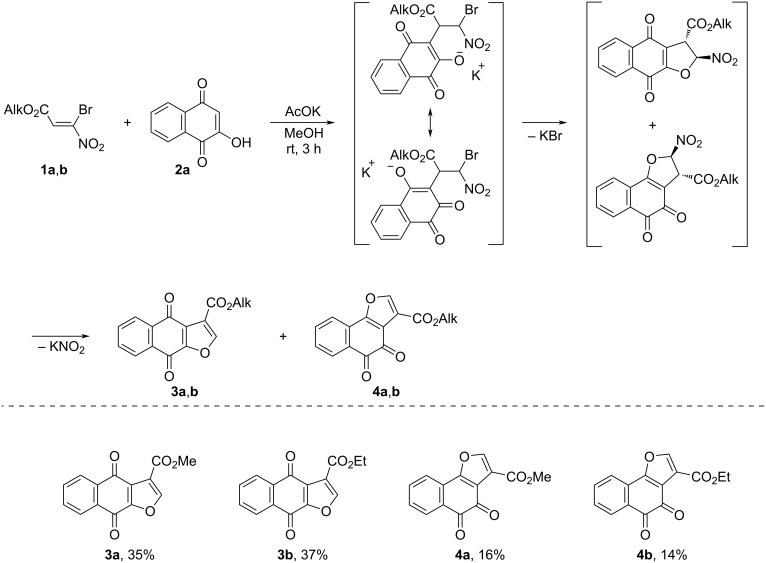
Reaction of alkyl 3-bromo-3-nitroacrylates **1a**,**b** with 2-hydroxynaphthalene-1,4-dione (**2a**).

We have proposed a synthesis of dihydronaphthofuran-3-carboxylates based on the interaction of alkyl 3-bromo-3-nitroacrylates **1a**,**b** with 2-hydroxynaphthalene-1,4-dione (**2a**). The reaction proceeds successfully in a methanol solution in the presence of AcOK for 3 h at room temperature, resulting in the formation of a mixture of alkyl 4,9-dioxo-4,9-dihydronaphtho[2,3-*b*]furan-3-carboxylate **3a**,**b** and alkyl 4,5-dioxo-4,5-dihydronaphtho[1,2-*b*]furan-3-carboxylate **4a**,**b** with a total yield of up to 73% (ratio **3a**/**4a** = 2:1; **3b**/**4b** = 2:1, according to ^1^H NMR spectroscopy) (Scheme 4). Using preparative chromatography, the mixture was separated into individual substances.

**Scheme 5:**
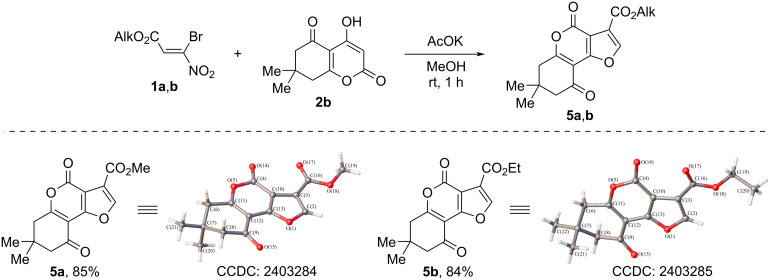
Reaction of alkyl 3-bromo-3-nitroacrylates **1a**,**b** with 4-hydroxy-7,7-dimethyl-7,8-dihydro-2*H*-1-benzopyran-2,5(6*H*)-dione (**2b**).

In turn, alkyl 7,7-dimethyl-4,9-dioxo-6,7,8,9-tetrahydro-4*H*-furo[3,2-*c*]chromene-3-carboxylates **5a**,**b** with a yield of 84–85% were obtained under the same conditions as a result of the interaction of bromonitroacrylates **1a**,**b** with 4-hydroxy-7,7-dimethyl-7,8-dihydro-2*H*-chromene-2,5(6*H*)-dione (**2b**) (ratio acrylate/CH-acid/AcOK = 1:1:1.5, room temperature, 1 h) (Scheme 5).

**Scheme 6:**
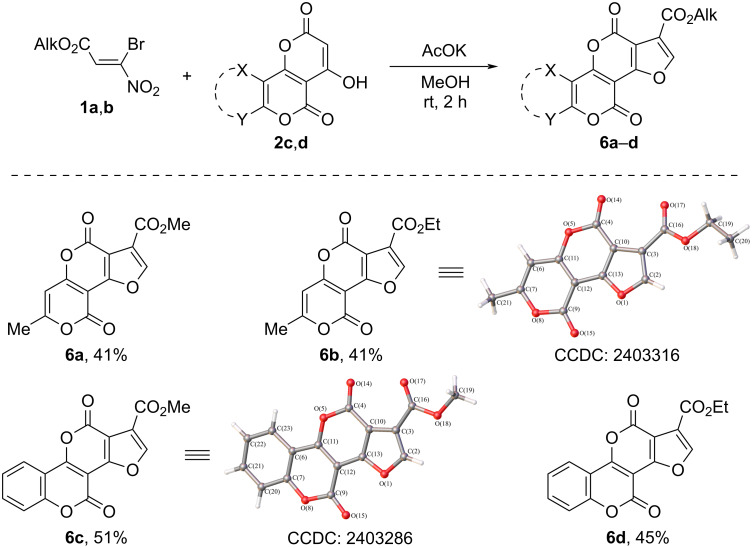
Reaction of alkyl 3-bromo-3-nitroacrylates **1a**,**b** with 4-hydroxy-7-methyl-2*H*,5*H*-pyrano[4,3-*b*]pyran-2,5-dione (**2c**) and 4-hydroxy-2*H*,5*H*-pyrano[3,2-*c*][1]benzopyran-2,5-dione (**2d**).

Polycyclic furan-3-carboxylates – alkyl 7-methyl-4,9-dioxo-4*H*,9*H*-furo[2,3-*d*]pyran[4,3-*b*]pyran-3-carboxylates **6a**,**b** or alkyl 4,11-dioxo-4*H*,11*H*-furo[2',3':4,5]pyran[3,2-*c*]chromene-1-carboxylates **6c**,**d** were obtained by reacting bromonitroacrylates **1a**,**b** with 4-hydroxy-7-methyl-2*H*,5*H*-pyrano[4,3-*b*]pyran-2,5-dione (**2c**) or 4-hydroxy-2*H*,5*H*-pyrano[3,2-*c*][1]benzopyran-2,5-dione (**2d**) in yields up to 51% (Scheme 6).

**Scheme 7:**
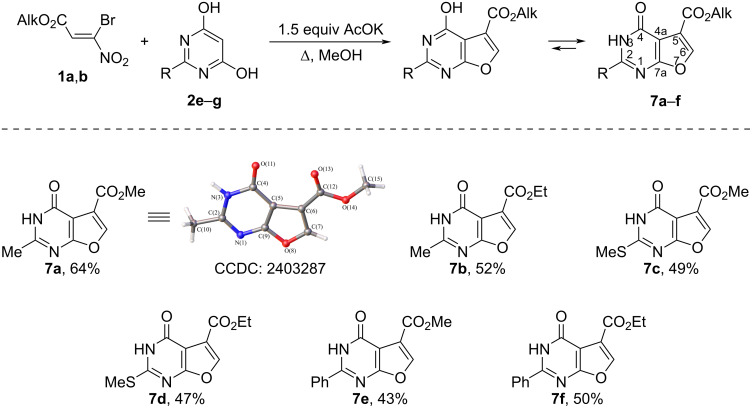
Reaction of alkyl 3-bromo-3-nitroacrylates **1a**,**b** with pyrimidine-4,6-diols **2e–g**.

Furopyrimidines **7a**–**f** were obtained by reacting bromonitroacrylates **1a**,**b** with representatives of substituted pyrimidines **2e**–**g**. The reactions proceed successfully by refluxing in a water–alcohol (in the case of **2e**) or alcohol (in the case of **2f**,**g**) solution for 1–3 h (ratio of acrylate/CH-acid/AcOK = 1:1:1.5) with a yield of 43–64% (Scheme 7).

The structures of the obtained condensed furans **3**–**7** were characterized by a set of physicochemical methods, including X-ray diffraction analysis.

Analysis of the IR spectra of compounds **5a**–**6d** containing an alkoxyfuropyran fragment shows that in the case of methyl esters **5a**, **6a**, and **6c** the absorption band of the alkoxycarbonyl function is shifted to a lower frequency region (1714–1721 cm^−1^), while ethyl esters **5b**, **6b**, and **6d** are characterized by higher frequency values (1725–1731 cm^−1^). This observation may be due to the position of the ester fragment relative to the heterocyclic system. The analysis of the results of X-ray diffraction analysis shows that the torsion angle C(10)–C(3)–C(16)–O(17) of methyl esters **5a** and **6c** is −0.8(2), and 5(2)°, and of ethyl esters **5b** and **6b** is −15.6(2) and −11.5(2)°, respectively, indicating that in the case of methyl esters, the alkoxycarbonyl fragment is less out of plane, causing a greater conjugation with the furan fragment.

The possibility of exhibiting fluorescent properties was investigated for tetracyclic furan-3-carboxylates **6c**,**d**. It was shown that the increased number of conjugated bonds in their molecules leads to a decrease in the energy of the singlet–singlet π→π* transition and the possibility of exhibiting luminescence of the molecules under study. According to the luminescence excitation spectral data, they luminesce in DMSO solution when irradiated with light at wavelengths of 352 and 283 nm.

Compounds **7a**–**f** are capable of existing as lactim–lactam tautomers due to the presence of an amide fragment in their structure (Scheme 7). At the same time, the ^1^H and ^13^C NMR spectra of compounds **7a**–**f** indicate their individuality. The presence of a broadened signal in the ^1^H NMR spectra (DMSO-*d*_6_) in the region of 12.49–12.84 ppm and a C4 signal in the ^13^C NMR spectra (DMSO-*d*_6_) in the region of 158.1–159.0 ppm does not allow us to unambiguously determine the tautomeric form of compounds **7a**–**f** in solution. In turn, in the IR spectra (KBr) of compounds **7a**–**f**, absorption bands of the ester fragment in the region of 1714–1750 cm^−1^ and absorption bands of the carbonyl group of the amide fragment in the region of 1674–1688 cm^−1^ are observed, which suggests the existence of these compounds in the solid phase in the lactam form. The study of compound **7a** by X-ray diffraction analysis convincingly confirms its existence in crystalline form in the lactam form. Thus, we have developed a method for synthesizing a wide range of condensed furancarboxylates. The structures of the compounds obtained were proven by physicochemical methods, as well as by X-ray diffraction analysis. The obtained compounds, due to the combination of different heterocyclic structures in their molecules, may be interesting as potential biologically active substances. In turn, the presence of active electrophilic centers in their structures expands the possibilities of further transformation and obtaining new compounds on their basis.

## Experimental

### General information

IR spectra were recorded on a Shimadzu IRPrestige-21 FT-IR spectrometer for samples in KBr pellets over 400–4000 cm^−1^ range. The electronic absorption spectra were recorded on a Shimadzu UV-2401 PC spectrometer for samples in DMSO solutions in fused quartz cuvettes (optical path length 1.01 mm). Luminescence excitation spectra and luminescence spectra were recorded on a Shimadzu SF-6000 spectrofluorimeter in a 1 cm thick quartz cuvette at room temperature in a DMSO solution (*c* = 10^−5^ mol/L). ^1^Н, ^13^С{^1^H}, ^1^H-^13^C HMQC, and ^1^H-^13^C HMBC NMR spectra were registered on a Jeol ECX–400A instrument at 400 (for ^1^H nuclei) and 100 MHz (for ^13^С nuclei) in CDCl_3_ (δH 7.26, δC 77.16 ppm) and DMSO-*d*_6_ (δH 2.47, δC 40.0 ppm) using residual signals of the nondeuterated solvent as the references. Elemental analysis was performed on a EuroVector EA3000 (dual mode) elemental analyzer. Melting points were determined on a PTP-M melting point apparatus. The reaction completion and purity of the obtained compounds were controlled by TLC on Silufol UV-254 plates with 3:1 hexane/EtOAc mobile phase and visualized under UV light (λ 254 nm).

### X-ray structure determination

X-ray diffraction studies of single crystals of compounds **5a**, **5b**, **6b**, **6c**, and **7a** were carried out on a Bruker D8 QUEST diffractometer. The cell parameters and experimental data were obtained at 100 K (graphite monochromator, λMo Kα = 0.71073 Å, ω and φ scanning in 0.5° steps) at the distributed spectral-analytical center of shared facilities for study of structure, composition and properties of substances and materials of FRC Kazan scientific center of Russian academy of sciences. Crystals of compounds suitable for X-ray structural analysis were obtained by crystallization from MeOH (**5a**), EtOH (**5b**), or DMFA (**6b**, **6c**, and **7a**). Single crystals of a suitable size were glued to the top of a glass fiber in a random orientation. The preliminary unit cell parameters were determined using three runs at different ω angle positions with 12 frames per run (φ-scan technique). Data collection and indexing, determination and refinement of unit cell parameters were carried out using the APEX2 software package [32]. Absorption correction was carried out according to the SADABS program [33]. The structures were solved by the direct method according to the SHELXT-2014/5 program [34] and refined by the full-matrix least-square on *F*^2^ according to the SHELXL-2018/3 program [35]. All calculations were performed using the WingX-2020.1 software package [36]. Structure **6c** was refined using OLEX2-1.5 software package [37]. Non-hydrogen atoms were refined in anisotropic approximation. The hydrogen atoms were placed in calculated positions and refined according to the rider model. The figures were performed in the Mercury 2020.3 program [38], the analysis of intermolecular contacts was performed according to the PLATON program [39].

The crystal of **5b** (*Z*’ = 3), contains 3 independent molecules, the crystals of **6c** and **7a** (*Z*’ = 2) contain 2 independent molecules, with all independent molecules having similar conformation and equivalent geometric parameters within the experimental error limits.

The crystallographic data of the structure are deposited in the Cambridge Crystal Structure Data Bank (CCDC **5a**: 2403284; CCDC **5b**: 2403285; CCDC **6b**: 2403316; CCDC **6c**: 2403286; CCDC **7a**: 2403287). Statistics on the collection of X-ray diffraction data and refinement of the structure are shown in Table S1 in Supporting Information File 1.

## Supporting Information

File 1 General synthetic procedures, characterization data and copies of IR spectra, ^1^H, ^13^C spectra of all synthesized compounds, as well as ^1^H-^13^C HMQC, ^1^H-^13^C HMBC spectra and the crystallographic data for compounds **5a**, **5b**, **6b**, **6c**, and **7a**.

## Data Availability

All data that supports the findings of this study is available in the published article and/or the supporting information of this article.
