# Supplementary material for: Synthesis of new condensed naphthoquinone, pyran and pyrimidine furancarboxylates
Source: Beilstein J Org Chem. 2025 Feb 12;21:340–7. doi: 10.3762/bjoc.21.24 (PMC11833173; doi:10.3762/bjoc.21.24)
Supplement: File 1 — General synthetic procedures, characterization data and copies of IR spectra, 1H, 13C spectra of all synthesized compounds, as well as 1H-13C HMQC, 1H-13C HMBC spectra and the crystallographic data for compounds 5a, 5b, 6b, 6c, and 7a. [file Beilstein_J_Org_Chem-21-340-s001.pdf]

BEILSTEIN JOURNAL OF ORGANIC CHEMISTRY

Supporting Information

for

Synthesis of new condensed naphthoquinone, pyran and pyrimidine furancarboxylates

Kirill A. Gomonov, Vasilii V. Pelipko, Igor A. Litvinov, Ilya A. Pilipenko, Anna M. Stepanova, Nikolai A. Lapatin, Ruslan I. Baichurin and Sergei V. Makarenko

Beilstein J. Org. Chem. **2025,** *21,* 340–347. doi:10.3762/bjoc.21.24

General synthetic procedures, characterization data and copies of IR spectra, ^{1}H, ^{13}C spectra of all synthesized compounds, as well as ^{1}H-^{13}C HMQC, ^{1}H-^{13}C HMBC spectra and the crystallographic data for compounds 5a, 5b, 6b, 6c, and 7a

License and Terms: This is a supporting information file under the terms of the Creative Commons Attribution License (https://creativecommons.org/licenses/by/4.0). Please note that the reuse, redistribution and reproduction in particular requires that the author(s) and source are credited and that individual graphics may be subject to special legal provisions.
The license is subject to the *Beilstein Journal of Organic Chemistry* terms and conditions: (https://www.beilstein-journals.org/bjoc/terms)

Table of contents

Procedure for the synthesis of CH-acids **2a**–**g** S6
Procedure for the synthesis of condensed furancarboxylates **3**–**7** S6
Spectra of the starting CH-acids
1. IR spectrum of 4-hydroxy-7-methyl-2*H*,5*H*-pyrano[4,3-*b*]pyran-2,5-dione (**2c**) in KBr S12
2. IR spectrum of 4-hydroxy-2*H*,5*H*-pyrano[3,2-*c*][1]benzopyran-2,5-dione (**2d**) in KBr S12
3. ^{1}H NMR spectrum of 2-hydroxynaphthalene-1,4-dione (**2a**) in DMSO-d_6 .... S13
4. ^{13}C{^{1}H} NMR spectrum of 2-hydroxynaphthalene-1,4-dione (**2a**) in DMSO-d_6 .... S13
5. ^{1}H NMR spectrum of 4-hydroxy-7-methyl-2*H*,5*H*-pyrano[4,3-*b*]pyran-2,5-dione (**2c**) in DMSO-d_6 .... S14
6. ^{13}C{^{1}H} NMR spectrum of 4-hydroxy-7-methyl-2*H*,5*H*-pyrano[4,3-*b*]pyran-2,5-dione (**2c**) in DMSO-d_6 .... S14
7. ^{1}H-^{13}C HMQC spectrum of 4-hydroxy-7-methyl-2*H*,5*H*-pyrano[4,3-*b*]pyran-2,5-dione (**2c**) in DMSO-d_6 .... S15
8. ^{1}H-^{13}C HMBC spectrum of 4-hydroxy-7-methyl-2*H*,5*H*-pyrano[4,3-*b*]pyran-2,5-dione (**2c**) in DMSO-d_6 .... S15
9. ^{1}H NMR spectrum of 4-hydroxy-2*H*,5*H*-pyrano[3,2-*c*][1]benzopyran-2,5-dione (**2d**) in DMSO-d_6 S16
10. ^{13}C{^{1}H} NMR spectrum of 4-hydroxy-2*H*,5*H*-pyrano[3,2-*c*][1]benzopyran-2,5-dione (**2d**) in DMSO-d_6 .... S16
11. ^{1}H-^{13}C HMQC spectrum of 4-hydroxy-2*H*,5*H*-pyrano[3,2-*c*][1]benzopyran-2,5-dione (**2d**) in DMSO-d_6 .... S17
12. ^{1}H-^{13}C HMBC spectrum of 4-hydroxy-2*H*,5*H*-pyrano[3,2-*c*][1]benzopyran-2,5-dione (**2d**) in DMSO-d_6 .... S17
13. ^{1}H NMR spectrum of 2-methylpyrimidine-4,6-diol (**2e**) in DMSO-d_6 .... S18
14. ^{13}C{^{1}H} NMR spectrum of 2-methylpyrimidine-4,6-diol (**2e**) in DMSO-d_6 .... S18
15. ^{1}H NMR spectrum of 2-(methylsulfanyl)pyrimidine-4,6-diol (**2f**) in DMSO-d_6 .... S19
16. ^{13}C{^{1}H} NMR spectrum of 2-(methylsulfanyl)pyrimidine-4,6-diol (**2f**) in DMSO-d_6 .... S19
17. ^{1}H NMR spectrum of 2-phenylpyrimidine-4,6-diol (**2g**) in DMSO-d_6 .... S20
18. ^{13}C{^{1}H} NMR spectrum of 2-phenylpyrimidine-4,6-diol (**2g**) in DMSO-d_6 .... S20
19. ^{1}H-^{13}C HMQC spectrum of 2-phenylpyrimidine-4,6-diol (**2g**) in DMSO-d_6 .... S21
20. ^{1}H-^{13}C HMBC spectrum of 2-phenylpyrimidine-4,6-diol (**2g**) in DMSO-d_6 .... S21
Spectra of condensed furancarboxylates
21. IR spectrum of methyl 4,9-dioxo-4,9-dihydronaphtho[2,3-*b*]furan-3-carboxylate (**3a**) in KBr S22
22. IR spectrum of methyl 4,5-dioxo-4,5-dihydronaphtho[1,2-*b*]furan-3-carboxylate (**4a**) in KBr S22
23. IR spectrum of ethyl 4,9-dioxo-4,9-dihydronaphtho[2,3-*b*]furan-3-carboxylate (**3b**) in KBr S23
24. IR spectrum of ethyl 4,5-dioxo-4,5-dihydronaphtho[1,2-*b*]furan-3-carboxylate (**4b**) in KBr S23
25. IR spectrum of methyl 7,7-dimethyl-4,9-dioxo-6,7,8,9-tetrahydro-4*H*-furo[3,2-*c*][1]benzopyran-3-carboxylate (**5a**) in KBr S24
26. IR spectrum of ethyl 7,7-dimethyl-4,9-dioxo-6,7,8,9-tetrahydro-4*H*-furo[3,2-*c*][1]benzopyran-3-carboxylate (**5b**) in KBr S24
27. IR spectrum of methyl 7-methyl-4,9-dioxo-4*H*,9*H*-furo[2,3-*d*]pyrano[4,3-*b*]pyran-3-carboxylate (**6a**) in KBr S25
28. IR spectrum of ethyl 7-methyl-4,9-dioxo-4*H*,9*H*-furo[2,3-*d*]pyrano[4,3-*b*]pyran-3-carboxylate (**6b**) in KBr S25
29. IR spectrum of methyl 4,11-dioxo-4*H*,11*H*-furo[2',3':4,5]pyrano[3,2-*c*]chromene-1-carboxylate (**6c**) in KBr S26
30. IR spectrum of ethyl 4,11-dioxo-4*H*,11*H*-furo[2',3':4,5]pyrano[3,2-*c*]chromene-1-carboxylate (**6d**) in KBr S26
31. IR spectrum of methyl 2-methyl-4-oxo-3,4-dihydrofuro[2,3-d]pyrimidine-5-carboxylate (**7a**) in

KBr S27
32. IR spectrum of ethyl 2-methyl-4-oxo-3,4-dihydrofuro[2,3-d]pyrimidine-5-carboxylate (**7b**) in KBr .. S27
33. IR spectrum of methyl 2-(methylsulfanyl)-4-oxo-3,4-dihydrofuro[2,3-d]pyrimidine-5-carboxylate (**7c**) in KBr S28
34. IR spectrum of ethyl 2-(methylsulfanyl)-4-oxo-3,4-dihydrofuro[2,3-d]pyrimidine-5-carboxylate (**7d**) in KBr S28
35. IR spectrum of methyl 4-oxo-2-phenyl-3,4-dihydrofuro[2,3-d]pyrimidine-5-carboxylate (**7e**) in KBr S29
36. IR spectrum of ethyl 4-oxo-2-phenyl-3,4-dihydrofuro[2,3-d]pyrimidine-5-carboxylate (**7f**) in KBr .. S29
37. ^{1}H NMR spectrum of the mixture methyl 4,9-dioxo-4,9-dihydronaphtho[2,3-*b*]furan-3-carboxylate (**3a**) and methyl 4,5-dioxo-4,5-dihydronaphtho[1,2-*b*]furan-3-carboxylate (**4a**) in $CDCl_3$ S30
38. ^{13}C{^{1}H} NMR spectrum of ^{1}H NMR spectrum of the mixture methyl 4,9-dioxo-4,9-dihydronaphtho[2,3-*b*]furan-3-carboxylate (**3a**) and methyl 4,5-dioxo-4,5-dihydronaphtho[1,2-*b*]furan-3-carboxylate (**4a**) in $CDCl_3$ S30
39. ^{1}H NMR spectrum of methyl 4,9-dioxo-4,9-dihydronaphtho[2,3-*b*]furan-3-carboxylate (**3a**) in $CDCl_3$ S31
40. ^{13}C{^{1}H} NMR spectrum of methyl 4,9-dioxo-4,9-dihydronaphtho[2,3-*b*]furan-3-carboxylate (**3a**) in $CDCl_3$ S31
41. ^{1}H-^{13}C HMQC spectrum of methyl 4,9-dioxo-4,9-dihydronaphtho[2,3-*b*]furan-3-carboxylate (**3a**) in $CDCl_3$ S32
42. ^{1}H-^{13}C HMBC spectrum of methyl 4,9-dioxo-4,9-dihydronaphtho[2,3-*b*]furan-3-carboxylate (**3a**) in $CDCl_3$ S32
43. ^{1}H NMR spectrum of methyl 4,5-dioxo-4,5-dihydronaphtho[1,2-*b*]furan-3-carboxylate (**4a**) in $CDCl_3$ S33
44. ^{13}C{^{1}H} NMR spectrum of methyl 4,5-dioxo-4,5-dihydronaphtho[1,2-*b*]furan-3-carboxylate (**4a**) in $CDCl_3$ S33
45. ^{1}H-^{13}C HMQC spectrum of methyl 4,5-dioxo-4,5-dihydronaphtho[1,2-*b*]furan-3-carboxylate (**4a**) in $CDCl_3$ S34
46. ^{1}H-^{13}C HMBC spectrum of methyl 4,5-dioxo-4,5-dihydronaphtho[1,2-*b*]furan-3-carboxylate (**4a**) in $CDCl_3$ S34
47. ^{1}H NMR spectrum of the mixture ethyl 4,9-dioxo-4,9-dihydronaphtho[2,3-*b*]furan-3-carboxylate **3b** and ethyl 4,5-dioxo-4,5-dihydronaphtho[1,2-*b*]furan-3-carboxylate (**4b**) in $CDCl_3$ S35
48. ^{13}C{^{1}H} NMR spectrum of the mixture ethyl 4,9-dioxo-4,9-dihydronaphtho[2,3-*b*]furan-3-carboxylate **3b** and ethyl 4,5-dioxo-4,5-dihydronaphtho[1,2-*b*]furan-3-carboxylate (**4b**) in $CDCl_3$ S35
49. ^{1}H NMR spectrum of ethyl 4,9-dioxo-4,9-dihydronaphtho[2,3-*b*]furan-3-carboxylate (**3b**) in $CDCl_3$ S36
50. ^{13}C{^{1}H} NMR spectrum of ethyl 4,9-dioxo-4,9-dihydronaphtho[2,3-*b*]furan-3-carboxylate (**3b**) in $CDCl_3$ S36
51. ^{1}H-^{13}C HMQC spectrum of ethyl 4,9-dioxo-4,9-dihydronaphtho[2,3-*b*]furan-3-carboxylate (**3b**) in $CDCl_3$ S37
52. ^{1}H-^{13}C HMBC spectrum of ethyl 4,9-dioxo-4,9-dihydronaphtho[2,3-*b*]furan-3-carboxylate (**3b**) in $CDCl_3$ S37
53. ^{1}H NMR spectrum of ethyl 4,5-dioxo-4,5-dihydronaphtho[1,2-*b*]furan-3-carboxylate (**4b**) in $CDCl_3$ S38
54. ^{13}C{^{1}H} NMR spectrum of ethyl 4,5-dioxo-4,5-dihydronaphtho[1,2-*b*]furan-3-carboxylate (**4b**) in $CDCl_3$ S38
55. ^{1}H-^{13}C HMQC spectrum of ethyl 4,5-dioxo-4,5-dihydronaphtho[1,2-*b*]furan-3-carboxylate (**4b**) in $CDCl_3$ S39

56. ^{1}H-^{13}C HMBC spectrum of ethyl 4,5-dioxo-4,5-dihydronaphtho[1,2-*b*]furan-3-carboxylate (**4b**) in $CDCl_3$ S39

57. ^{1}H NMR spectrum of methyl 7,7-dimethyl-4,9-dioxo-6,7,8,9-tetrahydro-4*H*-furo[3,2-*c*][1]benzopyran-3-carboxylate (**5a**) in $CDCl_3$ S40

58. ^{13}C{^{1}H} NMR spectrum of methyl 7,7-dimethyl-4,9-dioxo-6,7,8,9-tetrahydro-4*H*-furo[3,2-*c*][1]benzopyran-3-carboxylate (**5a**) in $CDCl_3$ S40

59. ^{1}H-^{13}C HMQC spectrum of methyl 7,7-dimethyl-4,9-dioxo-6,7,8,9-tetrahydro-4*H*-furo[3,2-*c*][1]benzopyran-3-carboxylate (**5a**) in $CDCl_3$ S41

60. ^{1}H-^{13}C HMBC spectrum of methyl 7,7-dimethyl-4,9-dioxo-6,7,8,9-tetrahydro-4*H*-furo[3,2-*c*][1]benzopyran-3-carboxylate (**5a**) in $CDCl_3$ S41

61. ^{1}H NMR spectrum of ethyl 7,7-dimethyl-4,9-dioxo-6,7,8,9-tetrahydro-4*H*-furo[3,2-*c*][1]benzopyran-3-carboxylate (**5b**) in $CDCl_3$ S42

62. ^{13}C{^{1}H} NMR spectrum of ethyl 7,7-dimethyl-4,9-dioxo-6,7,8,9-tetrahydro-4*H*-furo[3,2-*c*][1]benzopyran-3-carboxylate (**5b**) in $CDCl_3$ S42

63. ^{1}H-^{13}C HMQC spectrum of ethyl 7,7-dimethyl-4,9-dioxo-6,7,8,9-tetrahydro-4*H*-furo[3,2-*c*][1]benzopyran-3-carboxylate (**5b**) in $CDCl_3$ S43

64. ^{1}H-^{13}C HMBC spectrum of ethyl 7,7-dimethyl-4,9-dioxo-6,7,8,9-tetrahydro-4*H*-furo[3,2-*c*][1]benzopyran-3-carboxylate (**5b**) in $CDCl_3$ S43

65. ^{1}H NMR spectrum of methyl 7-methyl-4,9-dioxo-4*H*,9*H*-furo[2,3-*d*]pyrano[4,3-*b*]pyran-3-carboxylate (**6a**) in DMSO-d_6 .... S44

66. ^{13}C{^{1}H} NMR spectrum of methyl 7-methyl-4,9-dioxo-4*H*,9*H*-furo[2,3-*d*]pyrano[4,3-*b*]pyran-3-carboxylate (**6a**) in DMSO-d_6 .... S44

67. ^{1}H-^{13}C HMQC spectrum of methyl 7-methyl-4,9-dioxo-4*H*,9*H*-furo[2,3-*d*]pyrano[4,3-*b*]pyran-3-carboxylate (**6a**) in DMSO-d_6 .... S45

68. ^{1}H-^{13}C HMBC spectrum of methyl 7-methyl-4,9-dioxo-4*H*,9*H*-furo[2,3-*d*]pyrano[4,3-*b*]pyran-3-carboxylate (**6a**) in DMSO-d_6 .... S45

69. ^{1}H NMR spectrum of ethyl 7-methyl-4,9-dioxo-4*H*,9*H*-furo[2,3-*d*]pyrano[4,3-*b*]pyran-3-carboxylate (**6b**) in DMSO-d_6 .... S46

70. ^{13}C{^{1}H} NMR spectrum of ethyl 7-methyl-4,9-dioxo-4*H*,9*H*-furo[2,3-*d*]pyrano[4,3-*b*]pyran-3-carboxylate (**6b**) in DMSO-d_6 .... S46

71. ^{1}H-^{13}C HMQC spectrum of ethyl 7-methyl-4,9-dioxo-4*H*,9*H*-furo[2,3-*d*]pyrano[4,3-*b*]pyran-3-carboxylate (**6b**) in DMSO-d_6 .... S47

72. ^{1}H-^{13}C HMBC spectrum of ethyl 7-methyl-4,9-dioxo-4*H*,9*H*-furo[2,3-*d*]pyrano[4,3-*b*]pyran-3-carboxylate (**6b**) in DMSO-d_6 .... S47

73. ^{1}H NMR spectrum of methyl 4,11-dioxo-4*H*,11*H*-furo[2',3':4,5]pyrano[3,2-*c*]chromene-1-carboxylate (**6c**) in DMSO-d_6 .... S48

74. ^{13}C{^{1}H} NMR spectrum of methyl 4,11-dioxo-4*H*,11*H*-furo[2',3':4,5]pyrano[3,2-*c*]chromene-1-carboxylate (**6c**) in DMSO-d_6 .... S48

75. ^{1}H-^{13}C HMQC spectrum of methyl 4,11-dioxo-4*H*,11*H*-furo[2',3':4,5]pyrano[3,2-*c*]chromene-1-carboxylate (**6c**) in DMSO-d_6 .... S49

76. ^{1}H-^{13}C HMBC spectrum of methyl 4,11-dioxo-4*H*,11*H*-furo[2',3':4,5]pyrano[3,2-*c*]chromene-1-carboxylate (**6c**) in DMSO-d_6 .... S49

77. ^{1}H NMR spectrum of ethyl 4,11-dioxo-4*H*,11*H*-furo[2',3':4,5]pyrano[3,2-*c*]chromene-1-carboxylate (**6d**) in DMSO-d_6 .... S50

78. ^{13}C{^{1}H} NMR spectrum of ethyl 4,11-dioxo-4*H*,11*H*-furo[2',3':4,5]pyrano[3,2-*c*]chromene-1-carboxylate (**6d**) in DMSO-d_6 .... S50

79. ^{1}H-^{13}C HMQC spectrum of ethyl 4,11-dioxo-4*H*,11*H*-furo[2',3':4,5]pyrano[3,2-*c*]chromene-1-carboxylate (**6d**) in DMSO-d_6 .... S51

80. ^{1}H-^{13}C HMBC spectrum of ethyl 4,11-dioxo-4*H*,11*H*-furo[2',3':4,5]pyrano[3,2-*c*]chromene-1-carboxylate (**6d**) in DMSO-d_6 .... S51

81. ^{1}H NMR spectrum of methyl 2-methyl-4-oxo-3,4-dihydrofuro[2,3-*d*]pyrimidine-5-carboxylate (**7a**) in DMSO-d_6 .... S52
82. ^{13}C{^{1}H} NMR spectrum of methyl 2-methyl-4-oxo-3,4-dihydrofuro[2,3-*d*]pyrimidine-5-carboxylate (**7a**) in DMSO-d_6 .... S52
83. ^{1}H-^{13}C HMQC spectrum of methyl 2-methyl-4-oxo-3,4-dihydrofuro[2,3-*d*]pyrimidine-5-carboxylate (**7a**) in DMSO-d_6 .... S53
84. ^{1}H-^{13}C HMBC spectrum of methyl 2-methyl-4-oxo-3,4-dihydrofuro[2,3-*d*]pyrimidine-5-carboxylate (**7a**) in DMSO-d_6 .... S53
85. ^{1}H NMR spectrum of ethyl 2-methyl-4-oxo-3,4-dihydrofuro[2,3-*d*]pyrimidine-5-carboxylate (**7b**) in DMSO-d_6 .... S54
86. ^{13}C{^{1}H} NMR spectrum of ethyl 2-methyl-4-oxo-3,4-dihydrofuro[2,3-*d*]pyrimidine-5-carboxylate (**7b**) in DMSO-d_6 .... S54
87. ^{1}H-^{13}C HMQC spectrum of ethyl 2-methyl-4-oxo-3,4-dihydrofuro[2,3-*d*]pyrimidine-5-carboxylate (**7b**) in DMSO-d_6 .... S55
88. ^{1}H-^{13}C HMBC spectrum of ethyl 2-methyl-4-oxo-3,4-dihydrofuro[2,3-*d*]pyrimidine-5-carboxylate (**7b**) in DMSO-d_6 .... S55
89. ^{1}H NMR spectrum of methyl 2-(methylsulfanyl)-4-oxo-3,4-dihydrofuro[2,3-*d*]pyrimidine-5-carboxylate (**7c**) in DMSO-d_6 .... S56
90. ^{13}C{^{1}H} NMR spectrum of methyl 2-(methylsulfanyl)-4-oxo-3,4-dihydrofuro[2,3-*d*]pyrimidine-5-carboxylate (**7c**) in DMSO-d_6 .... S56
91. ^{1}H-^{13}C HMQC spectrum of methyl 2-(methylsulfanyl)-4-oxo-3,4-dihydrofuro[2,3-*d*]pyrimidine-5-carboxylate (**7c**) in DMSO-d_6 .... S57
92. ^{1}H-^{13}C HMBC spectrum of methyl 2-(methylsulfanyl)-4-oxo-3,4-dihydrofuro[2,3-*d*]pyrimidine-5-carboxylate (**7c**) in DMSO-d_6 .... S57
93. ^{1}H NMR spectrum of ethyl 2-(methylsulfanyl)-4-oxo-3,4-dihydrofuro[2,3-*d*]pyrimidine-5-carboxylate (**7d**) in DMSO-d_6 .... S 58
94. ^{13}C{^{1}H} NMR spectrum of ethyl 2-(methylsulfanyl)-4-oxo-3,4-dihydrofuro[2,3-*d*]pyrimidine-5-carboxylate (**7d**) in DMSO-d_6 .... S58
95. ^{1}H-^{13}C HMQC spectrum of ethyl 2-(methylsulfanyl)-4-oxo-3,4-dihydrofuro[2,3-*d*]pyrimidine-5-carboxylate (**7d**) in DMSO-d_6 .... S59
96. ^{1}H-^{13}C HMBC spectrum of ethyl 2-(methylsulfanyl)-4-oxo-3,4-dihydrofuro[2,3-*d*]pyrimidine-5-carboxylate (**7d**) in DMSO-d_6 .... S59
97. ^{1}H NMR spectrum of methyl 4-oxo-2-phenyl-3,4-dihydrofuro[2,3-*d*]pyrimidine-5-carboxylate (**7e**) in DMSO-d_6 .... S60
98. ^{13}C{^{1}H} NMR spectrum of methyl 4-oxo-2-phenyl-3,4-dihydrofuro[2,3-*d*]pyrimidine-5-carboxylate (**7e**) in DMSO-d_6 .... S60
99. ^{1}H-^{13}C HMQC spectrum of methyl 4-oxo-2-phenyl-3,4-dihydrofuro[2,3-*d*]pyrimidine-5-carboxylate (**7e**) in DMSO-d_6 .... S61
100. ^{1}H-^{13}C HMBC spectrum of methyl 4-oxo-2-phenyl-3,4-dihydrofuro[2,3-*d*]pyrimidine-5-carboxylate (**7e**) in DMSO-d_6 .... S61
101. ^{1}H NMR spectrum of ethyl 4-oxo-2-phenyl-3,4-dihydrofuro[2,3-*d*]pyrimidine-5-carboxylate (**7f**) in DMSO-d_6 .... S62
102. ^{13}C{^{1}H} NMR spectrum of ethyl 4-oxo-2-phenyl-3,4-dihydrofuro[2,3-*d*]pyrimidine-5-carboxylate (**7f**) in DMSO-d_6 .... S62
103. ^{1}H-^{13}C HMQC spectrum of ethyl 4-oxo-2-phenyl-3,4-dihydrofuro[2,3-*d*]pyrimidine-5-carboxylate (**7f**) in DMSO-d_6 .... S63
104. ^{1}H-^{13}C HMBC spectrum of ethyl 4-oxo-2-phenyl-3,4-dihydrofuro[2,3-*d*]pyrimidine-5-carboxylate (**7f**) in DMSO-d_6 .... S63
105. Luminescence excitation spectrum of compound **6c** S64
106. Luminescence excitation spectrum of compound **6d** S64

Table S1. Principal crystallographic parameters of compound **5a**, **5b**, **6b**, **6c**, **7a** S65
Table S2. Torsion angles (τ) in the molecule of compounds **5a**, **5b** S67
Table S3. Angles (τ) in the molecule of compounds **5a**, **5b** S69
Table S4. Bond lengths (*d*) in the molecule of compounds **5a**, **5b** S71
Table S5. Torsion angles (τ) in the molecule of compounds **6b**, **6c** S72
Table S6. Angles (τ) in the molecule of compounds **6b**, **6c** S74
Table S7. Bond lengths (*d*) in the molecule of compounds **6b**, **6c** S76
Table S8. Torsion angles (τ) in the molecule of compound **7a** S77
Table S9. Angles (τ) in the molecule of compound **7a** S78
Table S10. Bond lengths (*d*) in the molecule of compound **7a** S79
References.. S79

Procedure for the synthesis of CH-acids 2a–g

2-Hydroxynaphthalene-1,4-dione (2a) was obtained according to a previously published procedure [1].

4-Hydroxy-7,7-dimethyl-7,8-dihydro-2*H*-1-benzopyran-2,5(6*H*)-dione (2b) [2].

4-Hydroxy-7-methyl-2*H*,5*H*-pyrano[4,3-*b*]pyran-2,5-dione (2c). A mixture of 4-hydroxy-6-methyl-2*H*-pyran-2-one (1 g, 7.9 mmol) and 2,2-dimethyl-1,3-dioxane-4,6-dione (2.28 g, 15.8 mmol) [3] in *o*-xylene (2 mL) was heated at 140 °C for 30 min. The cooled mixture was filtered, washed with EtOH and recrystallized from glacial AcOH. Yield 0.86 g (56%), orange crystals, mp 228–231 °C (AcOH), lit. mp 225 °C, [4]. IR spectrum (KBr), ν, cm^{-1}: 1691, 1761 (s, C=O), 3226 (m, OH). ^{1}H NMR spectrum (DMSO-d_6), δ, ppm (*J*, Hz): 2.27 (3H, s, CH_3); 5.39 (1H, s, H-3); 6.55 (1H, s, H-8); 11.94 (1H, br. s, OH). ^{13}C NMR spectrum (DMSO-d_6), δ, ppm: 20.3 (CH_3); 89.4 (C-3); 96.5 (C-4a); 99.7 (C-8); 158.7 (C-8a); 160.5 (C-4); 167.2 (C-7); 168.0 (C-5); 168.3 (C-2).

4-Hydroxy-2*H*,5*H*-pyrano[3,2-*c*][1]benzopyran-2,5-dione (2d). A mixture of 4-hydroxy-2*H*-1-benzopyran-2-one (2 g, 12.35 mmol) and 2,2-dimethyl-1,3-dioxane-4,6-dione (3.56 g, 24.69 mmol) in *o*-xylene (3 mL) was heated at 140 °C for 30 min. The cooled mixture was filtered, washed with EtOH and recrystallized from glacial AcOH. Yield 1.23 g (43%), orange crystals, mp 249–254 °C (AcOH), lit. mp 242-244 ($CHCl_3$) [5]. IR spectrum (KBr), ν, cm^{-1}: 1699, 1742 (s, C=O), 3249 (m, OH). ^{1}H NMR spectrum (DMSO-d_6), δ, ppm (*J*, Hz): 7.47 (1H, d. t, ^{3}J = 8.0, ^{4}J = 1.0 Hz, H-9); 7.49 (1H, d. d, ^{3}J = 8.0, ^{4}J = 1.0 Hz,H-10); 7.78 (1H, d. t, ^{3}J = 8.0, ^{4}J = 1.6 Hz, H-8); 7.97 (1H, d. d, ^{3}J = 8.0, ^{4}J = 1.6 Hz, H-7); 12.11 (1H, br. s, OH). ^{13}C NMR spectrum (DMSO-d_6), δ, ppm: 90.9 (C-3); 99.2 (C-4a); 113.5 (C-10a); 117.4 (C-10); 124.3 (C-7); 125.8 (C-9); 135.5 (C-8); 153.3 (C-6a); 157.7 (C-10b); 160.2 (C-4); 163.4 (C-5); 168.4 (C-2).

2-Methylpyrimidine-4,6-diol (2e), **2-(methylsulfanyl)pyrimidine-4,6-diol (2f)**, and **2-phenylpyrimidine-4,6-diol (2g)** were prepared according to the literature procedure [6,7].

Procedure for the synthesis of condensed furancarboxylates 3–7

Methyl 4,9-dioxo-4,9-dihydronaphtho[2,3-*b*]furan-3-carboxylate (3a), methyl 4,5-dioxo-4,5-dihydronaphtho[1,2-b]furan-3-carboxylate (4a). A solution of bromonitroacrylate **1a** (200 mg, 0.89 mmol) in anhydrous MeOH (5 mL) was added to a suspension of 2-hydroxynaphthalene-1,4-dione (**2a**, 155 mg, 0.89 mmol) and freshly molten AcOK (131 mg, 1.34 mmol) in anhydrous MeOH (5 mL). The resulted mixture was stirred at 18–20 °C for 3 h and then poured on crushed ice. The formed solid was filtered off. Yield 165 mg (68%), orange powder, mp 145-150°C (MeOH), (ratio **3a**:**4a** ≈ 2:1, by the ^{1}H NMR spectra). Found, %: C 66.01; H 3.54. $C_{14}H_8O_5$. Calculated, %: C 65.63; H 3.15.

A mixture of isomers was separated into individual substances by preparative chromatography (eluent – benzene).

Methyl 4,9-dioxo-4,9-dihydronaphtho[2,3-*b*]furan-3-carboxylate (3a). Yield 85 mg (35%), yellow powder, mp 165-169 °C. IR spectrum (KBr), ν, cm^{-1}: 1562 (m, C=C), 1684 (s), 1738 (s, C=O). ^{1}H NMR spectrum ($CDCl_3$), δ, ppm (*J*, Hz): 3.96 (3H, s, OC<u>H</u>$_3$); 7.74-7.82 (2H, m, Ar); 8.19-8.25 (2H, m, Ar); 8.28 (1H, s, C^2H). ^{13}C NMR spectrum ($CDCl_3$), δ, ppm: 52.6

(O<u>C</u>H_3); 118.6 (C-3); 126.8 (Ar); 127.0 (C-3a); 127.7 (Ar); 131.6 (Ar); 133.8 (Ar); 134.0 (Ar); 134.6 (Ar); 152.8 (C-2); 154.0 (C-9a); 161.0 (C=O ester); 173.9; 178.5 (C=O).

Methyl 4,5-dioxo-4,5-dihydronaphtho[1,2-b]furan-3-carboxylate (4a). Yield 39 mg (16%), orange powder. IR spectrum (KBr), ν, cm^{-1}: 1541 (m, C=C), 1684 (s), 1707 (s, C=O). ^{1}H NMR spectrum ($CDCl_3$), δ, ppm (*J*, Hz): 3.93 (3H, s, OC<u>H</u>$_3$); 7.53 (1H, d. t, ^3J = 7.6, ^4J = 1.2 Hz, C^7H); 7.70 (1H, d. t, ^3J = 7.6, ^4J = 1.3 Hz, C^8H); 7.79 (1H, d. d. d, ^3J = 7.7, ^4J = 1.0, ^5J = 0.4 Hz, C^9H); 8.08 c (1H, s, C^2H); 8.12 (1H, d. d. d, ^3J = 7.7, ^4J = 1.2, ^5J = 0.4 Hz, C^6H). ^{13}C NMR spectrum ($CDCl_3$), δ, ppm: 52.5 (O<u>C</u>H_3); 118.5 (C-3); 119.3 (C-3a); 122.8 (C-9); 127.9 (C-9a); 129.0 (C-5a); 130.7 (C-6); 131.0 (C-7); 135.6 (C-8); 149.9 (C-2); 161.5 (C=O ester); 161.6 (C-9b); 173.1 (C^4=O); 179.8 (C^5=O).

Ethyl 4,9-dioxo-4,9-dihydronaphtho[2,3-*b*]furan-3-carboxylate (3b), ethyl 4,5-dioxo-4,5-dihydronaphtho[1,2-b]furan-3-carboxylate (4b). A solution of bromonitroacrylate **1b** (300 mg, 1.34 mmol) in anhydrous MeOH (5 mL) was added to a suspension of 2-hydroxynaphthalene-1,4-dione (**2a**, 233 mg,1.34 mmol) and freshly molten AcOK (197 mg, 2.01 mmol) in anhydrous MeOH (5 mL). The resulted mixture was stirred at 18–20 °C for 3 h and then poured on crushed ice. The formed solid was filtered off. Yield 263 mg (73%), orange powder, mp 111-113°C (EtOH), (ratio **3b**:**4b** ≈ 2:1, by the ^{1}H NMR spectra). Found, %: C 66.98; H 4.08. $C_{15}H_{10}O_5$. Calculated, %: C 66.67; H 3.73.

A mixture of isomers was separated into individual substances by preparative chromatography (eluent – benzene).

Ethyl 4,9-dioxo-4,9-dihydronaphtho[2,3-*b*]furan-3-carboxylate (3b). Yield 136 mg (37%), yellow powder, mp 125-130 °C. IR spectrum (KBr), ν, cm^{-1}: 1563 (m, C=C), 1674 (s), 1736 (s, C=O). ^{1}H NMR spectrum ($CDCl_3$), δ, ppm (*J*, Hz): 1.42 (3H, t, ^3J = 7.1 Hz, OCH_2C<u>H</u>$_3$); 4.42 (2H, q, ^3J = 7.1 Hz, OC<u>H</u>$_2CH_3$); 7.72-7.82 (2H, m, Ar); 8.16-8.25 (2H, m, Ar); 8.26 (1H, s, C^2H). ^{13}C NMR spectrum ($CDCl_3$), δ, ppm: 14.3 (OCH_2<u>C</u>H_3); 61.8 (O<u>C</u>H_2CH_3); 119.1 (C-3); 126.7 (Ar); 127.1 (C-3a); 127.8 (Ar); 131.6 (Ar); 133.8 (Ar); 133.9 (Ar); 134.5 (Ar); 152.6 (C-2); 154.0 (C-9a); 160.5 (C=O ester); 173.9, 178.5 (C=O).

Ethyl 4,5-dioxo-4,5-dihydronaphtho[1,2-b]furan-3-carboxylate (4b). Yield 52 mg (14%), orange powder, mp 130-135°C. IR spectrum (KBr), ν, cm^{-1}: 1542 (m, C=C), 1683 (s), 1703 (s), 1711 (s, C=O). ^{1}H NMR spectrum ($CDCl_3$), δ, ppm (*J*, Hz): 1.41 (3H, t, OCH_2C<u>H</u>$_3$, ^3J = 7.2 Hz); 4.39 (2H, q, ^3J = 7.2 Hz, OC<u>H</u>$_2CH_3$); 7.53 (1H, d. t, ^3J = 7.6, ^4J = 1.2 Hz, C^7H); 7.70 (1H, d. t, ^3J = 7.6, ^4J = 1.3 Hz, C^8H); 7.78 (1H, d. d. d, ^3J = 7.7, ^4J = 1.2, ^5J = 0.5 Hz, C^9H); 8.08 c (1H, s, C^2H); 8.12 (1H, d. d. d, ^3J = 7.7, ^4J = 1.3, ^5J = 0.5 Hz, C^6H). ^{13}C NMR spectrum ($CDCl_3$), δ, ppm: 14.2 (OCH_2<u>C</u>H_3); 61.6 (O<u>C</u>H_2CH_3); 118.5 (C-3); 119.7 (C-3a); 122.8 (C-9); 128.0 (C-9a); 129.0 (C-5a); 130.7 (C-6); 131.0 (C-7); 135.6 (C-8); 149.8 (C-2); 161.1 (C=O ester); 161.6 (C-9b); 173.0 (C^4=O); 179.8 (C^5=O).

Methyl 7,7-dimethyl-4,9-dioxo-6,7,8,9-tetrahydro-4*H*-furo[3,2-*c*][1]benzopyran-3-carboxylate (5a). To a solution of 4-hydroxy-7,7-dimethyl-7,8-dihydro-2*H*-1-benzopyran-2,5(6*H*)-dione (**2b**, 195 mg, 0.95 mmol) and freshly molten AcOK (140 mg,1.43 mmol) in anhydrous MeOH (5 mL), a solution of bromonitroacrylate **1a** (200 mg, 0.95 mmol) in anhydrous MeOH (5 mL) was added. The resulted mixture was stirred at 18–20 °C for 1 h. The formed precipitate was filtered off. The mother liquor was evaporated, and an additional amount of crystalline product was obtained. Yield 230 mg (85%), colorless powder, mp 194-198°C (EtOH). IR spectrum (KBr), ν, cm^{-1}: 1678 (s), 1714 (s), 1769 (s), 1786 (s, C=O). ^{1}H NMR spectrum ($CDCl_3$), δ, ppm (*J*, Hz): 1.16 (6H, s, $2CH_3$); 2.49 (2H, s, C^8H_2); 2.80 (2H, s, C^6H_2); 3.90 (3H, s, OCH_3); 8.16 (1H, s, C^2H). ^{13}C NMR spectrum ($CDCl_3$), δ, ppm: 28.3

(CH_3); 32.7 (C-7); 41.9 (C-6); 51.4 (C-8); 52.5 (OCH_3); 106.6 (C-3a); 107.2 (C-9a); 116.9 (C-3); 150.2 (C-2); 154.9 (C^4=O); 159.1 (C-9b); 161.1 (C=O ester); 172.4 (C-5a); 192.3 (C^9=O). Found, %: C 61.95; H 4.55. $C_{15}H_{14}O_6$. Calculated, %: C 62.07; H 4.86.

Ethyl 7,7-dimethyl-4,9-dioxo-6,7,8,9-tetrahydro-4H-furo[3,2-*c*][1]benzopyran-3-carboxylate (5b). To a solution of 4-hydroxy-7,7-dimethyl-7,8-dihydro-2*H*-1-benzopyran-2,5(6*H*)-dione (**2b**, 186 mg, 0.89 mmol) and freshly molten AcOK (131 mg,1.43 mmol) in anhydrous MeOH (5 mL), a solution of bromonitroacrylate **1b** (200 mg, 0.89 mmol) in anhydrous MeOH (5 mL) was added. The resulted mixture was stirred at 18–20 °C for 1 h. The formed precipitate was filtered off. The mother liquor was evaporated, and an additional amount of crystalline product was obtained. Yield 230 mg (84%), colorless powder, mp 193-198°C (EtOH). IR spectrum (KBr), ν, cm^{-1}: 1678 (s), 1731 (s), 1776 (s), 1791 (s, C=O). ^{1}H NMR spectrum ($CDCl_3$), δ, ppm (*J*, Hz): 1.17 (6H, s, 2CH_3); 1.38 (3H, t, ^3J = 7.2 Hz, $OCH_2C\underline{H}_3$); 2.50 (2H, s, C^8H_2); 2.81 (2H, s, C^6H_2); 4.38 (2H, q, ^3J = 7.2 Hz, $OC\underline{H}_2CH_3$); 8.15 (1H, s, C^2H). ^{13}C NMR spectrum ($CDCl_3$), δ, ppm: 14.3 ($OCH_2\underline{C}H_3$); 28.3 (CH_3); 32.7 (C-7); 41.9 (C-6); 51.4 (C-8); 61.6 ($O\underline{C}H_2CH_3$); 106.7 (C-3a); 107.2 (C-9a); 117.3 (C-3); 150.0 (C-2); 154.9 (C^4=O); 159.1 (C-9b); 160.6 (C=O ester); 172.3 (C-5a); 192.3 (C^9=O). Found, %: C 62.98; H 5.05. $C_{16}H_{16}O_6$. Calculated, %: C 63.15; H 5.30.

Methyl 7-methyl-4,9-dioxo-4*H*,9*H*-furo[2,3-*d*]pyrano[4,3-*b*]pyran-3-carboxylate (6a). A mixture of 4-hydroxy-7-methyl-2*H*,5*H*-pyrano[4,3-*b*]pyran-2,5-dione (**2c**, 80 mg, 0.414 mmol), freshly molten AcOK (61 mg, 0.622 mmol) and bromonitroacrylate **1a** (131 mg, 0.622 mmol) in anhydrous MeOH (20 mL) was stirred at 18–20 °C for 2 h. The formed precipitate was filtered off. Yield 47 mg (41%), orange powder, mp 281-284 °C (AcOH). IR spectrum (KBr), ν, cm^{-1}: 1713 (shoulder), 1720 (s), 1746 (shoulder), 1784 (s), 1799 (shoulder, C=O), 1560 (s), 1583 (m), 1627 (s, C=C). UV spectrum, $λ_{max}$, nm (ε, l·mol^{-1}·cm^{-1}): 295 (12762), 343 (17963). ^{1}H NMR spectrum (DMSO-d_6), δ, ppm (*J*, Hz): 2.33 (3H, br. s, CH_3); 3.80 (3H, s, OCH_3); 6.75 (1H, q, ^4J = 0.7 Hz, H-6); 8.77 (1H, s, H-2). ^{13}C NMR spectrum (DMSO-d_6), δ, ppm: 20.5 (CH_3); 52.7 (OCH_3); 94.9 (C-9a); 99.5 (C-6); 106.2 (C-3a); 117.0 (C-3); 151.9 (C-2); 153.9, 156.9 (C-4, C-9); 158.4 (C-9b); 161.1 (C=O ester); 165.4 (C-7); 166.5 (C-5a). Found, %: C 56.39; H 2.66. $C_{13}H_8O_7$. Calculated, %: C 56.53; H 2.92.

Ethyl 7-methyl-4,9-dioxo-4*H*,9*H*-furo[2,3-*d*]pyrano[4,3-*b*]pyran-3-carboxylate (6b). A mixture of 4-hydroxy-7-methyl-2*H*,5*H*-pyrano[4,3-*b*]pyran-2,5-dione (**2c**, 116 mg, 0.595 mmol), freshly molten AcOK (88 mg, 0.893 mmol) and bromonitroacrylate **1b** (200 mg, 0.893 mmol) in anhydrous MeOH (20 mL) was stirred at 18–20 °C for 2 h. The formed precipitate was filtered off. Yield 47 mg (41%), orange powder, mp 264-266 °C (AcOH). IR spectrum (KBr), ν, cm^{-1}: 1721 (s), 1730 (s), 1742 (shoulder), 1785 (s), 1801 (shoulder, C=O), 1549 (m), 1561 (s), 1584 (m), 1629 (m, C=C). UV spectrum, $λ_{max}$, nm (ε, l·mol^{-1}·cm^{-1}): 295 (10310), 343 (14448). ^{1}H NMR spectrum (DMSO-d_6), δ, ppm (*J*, Hz): 1.27 (3H, t, ^3J = 7.1 Hz, $OCH_2C\underline{H}_3$); 2.33 (3H, d, ^4J = 0.9 Hz, CH_3); 4.26 (2H, q, ^3J = 7.1 Hz, $OC\underline{H}_2CH_3$); 6.75 (1H, q, ^4J = 0.9 Hz, H-6); 8.76 (1H, s, H-2). ^{13}C NMR spectrum (DMSO-d_6), δ, ppm: 14.6 ($OCH_2\underline{C}H_3$); 20.5 (CH_3); 61.5 ($O\underline{C}H_2CH_3$); 94.9 (C-9a); 99.5 (C-6); 106.3 (C-3a); 117.3 (C-3); 151.7 (C-2); 153.9, 156.9 (C-4, C-9); 158.4 (C-9b); 160.7 (C=O ester); 165.4 (C-7); 166.4 (C-5a). Found, %: C 57.60; H 3.09. $C_{14}H_{10}O_7$. Calculated, %: C 57.94; H 3.47.

Methyl 4,11-dioxo-4*H*,11*H*-furo[2',3':4,5]pyrano[3,2-*c*]chromene-1-carboxylate (6c). A mixture of 4-hydroxy-2*H*,5*H*-pyrano[3,2-*c*][1]benzopyran-2,5-dione (**2d**, 146 mg,

0.635 mmol), freshly molten AcOK (93 mg, 0.952 mmol) and bromonitroacrylate **1a** (200 mg, 0.952 mmol) in anhydrous MeOH (20 mL) was stirred at 18–20 °C for 2 h. The formed precipitate was filtered off. Yield 100 mg (51%), orange powder, mp 288-290 °C (AcOH). IR spectrum (KBr), ν, cm^{-1}: 1720 (br. s), 1790 (s, C=O), 1548 (m), 1566 (m), 1583 (m), 1615 (s, C=C). UV spectrum, λ_{max}, nm (ε, l·mol^{-1}·cm^{-1}): 283 (10958), 352 (18109). ^{1}H NMR spectrum (DMSO-*d*$_6$), δ, ppm (*J*, Hz): 3.83 (3H, s, OCH_3); 7.51 (1H, d. t, $<{}^{3}J>$ = 7.6, ^{4}J = 0.9 Hz, H-8); 7.55 (1H, d, ^{3}J = 8.1 Hz, H-6); 7.80 (1H, d. t, $<{}^{3}J>$ = 7.8, ^{4}J = 1.5 Hz, H-7); 8.06 (1H, d. d, ^{3}J = 7.8, ^{4}J = 1.5 Hz, H-9); 8.88 (1H, s, H-2). ^{13}C NMR spectrum (DMSO-*d*$_6$), δ, ppm: 52.8 (OCH_3); 97.7 (C-3b); 107.7 (C-11a); 113.3 (C-9a); 117.1 (C-1); 117.7 (C-6); 124.2 (C-9); 126.1 (C-8); 135.2 (C-7); 152.5 (C-2); 153.1 (C-5a); 153.6, 155.6 (C-4, C-11); 158.4 (C-3a); 160.6 (C-9b); 161.0 (C=O ester). Found, %: C 61.18; H 2.23. $C_{16}H_8O_7$. Calculated, %: C 61.55; H 2.58.

Ethyl 4,11-dioxo-4*H*,11*H*-furo[2',3':4,5]pyrano[3,2-*c*]chromene-1-carboxylate (6d). A mixture of 4-hydroxy-2*H*,5*H*-pyrano[3,2-*c*][1]benzopyran-2,5-dione (**2d**, 137 mg, 0.595 mmol), freshly molten AcOK (88 mg, 0.893 mmol) and bromonitroacrylate **1b** (200 mg, 0.893 mmol) in anhydrous MeOH (20 mL) was stirred at 18–20 °C for 2 h. The formed precipitate was filtered off. Yield 87 mg (45%), orange powder, mp 242-244 °C (AcOH). IR spectrum (KBr), ν, cm^{-1}: 1725 (br. s), 1781 (s, C=O), 1546 (m), 1565 (m), 1579 (m), 1616 (s, C=C). UV spectrum, λ_{max}, nm (ε, l·mol^{-1}·cm^{-1}): 283 (11981), 352 (20357). ^{1}H NMR spectrum (DMSO-*d*$_6$), δ, ppm (*J*, Hz): 1.29 (3H, t, ^{3}J = 7.1, $OCH_2C\underline{H}_3$); 4.29 (2H, q, ^{3}J = 7.1 Hz, $OC\underline{H}_2CH_3$); 7.50 (1H, t, $<{}^{3}J>$ = 7.6 Hz, H-8); 7.54 (1H, d, ^{3}J = 8.3 Hz, H-6); 7.79 (1H, d. t, $<{}^{3}J>$ = 7.9, ^{4}J = 1.3 Hz, H-7); 8.04 (1H, d. d, ^{3}J = 7.9, ^{4}J = 1.3 Hz, H-9); 8.85 (1H, s, H-2). ^{13}C NMR spectrum (DMSO-*d*$_6$), δ, ppm: 14.6 ($OCH_2\underline{C}H_3$); 61.6 ($O\underline{C}H_2CH_3$); 97.6 (C-3b); 107.7 (C-11a); 113.3 (C-9a); 117.4 (C-1); 117.7 (C-6); 124.2 (C-9); 126.1 (C-8); 135.2 (C-7); 152.3 (C-2); 153.1 (C-5a); 153.6, 155.6 (C-4, C-11); 158.4 (C-3a); 160.6 (C-9b); 160.6 (C=O ester). Found, %: C 62.37; H 2.98. $C_{17}H_{10}O_7$. Calculated, %: C 62.58; H 3.09.

Methyl 2-methyl-4-oxo-3,4-dihydrofuro[2,3-*d*]pyrimidine-5-carboxylate (7a). A mixture of 2-methylpyrimidine-4,6-diol (**2e**, 120 mg, 0.95 mmol), AcOK (140 mg, 1.43 mmol) and bromonitroacrylate **1a** (200 mg, 0.95 mmol) was refluxed in a water-alcohol solution (H_2O:MeOH = 1:1, 10 mL) for 1 hour. The mother liquor was evaporated to a minimum amount and the precipitate was filtered off. Yield 126 mg (64%), white powder, mp 235-238 °C (H_2O). IR spectrum (KBr), ν, cm^{-1}: 3161 (m), 3021 (m, NH), 1750 (s), 1688 (s, C=O), 1595 (s), 1550 (s, C=C). ^{1}H NMR spectrum (DMSO-*d*$_6$), δ, ppm (*J*, Hz): 2.33 (3H, s, CH_3); 3.75 (3H, s, OCH_3); 8.38 (1H, s, H-6); 12.55 (1H, br. s, NH). ^{13}C NMR spectrum (DMSO-*d*$_6$), δ, ppm: 21.5 (CH_3); 52.4 (OCH_3); 102.1 (C-4a); 115.8 (C-5); 147.2 (C-6); 158.1 (C-4); 158.5 (C-2); 162.0 (C=O ester); 166.7 (C-7a). Found, %: C 51.65; H 3.56; N 13.11. $C_9H_8N_2O_4$. Calculated, %: C 51.93; H 3.87; N 13.46.

Ethyl 2-methyl-4-oxo-3,4-dihydrofuro[2,3-*d*]pyrimidine-5-carboxylate (7b). A mixture of 2-methylpyrimidine-4,6-diol (**2e**, 112 mg, 0.89 mmol), AcOK (131 mg, 1.34 mmol) and bromonitroacrylate **1b** (200 mg, 0.89 mmol) was refluxed in a water-alcohol solution (H_2O:EtOH = 1:1, 10 mL) for 1 hour. The mother liquor was evaporated to a minimum amount and the precipitate was filtered off. Yield 103 mg (52%), white powder, decomp. 240 °C (H_2O). IR spectrum (KBr), ν, cm^{-1}: 3144 (m), 3094 (m, NH), 1710 (s), 1680 (s, C=O), 1554 (s), 1506 (s, C=C). ^{1}H NMR spectrum (DMSO-*d*$_6$), δ, ppm (*J*, Hz): 1.25 (3H, t, ^{3}J = 7.1 Hz,

$OCH_2C\underline{H}_3$); 2.33 (3H, s, CH_3); 4.22 (2H, q, ^3J = 7.1 Hz, $OC\underline{H}_2CH_3$); 8.37 (1H, s, H-6); 12.49 (1H, br. s, NH). ^{13}C NMR spectrum (DMSO-d_6), δ, ppm: 14.7 ($OCH_2\underline{C}H_3$); 21.5 (CH_3); 61.0 ($O\underline{C}H_2CH_3$); 102.2 (C-4a); 116.1 (C-5); 147.1 (C-6); 158.1 (C-4); 158.5 (C-2); 161.5 (C=O ester); 166.6 (C-7a). Found, %: C 53.77; H 4.40; N 12.57. $C_{10}H_{10}N_2O_4$. Calculated, %: C 54.05; H 4.54; N 12.61.

Methyl 2-(methylsulfanyl)-4-oxo-3,4-dihydrofuro[2,3-*d*]pyrimidine-5-carboxylate (7c). A mixture of 2-(methylsulfanyl)pyrimidine-4,6-diol (**2f**, 226 mg, 1.43 mmol), freshly molten AcOK (210 mg, 2.14 mmol) and bromonitroacrylate **1a** (300 mg, 1.43 mmol) was refluxed in anhydrous MeOH (20 mL) for 1 hour. The mother liquor was evaporated to a minimum amount and the precipitate was filtered off. Yield 167 mg (49%), white powder, 227-230 °C (EtOH). IR spectrum (KBr), ν, cm^{-1}: 3135 (m), 3092 (m, NH), 1706 (s), 1674 (s, C=O), 1573 (s), 1554 (s, C=C). ^1H NMR spectrum (DMSO-d_6), δ, ppm (*J*, Hz): 2.49 (3H, s, CH_3); 3.75 (3H, s, OCH_3); 8.31 (1H, s, H-6); 12.84 (1H, br. s, NH). ^{13}C NMR spectrum (DMSO-d_6), δ, ppm: 13.5 (CH_3); 52.3 (OCH_3); 100.5 (C-4a); 115.9 (C-5); 146.5 (C-6); 158.3 (C-4); 162.0 (C-2); 161.8 (C=O ester); 166.3 (C-7a). Found, %: C 44.91; H 3.22; N 11.81. $C_9H_8N_2O_4S$. Calculated, %: C 45.00; H 3.36; N 11.66.

Ethyl 2-(methylsulfanyl)-4-oxo-3,4-dihydrofuro[2,3-*d*]pyrimidine-5-carboxylate (7d). A mixture of 2-(methylsulfanyl)pyrimidine-4,6-diol (**2f**, 212 mg, 1.34 mmol), freshly molten AcOK (197 mg, 2.01 mmol) and bromonitroacrylate **1b** (300 mg, 1.34 mmol) was refluxed in anhydrous MeOH (20 mL) for 1 hour. The mother liquor was evaporated to a minimum amount and the precipitate was filtered off. Yield 159 mg (47%), white powder, 199-201 °C (EtOH). IR spectrum (KBr), ν, cm^{-1}: 3147 (m), 3090 (m, NH), 1714 (s), 1681 (s, C=O), 1574 (s), 1553 (s, C=C). ^1H NMR spectrum (DMSO-d_6), δ, ppm (*J*, Hz): 1.25 (3H, t, ^3J = 7.1 Hz, $OCH_2C\underline{H}_3$); 2.47 (3H, s, CH_3); 4.21 (2H, q, ^3J = 7.1 Hz, $OC\underline{H}_2CH_3$); 8.26 (1H, s, H-6); 12.84 (1H, br. s, NH). ^{13}C NMR spectrum (DMSO-d_6), δ, ppm: 14.7 ($OCH_2\underline{C}H_3$); 13.5 (CH_3); 60.9 ($O\underline{C}H_2CH_3$); 100.4 (C-4a); 116.2 (C-5); 146.0 (C-6); 159.0 (C-4); 162.1 (C-2); 161.6 (C=O ester); 166.5 (C-7a). Found, %: C 46.85; H 7.76; N 10.96. $C_{10}H_{10}N_2O_4S$. Calculated, %: C 47.24; H 3.96; N 11.02.

Methyl 4-oxo-2-phenyl-3,4-dihydrofuro[2,3-*d*]pyrimidine-5-carboxylate (7e). A mixture of 2-phenylpyrimidine-4,6-diol (**2g**, 269 mg, 1.43 mmol), freshly molten AcOK (210 mg, 2.14 mmol) and bromonitroacrylate **1a** (300 mg, 1.43 mmol) was refluxed in anhydrous MeOH (20 mL) for 3 h. The mother liquor was evaporated to a minimum amount and the precipitate was filtered off. Yield 166 mg (43%), white powder, decomp. 230 °C (*o*-xylene). IR spectrum (KBr), ν, cm^{-1}: 3452 (m, NH), 1743 (s), 1684 (s, C=O), 1568 (m), 1541 (s, C=C). ^1H NMR spectrum (DMSO-d_6), δ, ppm (*J*, Hz): 3.79 (3H, s, OCH_3); 7.52 (2H, t, ^3J = 7.4 Hz, H^m); 7.58 (1H, t, ^3J = 7.3 Hz, H^p); 8.12 (2H, d, ^3J = 8.1 Hz, H^o); 8.48 (1H, s, H-6); 12.67 (1H, br. s, NH). ^{13}C NMR spectrum (DMSO-d_6), δ, ppm: 52.4 (OCH_3); 102.9 (C-4a); 116.0 (C-5); 128.5 (C^o); 129.3 (C^m); 132.2 (C^i); 132.5 (C^p); 148.0 (C-6); 156.1 (C-2); 158.8 (C-4); 162.0 (C=O ester); 166.7 (C-7a). Found, %: C 62.05; H 3.60; N 10.17. $C_{14}H_{10}N_2O_4$. Calculated, %: C 62.22; H 3.73; N 10.37.

Ethyl 4-oxo-2-phenyl-3,4-dihydrofuro[2,3-*d*]pyrimidine-5-carboxylate (7f). A mixture of 2-phenylpyrimidine-4,6-diol (**2g**, 252 mg, 1.34 mmol), freshly molten AcOK (197 mg, 2.01 mmol) and bromonitroacrylate **1b** (300 mg, 1.34 mmol) was refluxed in

anhydrous MeOH (20 mL) for 2 h. The mother liquor was evaporated to a minimum amount and the precipitate was filtered off. Yield 126 mg (50%), white powder, 240-245 °C (EtOH). IR spectrum (KBr), ν, cm^{-1}: 3152 (m), 3075 (m), 2984 (m, NH), 1733 (s), 1684 (s, C=O), 1573 (m), 1540 (s), 1510 (m, C=C). ^{1}H NMR spectrum (DMSO-d_6), δ, ppm (*J*, Hz): 1.28 (3H, t, ^{3}J = 7.1 Hz, $OCH_2C\underline{H}_3$); 4.25 (2H, q, ^{3}J = 7.1 Hz, $OC\underline{H}_2CH_3$); 7.52 (2H, t, ^{3}J = 7.4 Hz, H^m); 7.58 (1H, t, ^{3}J = 7.3 Hz, H^p); 8.11 (2H, d, ^{3}J = 8.1 Hz, H^o); 8.46 (1H, s, H-6); 12.78 (1H, br. s, NH). ^{13}C NMR spectrum (DMSO-d_6), δ, ppm: 14.7 ($OCH_2\underline{C}H_3$); 61.1 ($O\underline{C}H_2CH_3$); 103.0 (C-4a); 116.4 (C-5); 128.5 (C^o); 129.3 (C^m); 132.0 (C^i); 132.5 (C^p); 147.8 (C-6); 155.9 (C-2); 158.6 (C-4); 161.4 (C=O ester); 166.6 (C-7a). Found, %: C 63.12; H 3.96; N 9.56. $C_{15}H_{12}N_2O_4$. Calculated, %: C 63.38; H 4.26; N 9.85.

Spectra of the starting acids

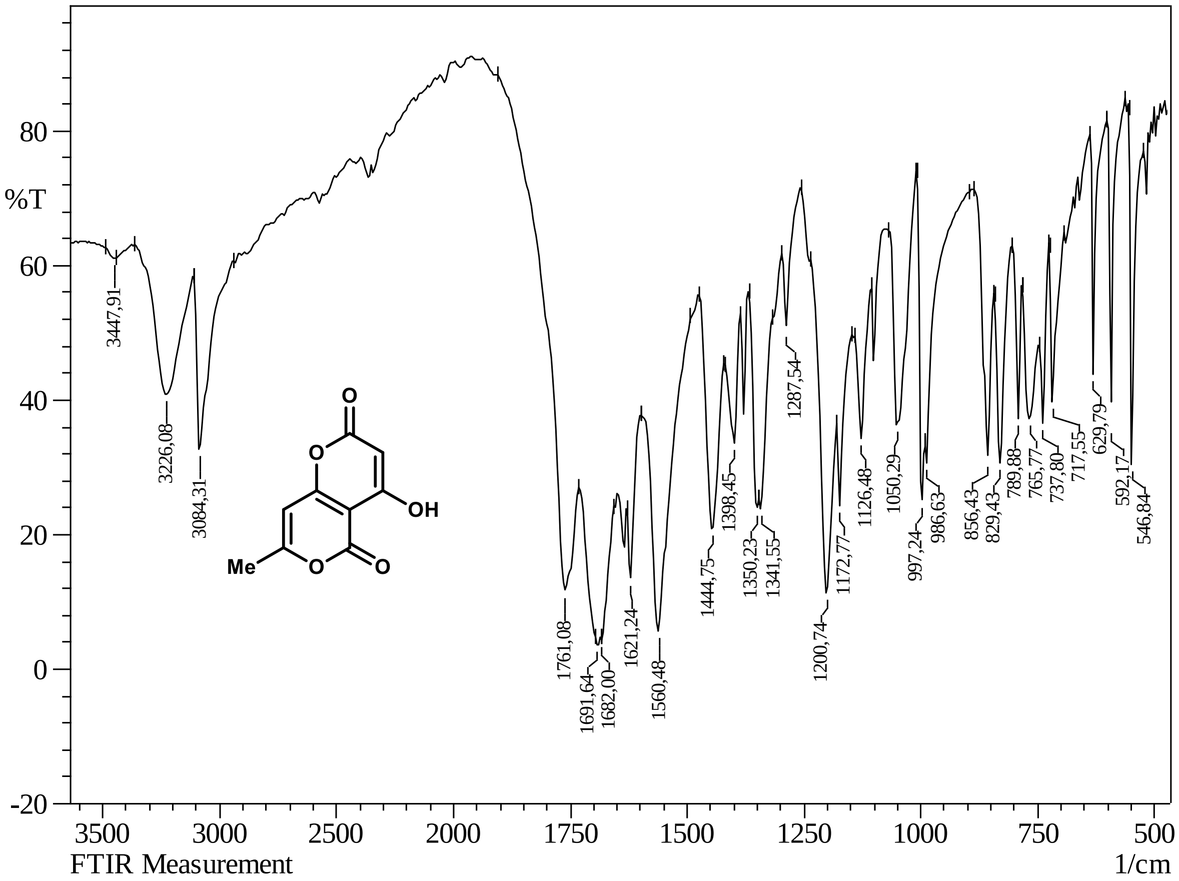

Figure S1. IR spectrum of 4-hydroxy-7-methyl-2*H*,5*H*-pyrano[4,3-*b*]pyran-2,5-dione (**2c**) in KBr

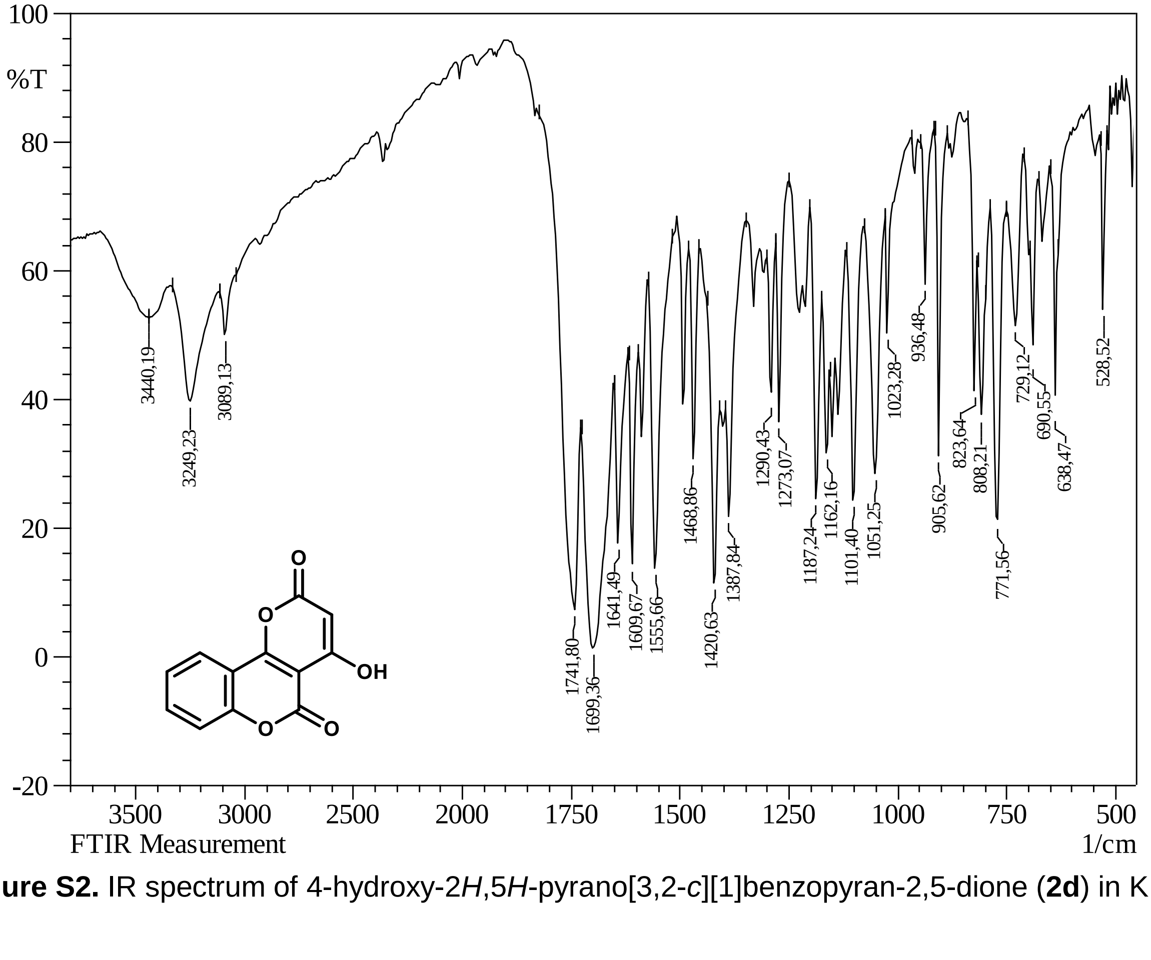

Figure S2. IR spectrum of 4-hydroxy-2*H*,5*H*-pyrano[3,2-*c*][1]benzopyran-2,5-dione (**2d**) in KBr

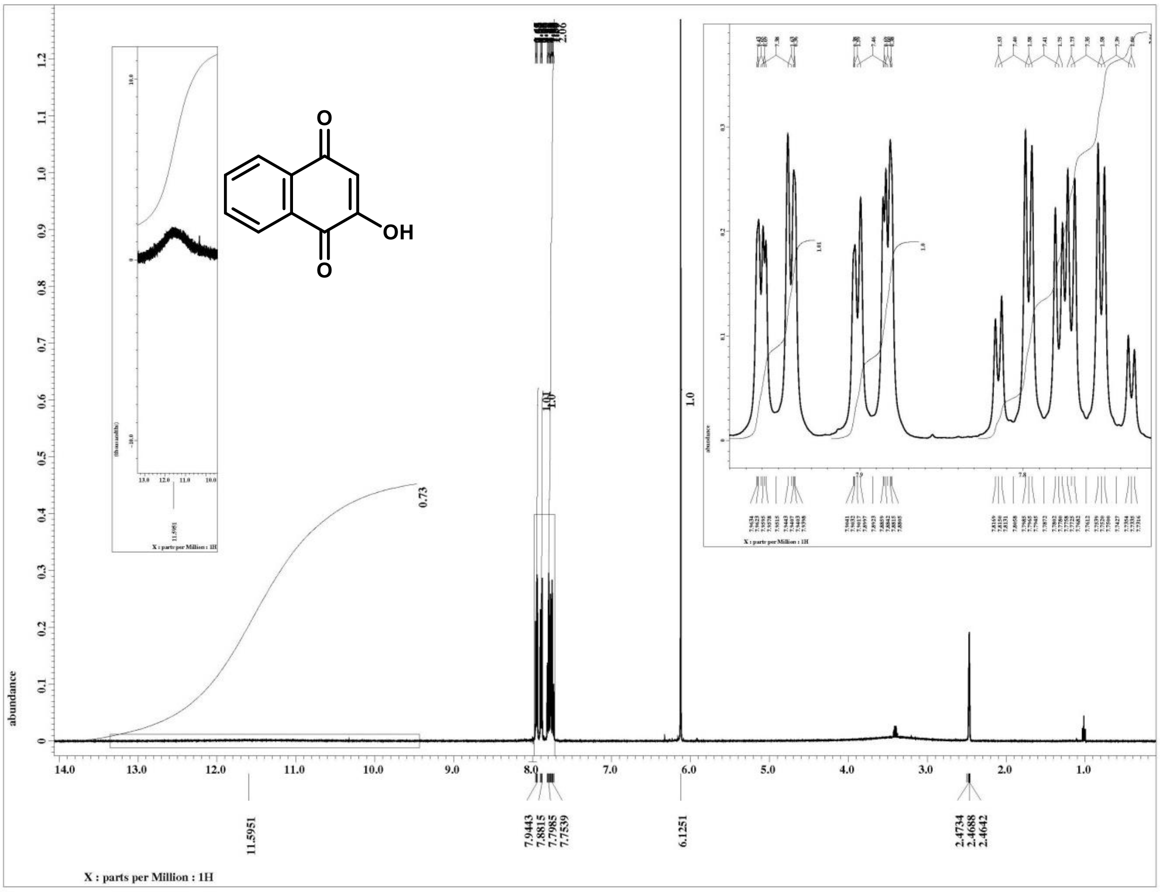

Figure S3. ^{1}H NMR spectrum of 2-hydroxynaphthalene-1,4-dione (**2a**) in DMSO-d_6

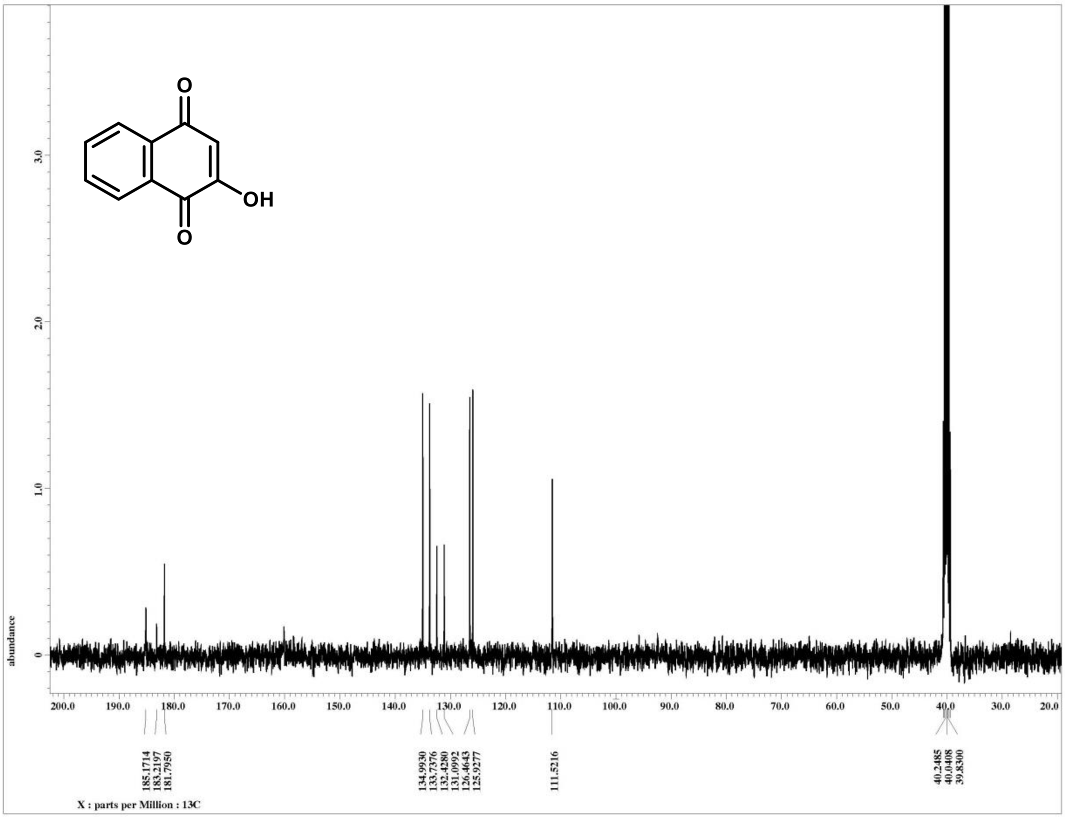

Figure S4. $^{13}C\{^{1}H\}$ NMR spectrum of 2-hydroxynaphthalene-1,4-dione (**2a**) in DMSO-d_6

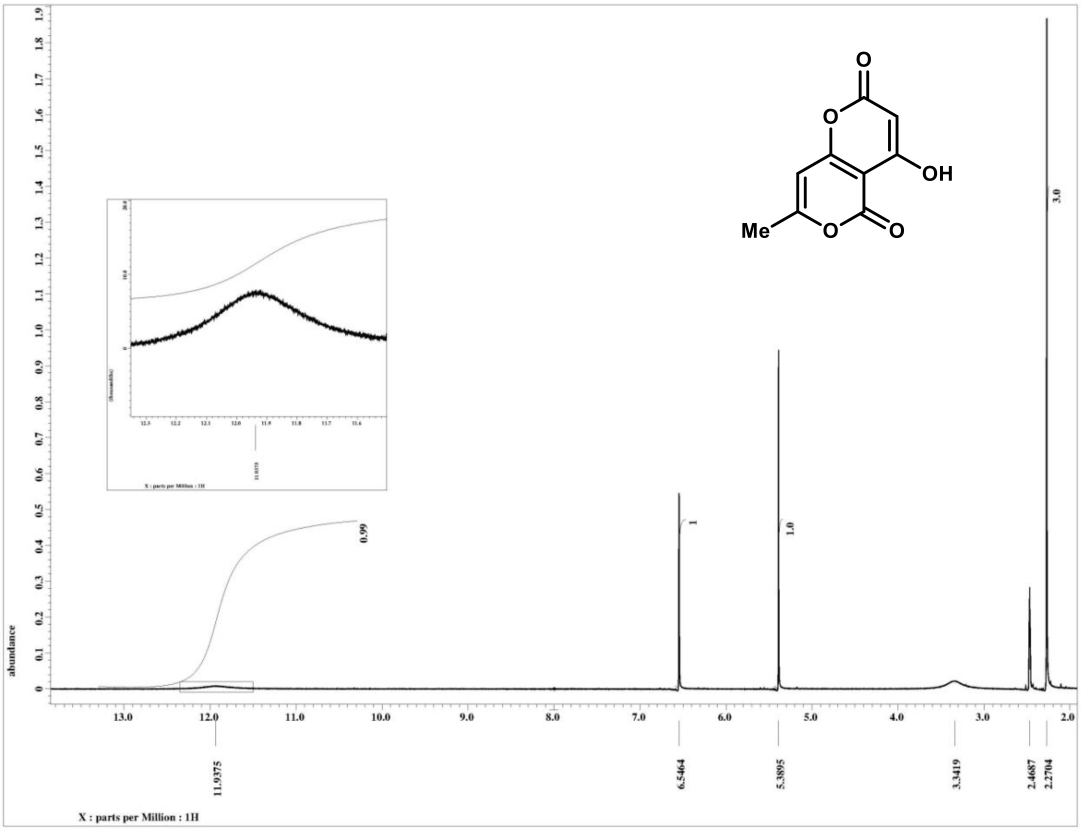

Figure S5. ^{1}H NMR spectrum of 4-hydroxy-7-methyl-2*H*,5*H*-pyrano[4,3-*b*]pyran-2,5-dione (**2c**) in DMSO-d_6

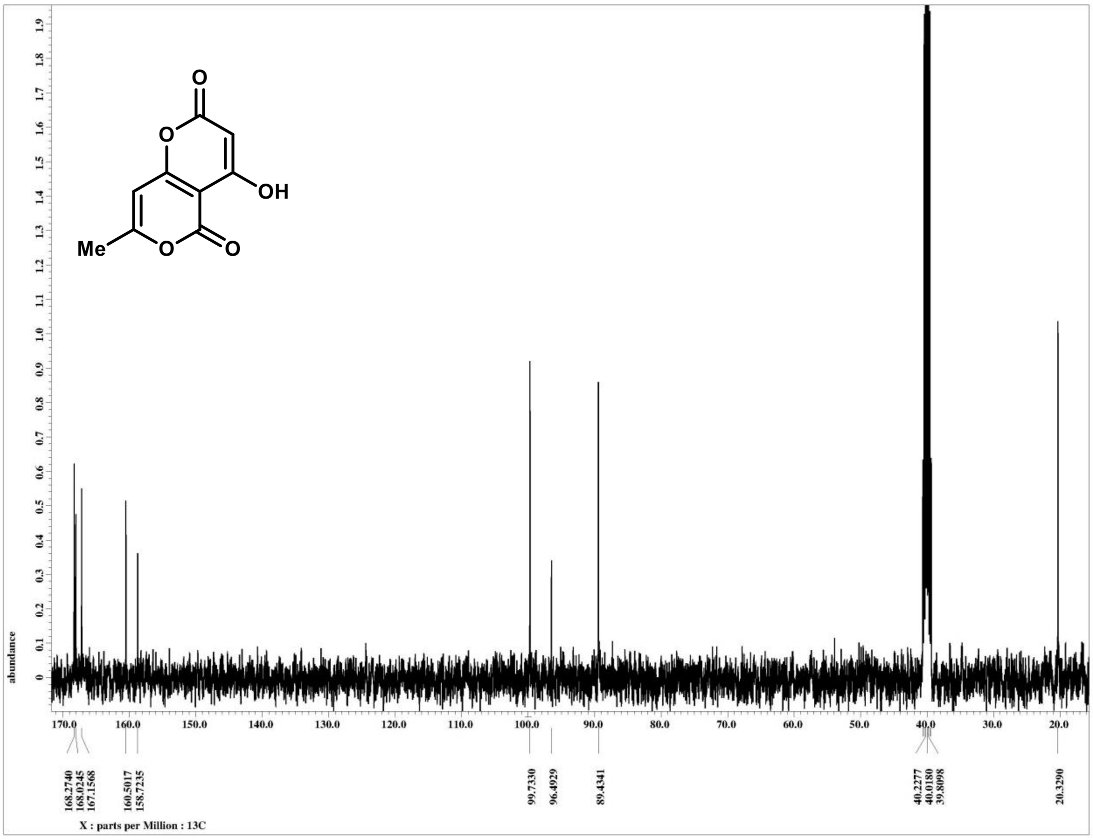

Figure S6. ^{13}C{^{1}H} NMR spectrum of 4-hydroxy-7-methyl-2*H*,5*H*-pyrano[4,3-*b*]pyran-2,5-dione (**2c**) in DMSO-d_6

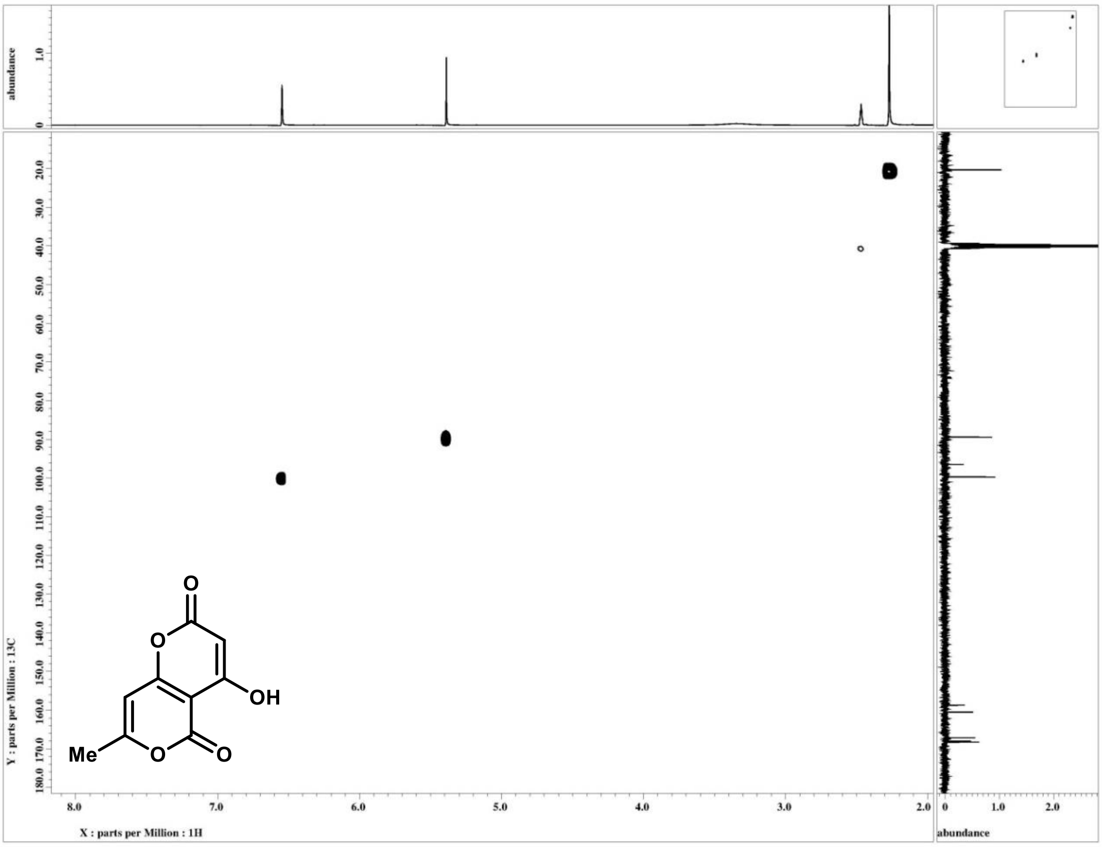

Figure S7. ^{1}H-^{13}C HMQC spectrum of 4-hydroxy-7-methyl-2*H*,5*H*-pyrano[4,3-*b*]pyran-2,5-dione (**2c**) in DMSO-d_6

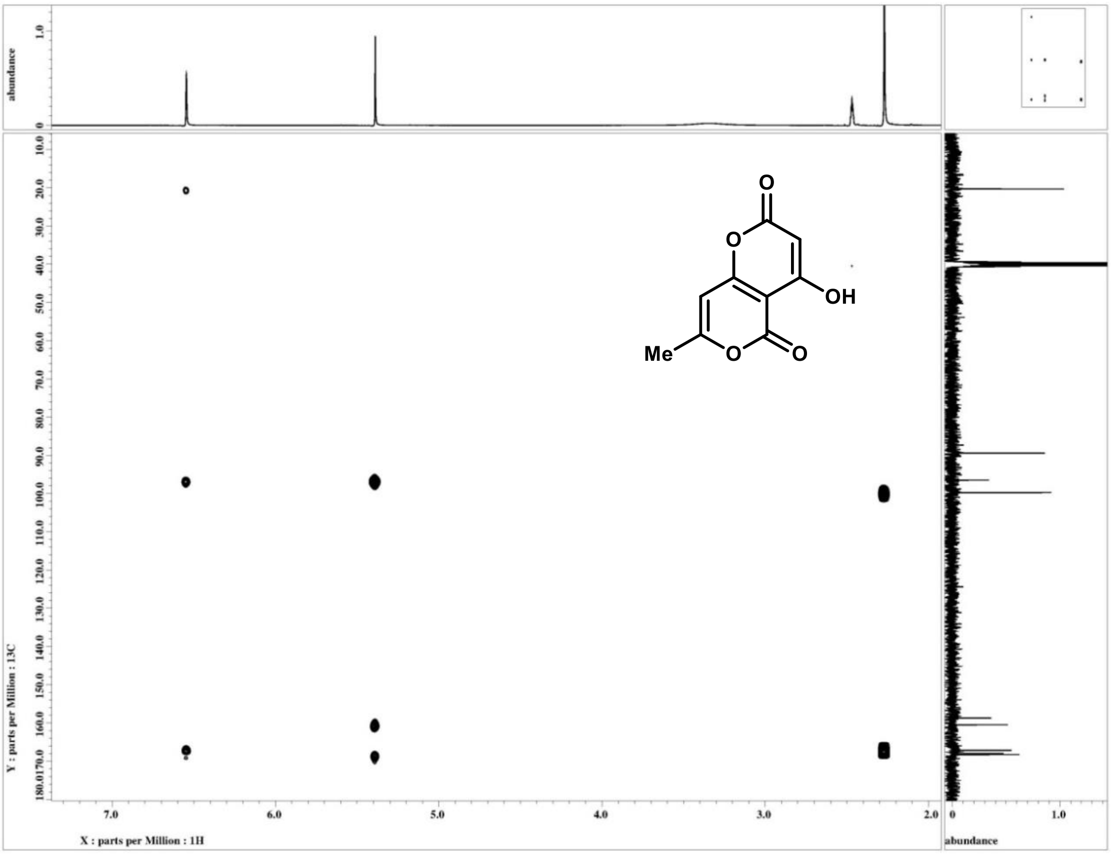

Figure S8. ^{1}H-^{13}C HMBC spectrum of 4-hydroxy-7-methyl-2*H*,5*H*-pyrano[4,3-*b*]pyran-2,5-dione (**2c**) in DMSO-d_6

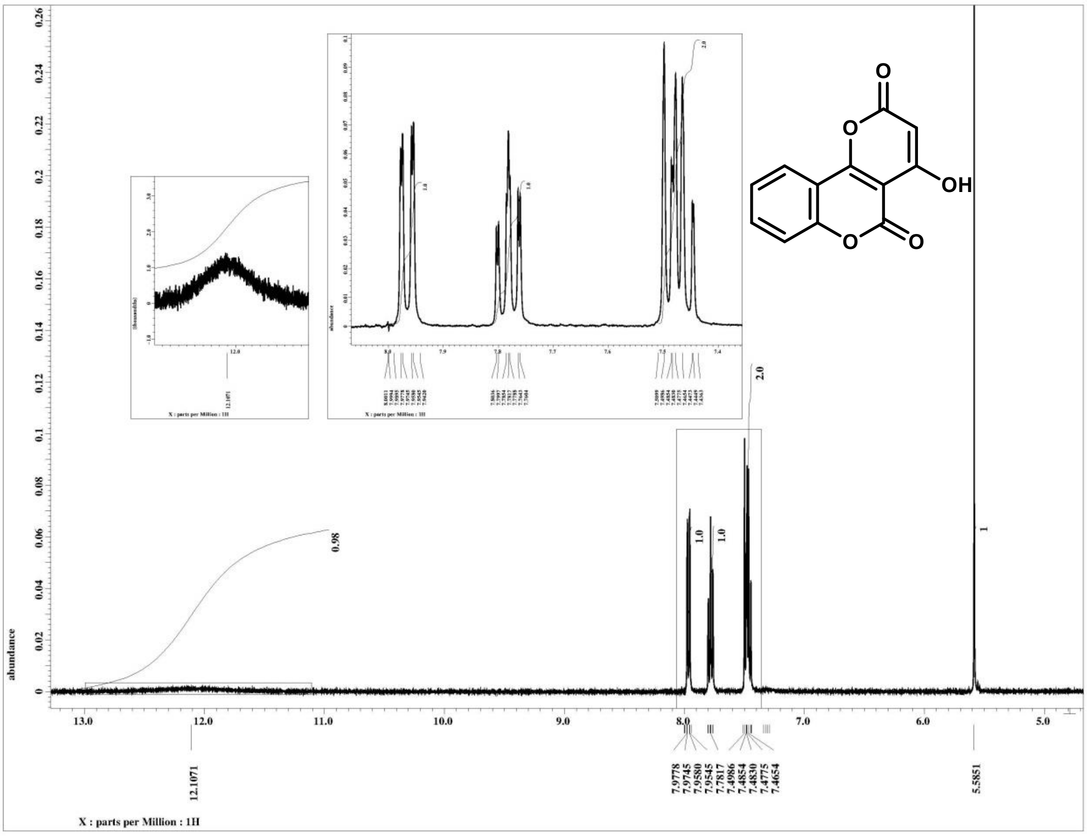

Figure S9. ^{1}H NMR spectrum of 4-hydroxy-2*H*,5*H*-pyrano[3,2-*c*][1]benzopyran-2,5-dione (**2d**) in DMSO-d_6

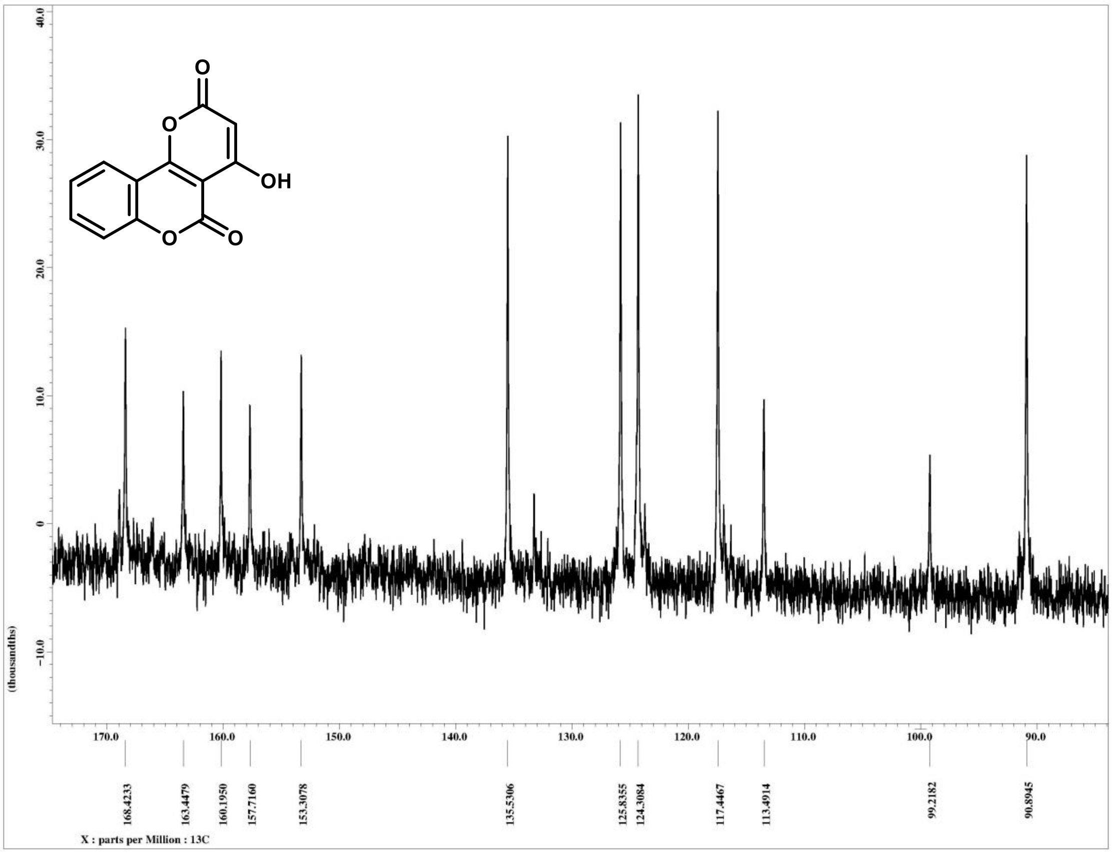

Figure S10. $^{13}C\{^{1}H\}$ NMR spectrum of 4-hydroxy-2*H*,5*H*-pyrano[3,2-*c*][1]benzopyran-2,5-dione (**2d**) in DMSO-d_6

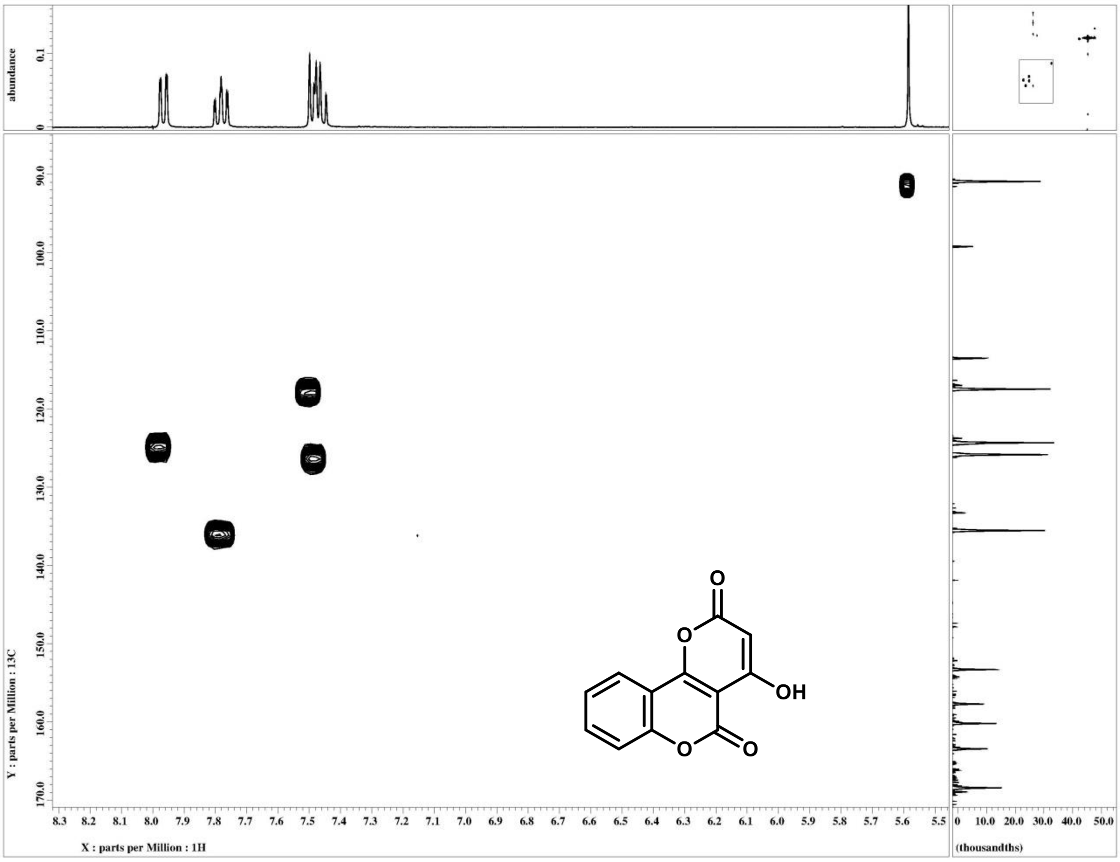

Figure S11. ^{1}H-^{13}C HMQC spectrum of 4-hydroxy-2*H*,5*H*-pyrano[3,2-*c*][1]benzopyran-2,5-dione (**2d**) in DMSO-d_6

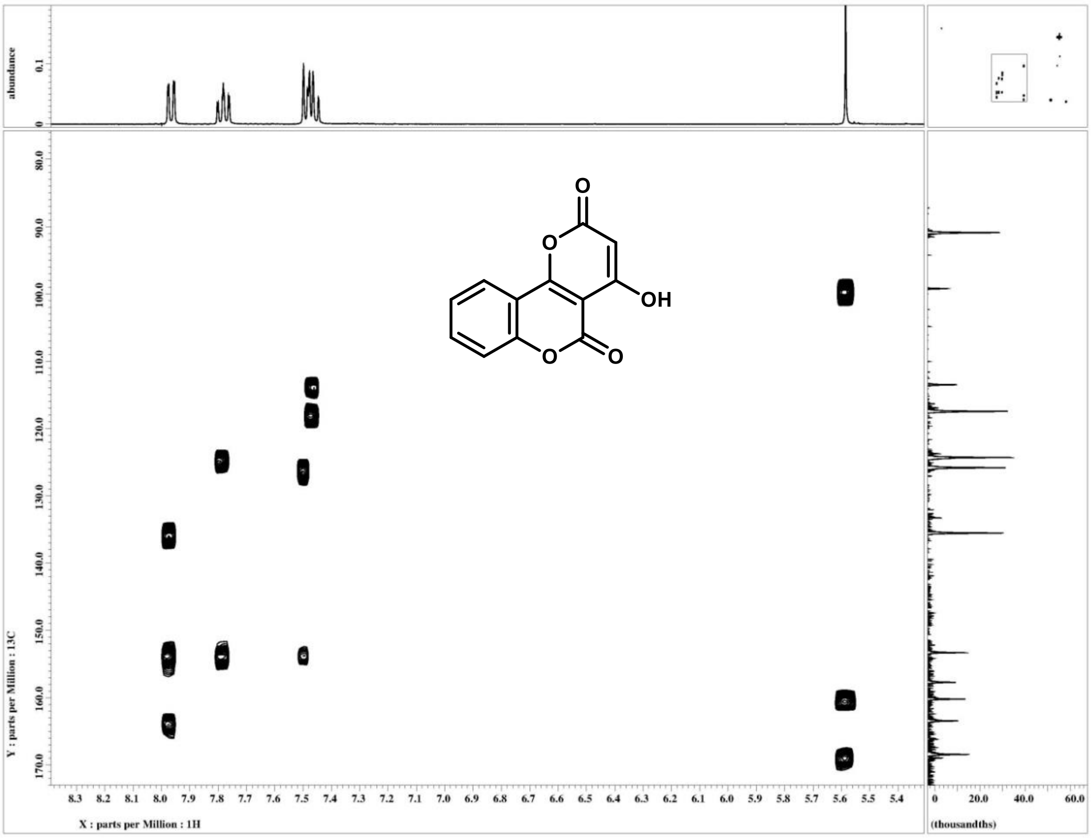

Figure S12. ^{1}H-^{13}C HMBC spectrum of 4-hydroxy-2*H*,5*H*-pyrano[3,2-*c*][1]benzopyran-2,5-dione (**2d**) in DMSO-d_6

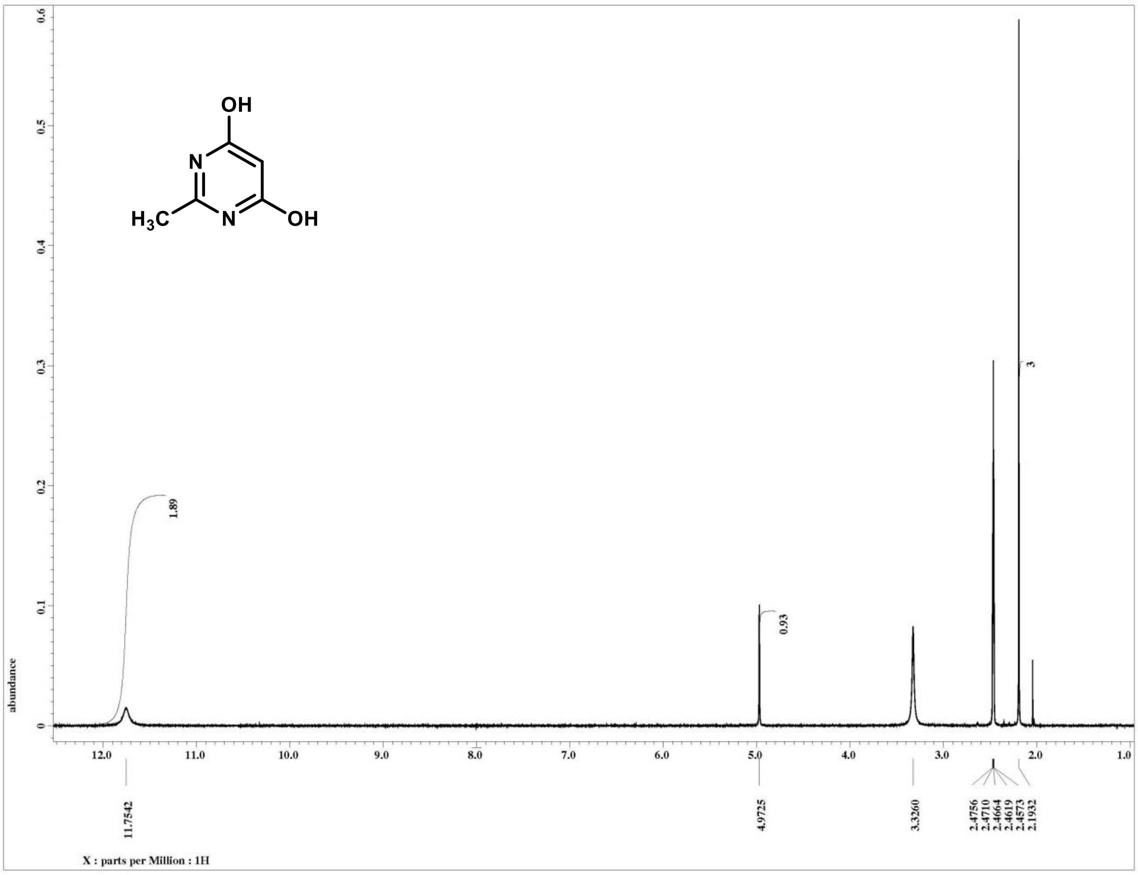

Figure S13. ^{1}H NMR spectrum of 2-methylpyrimidine-4,6-diol (**2e**) in DMSO-d_6

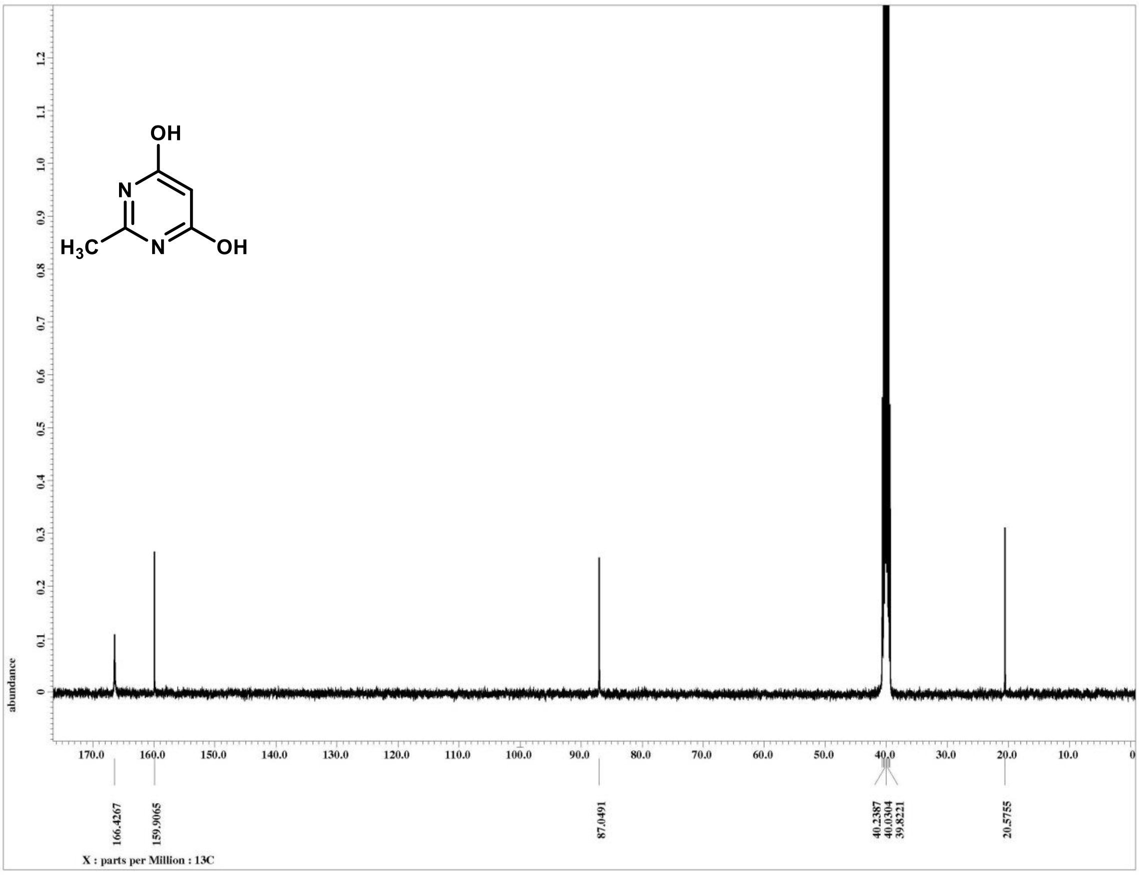

Figure S14. ^{13}C{^{1}H} NMR spectrum of 2-methylpyrimidine-4,6-diol (**2e**) in DMSO-d_6

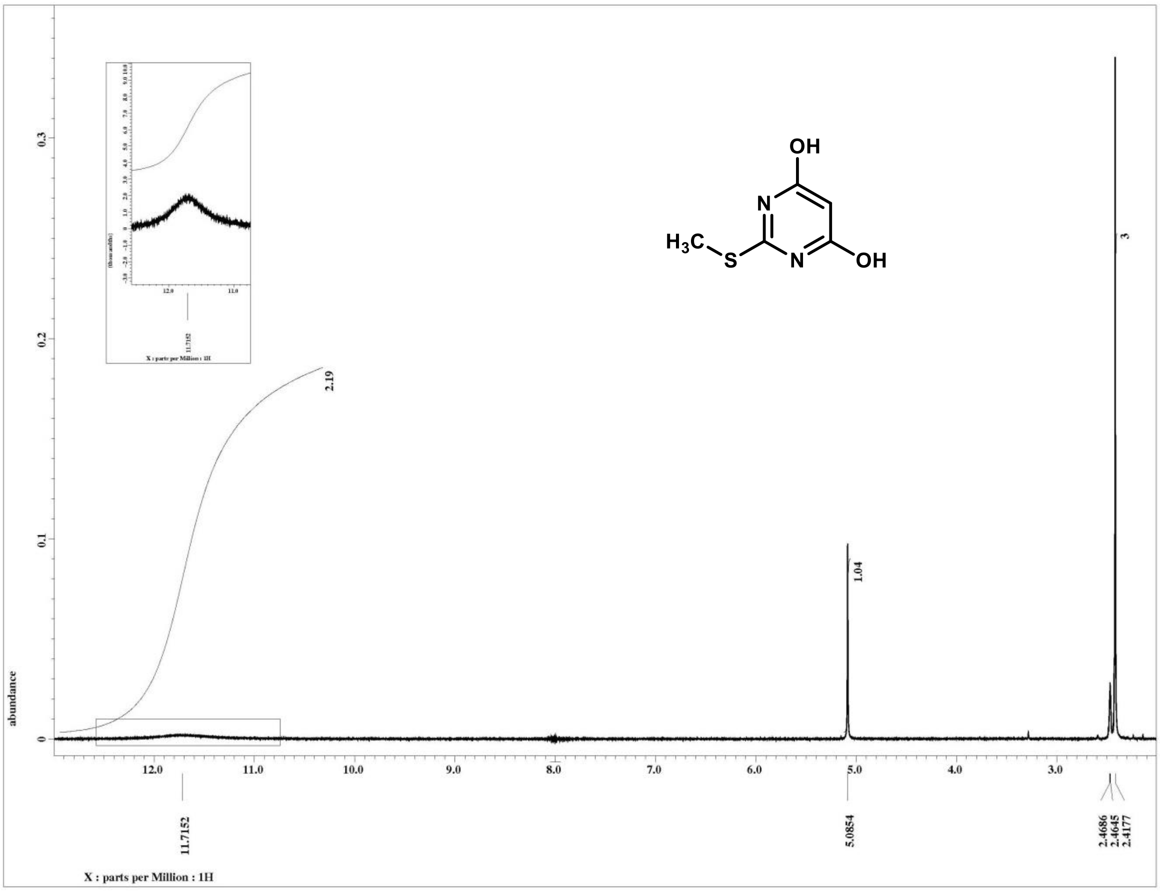

Figure S15. ^{1}H NMR spectrum of 2-(methylsulfanyl)pyrimidine-4,6-diol (**2f**) in DMSO-d_6

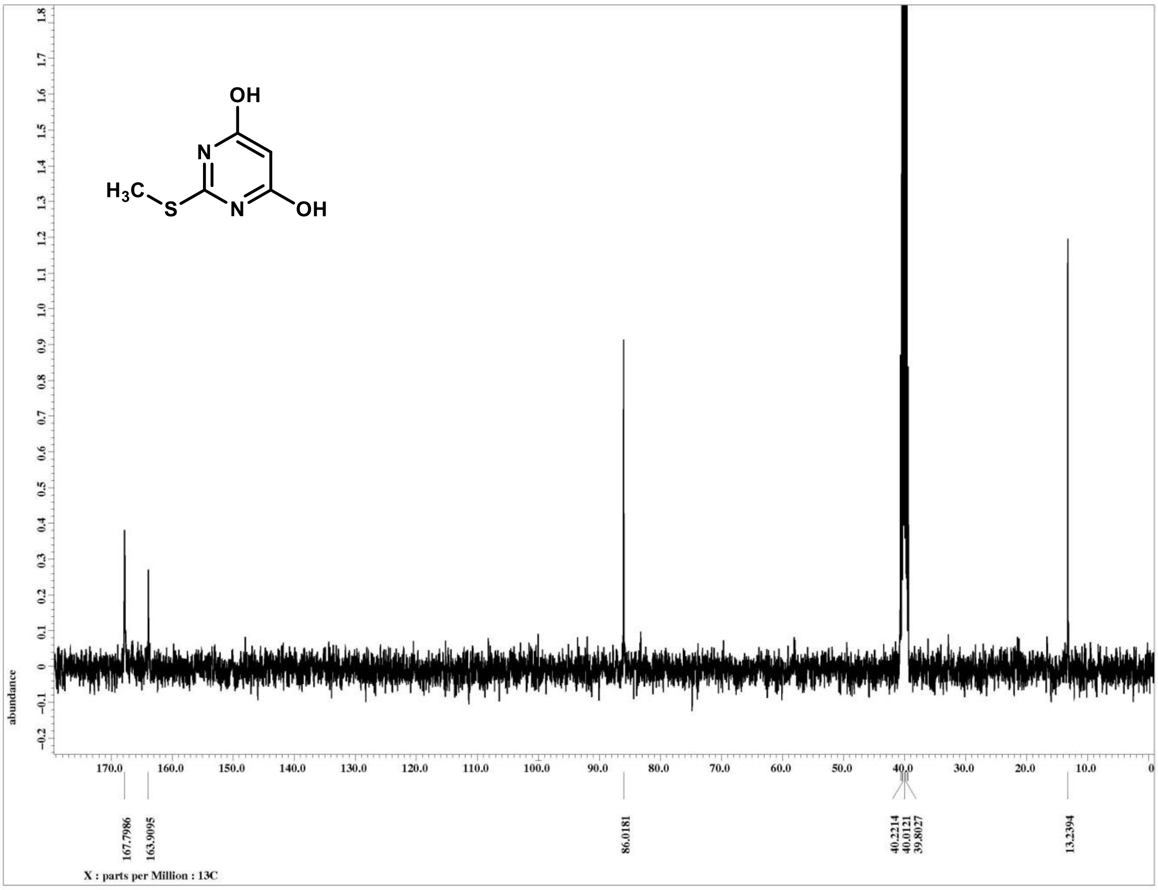

Figure S16. $^{13}C\{^{1}H\}$ NMR spectrum of 2-(methylsulfanyl)pyrimidine-4,6-diol (**2f**) in DMSO-d_6

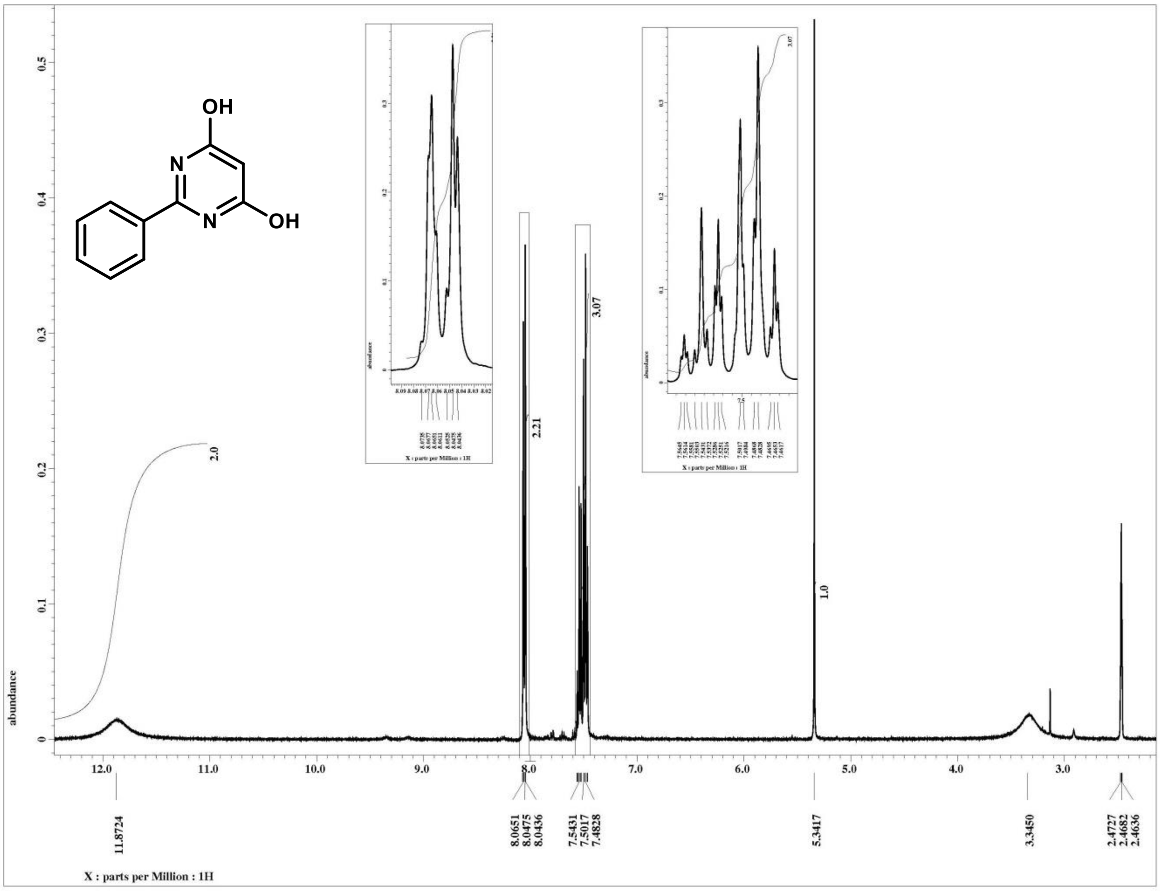

Figure S17. ^{1}H NMR spectrum of 2-phenylpyrimidine-4,6-diol (**2g**) in DMSO-d_6

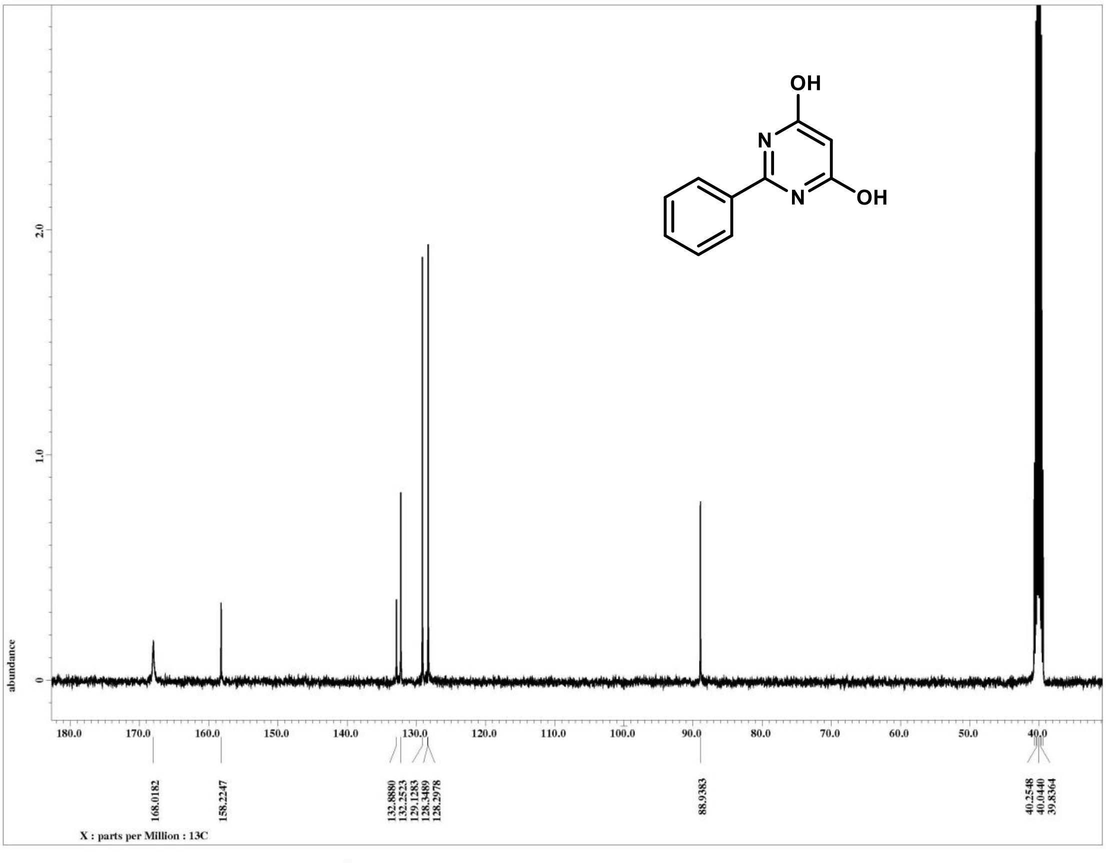

Figure S18. ^{13}C{^{1}H} NMR spectrum of 2-phenylpyrimidine-4,6-diol (**2g**) in DMSO-d_6

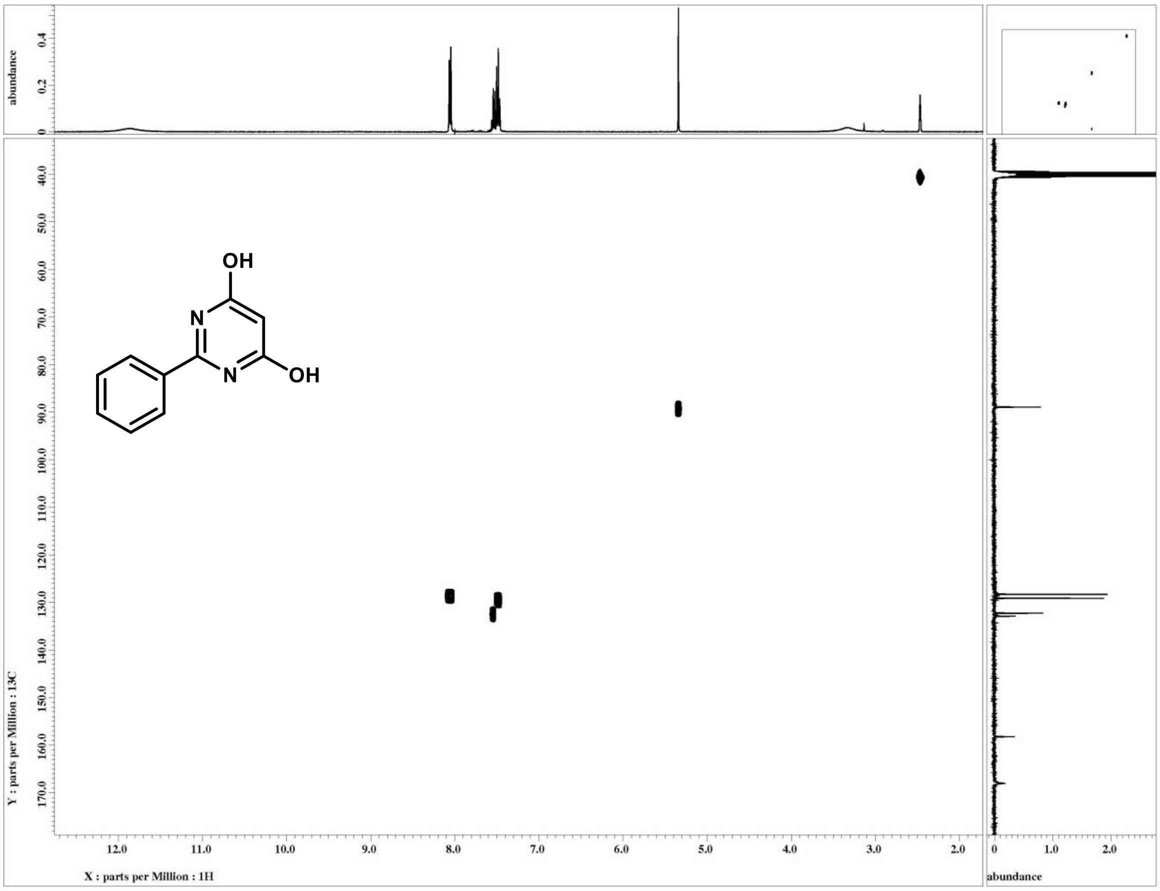

Figure S19. ^{1}H-^{13}C HMQC spectrum of 2-phenylpyrimidine-4,6-diol (**2g**) in DMSO-d_6

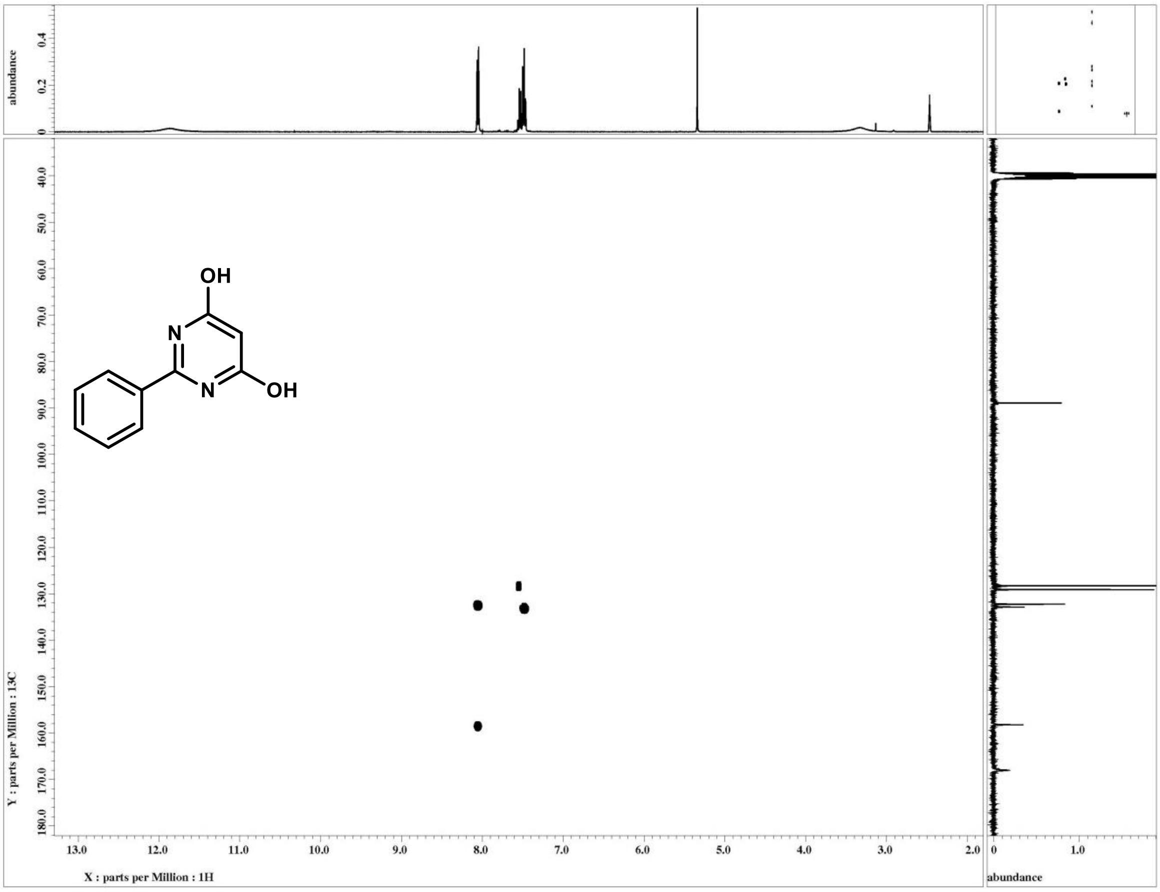

Figure S20. ^{1}H-^{13}C HMBC spectrum of 2-phenylpyrimidine-4,6-diol (**2g**) in DMSO-d_6

Spectra of condensed furancarboxylates

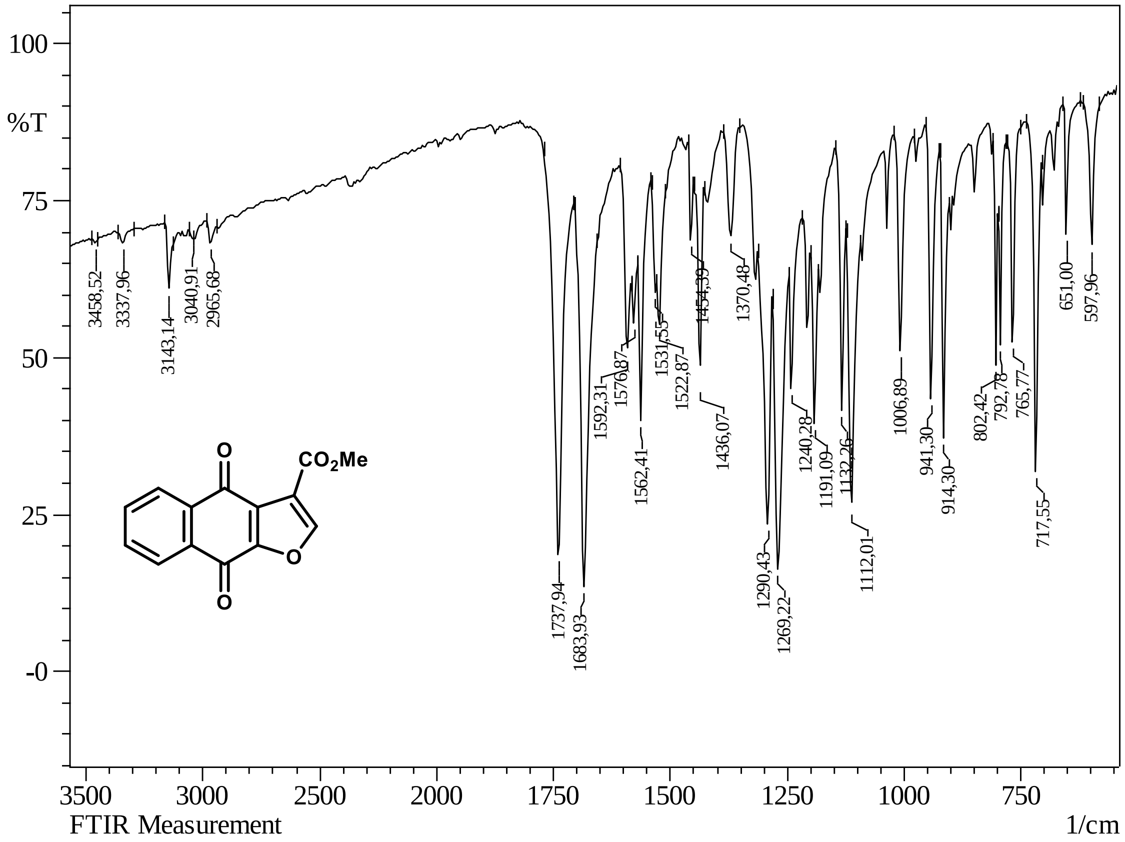

Figure S21. IR spectrum of methyl 4,9-dioxo-4,9-dihydronaphtho[2,3-*b*]furan-3-carboxylate (**3a**) in KBr

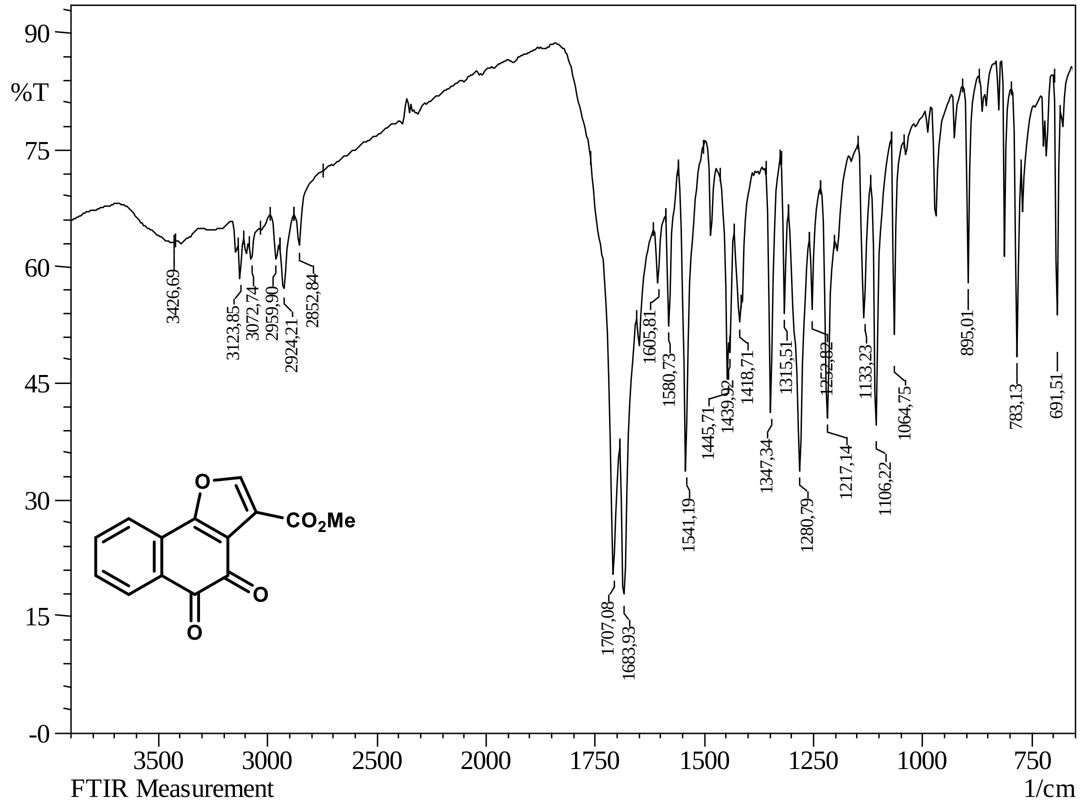

Figure S22. IR spectrum of methyl 4,5-dioxo-4,5-dihydronaphtho[1,2-*b*]furan-3-carboxylate (**4a**) in KBr

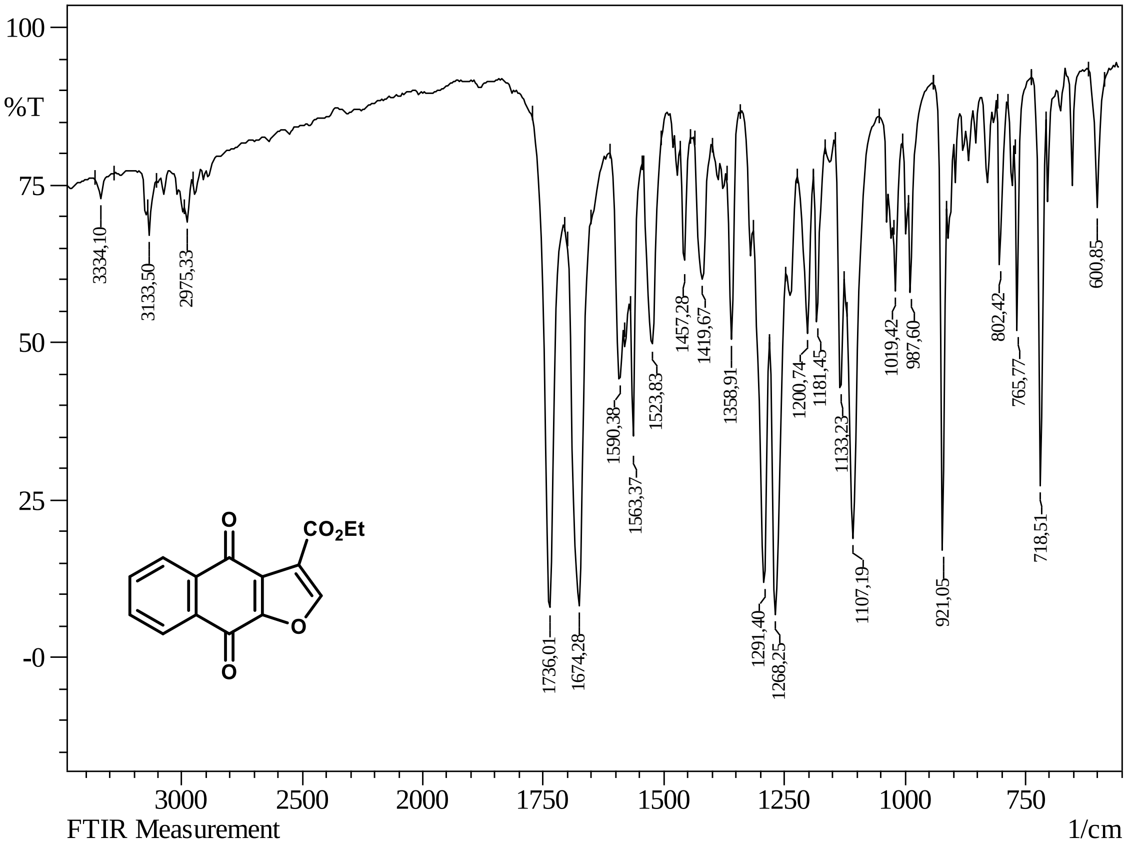

Figure S23. IR spectrum of ethyl 4,9-dioxo-4,9-dihydronaphtho[2,3-*b*]furan-3-carboxylate (**3b**) in KBr

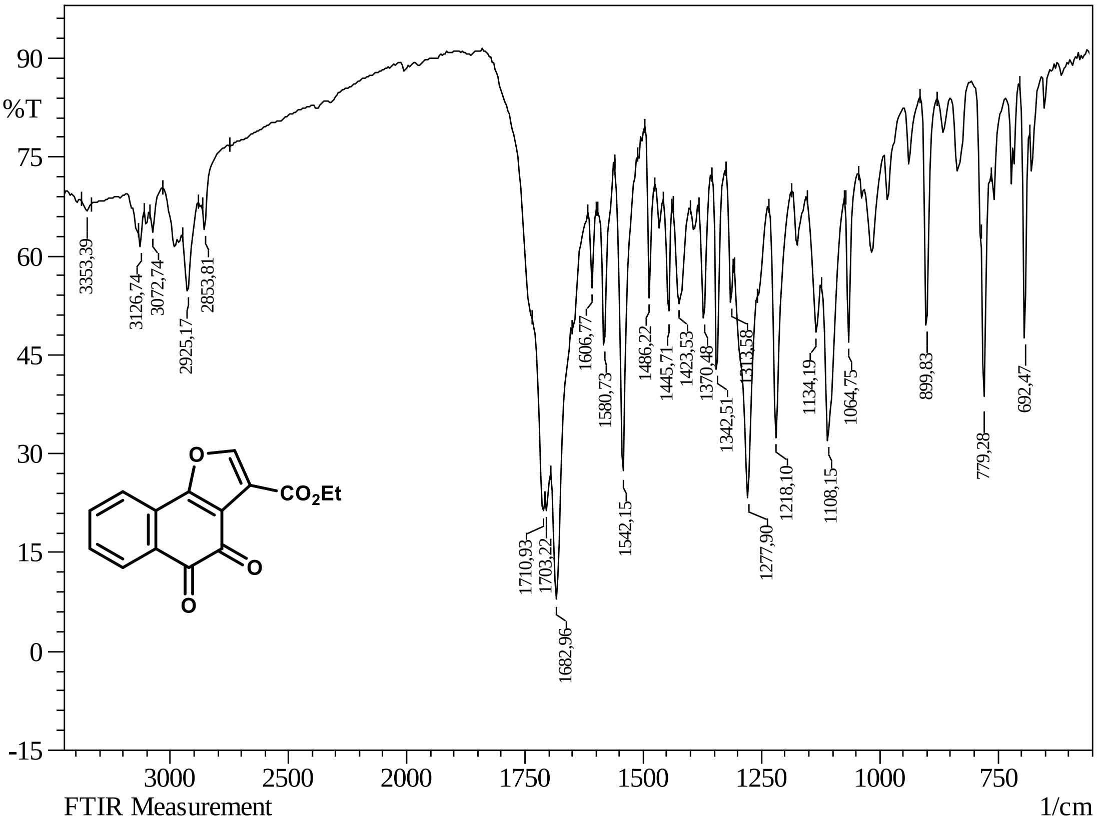

Figure S24. IR spectrum of ethyl 4,5-dioxo-4,5-dihydronaphtho[1,2-*b*]furan-3-carboxylate (**4b**) in KBr

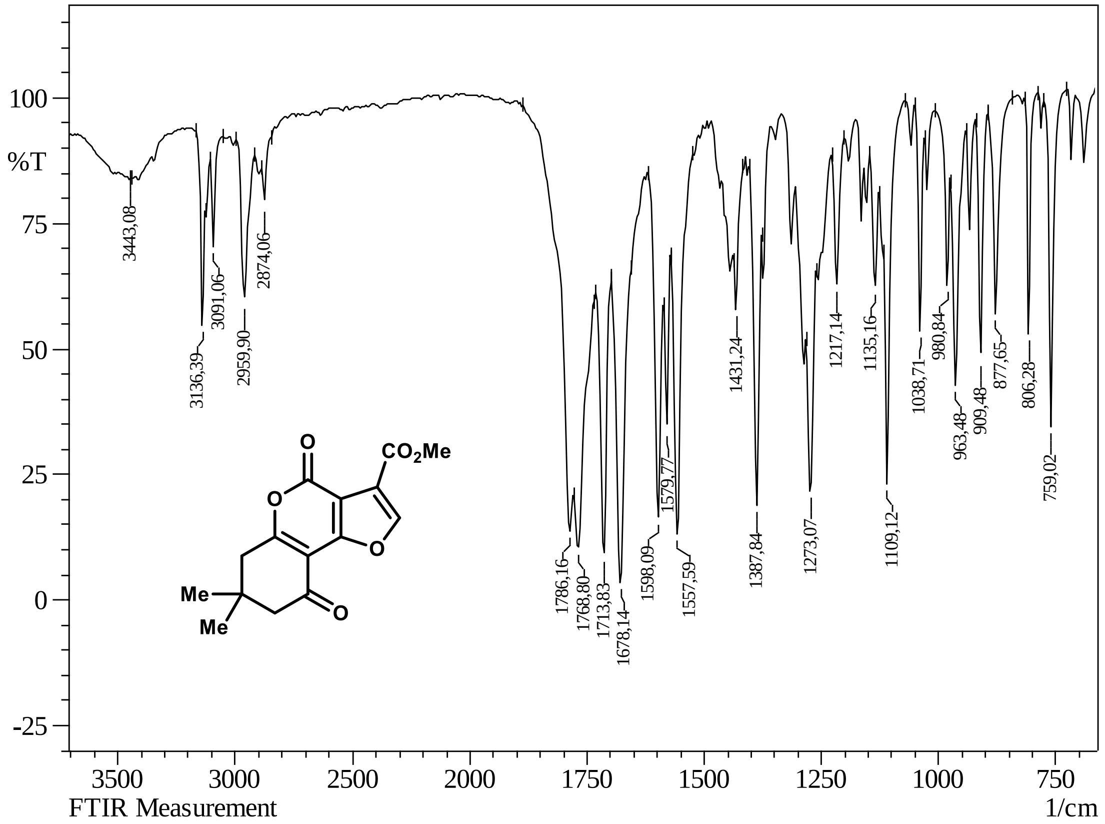

Figure S25. IR spectrum of methyl 7,7-dimethyl-4,9-dioxo-6,7,8,9-tetrahydro-4*H*-furo[3,2-*c*][1]benzopyran-3-carboxylate (**5a**) in KBr

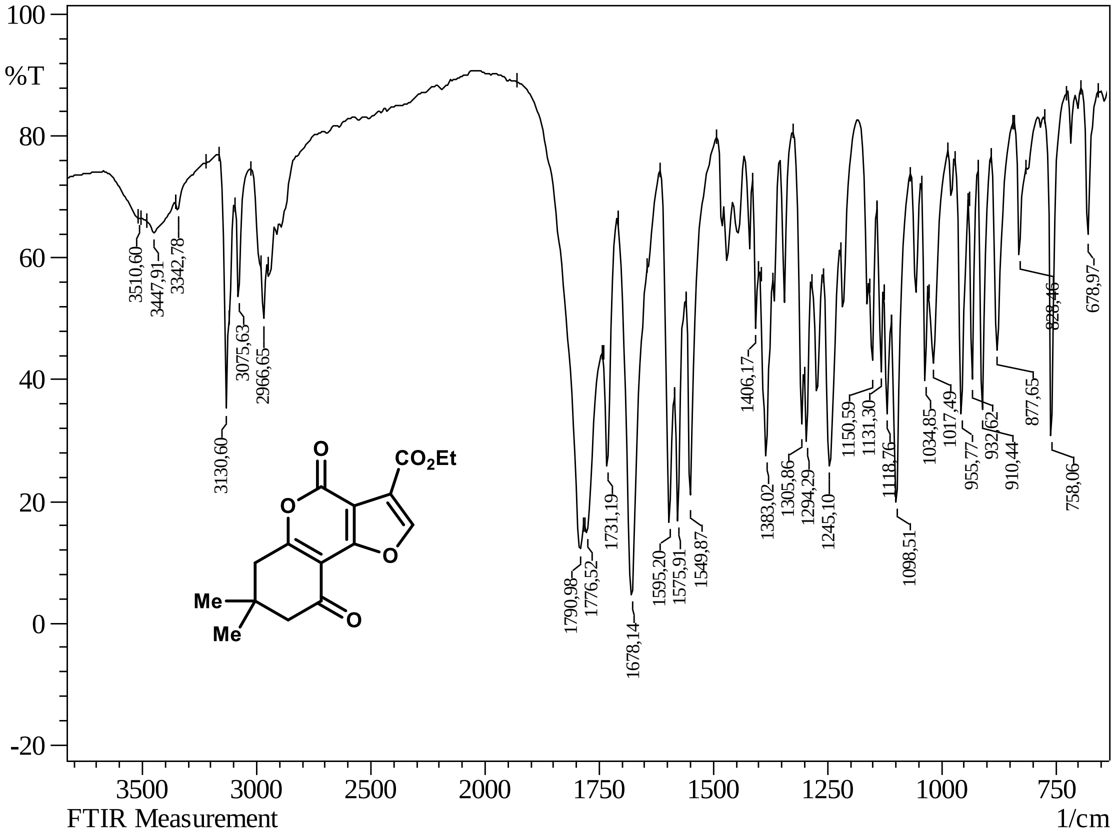

Figure S26. IR spectrum of ethyl 7,7-dimethyl-4,9-dioxo-6,7,8,9-tetrahydro-4*H*-furo[3,2-*c*][1]benzopyran-3-carboxylate (**5b**) in KBr

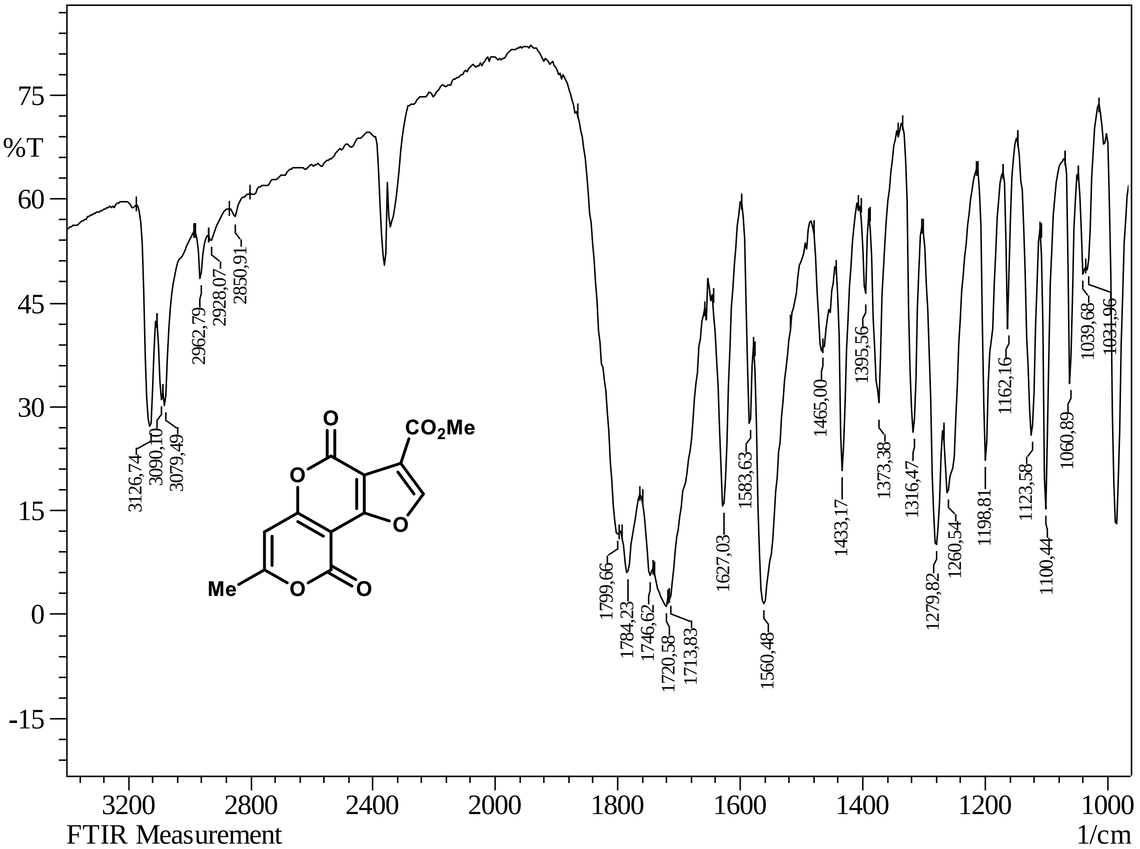

Figure S27. IR spectrum of methyl 7-methyl-4,9-dioxo-4*H*,9*H*-furo[2,3-*d*]pyrano[4,3-*b*]pyran-3-carboxylate (**6a**) in KBr

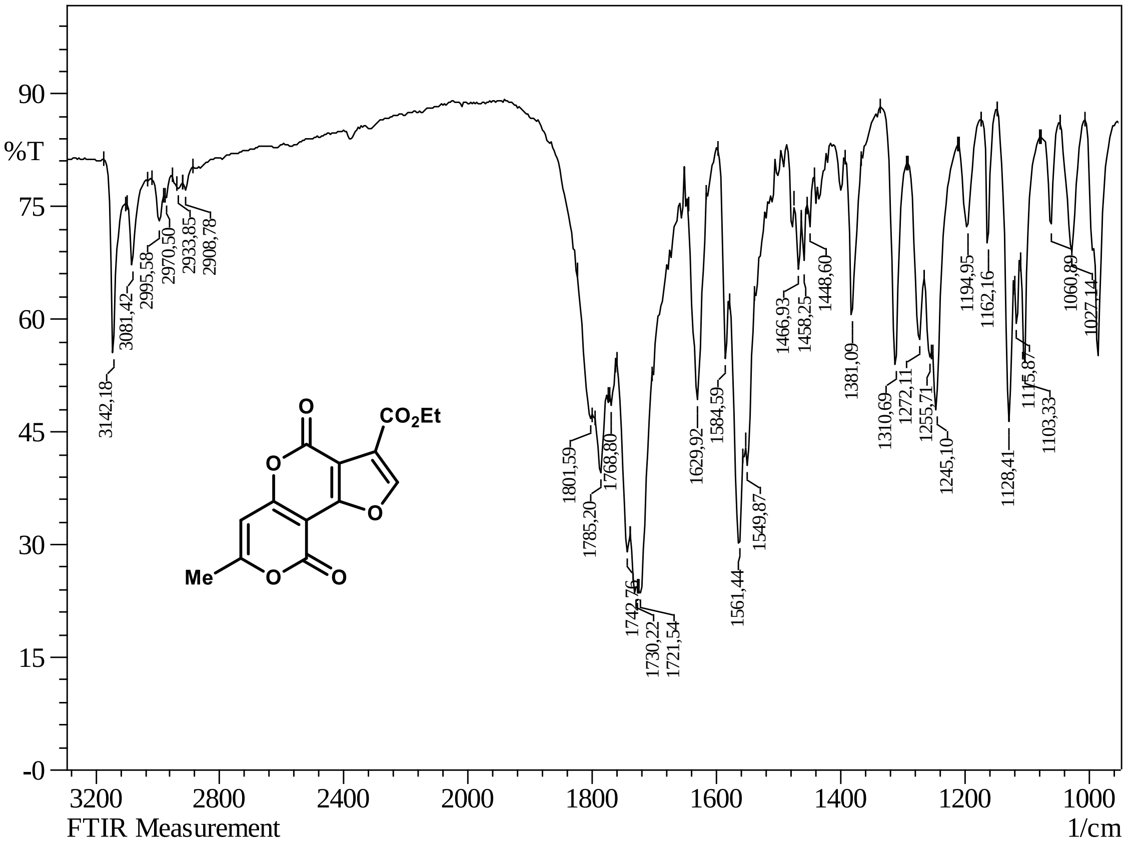

Figure S28. IR spectrum of ethyl 7-methyl-4,9-dioxo-4*H*,9*H*-furo[2,3-*d*]pyrano[4,3-*b*]pyran-3-carboxylate (**6b**) in KBr

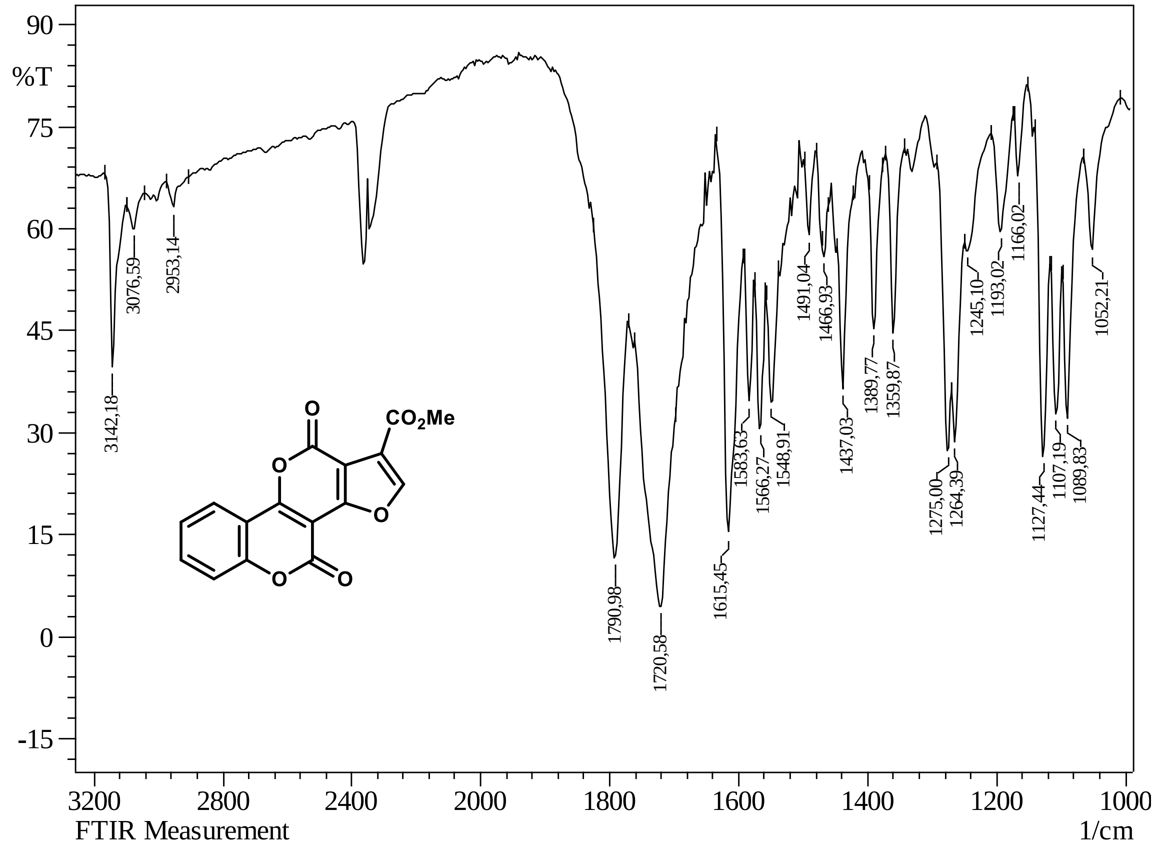

Figure S29. IR spectrum of methyl 4,11-dioxo-4*H*,11*H*-furo[2',3':4,5]pyrano[3,2-*c*]chromene-1-carboxylate (**6c**) in KBr

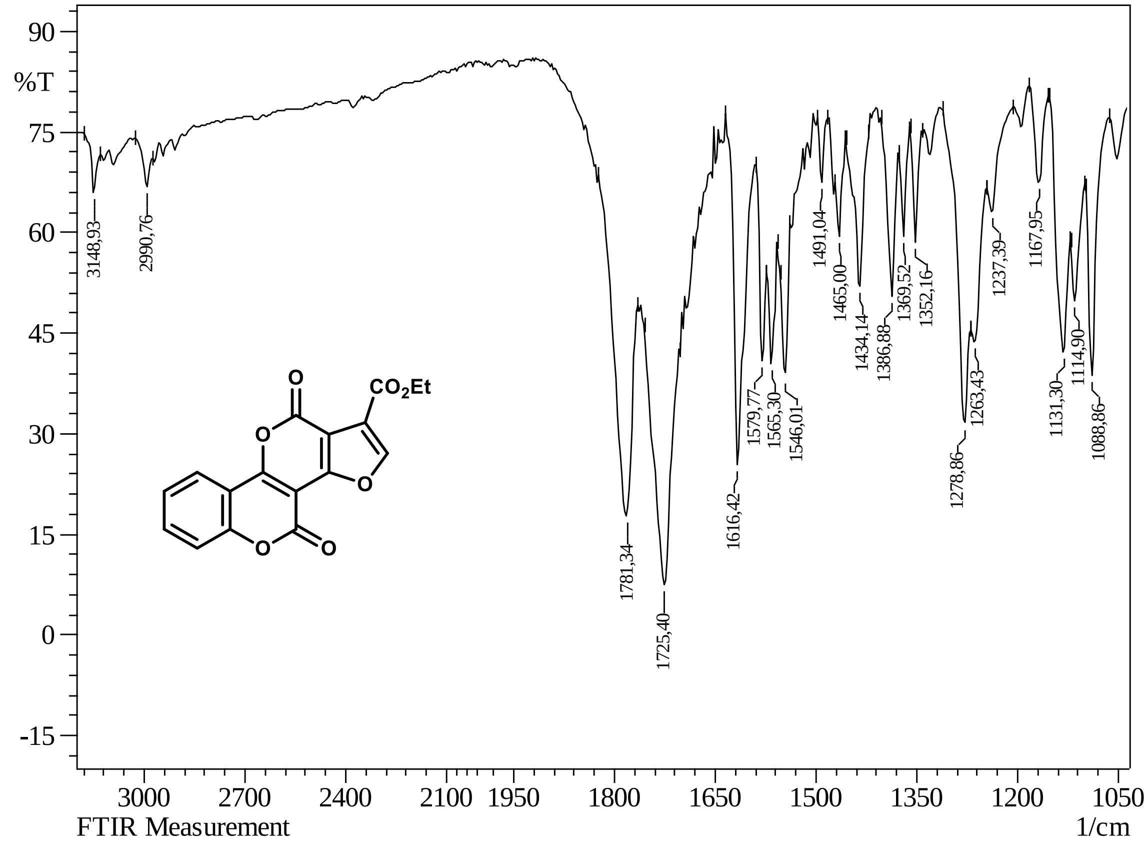

Figure S30. IR spectrum of ethyl 4,11-dioxo-4*H*,11*H*-furo[2',3':4,5]pyrano[3,2-*c*]chromene-1-carboxylate (**6d**) in KBr

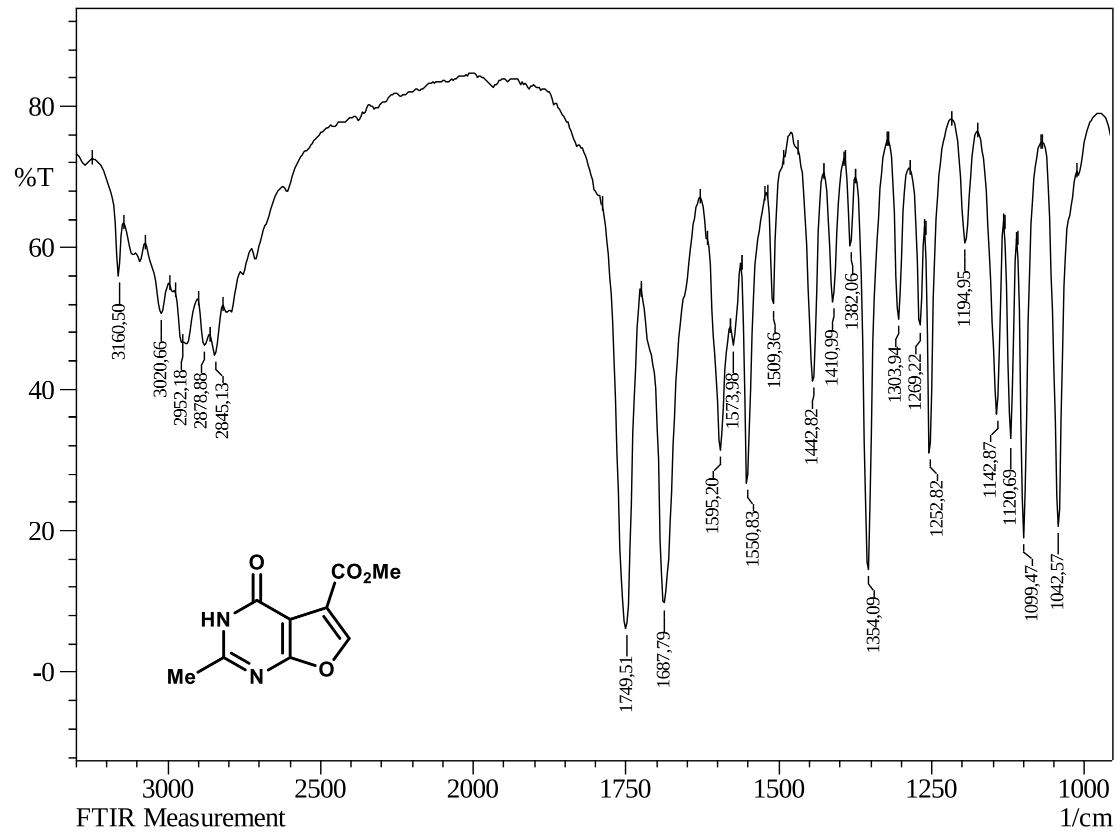

Figure S31. IR spectrum of methyl 2-methyl-4-oxo-3,4-dihydrofuro[2,3-*d*]pyrimidine-5-carboxylate (**7a**) in KBr

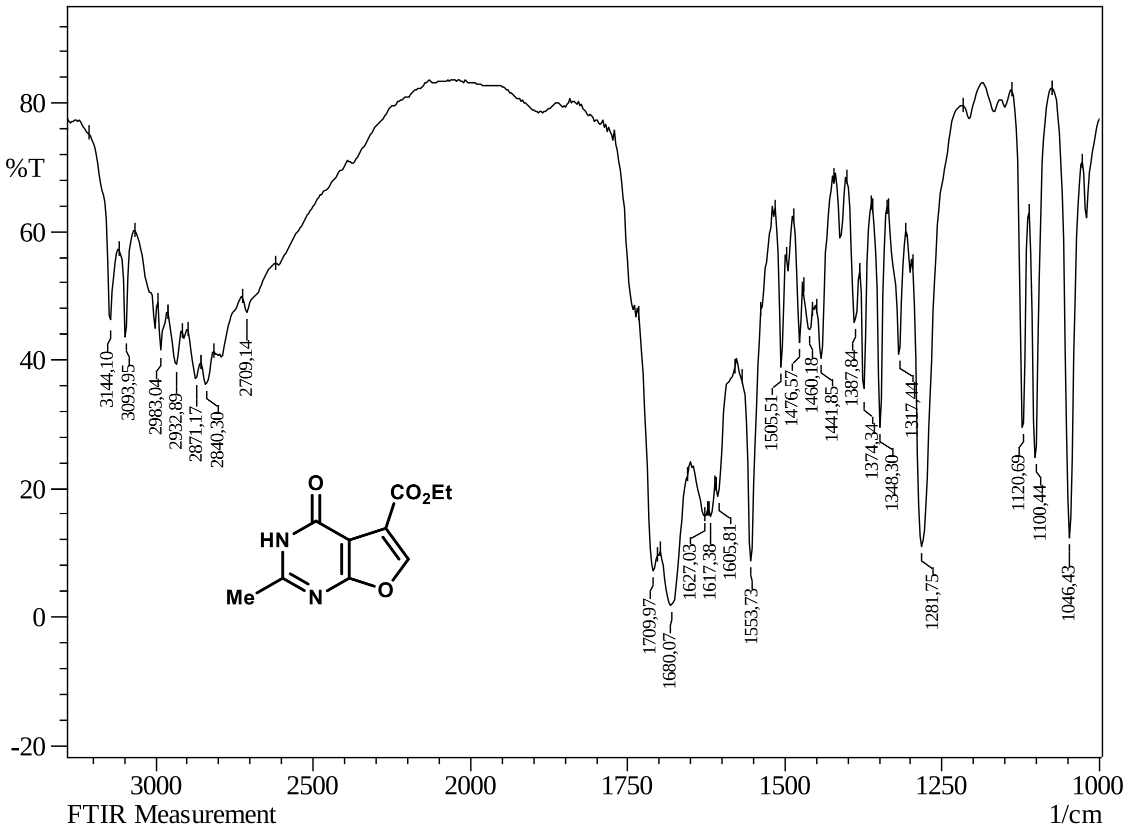

Figure S32. IR spectrum of ethyl 2-methyl-4-oxo-3,4-dihydrofuro[2,3-*d*]pyrimidine-5-carboxylate (**7b)** in KBr

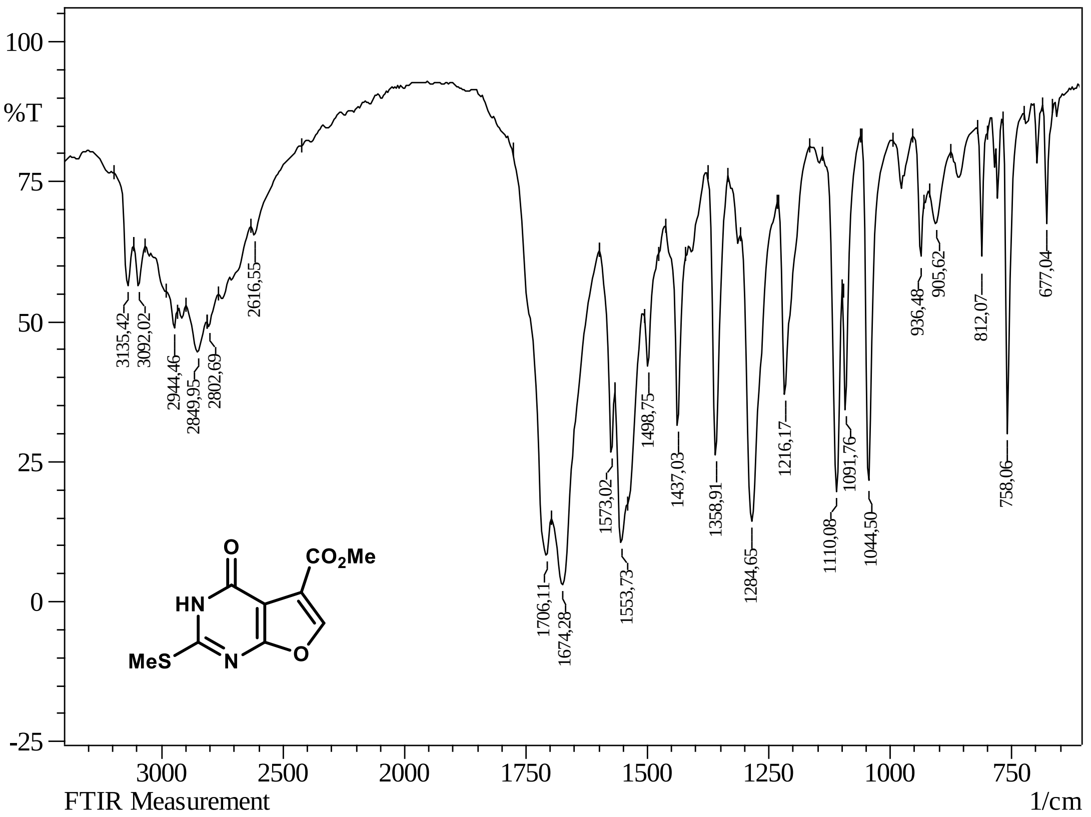

Figure S33. IR spectrum of methyl 2-(methylsulfanyl)-4-oxo-3,4-dihydrofuro[2,3-*d*]pyrimidine-5-carboxylate (**7c**) in KBr

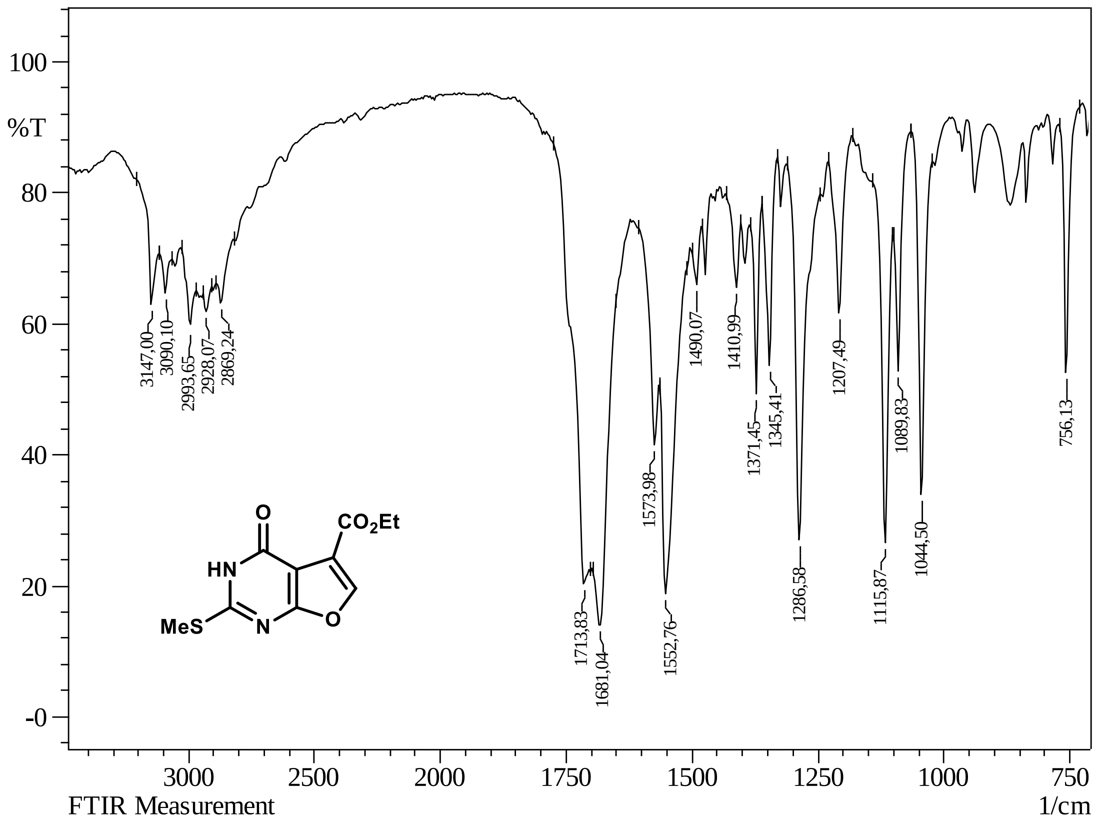

Figure S34. IR spectrum of ethyl 2-(methylsulfanyl)-4-oxo-3,4-dihydrofuro[2,3-*d*]pyrimidine-5-carboxylate (**7d**) in KBr

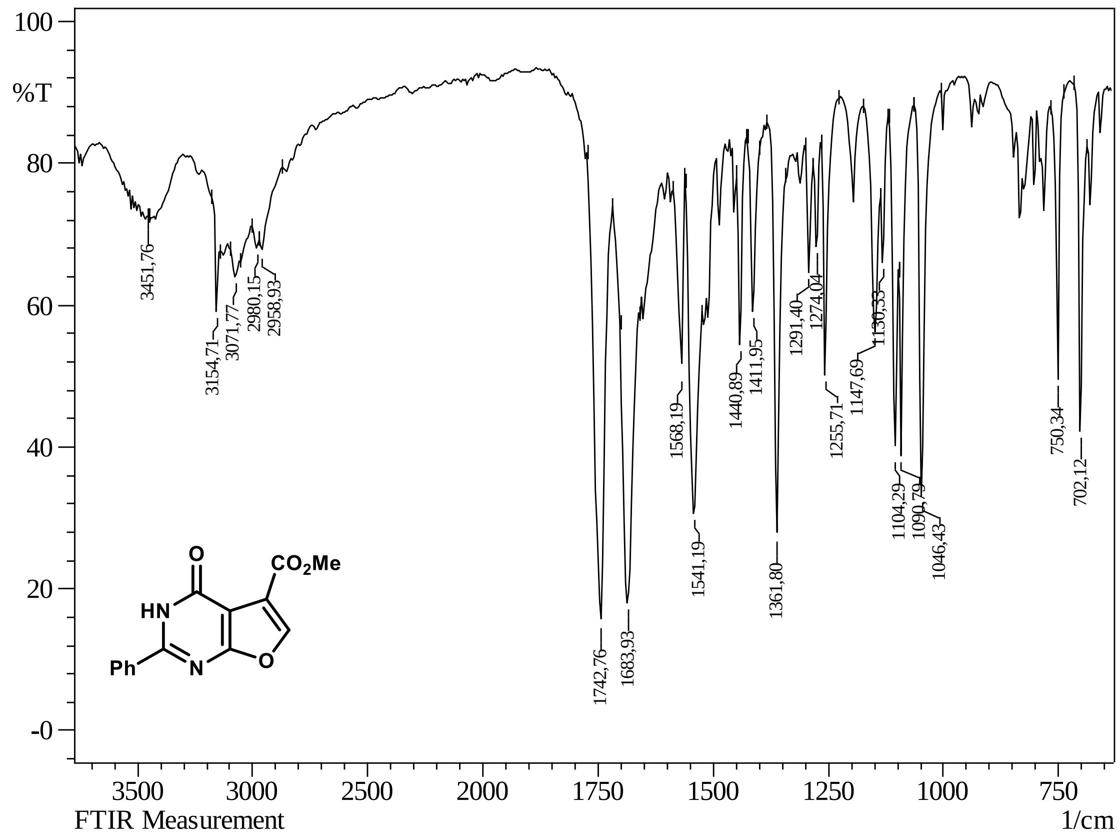

Figure S35. IR spectrum of methyl 4-oxo-2-phenyl-3,4-dihydrofuro[2,3-*d*]pyrimidine-5-carboxylate (**7e**) in KBr

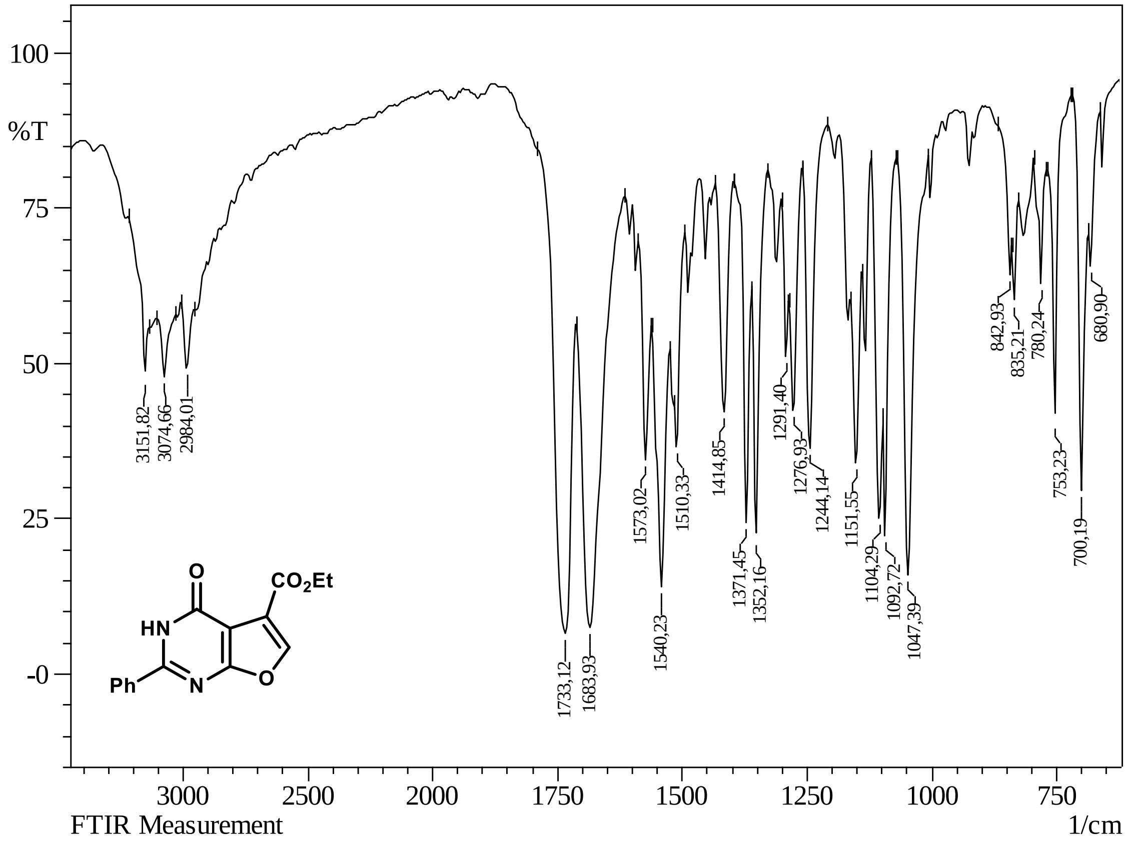

Figure S36. IR spectrum of ethyl 4-oxo-2-phenyl-3,4-dihydrofuro[2,3-*d*]pyrimidine-5-carboxylate (**7f**) in KBr

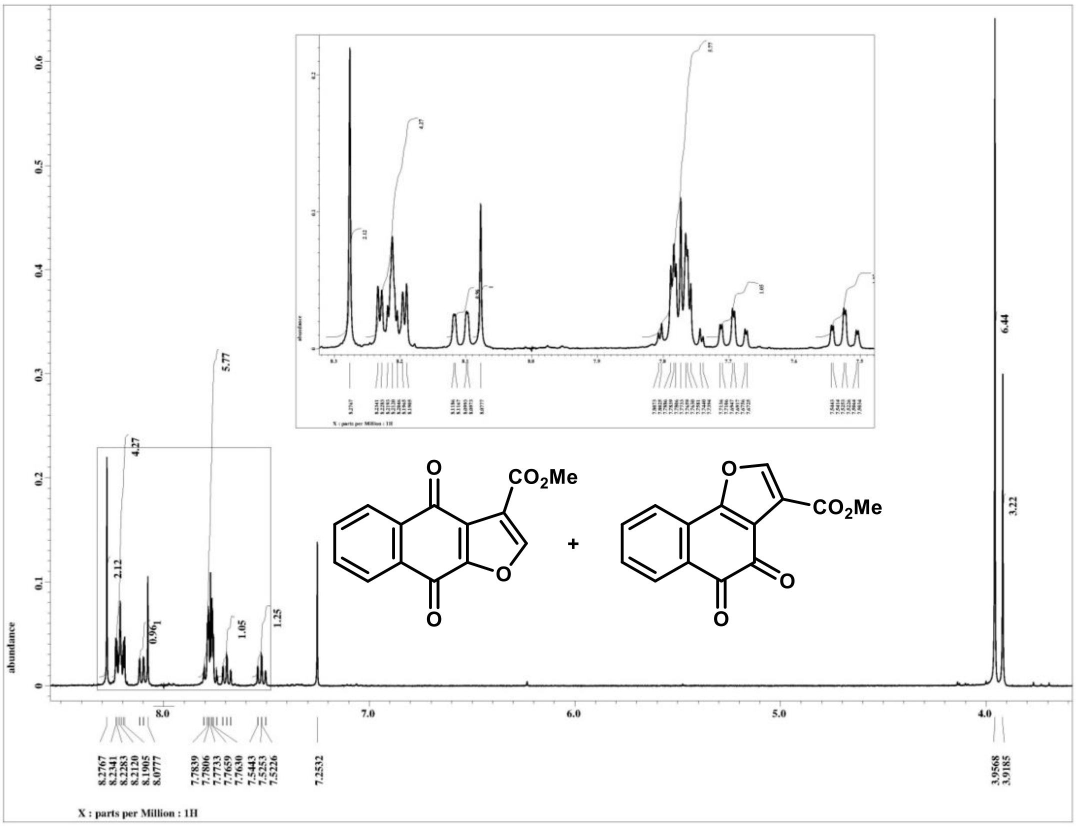

Figure S37. ^{1}H NMR spectrum of the mixture methyl 4,9-dioxo-4,9-dihydronaphtho[2,3-*b*]furan-3-carboxylate (**3a**) and methyl 4,5-dioxo-4,5-dihydronaphtho[1,2-*b*]furan-3-carboxylate (**4a**) in $CDCl_3$

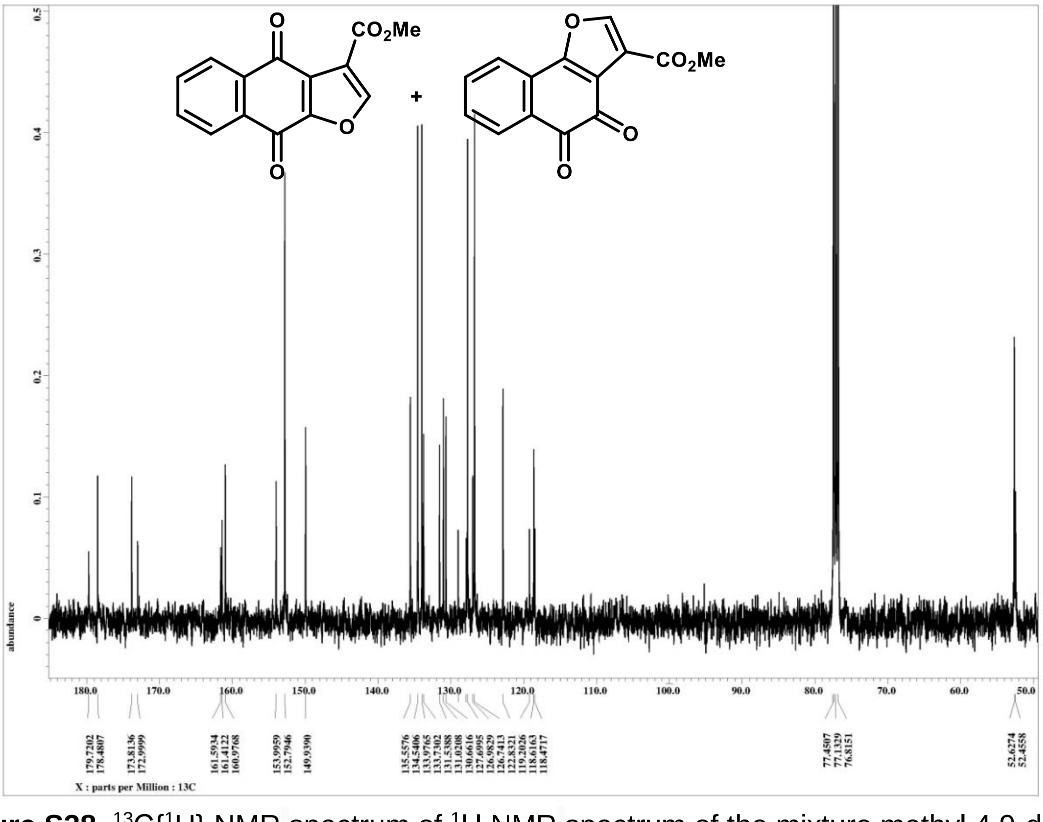

Figure S38. $^{13}C\{^{1}H\}$ NMR spectrum of ^{1}H NMR spectrum of the mixture methyl 4,9-dioxo-4,9-dihydronaphtho[2,3-*b*]furan-3-carboxylate (**3a**) and methyl 4,5-dioxo-4,5-dihydronaphtho[1,2-*b*]furan-3-carboxylate (**4a**) in $CDCl_3$

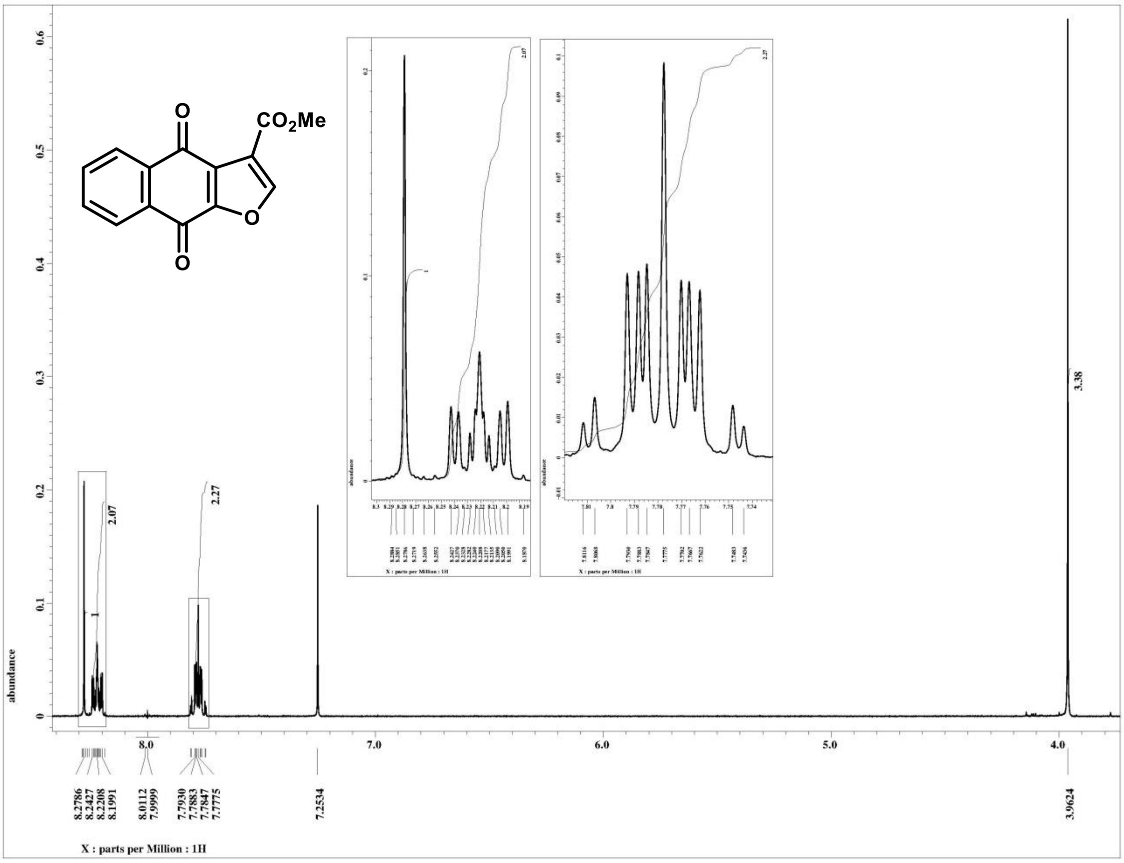

Figure S39. ^{1}H NMR spectrum of methyl 4,9-dioxo-4,9-dihydronaphtho[2,3-*b*]furan-3-carboxylate (**3a**) in $CDCl_3$

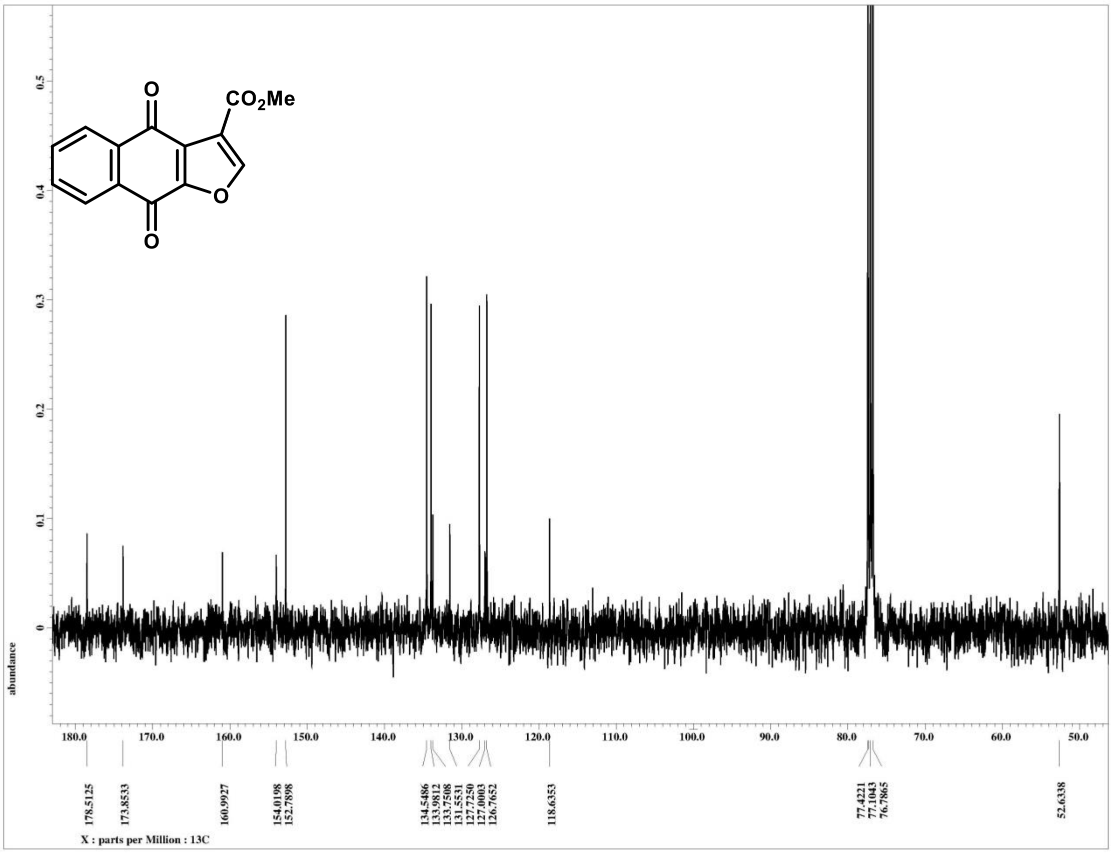

Figure S40. $^{13}C\{^{1}H\}$ NMR spectrum of methyl 4,9-dioxo-4,9-dihydronaphtho[2,3-*b*]furan-3-carboxylate (**3a**) in $CDCl_3$

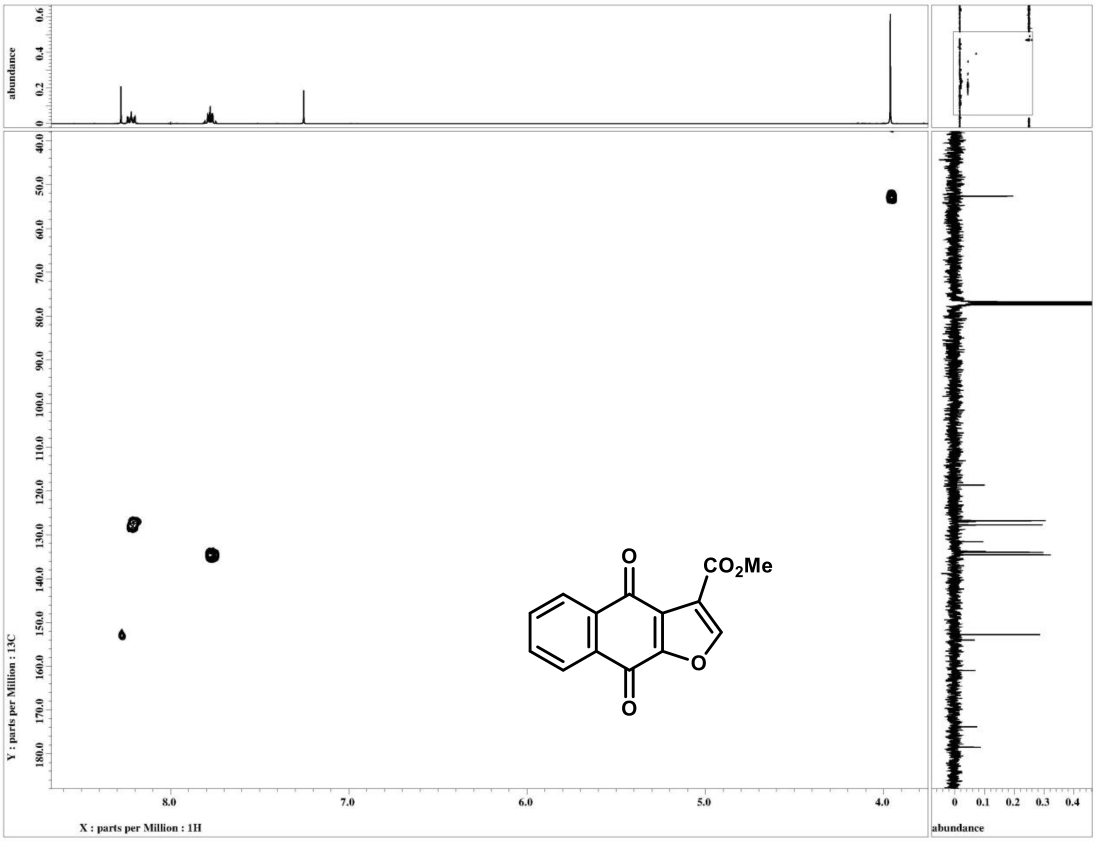

Figure S41. ^{1}H-^{13}C HMQC spectrum of methyl 4,9-dioxo-4,9-dihydronaphtho[2,3-*b*]furan-3-carboxylate (**3a**) in $CDCl_3$

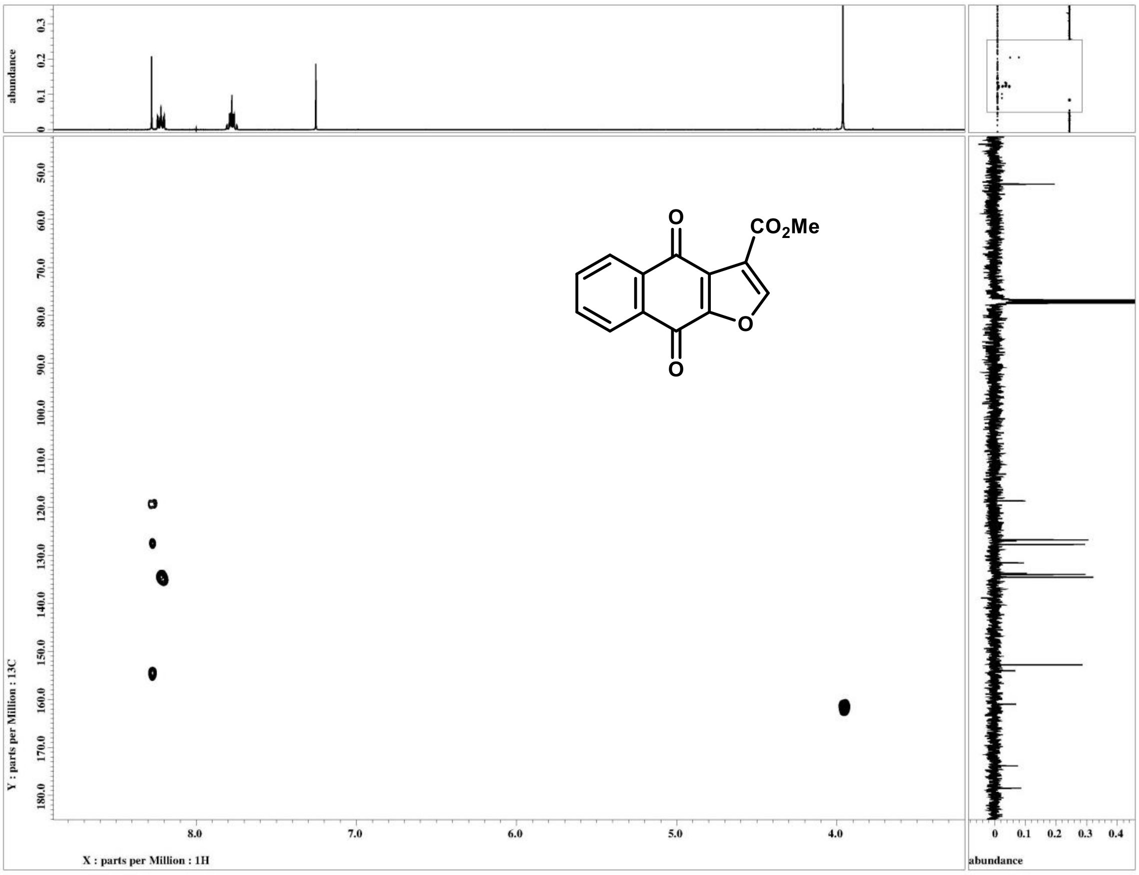

Figure S42. ^{1}H-^{13}C HMBC spectrum of methyl 4,9-dioxo-4,9-dihydronaphtho[2,3-*b*]furan-3-carboxylate (**3a**) in $CDCl_3$

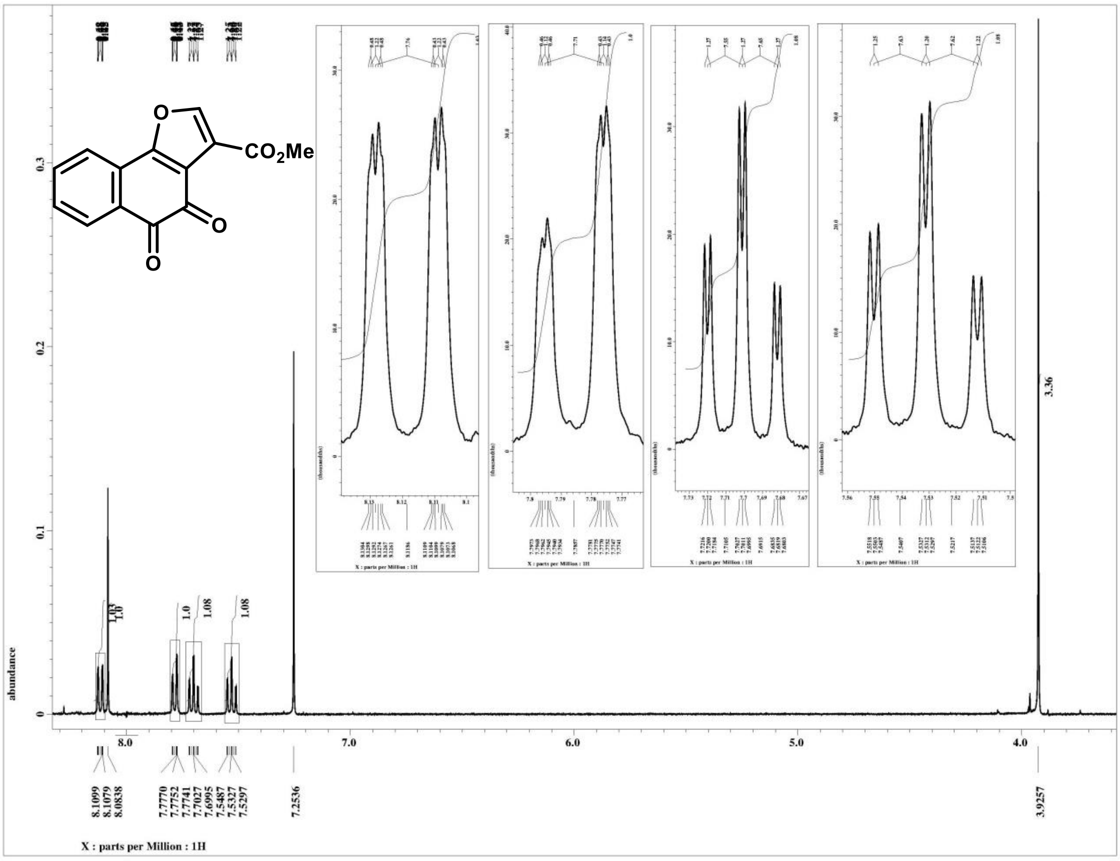

Figure S43. ^{1}H NMR spectrum of methyl 4,5-dioxo-4,5-dihydronaphtho[1,2-*b*]furan-3-carboxylate (**4a**) in $CDCl_3$

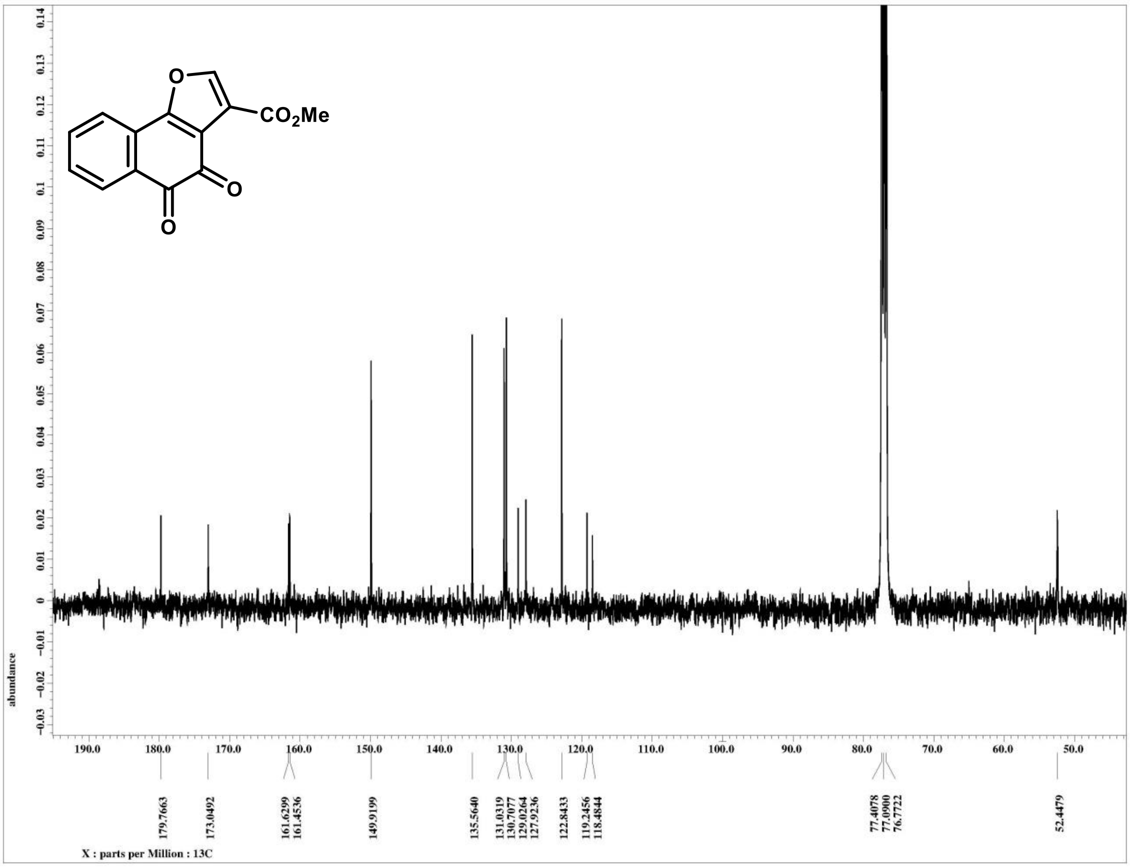

Figure S44. $^{13}C\{^{1}H\}$ NMR spectrum of methyl 4,5-dioxo-4,5-dihydronaphtho[1,2-*b*]furan-3-carboxylate (**4a**) in $CDCl_3$

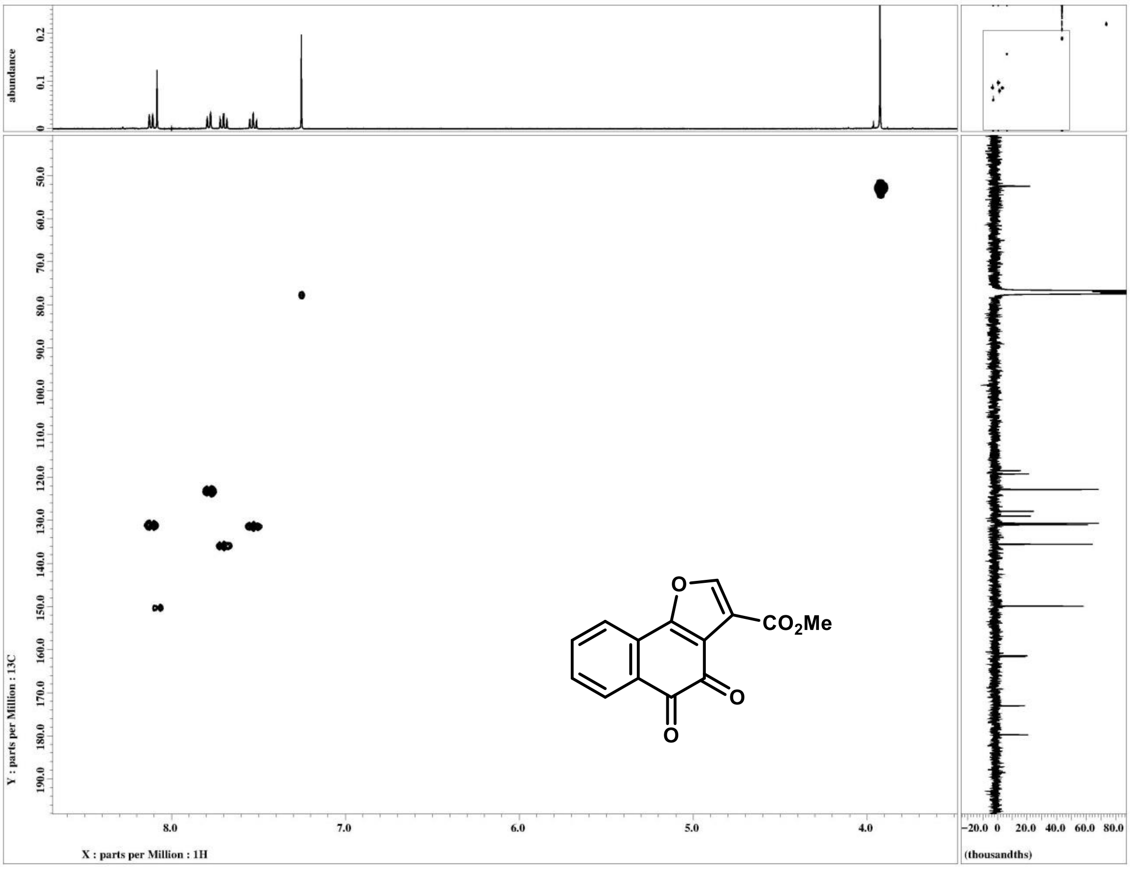

Figure S45. ^{1}H-^{13}C HMQC spectrum of methyl 4,5-dioxo-4,5-dihydronaphtho[1,2-*b*]furan-3-carboxylate (**4a**) in $CDCl_3$

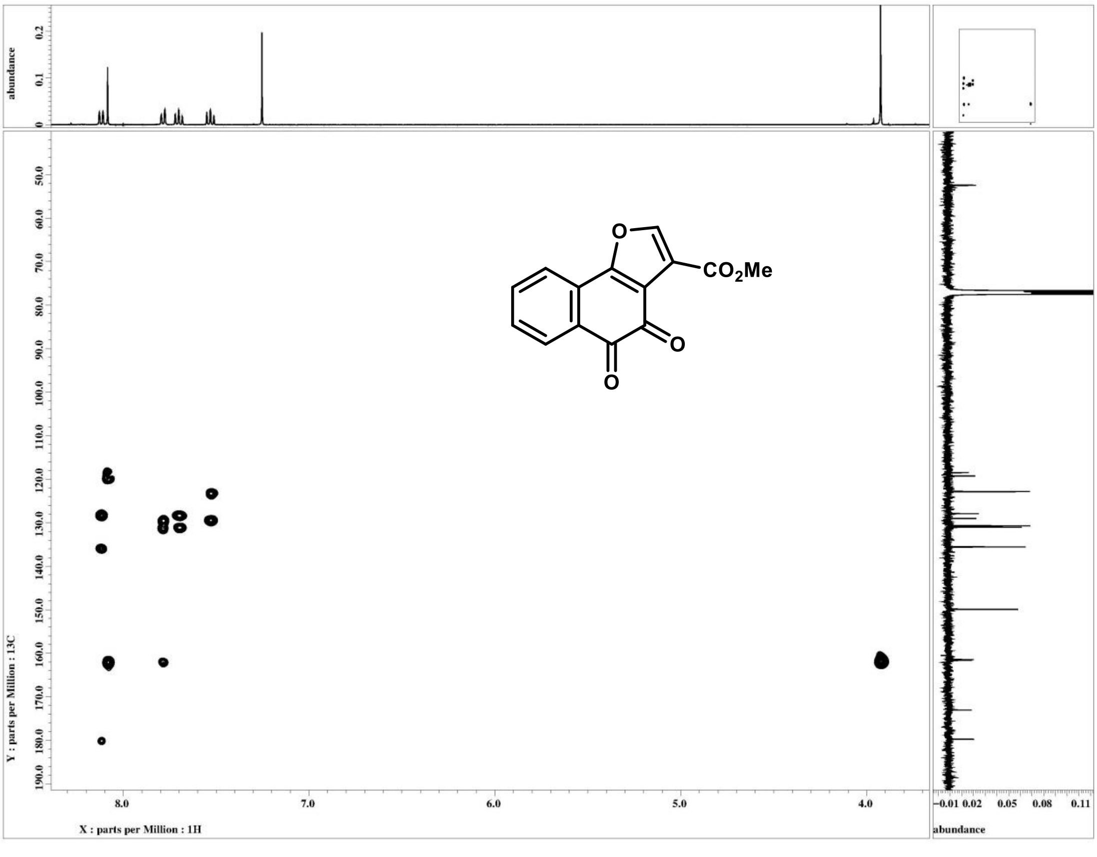

Figure S46. ^{1}H-^{13}C HMBC spectrum of methyl 4,5-dioxo-4,5-dihydronaphtho[1,2-*b*]furan-3-carboxylate (**4a**) in $CDCl_3$

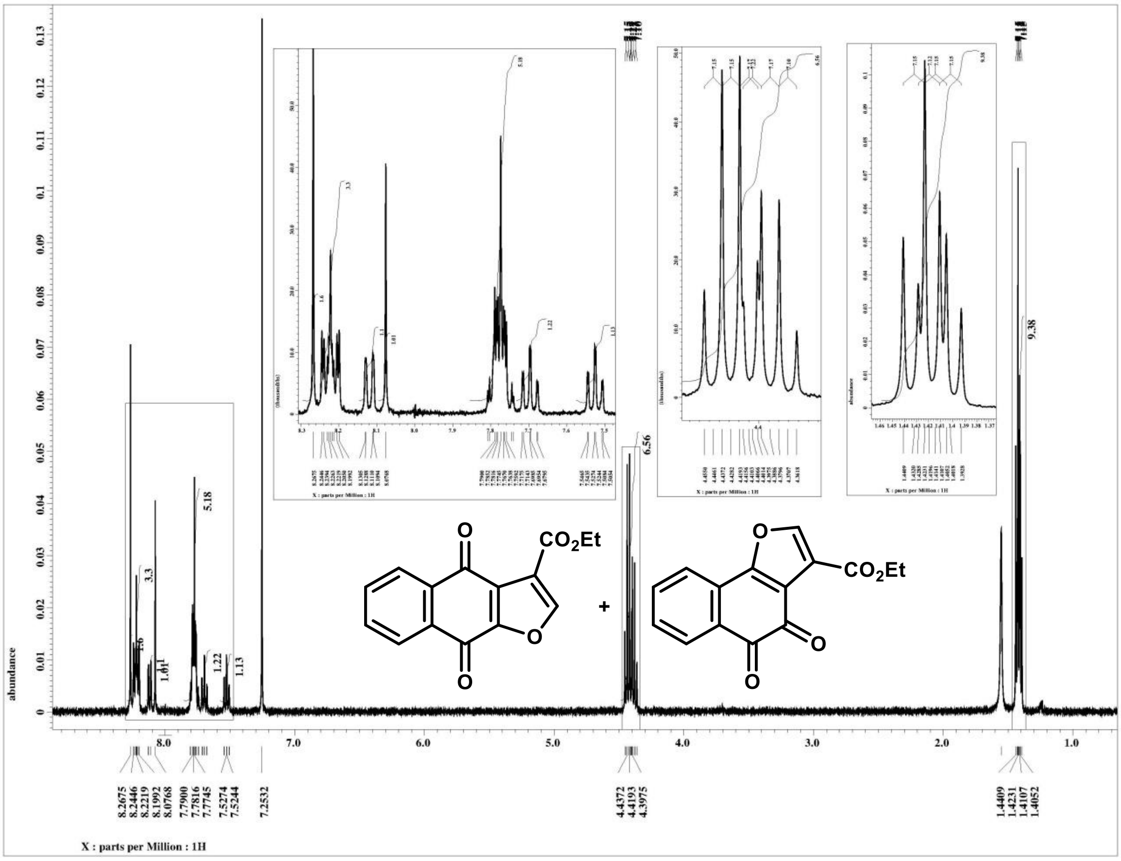

Figure S47. ^{1}H NMR spectrum of the mixture ethyl 4,9-dioxo-4,9-dihydronaphtho[2,3-*b*]furan-3-carboxylate (**3b**) and ethyl 4,5-dioxo-4,5-dihydronaphtho[1,2-*b*]furan-3-carboxylate (**4b**) in $CDCl_3$

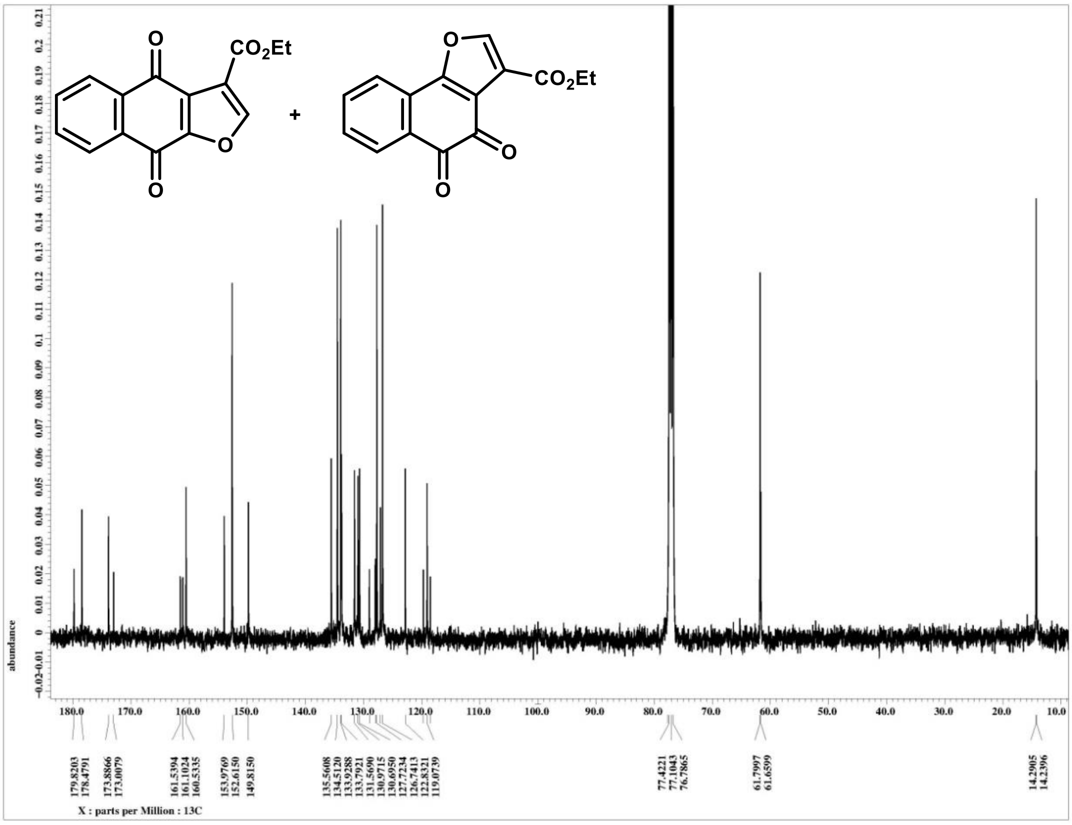

Figure S48. $^{13}C\{^{1}H\}$ NMR spectrum of the mixture ethyl 4,9-dioxo-4,9-dihydronaphtho[2,3-*b*]furan-3-carboxylate (**3b**) and ethyl 4,5-dioxo-4,5-dihydronaphtho[1,2-*b*]furan-3-carboxylate (**4b**) in $CDCl_3$

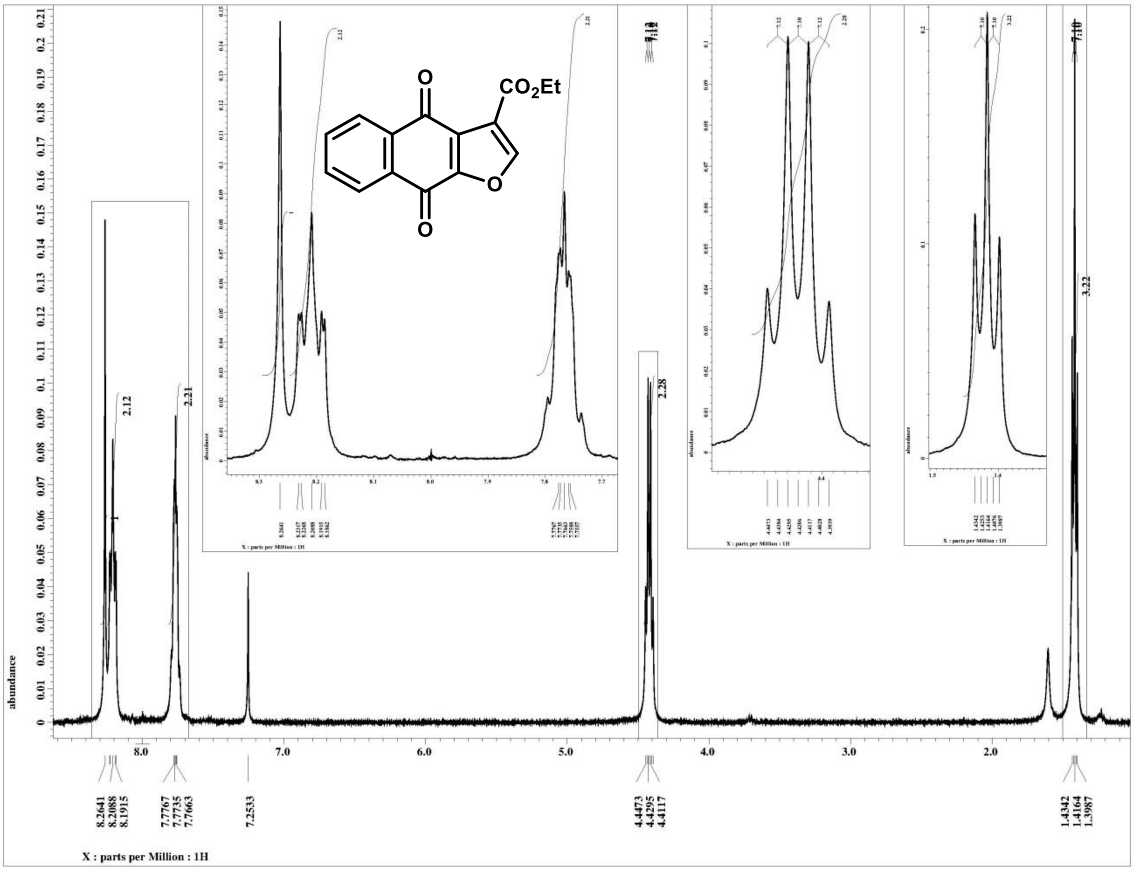

Figure S49. ^{1}H NMR spectrum of ethyl 4,9-dioxo-4,9-dihydronaphtho[2,3-*b*]furan-3-carboxylate (**3b**) in $CDCl_3$

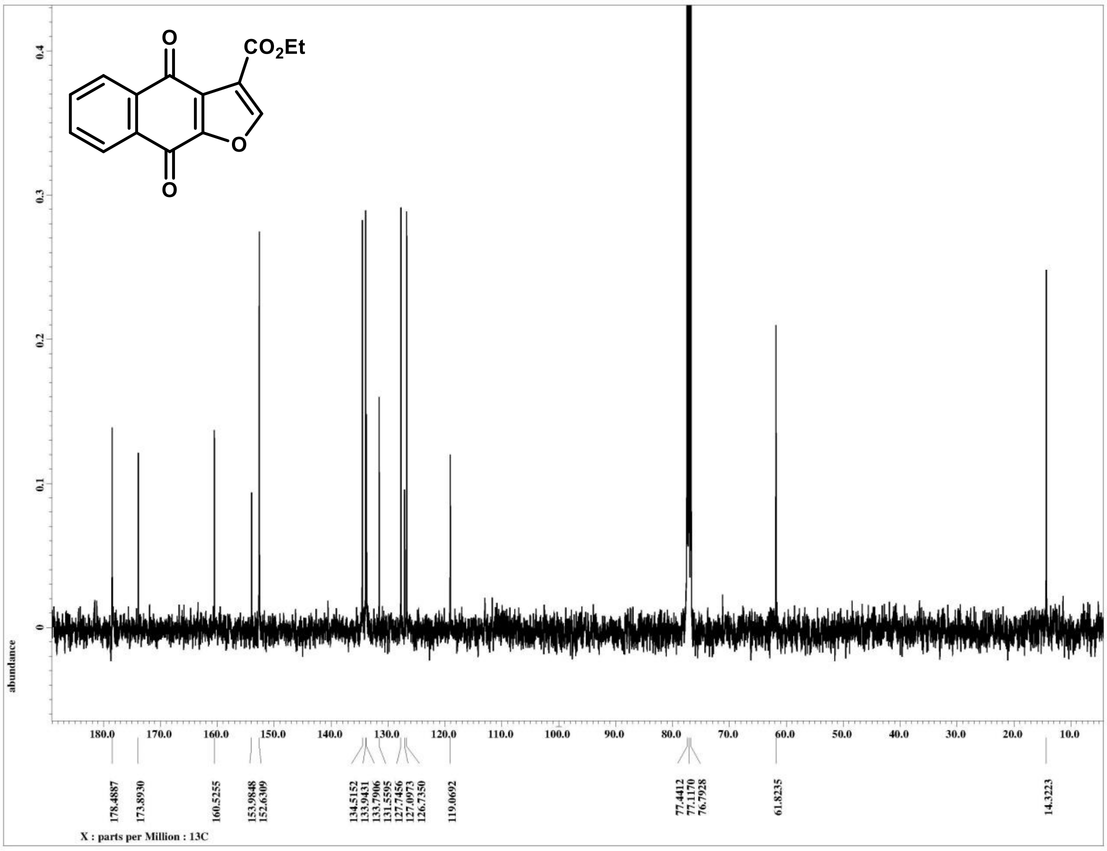

Figure S50. $^{13}C\{^{1}H\}$ NMR spectrum of ethyl 4,9-dioxo-4,9-dihydronaphtho[2,3-*b*]furan-3-carboxylate (**3b**) in $CDCl_3$

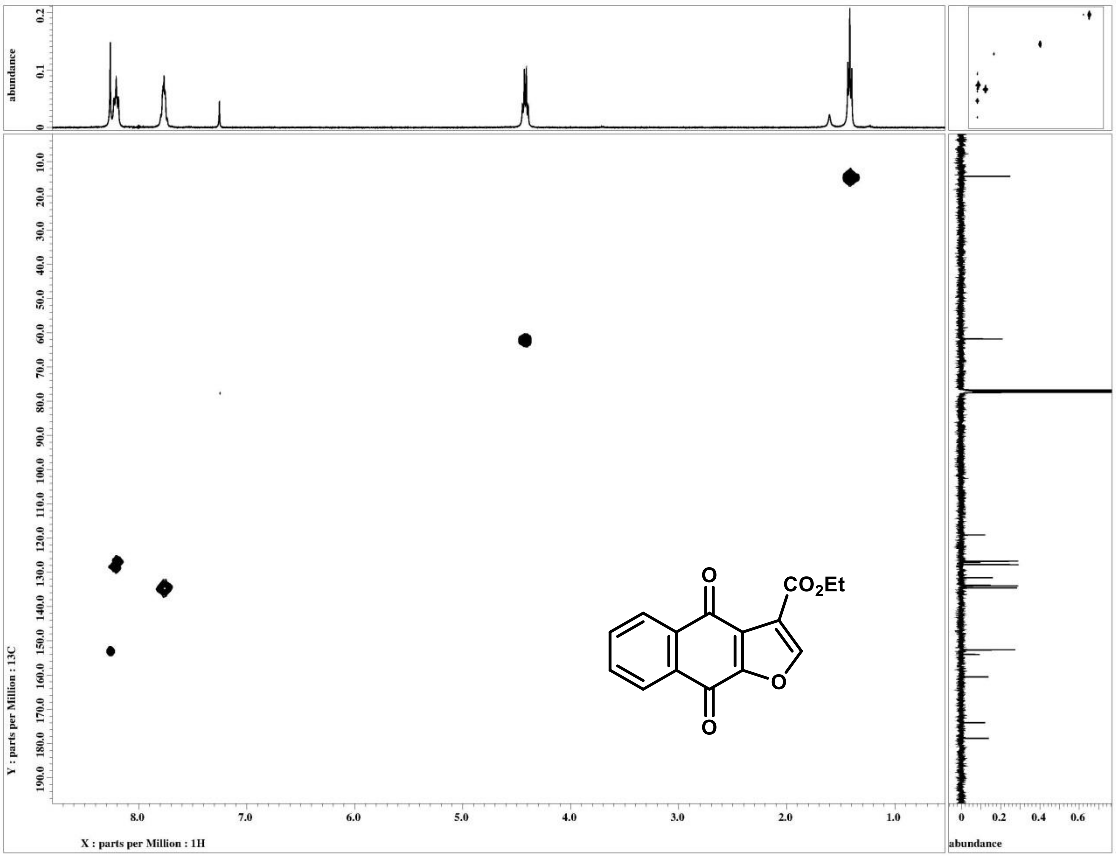

Figure S51. ^{1}H-^{13}C HMQC spectrum of ethyl 4,9-dioxo-4,9-dihydronaphtho[2,3-*b*]furan-3-carboxylate (**3b**) in $CDCl_3$

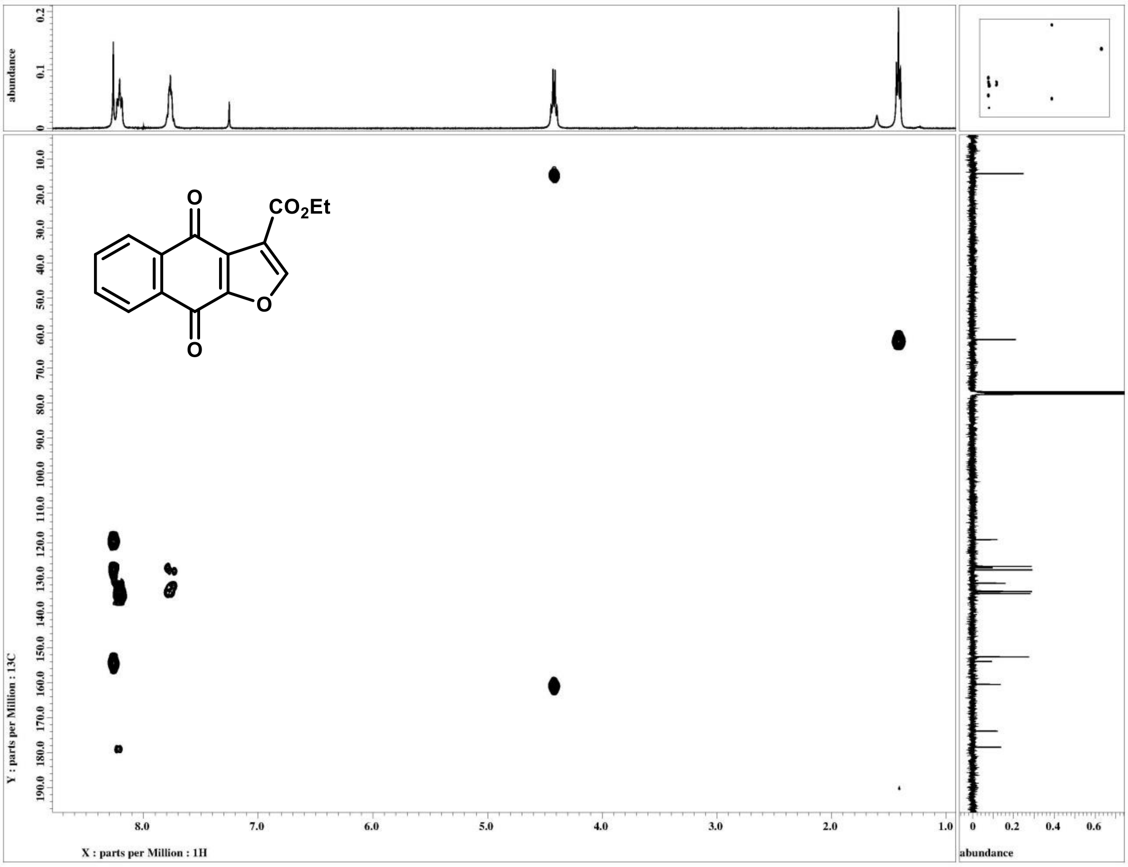

Figure S52. ^{1}H-^{13}C HMBC spectrum of ethyl 4,9-dioxo-4,9-dihydronaphtho[2,3-*b*]furan-3-carboxylate (**3b**) in $CDCl_3$

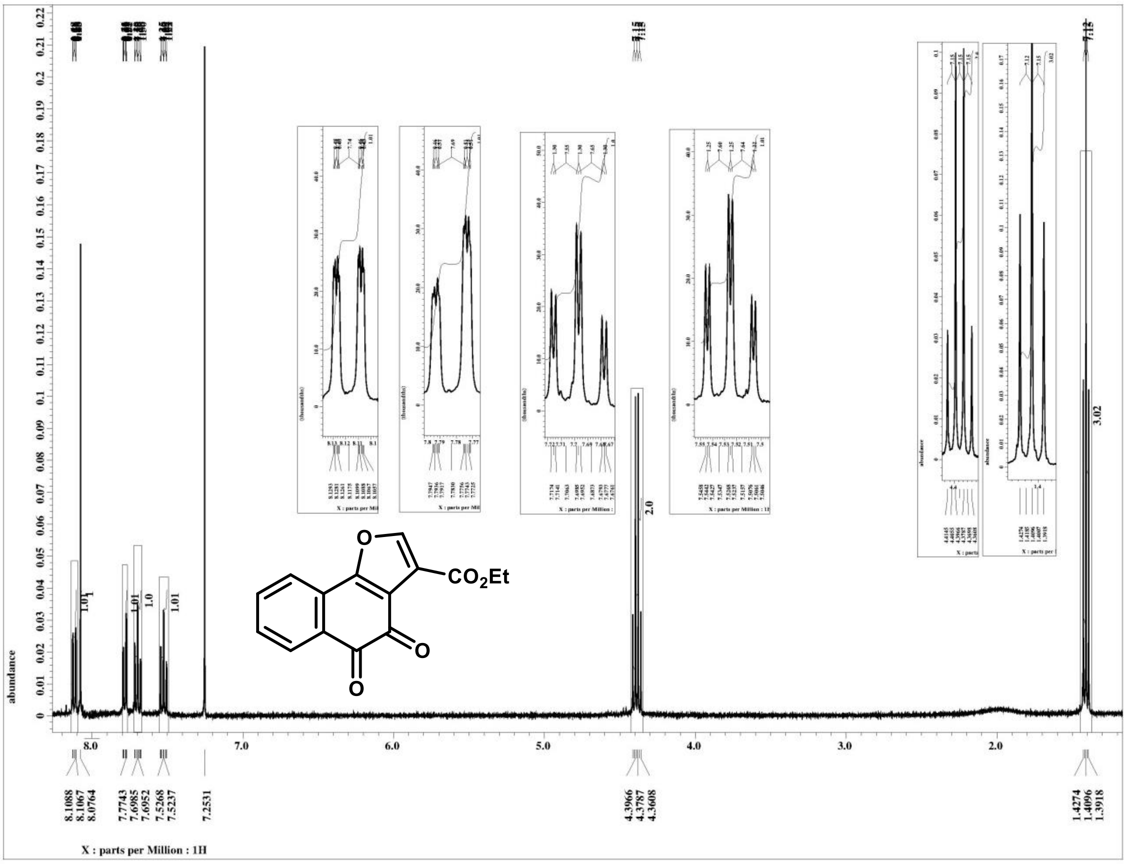

Figure S53. ^{1}H NMR spectrum of ethyl 4,5-dioxo-4,5-dihydronaphtho[1,2-*b*]furan-3-carboxylate (**4b**) in $CDCl_3$

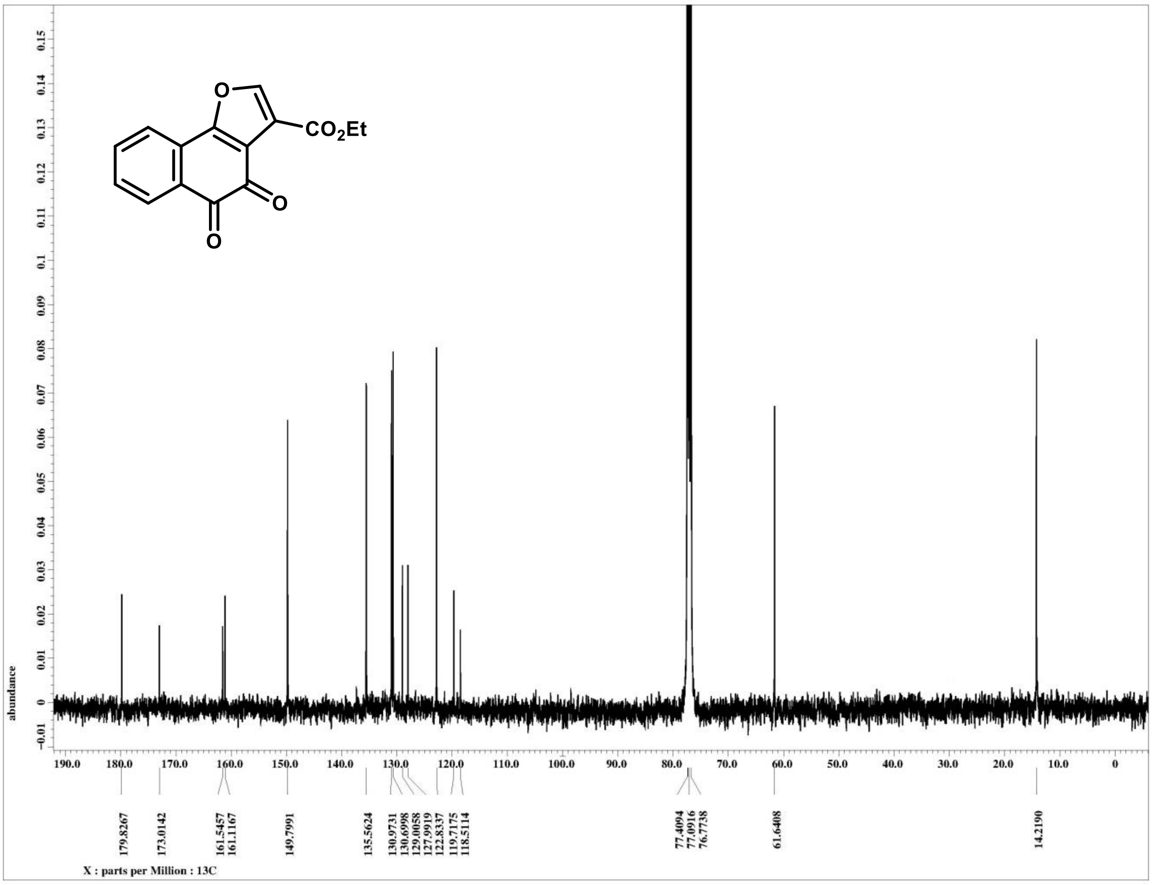

Figure S54. $^{13}C\{^{1}H\}$ NMR spectrum of ethyl 4,5-dioxo-4,5-dihydronaphtho[1,2-*b*]furan-3-carboxylate (**4b**) in $CDCl_3$

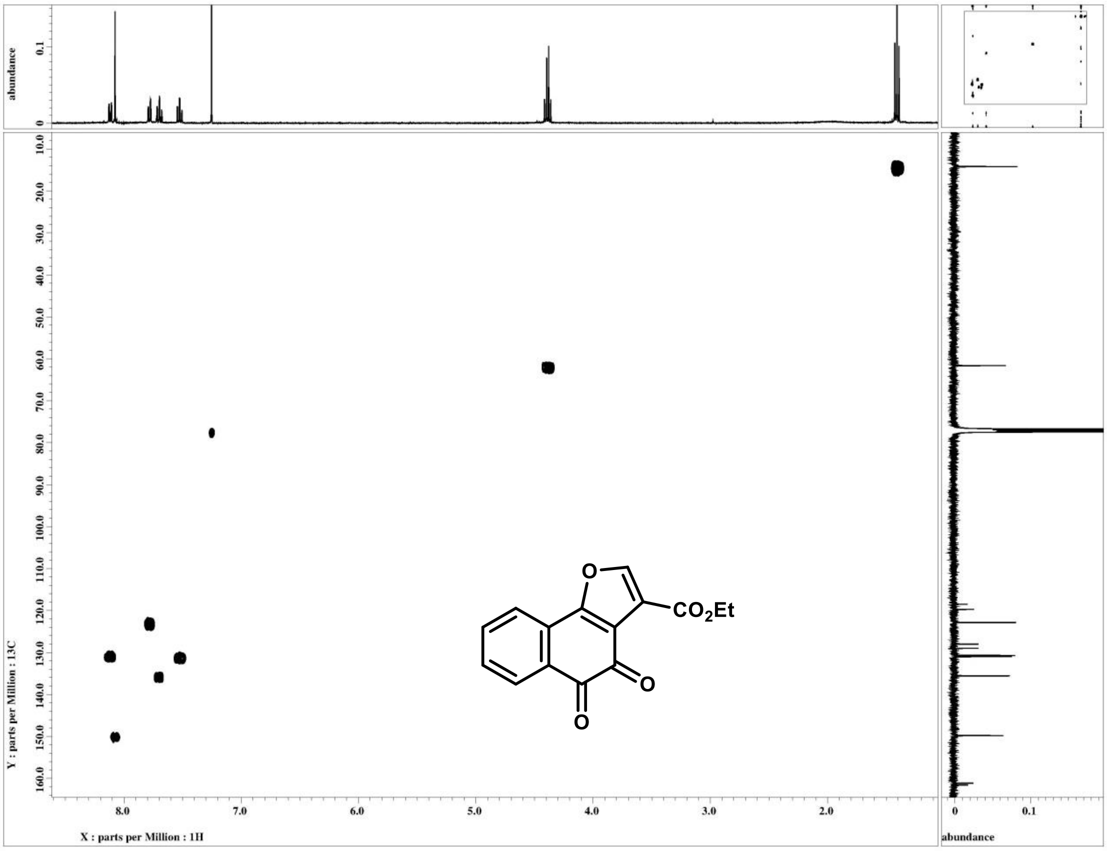

Figure S55. ^{1}H-^{13}C HMQC spectrum of ethyl 4,5-dioxo-4,5-dihydronaphtho[1,2-*b*]furan-3-carboxylate (**4b**) in $CDCl_3$

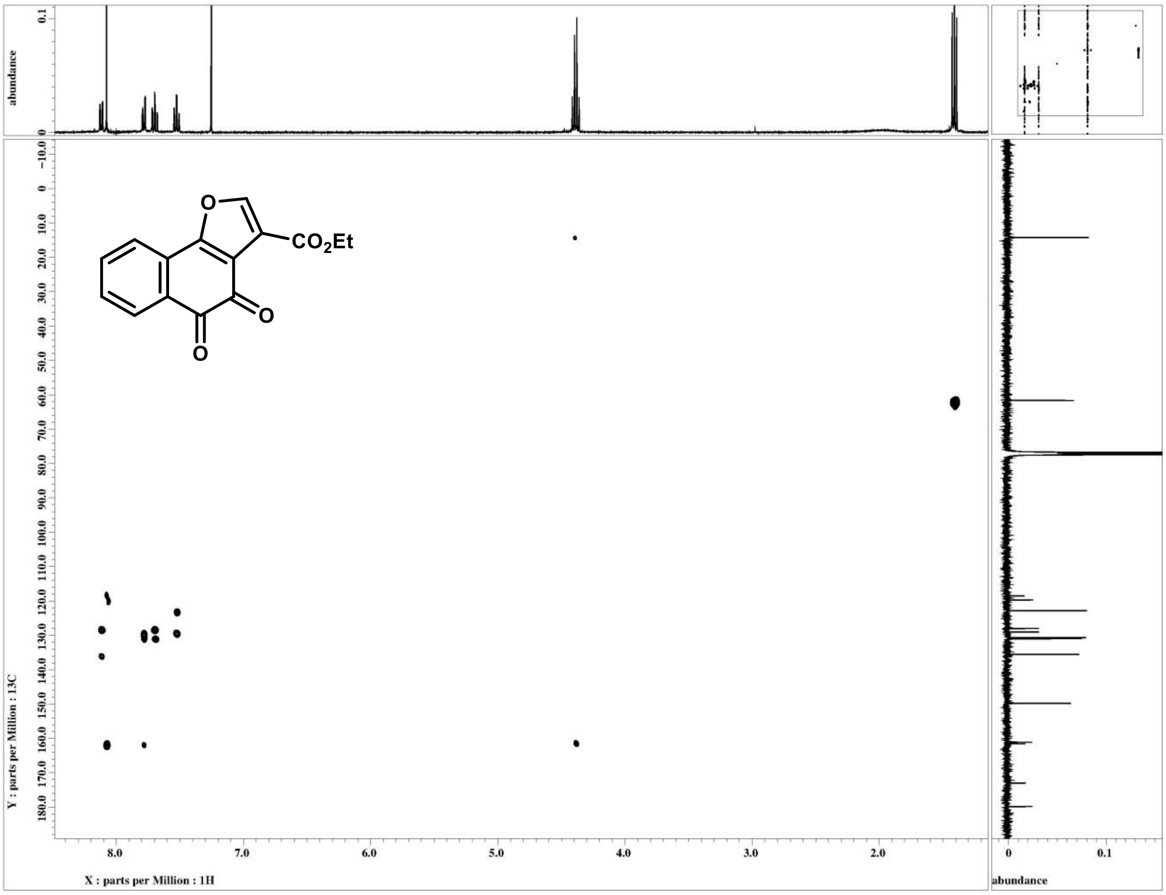

Figure S56. ^{1}H-^{13}C HMBC spectrum of ethyl 4,5-dioxo-4,5-dihydronaphtho[1,2-*b*]furan-3-carboxylate (**4b**) in $CDCl_3$

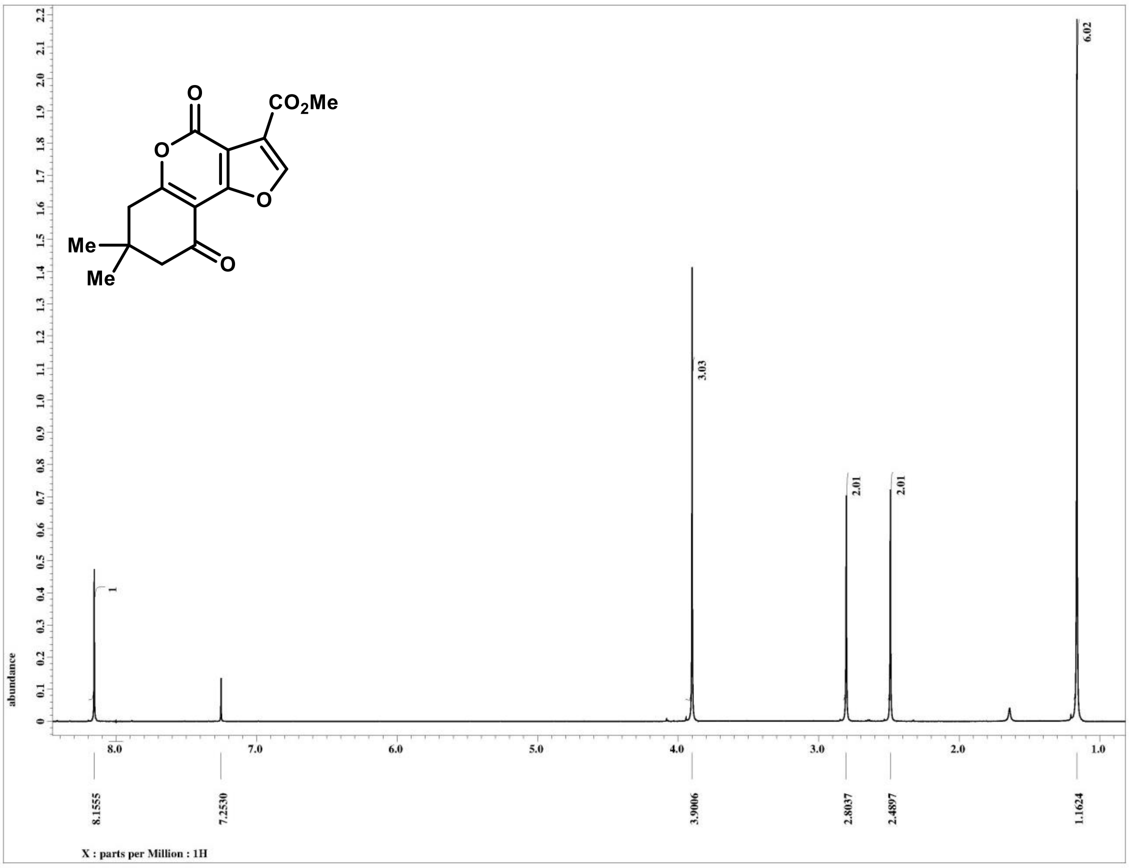

Figure S57. ^{1}H NMR spectrum of methyl 7,7-dimethyl-4,9-dioxo-6,7,8,9-tetrahydro-4*H*-furo[3,2-*c*][1]benzopyran-3-carboxylate (**5a**) in $CDCl_3$

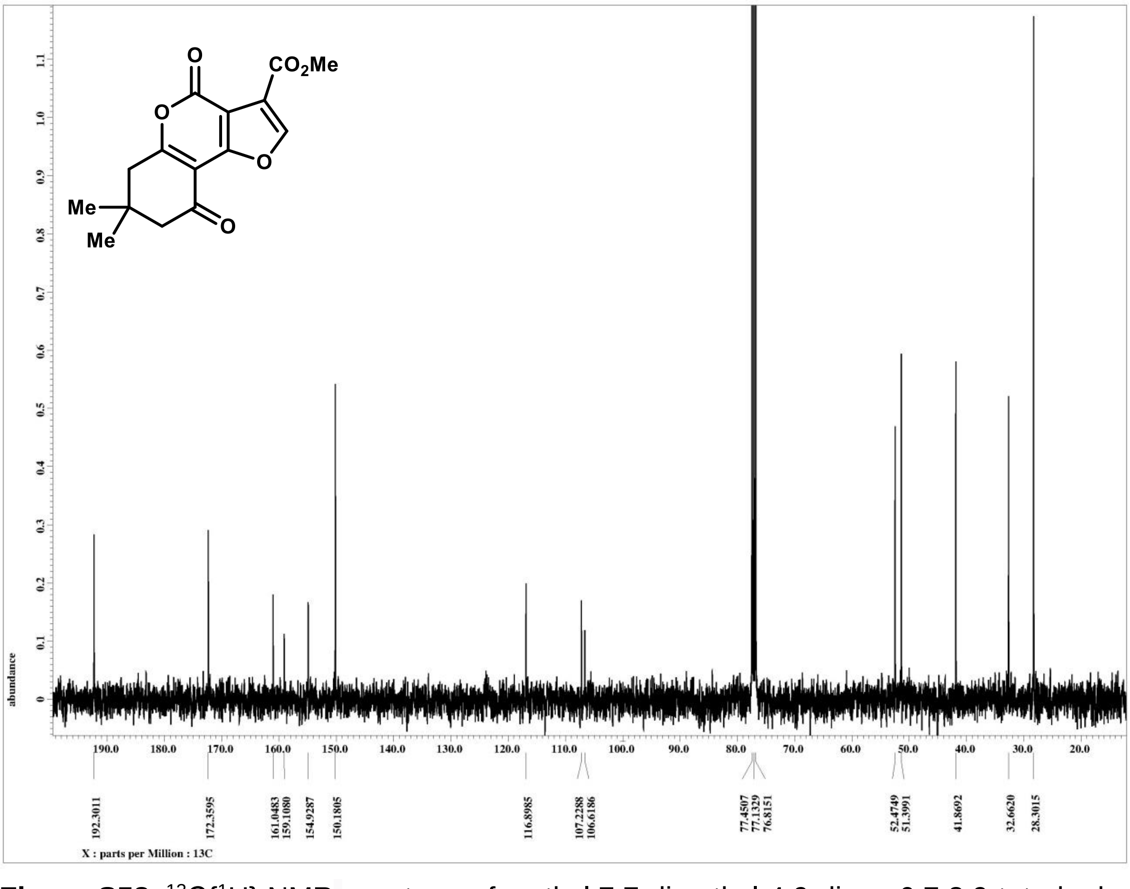

Figure S58. $^{13}C\{^{1}H\}$ NMR spectrum of methyl 7,7-dimethyl-4,9-dioxo-6,7,8,9-tetrahydro-4*H*-furo[3,2-*c*][1]benzopyran-3-carboxylate (**5a**) in $CDCl_3$

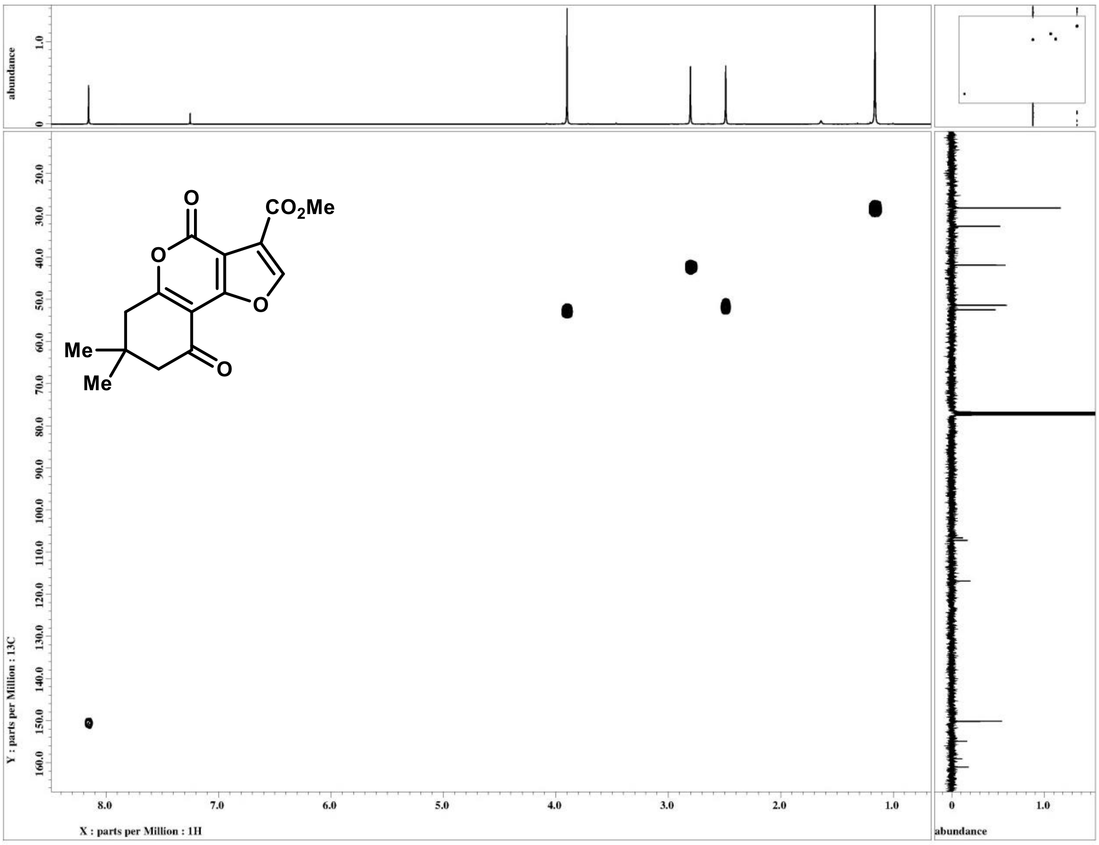

Figure S59. ^{1}H-^{13}C HMQC spectrum of methyl 7,7-dimethyl-4,9-dioxo-6,7,8,9-tetrahydro-4*H*-furo[3,2-*c*][1]benzopyran-3-carboxylate (**5a**) in $CDCl_3$

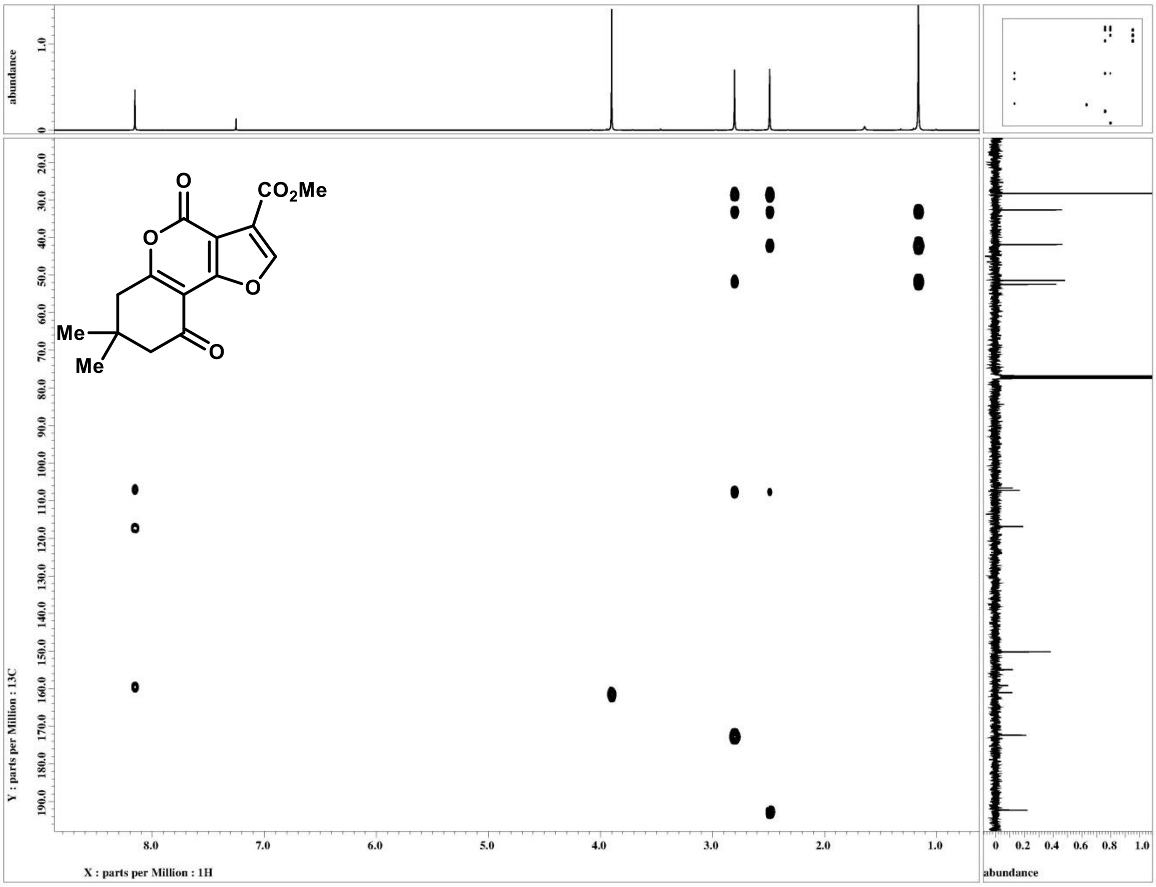

Figure S60. ^{1}H-^{13}C HMBC spectrum of methyl 7,7-dimethyl-4,9-dioxo-6,7,8,9-tetrahydro-4*H*-furo[3,2-*c*][1]benzopyran-3-carboxylate (**5a**) in $CDCl_3$

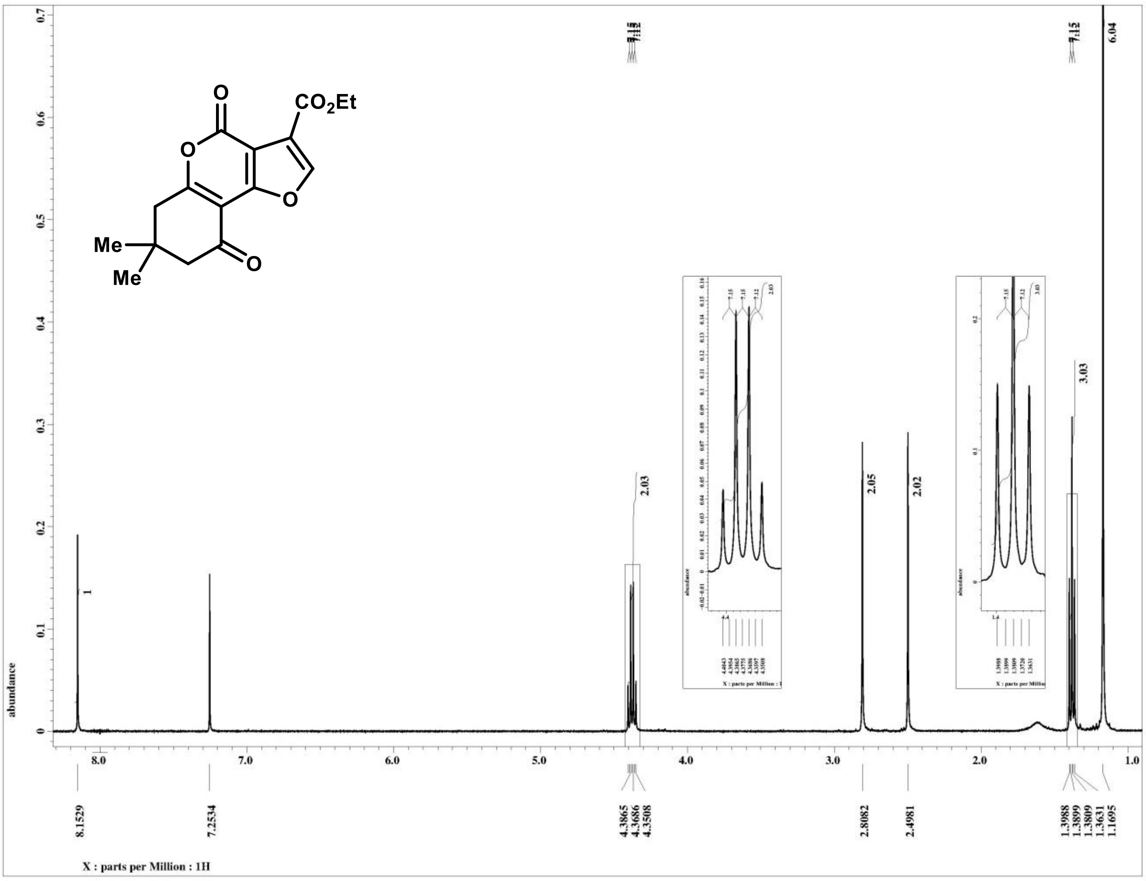

Figure S61. ^{1}H NMR spectrum of ethyl 7,7-dimethyl-4,9-dioxo-6,7,8,9-tetrahydro-4*H*-furo[3,2-*c*][1]benzopyran-3-carboxylate (**5b**) in $CDCl_3$

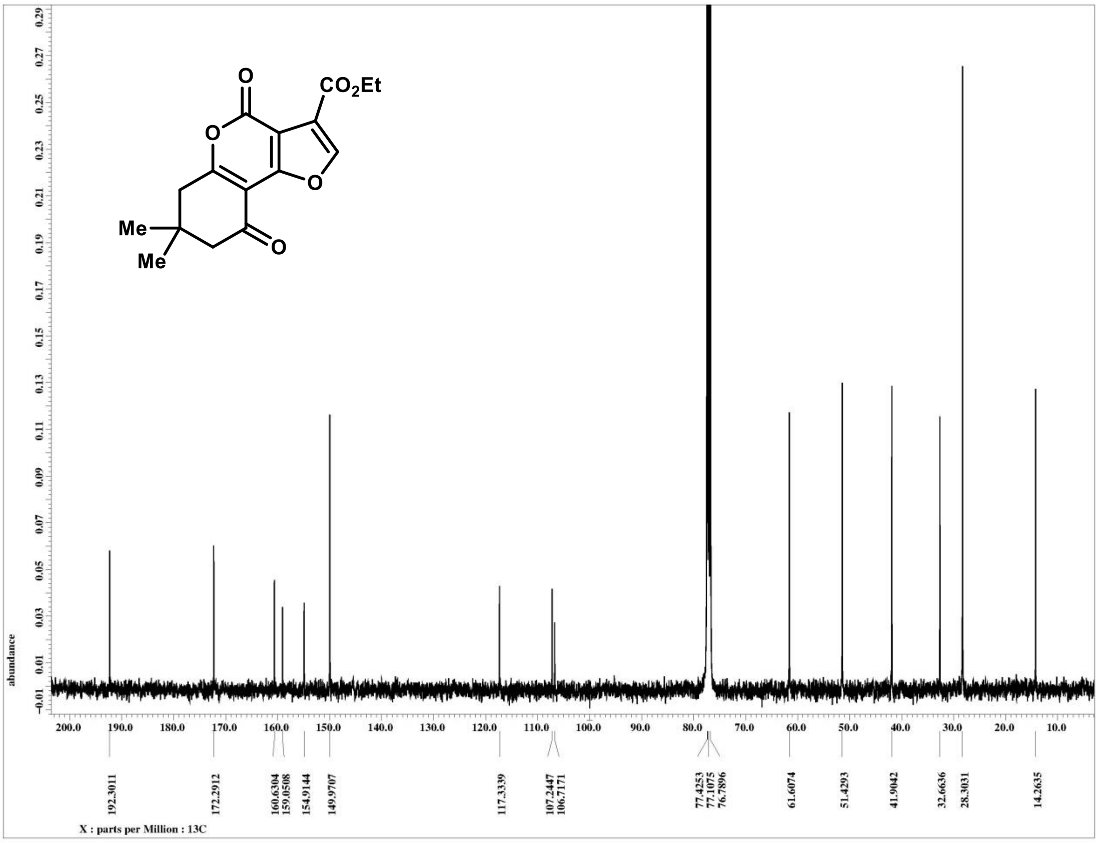

Figure S62. ^{13}C{^{1}H} NMR spectrum of ethyl 7,7-dimethyl-4,9-dioxo-6,7,8,9-tetrahydro-4*H*-furo[3,2-*c*][1]benzopyran-3-carboxylate (**5b**) in $CDCl_3$

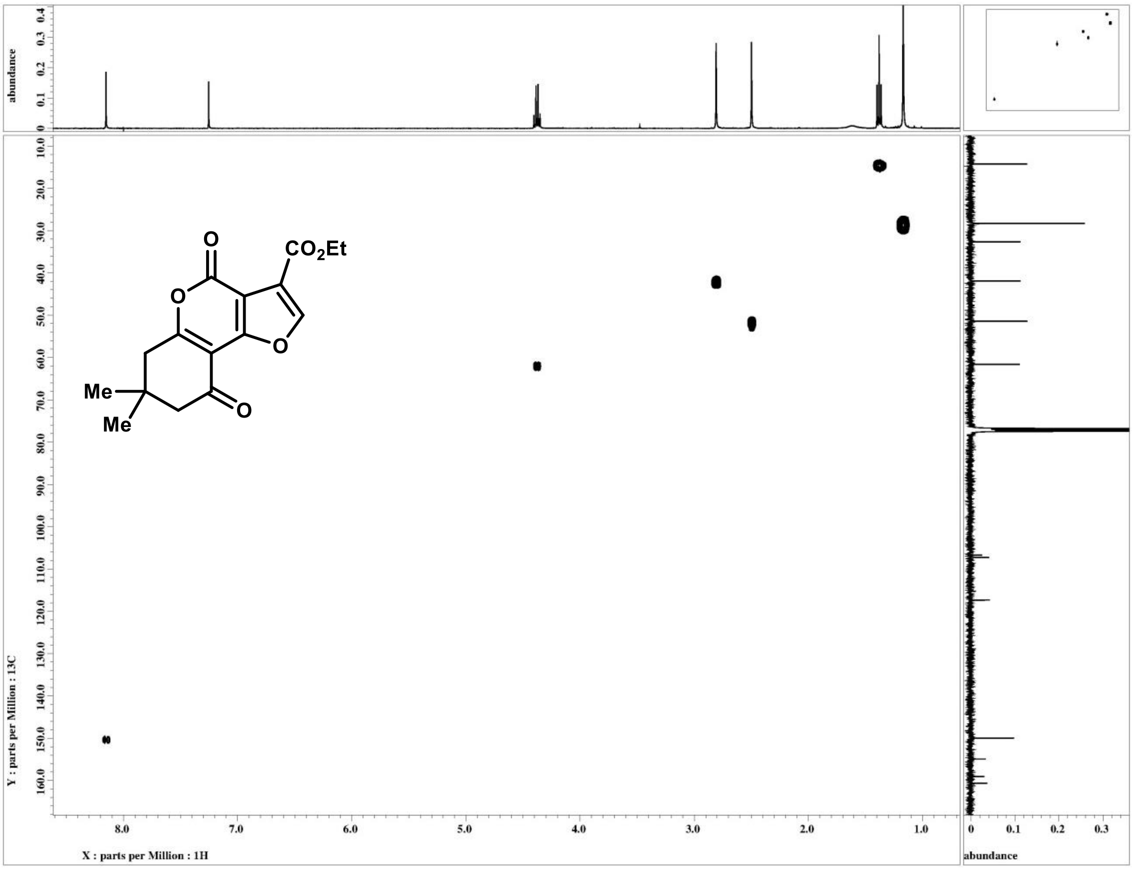

Figure S63. ^{1}H-^{13}C HMQC spectrum of ethyl 7,7-dimethyl-4,9-dioxo-6,7,8,9-tetrahydro-4*H*-furo[3,2-*c*][1]benzopyran-3-carboxylate (**5b**) in $CDCl_3$

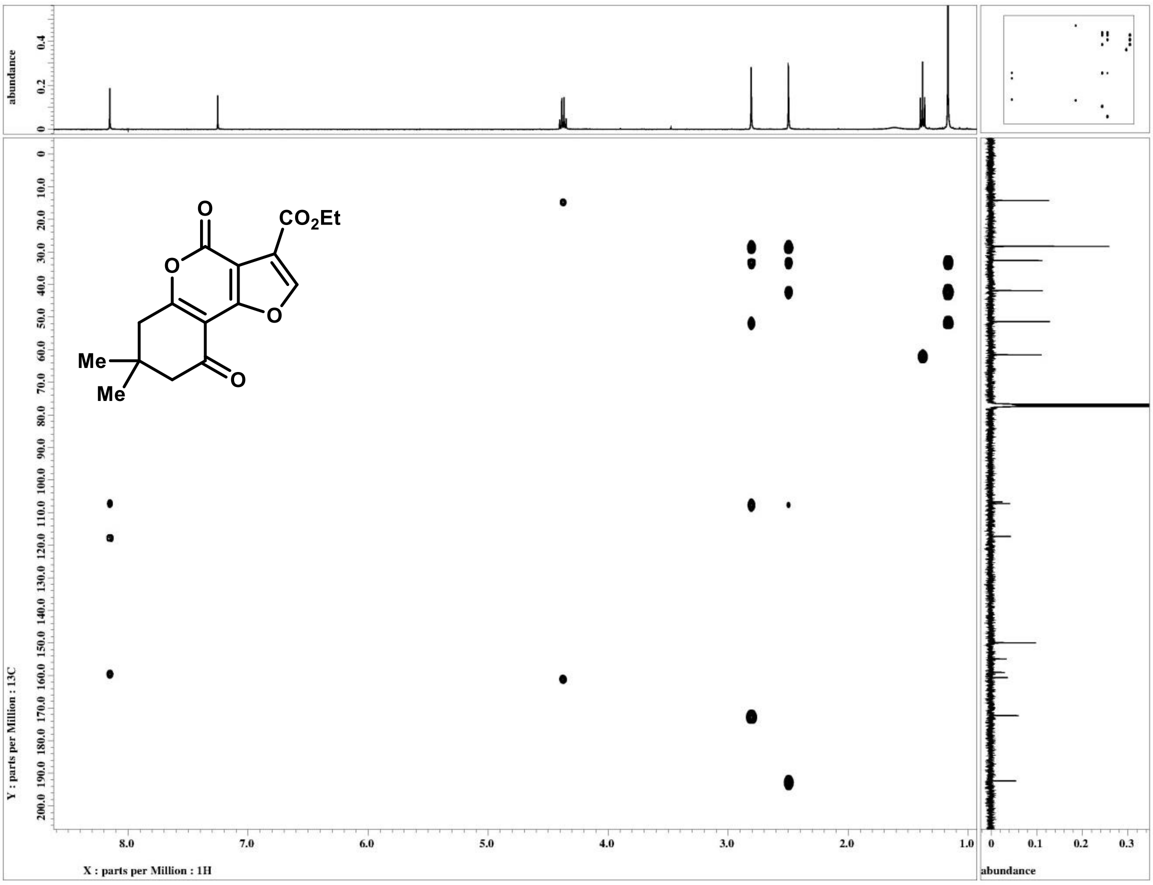

Figure S64. ^{1}H-^{13}C HMBC spectrum of ethyl 7,7-dimethyl-4,9-dioxo-6,7,8,9-tetrahydro-4*H*-furo[3,2-*c*][1]benzopyran-3-carboxylate (**5b**) in $CDCl_3$

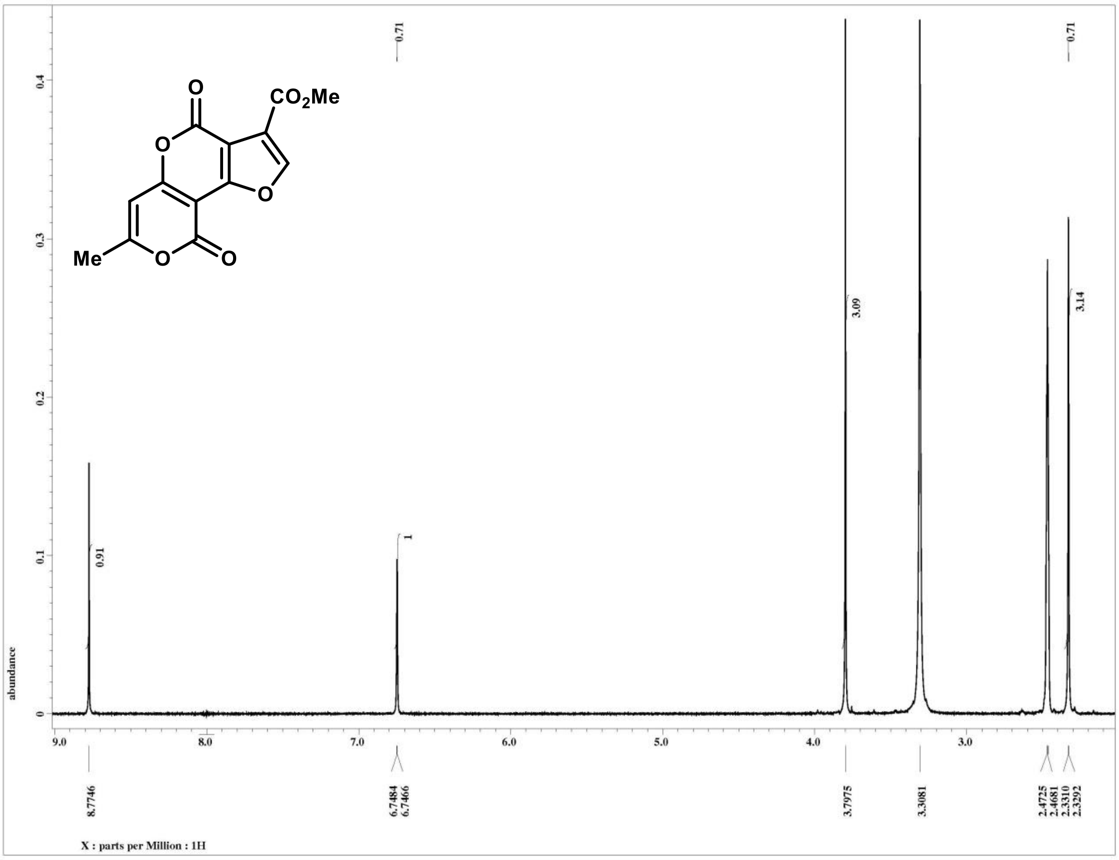

Figure S65. ^{1}H NMR spectrum of methyl 7-methyl-4,9-dioxo-4*H*,9*H*-furo[2,3-*d*]pyrano[4,3-*b*]pyran-3-carboxylate (**6a**) in DMSO-d_6

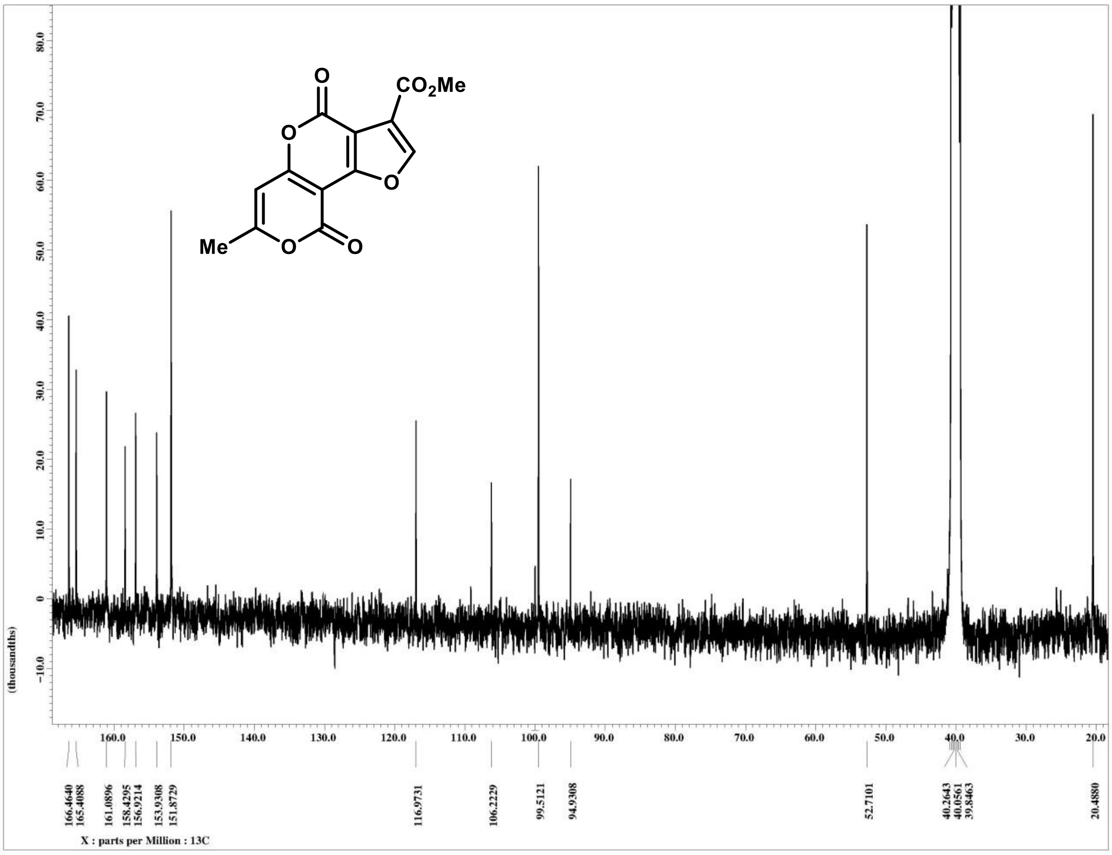

Figure S66. ^{13}C{^{1}H} NMR spectrum of methyl 7-methyl-4,9-dioxo-4*H*,9*H*-furo[2,3-*d*]pyrano[4,3-*b*]pyran-3-carboxylate (**6a**) in DMSO-d_6

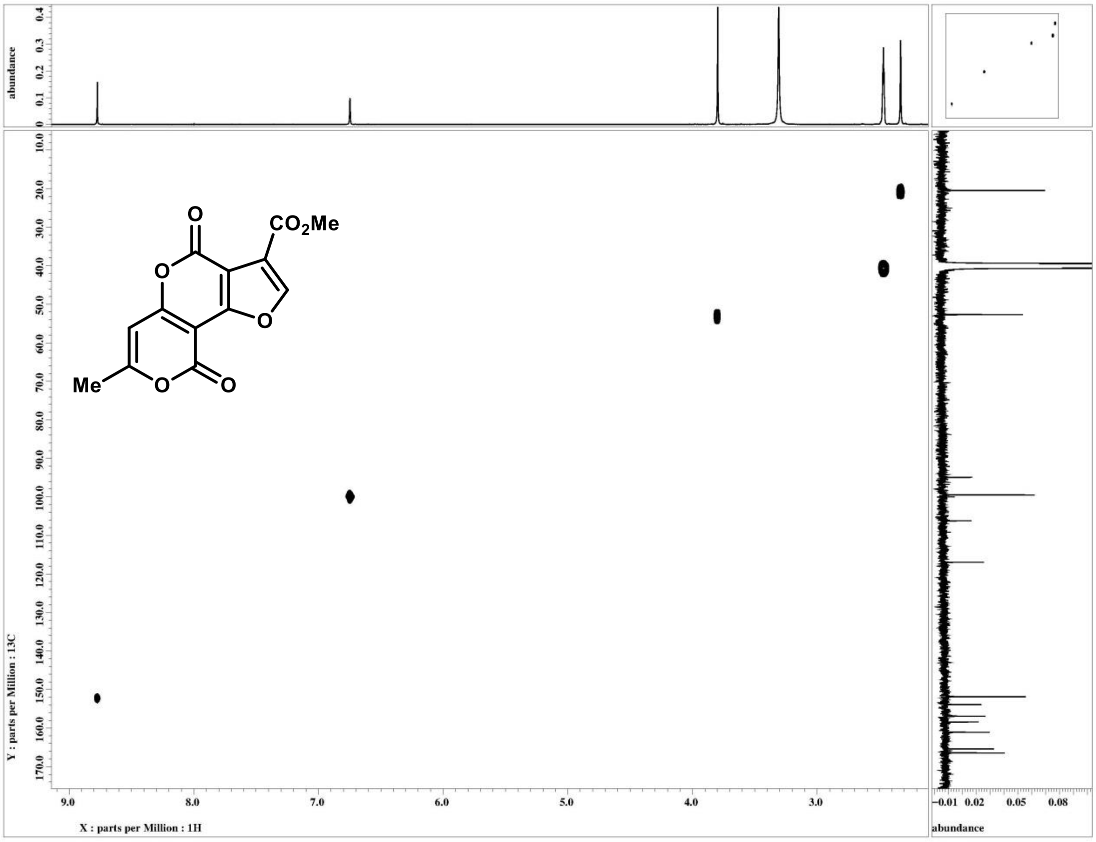

Figure S67. ^{1}H-^{13}C HMQC spectrum of methyl 7-methyl-4,9-dioxo-4*H*,9*H*-furo[2,3-*d*]pyrano[4,3-*b*]pyran-3-carboxylate (**6a**) in DMSO-d_6

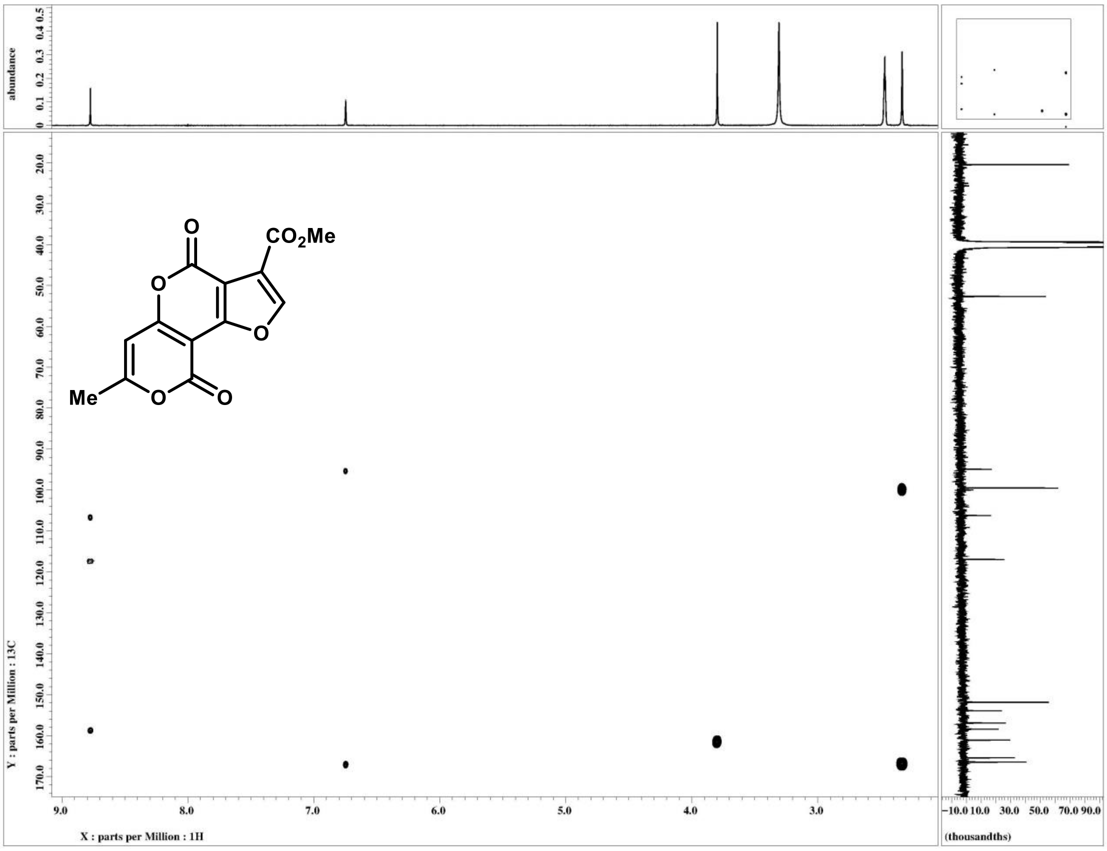

Figure S68. ^{1}H-^{13}C HMBC spectrum of methyl 7-methyl-4,9-dioxo-4*H*,9*H*-furo[2,3-*d*]pyrano[4,3-*b*]pyran-3-carboxylate (**6a**) in DMSO-d_6

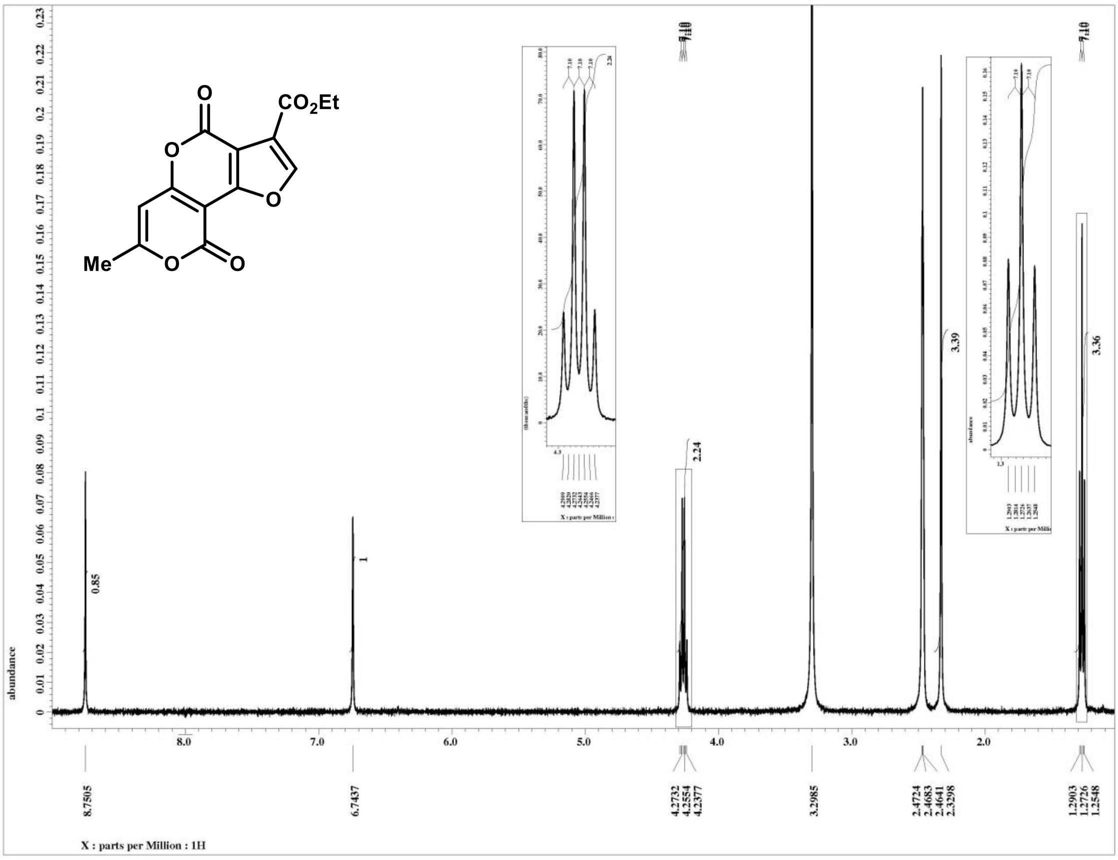

Figure S69. ^{1}H NMR spectrum of ethyl 7-methyl-4,9-dioxo-4*H*,9*H*-furo[2,3-*d*]pyrano[4,3-*b*]pyran-3-carboxylate (**6b**) in DMSO-d_6

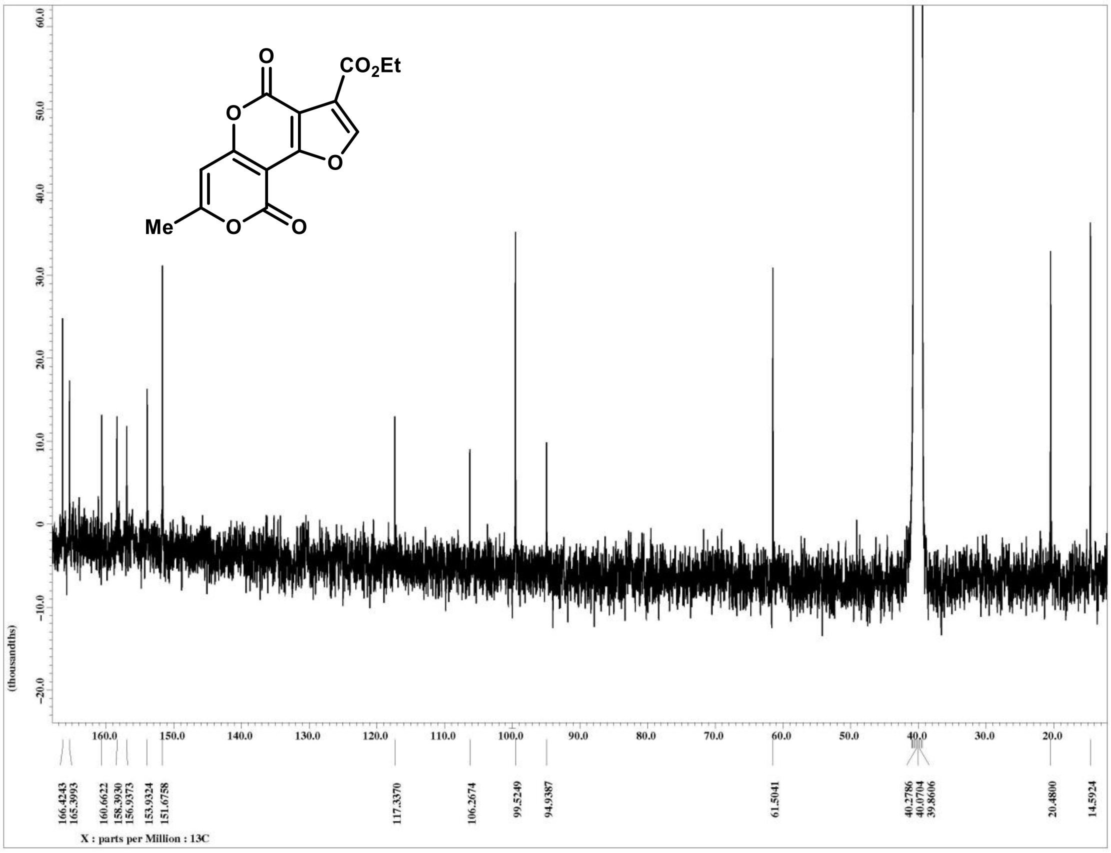

Figure S70. ^{13}C{^{1}H} NMR spectrum of ethyl 7-methyl-4,9-dioxo-4*H*,9*H*-furo[2,3-*d*]pyrano[4,3-*b*]pyran-3-carboxylate (**6b**) in DMSO-d_6

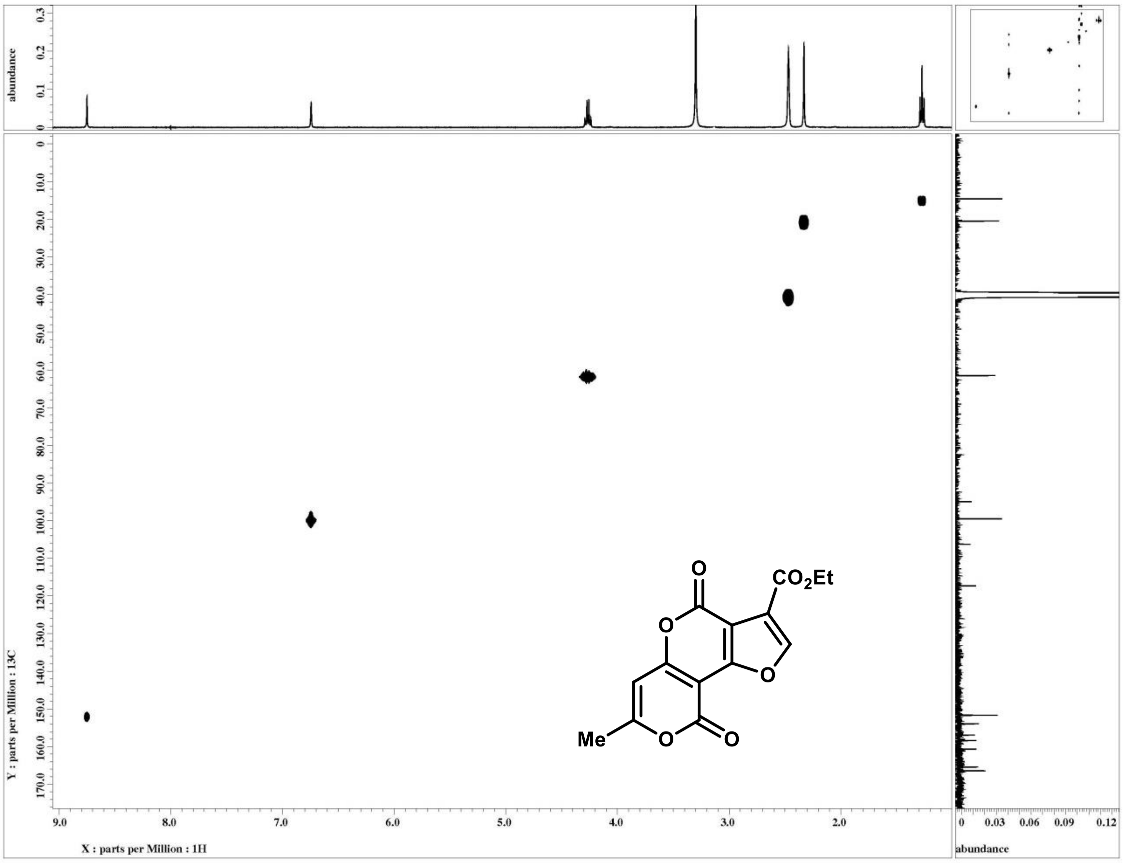

Figure S71. ^{1}H-^{13}C HMQC spectrum of ethyl 7-methyl-4,9-dioxo-4*H*,9*H*-furo[2,3-*d*]pyrano[4,3-*b*]pyran-3-carboxylate (**6b**) in DMSO-d_6

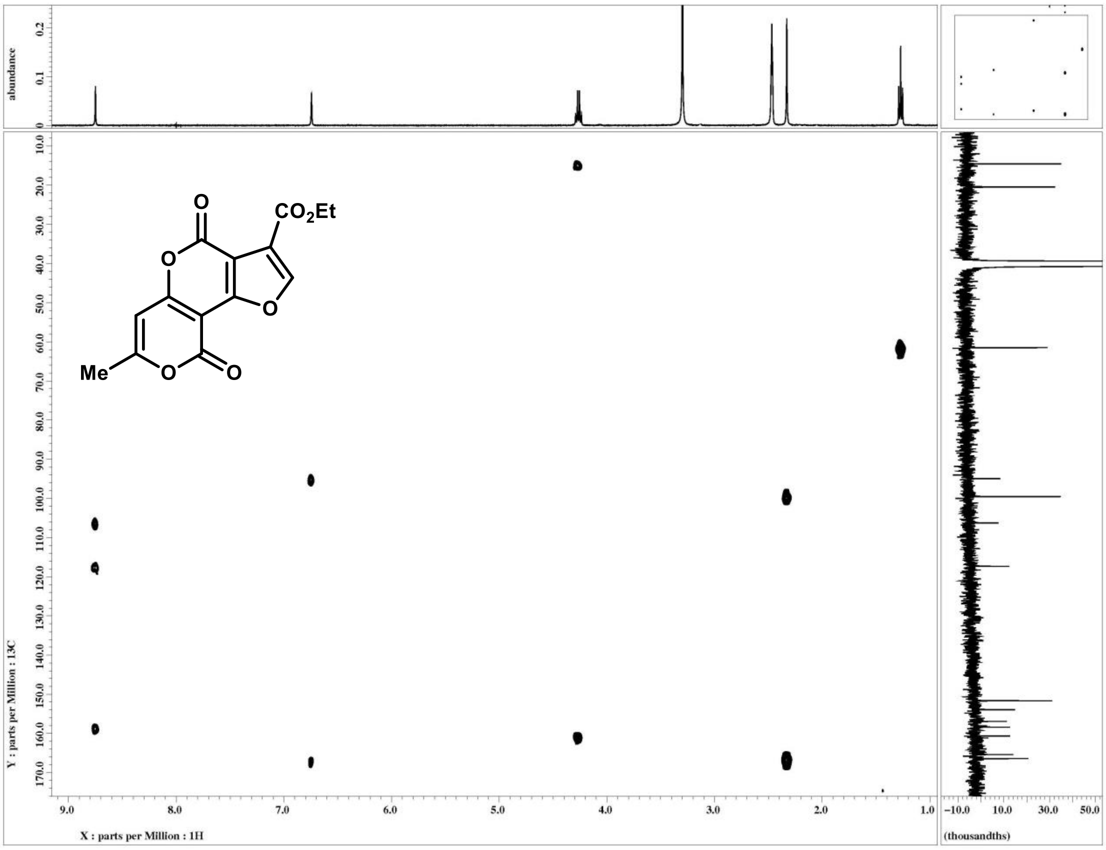

Figure S72. ^{1}H-^{13}C HMBC spectrum of ethyl 7-methyl-4,9-dioxo-4*H*,9*H*-furo[2,3-*d*]pyrano[4,3-*b*]pyran-3-carboxylate (**6b**) in DMSO-d_6

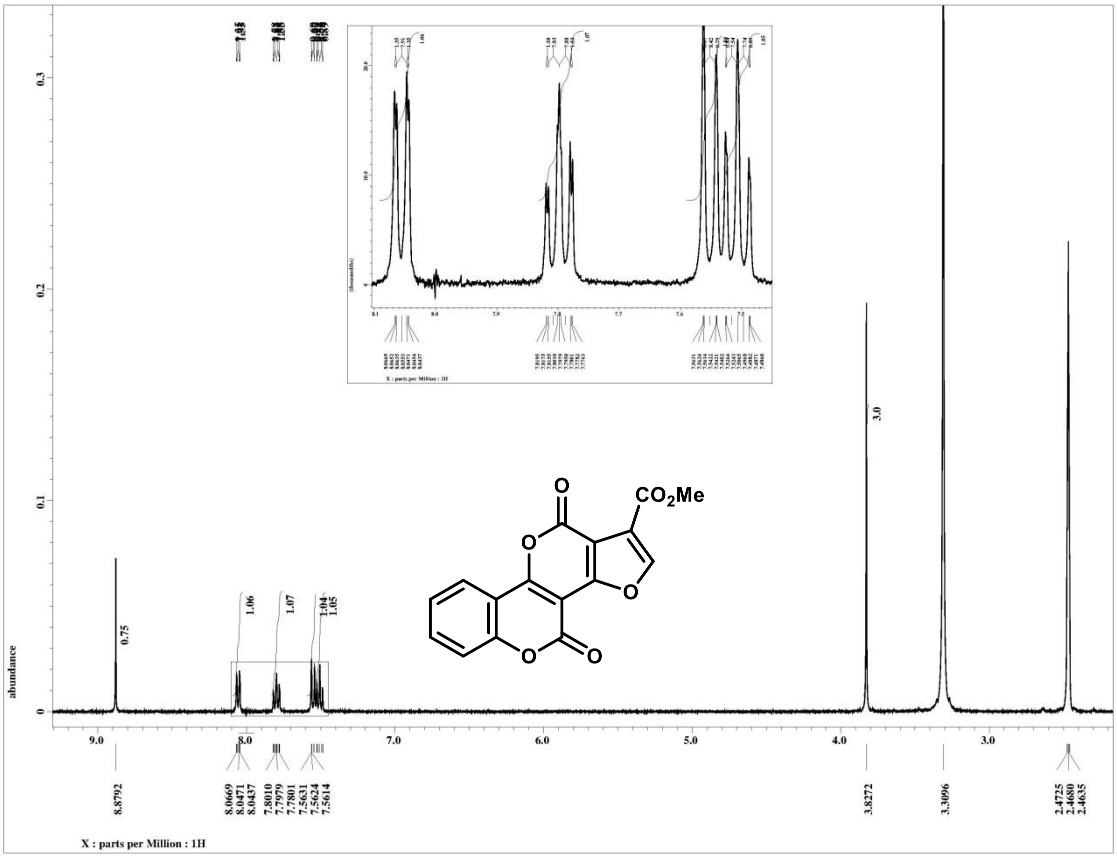

Figure S73. ^{1}H NMR spectrum of methyl 4,11-dioxo-4*H*,11*H*-furo[2',3':4,5]pyrano[3,2-*c*]chromene-1-carboxylate (**6c**) in DMSO-d_6

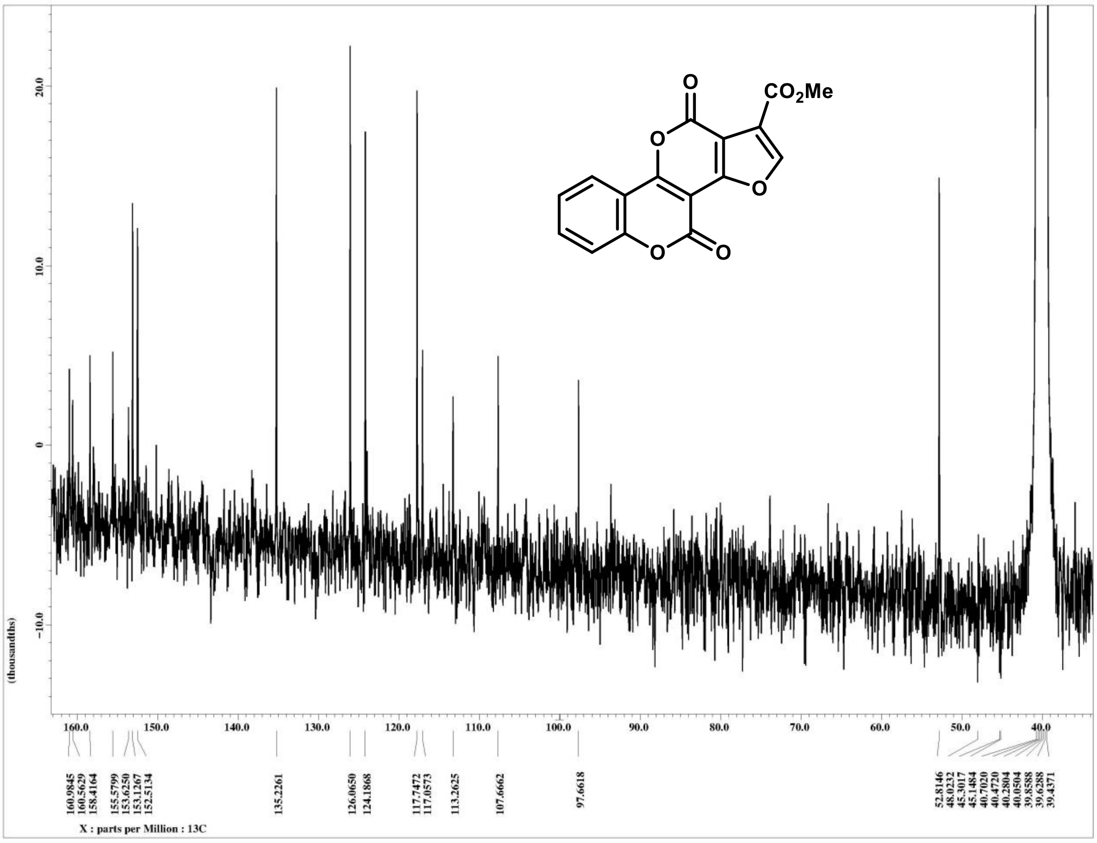

Figure S74. $^{13}C\{^{1}H\}$ NMR spectrum of methyl 4,11-dioxo-4*H*,11*H*-furo[2',3':4,5]pyrano[3,2-*c*]chromene-1-carboxylate (**6c**) in DMSO-d_6

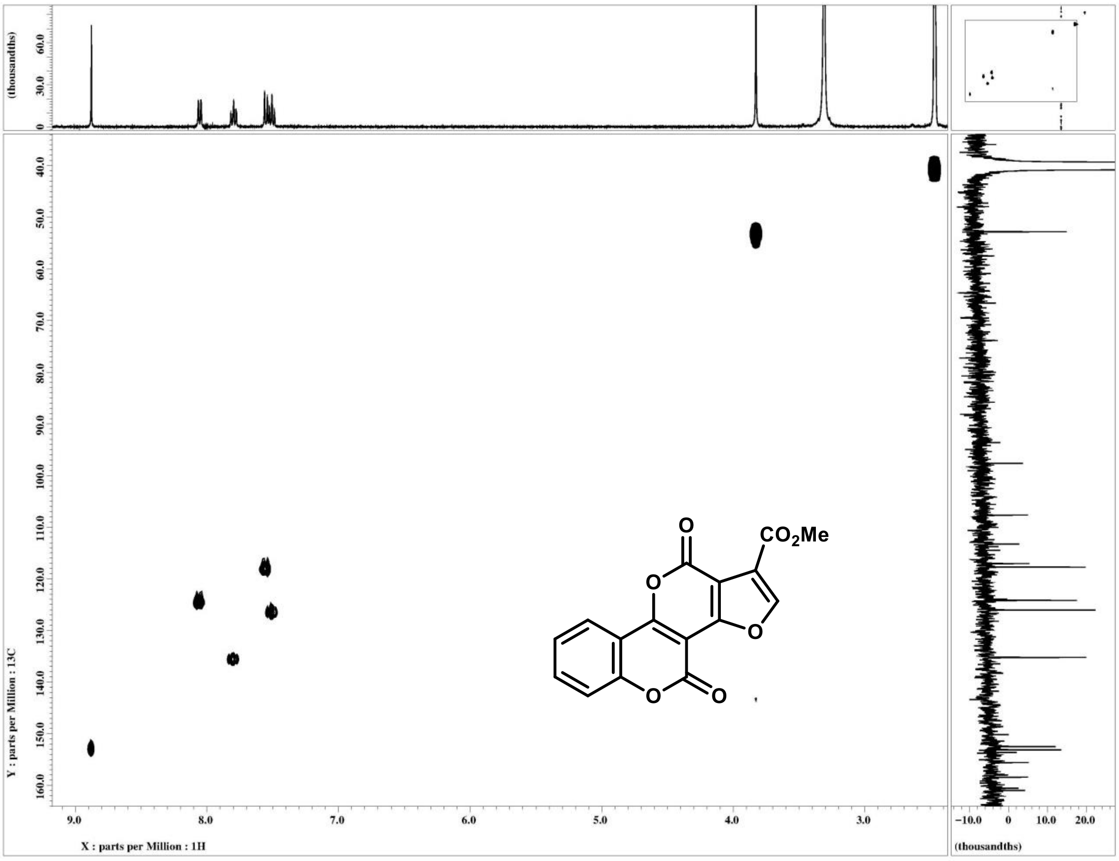

Figure S75. ^{1}H-^{13}C HMQC spectrum of methyl 4,11-dioxo-4*H*,11*H*-furo[2',3':4,5]pyrano[3,2-*c*]chromene-1-carboxylate (**6c**) in DMSO-d_6

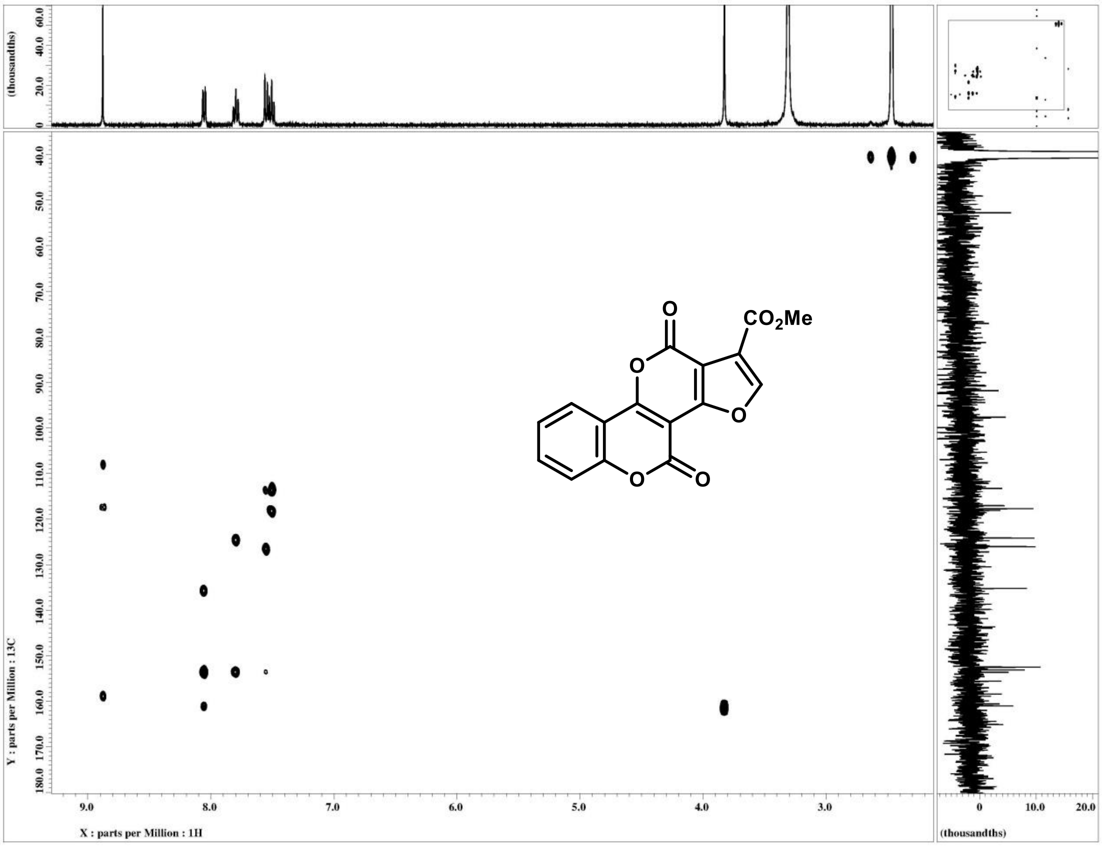

Figure S76. ^{1}H-^{13}C HMBC spectrum of methyl 4,11-dioxo-4*H*,11*H*-furo[2',3':4,5]pyrano[3,2-*c*]chromene-1-carboxylate (**6c**) in DMSO-d_6

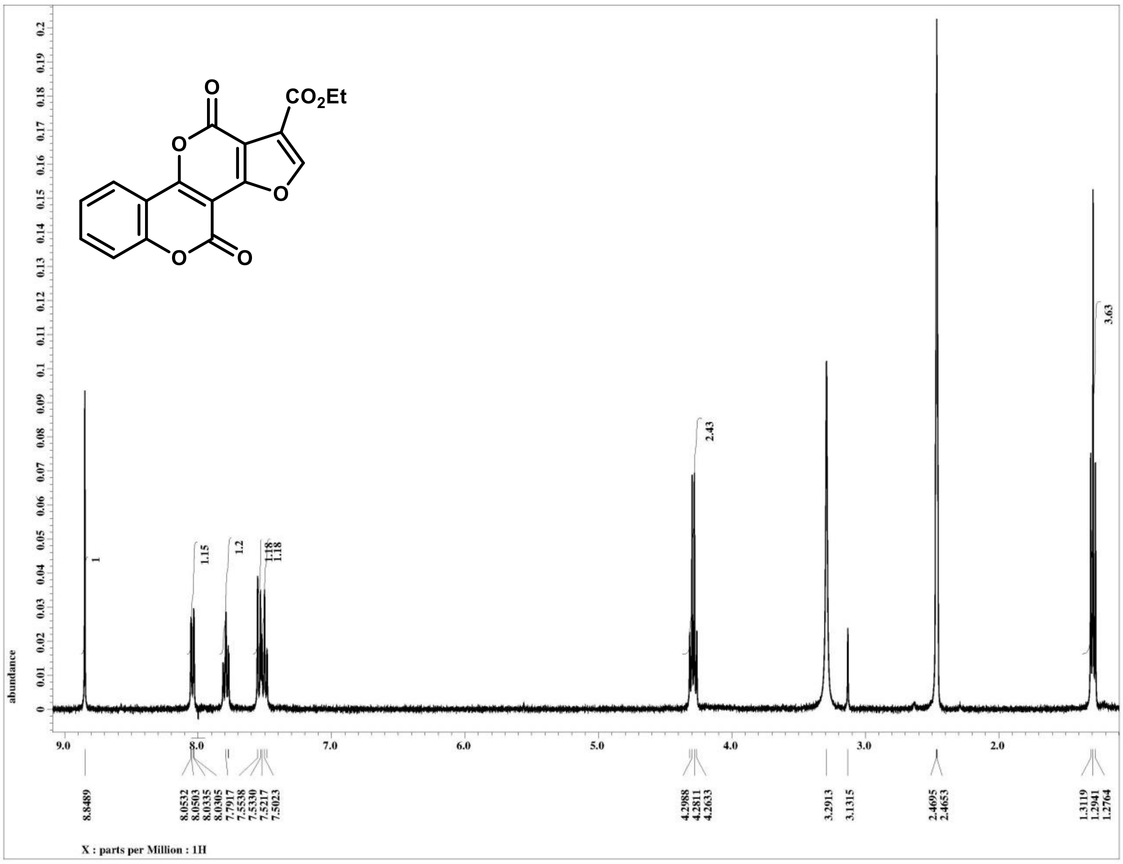

Figure S77. ^{1}H NMR spectrum of ethyl 4,11-dioxo-4*H*,11*H*-furo[2',3':4,5]pyrano[3,2-*c*]chromene-1-carboxylate (**6d**) in DMSO-d_6

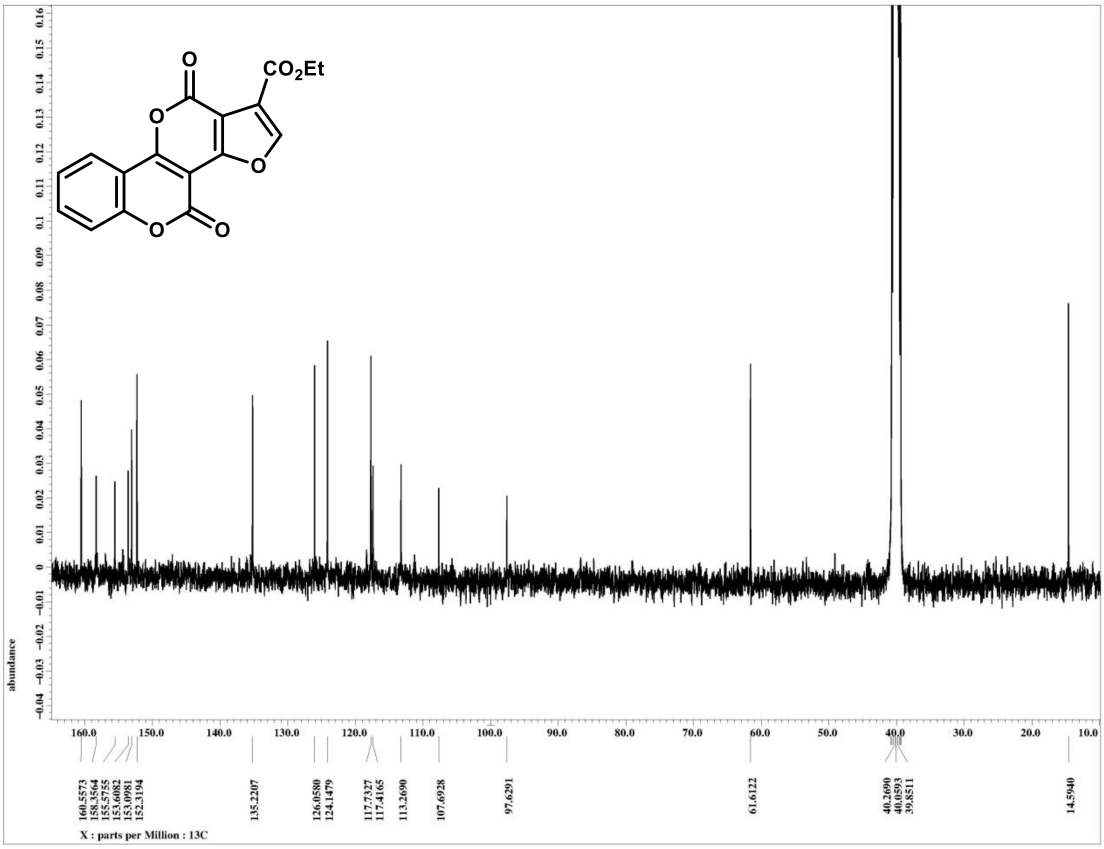

Figure S78. $^{13}C\{^{1}H\}$ NMR spectrum of ethyl 4,11-dioxo-4*H*,11*H*-furo[2',3':4,5]pyrano[3,2-*c*]chromene-1-carboxylate (**6d**) in DMSO-d_6

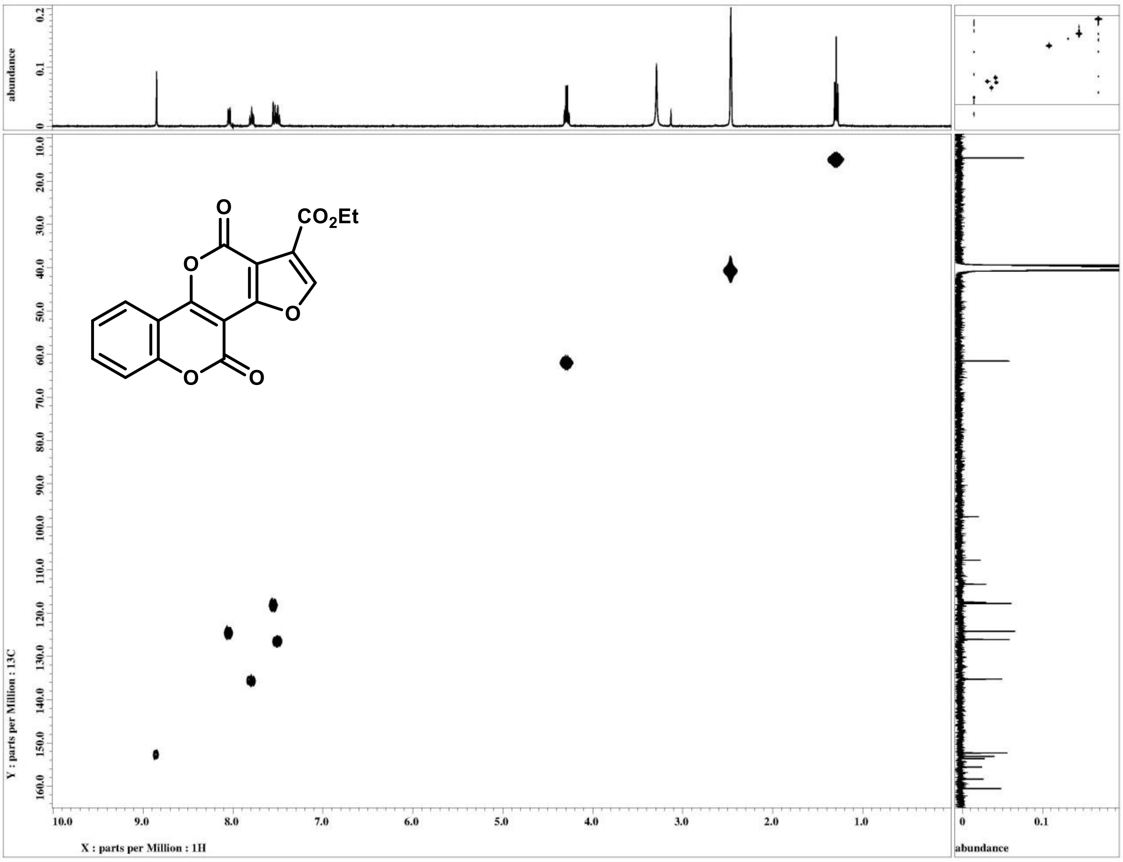

Figure S79. ^{1}H-^{13}C HMQC spectrum of ethyl 4,11-dioxo-4*H*,11*H*-furo[2',3':4,5]pyrano[3,2-*c*]chromene-1-carboxylate (**6d**) in DMSO-d_6

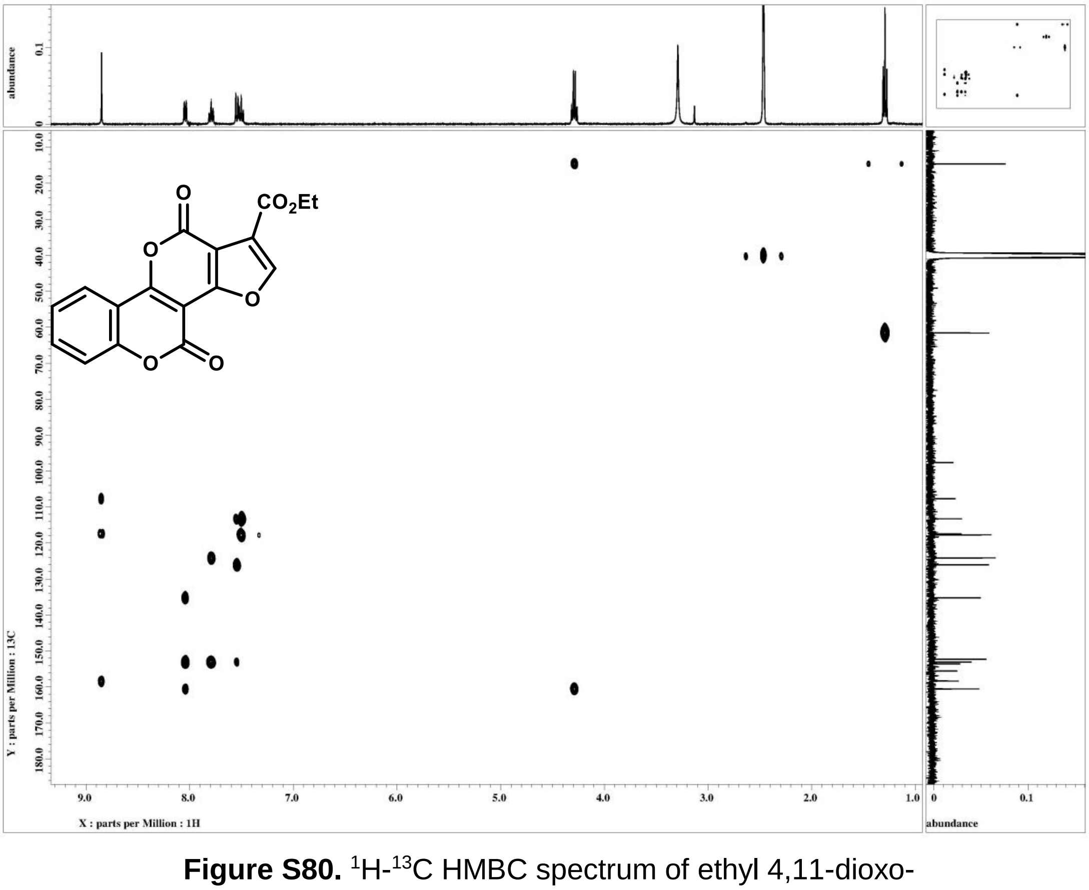

Figure S80. ^{1}H-^{13}C HMBC spectrum of ethyl 4,11-dioxo-4*H*,11*H*-furo[2',3':4,5]pyrano[3,2-*c*]chromene-1-carboxylate (**6d**) in DMSO-d_6

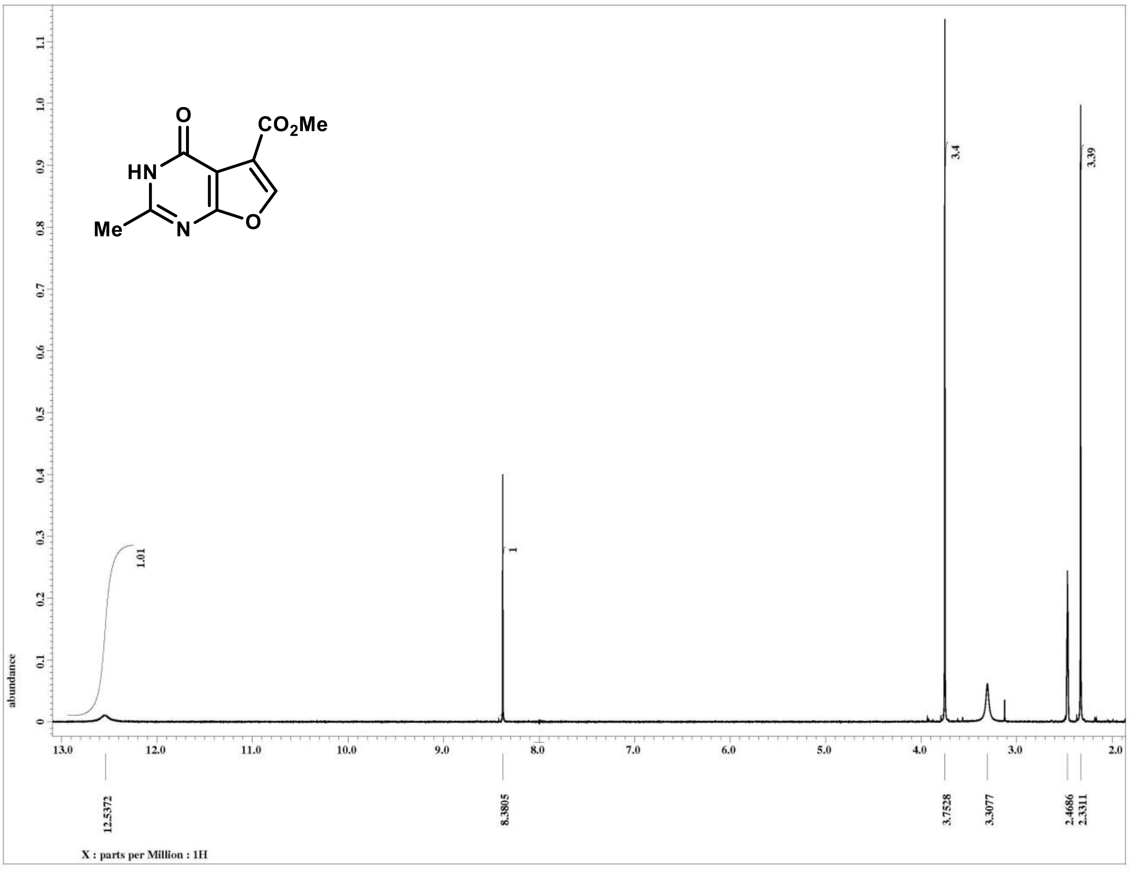

Figure S81. ^{1}H NMR spectrum of methyl 2-methyl-4-oxo-3,4-dihydrofuro[2,3-*d*]pyrimidine-5-carboxylate (**7a**) in DMSO-d_6

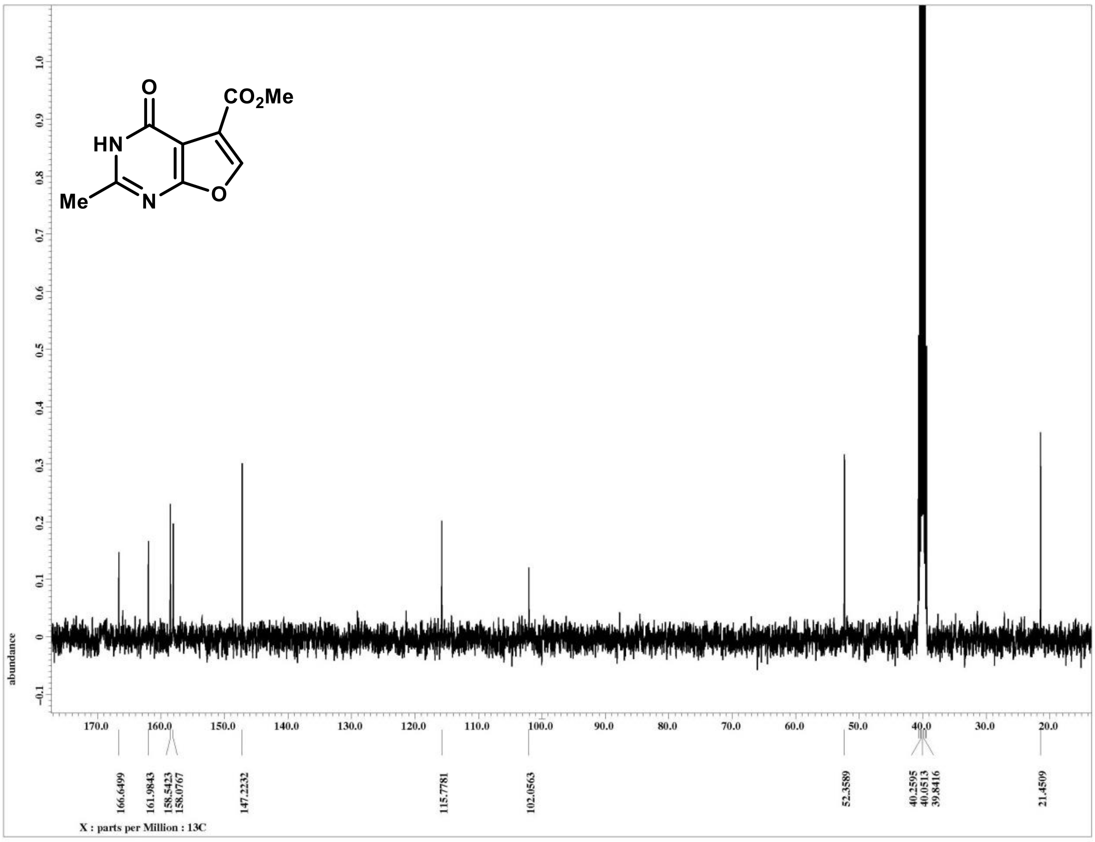

Figure S82. $^{13}C\{^{1}H\}$ NMR spectrum of methyl 2-methyl-4-oxo-3,4-dihydrofuro[2,3-*d*]pyrimidine-5-carboxylate (**7a**) in DMSO-d_6

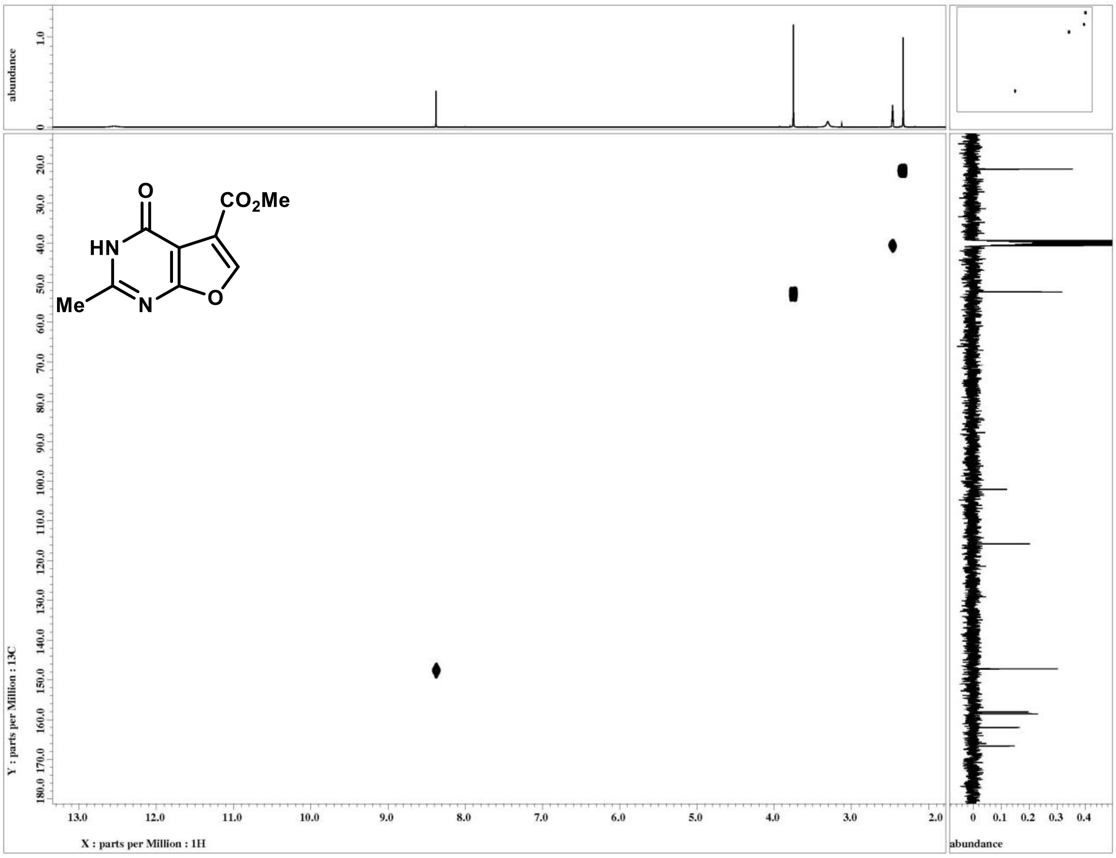

Figure S83. ^{1}H-^{13}C HMQC spectrum of methyl 2-methyl-4-oxo-3,4-dihydrofuro[2,3-*d*]pyrimidine-5-carboxylate (**7a**) in DMSO-d_6

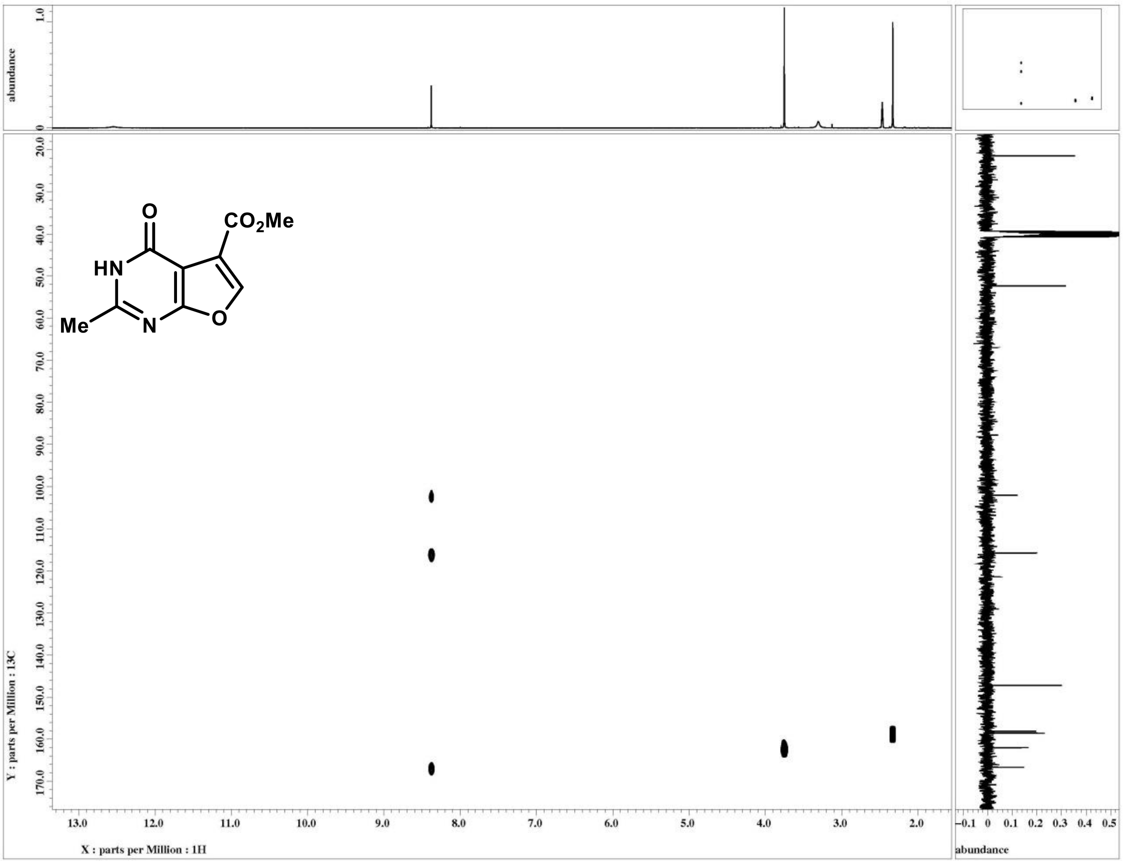

Figure S84. ^{1}H-^{13}C HMBC spectrum of methyl 2-methyl-4-oxo-3,4-dihydrofuro[2,3-*d*]pyrimidine-5-carboxylate (**7a**) in DMSO-d_6

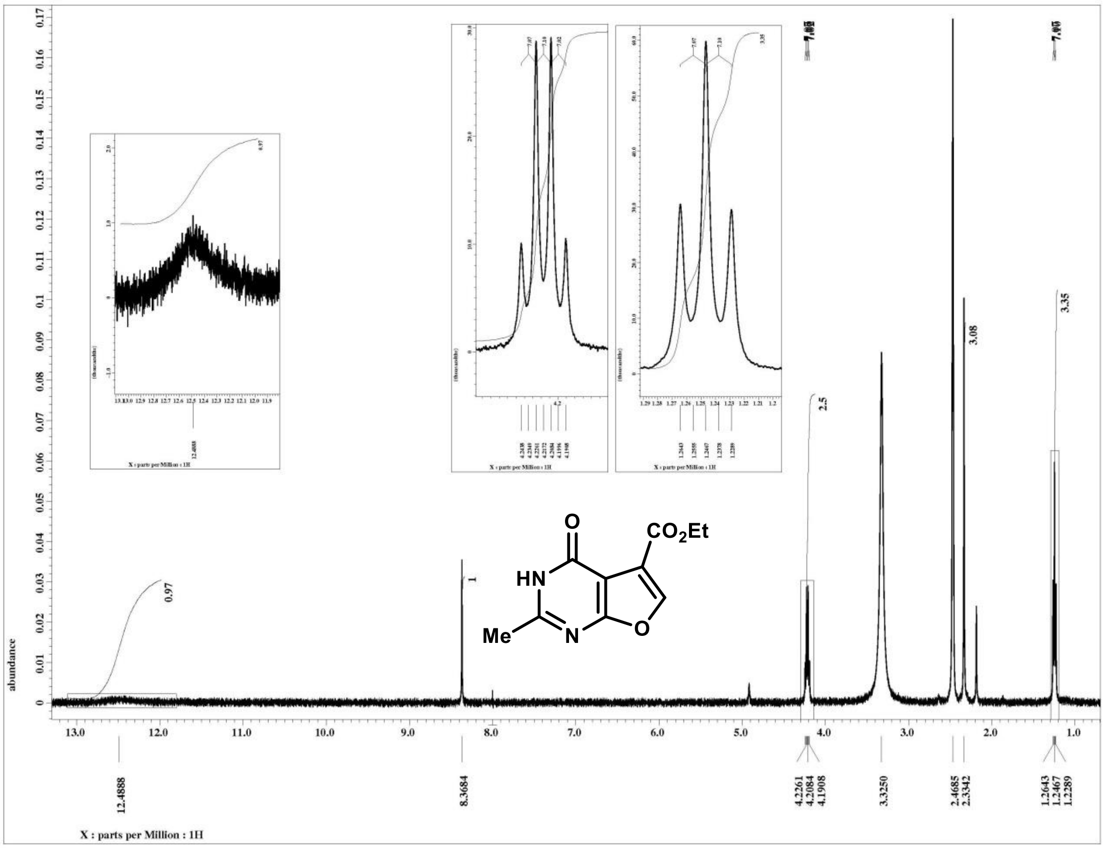

Figure S85. ^{1}H NMR spectrum of ethyl 2-methyl-4-oxo-3,4-dihydrofuro[2,3-*d*]pyrimidine-5-carboxylate (**7b**) in DMSO-d_6

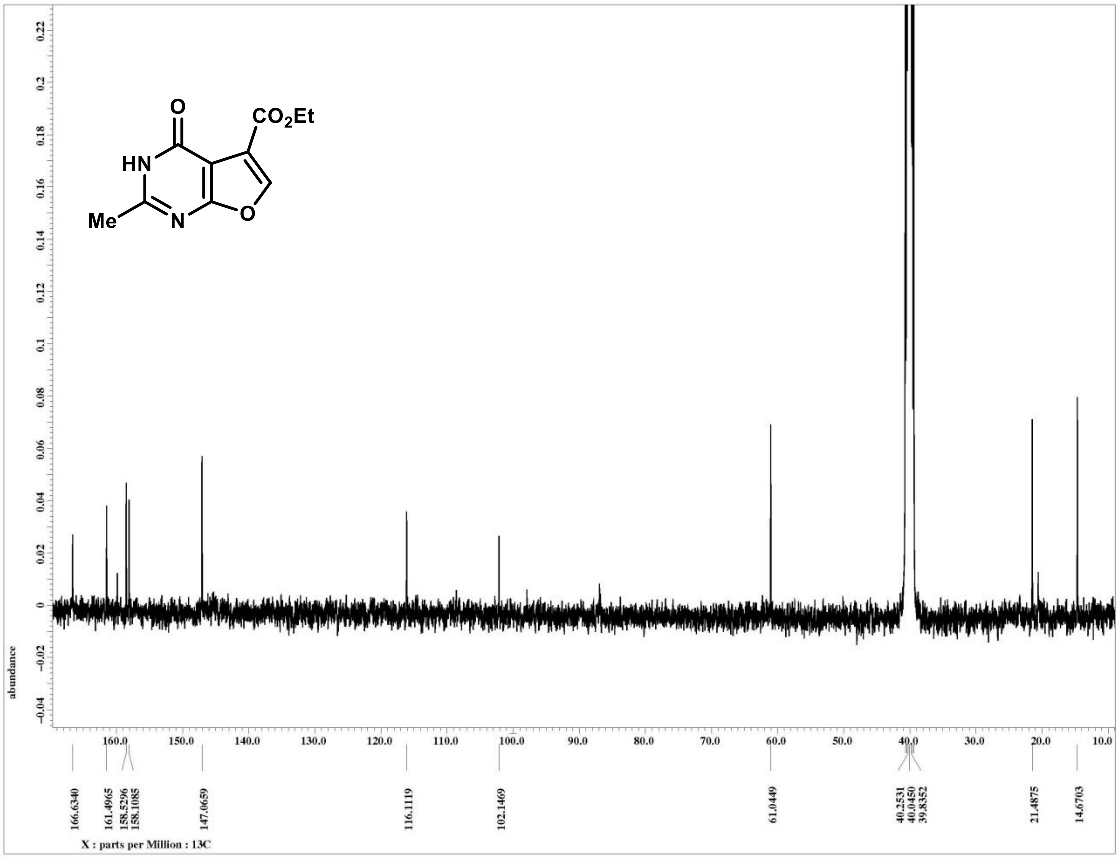

Figure S86. ^{13}C{^{1}H} NMR spectrum of ethyl 2-methyl-4-oxo-3,4-dihydrofuro[2,3-*d*]pyrimidine-5-carboxylate (**7b**) in DMSO-d_6

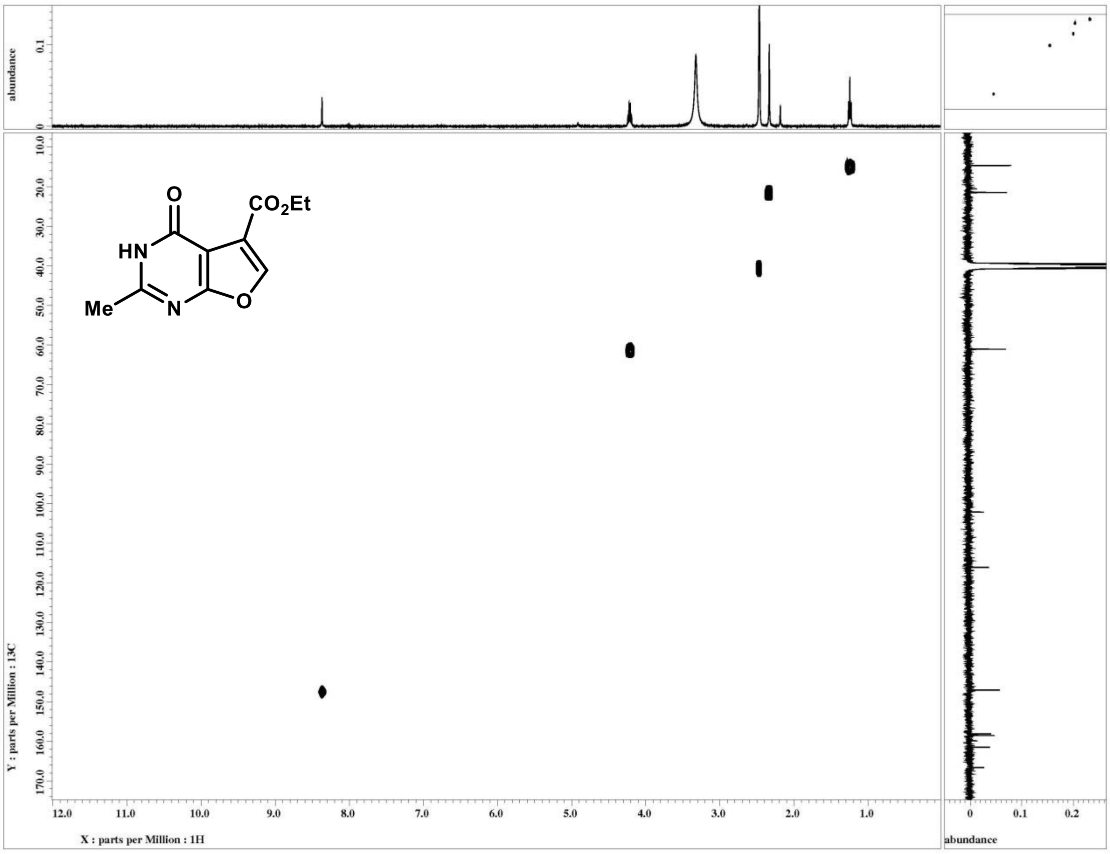

Figure S87. ^{1}H-^{13}C HMQC spectrum of ethyl 2-methyl-4-oxo-3,4-dihydrofuro[2,3-*d*]pyrimidine-5-carboxylate (**7b**) in DMSO-d_6

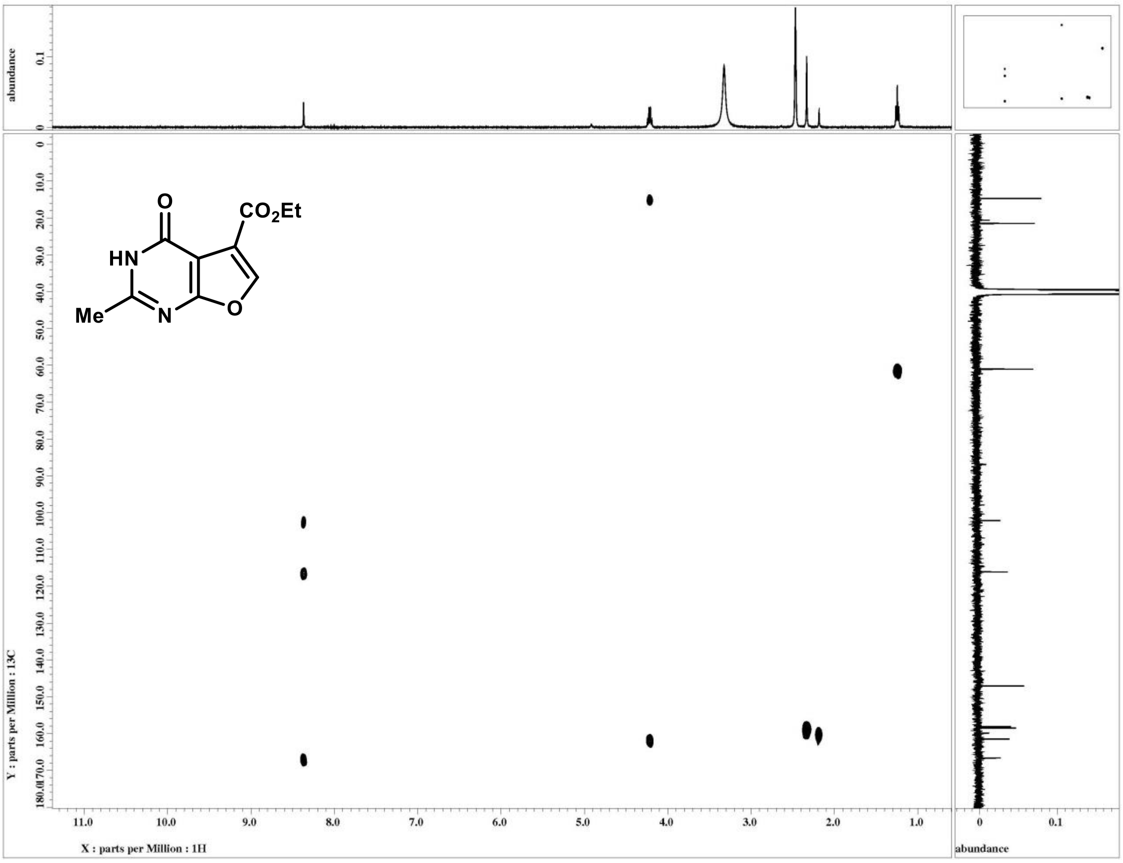

Figure S88. ^{1}H-^{13}C HMBC spectrum of ethyl 2-methyl-4-oxo-3,4-dihydrofuro[2,3-*d*]pyrimidine-5-carboxylate (**7b**) in DMSO-d_6

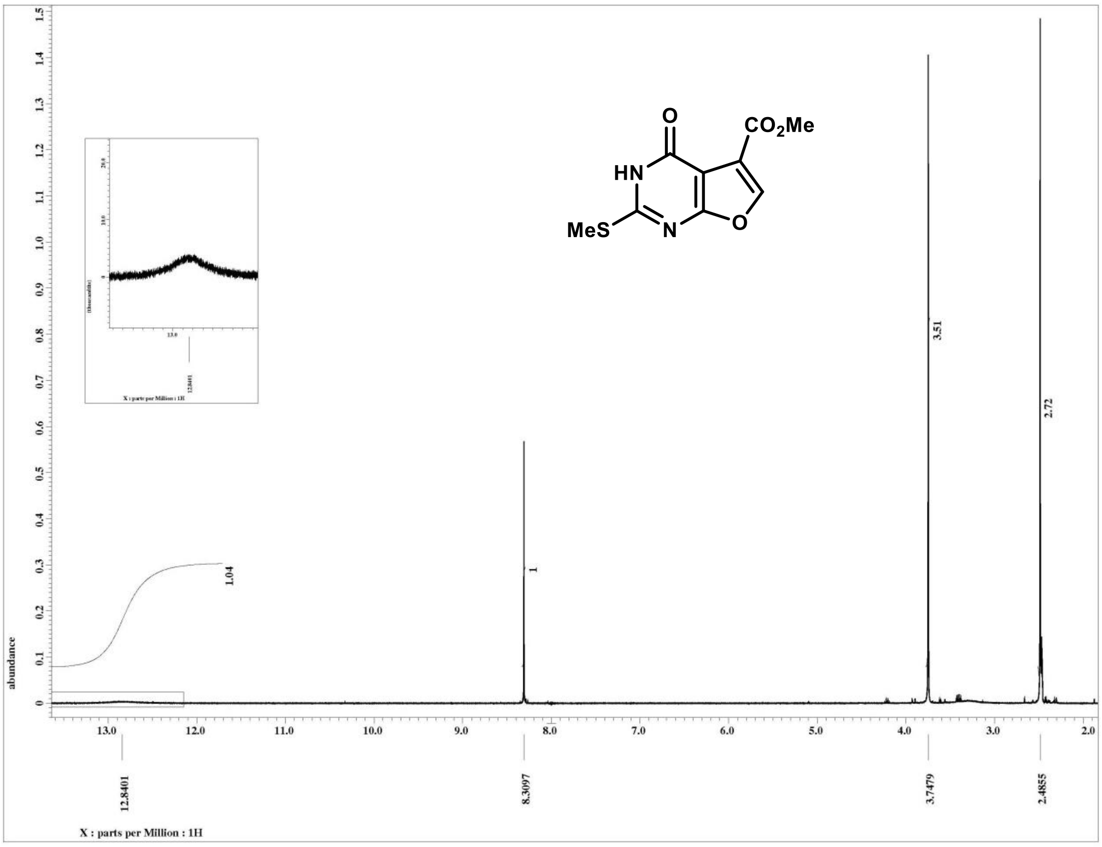

Figure S89. ^{1}H NMR spectrum of methyl 2-(methylsulfanyl)-4-oxo-3,4-dihydrofuro[2,3-*d*]pyrimidine-5-carboxylate (**7c**) in DMSO-d_6

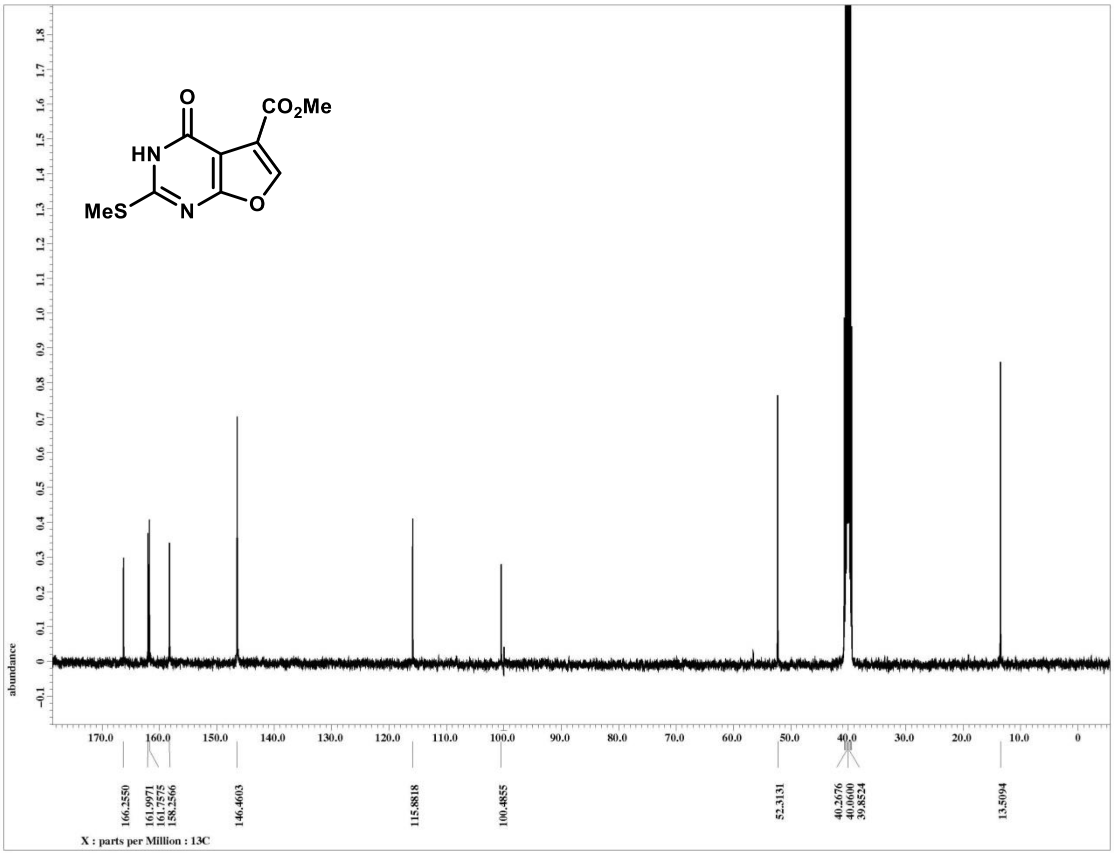

Figure S90. $^{13}C\{^{1}H\}$ NMR spectrum of methyl 2-(methylsulfanyl)-4-oxo-3,4-dihydrofuro[2,3-*d*]pyrimidine-5-carboxylate (**7c**) in DMSO-d_6

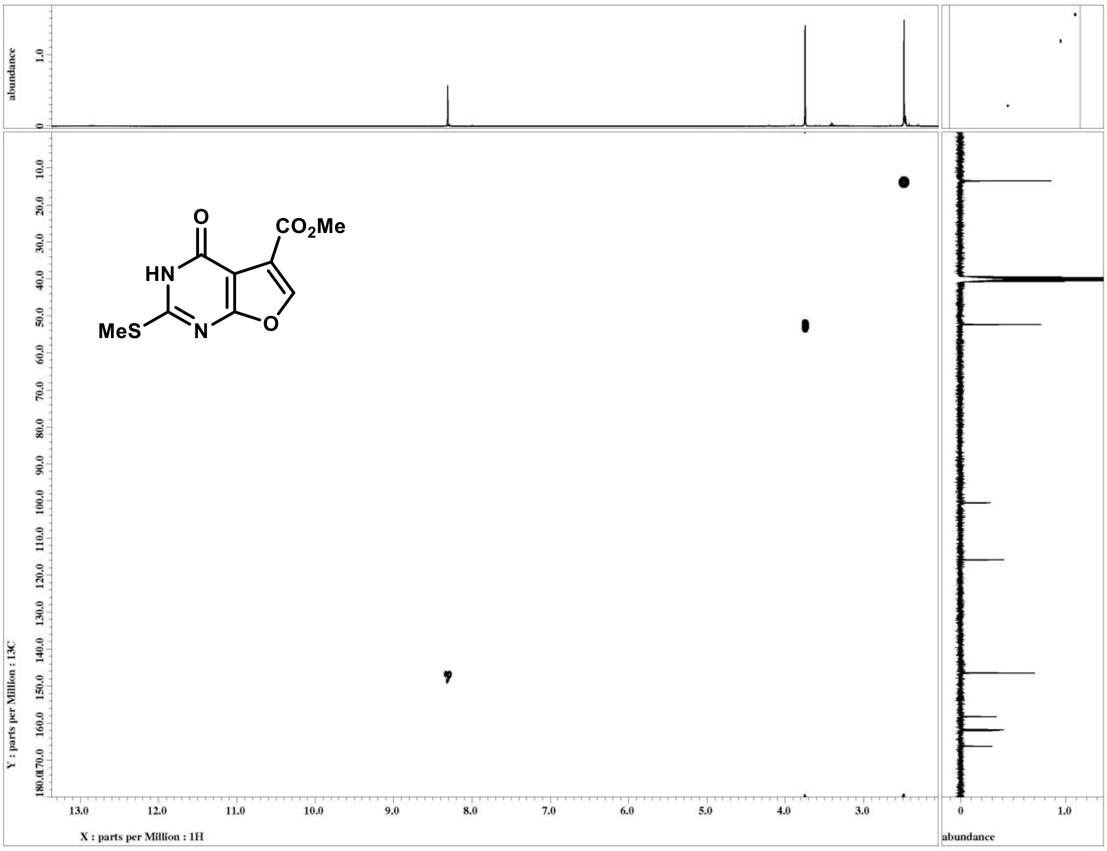

Figure S91. ^{1}H-^{13}C HMQC spectrum of methyl 2-(methylsulfanyl)-4-oxo-3,4-dihydrofuro[2,3-*d*]pyrimidine-5-carboxylate (**7c**) in DMSO-d_6

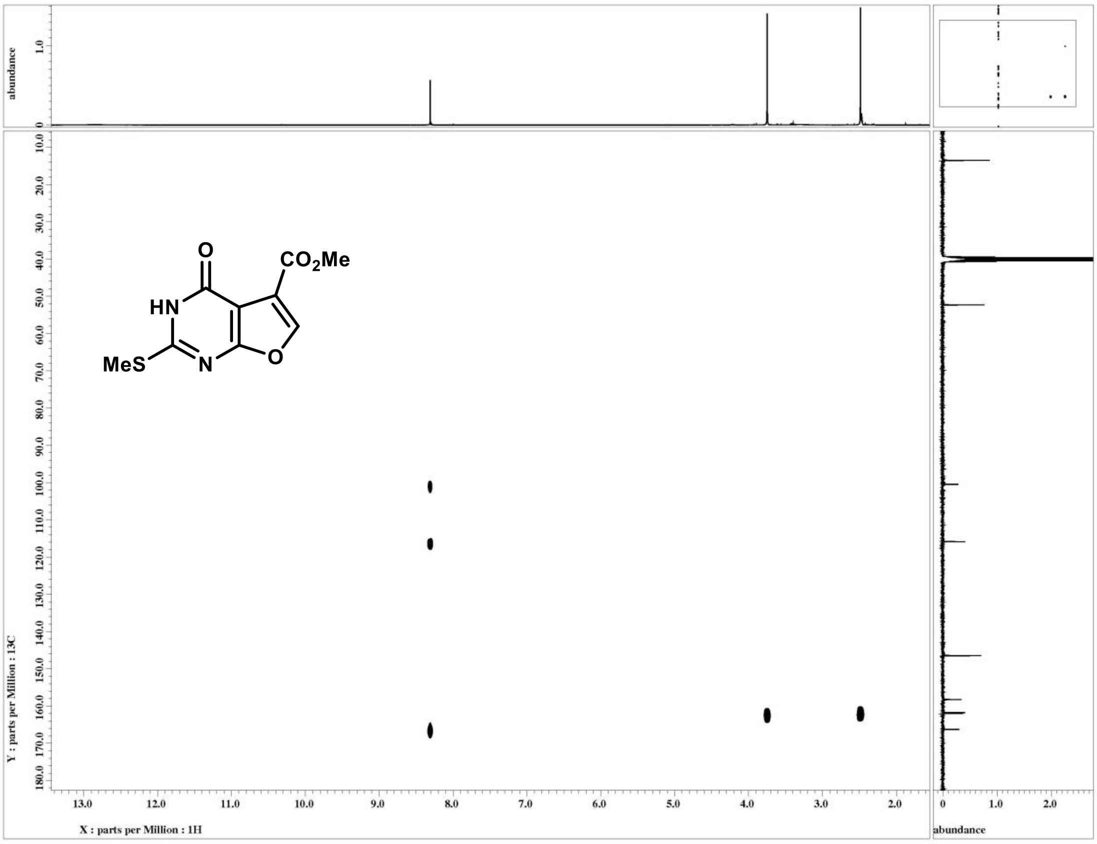

Figure S92. ^{1}H-^{13}C HMBC spectrum of methyl 2-(methylsulfanyl)-4-oxo-3,4-dihydrofuro[2,3-*d*]pyrimidine-5-carboxylate (**7c**) in DMSO-d_6

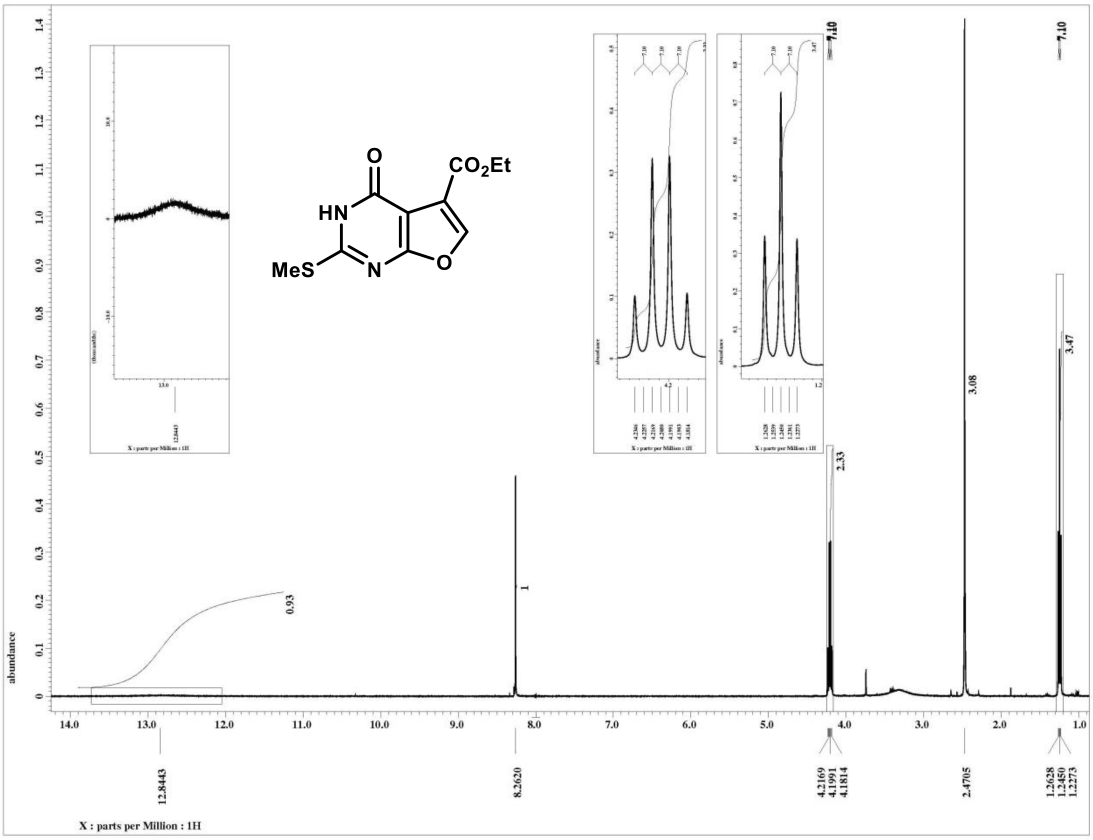

Figure S93. ^{1}H NMR spectrum of ethyl 2-(methylsulfanyl)-4-oxo-3,4-dihydrofuro[2,3-*d*]pyrimidine-5-carboxylate (**7d**) in DMSO-d_6

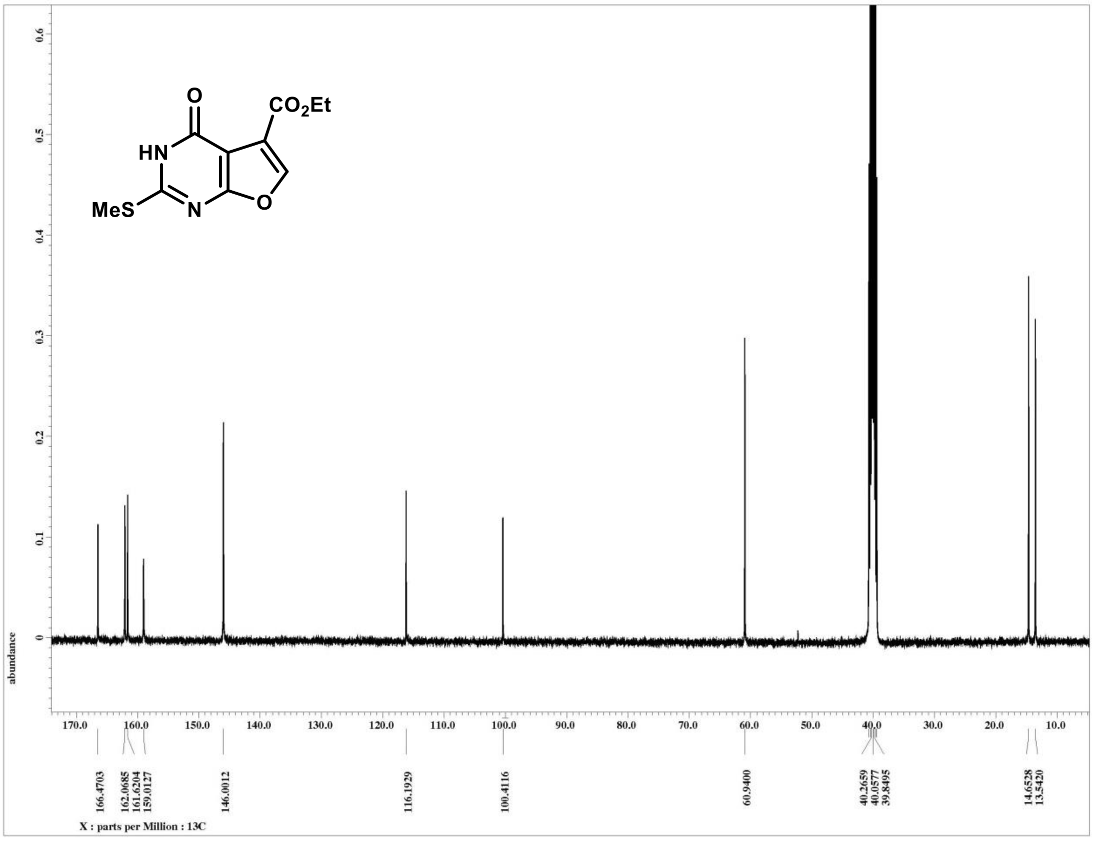

Figure S94. $^{13}C\{^{1}H\}$ NMR spectrum of ethyl 2-(methylsulfanyl)-4-oxo-3,4-dihydrofuro[2,3-*d*]pyrimidine-5-carboxylate (**7d**) in DMSO-d_6

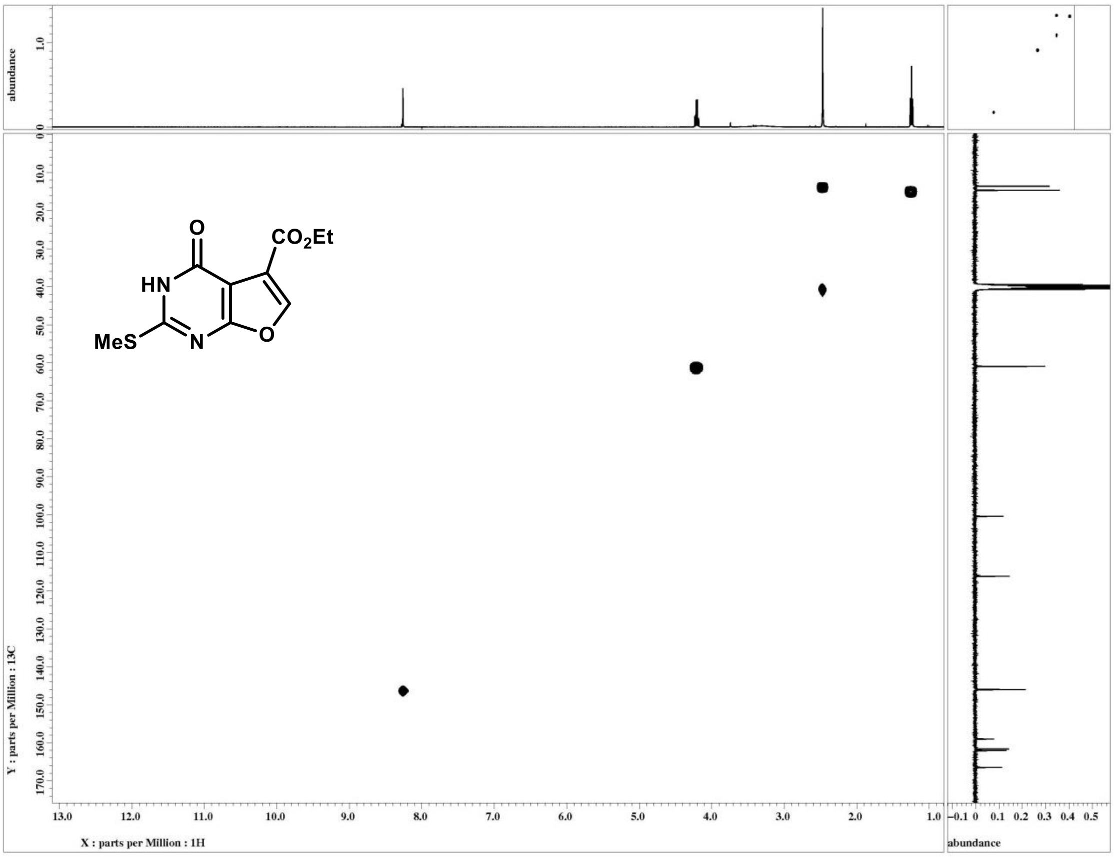

Figure S95. ^1H-^{13}C HMQC spectrum of ethyl 2-(methylsulfanyl)-4-oxo-3,4-dihydrofuro[2,3-*d*]pyrimidine-5-carboxylate (**7d**) in DMSO-d_6

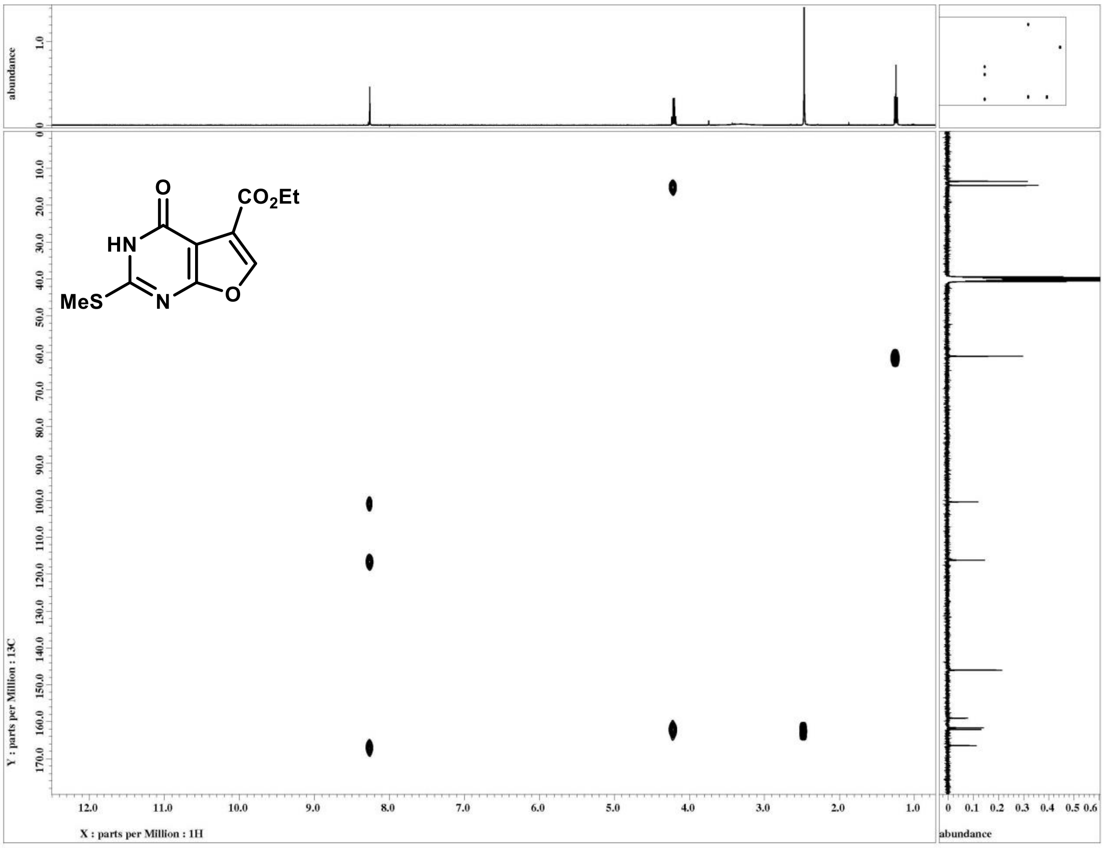

Figure S96. ^1H-^{13}C HMBC spectrum of ethyl 2-(methylsulfanyl)-4-oxo-3,4-dihydrofuro[2,3-*d*]pyrimidine-5-carboxylate (**7d**) in DMSO-d_6

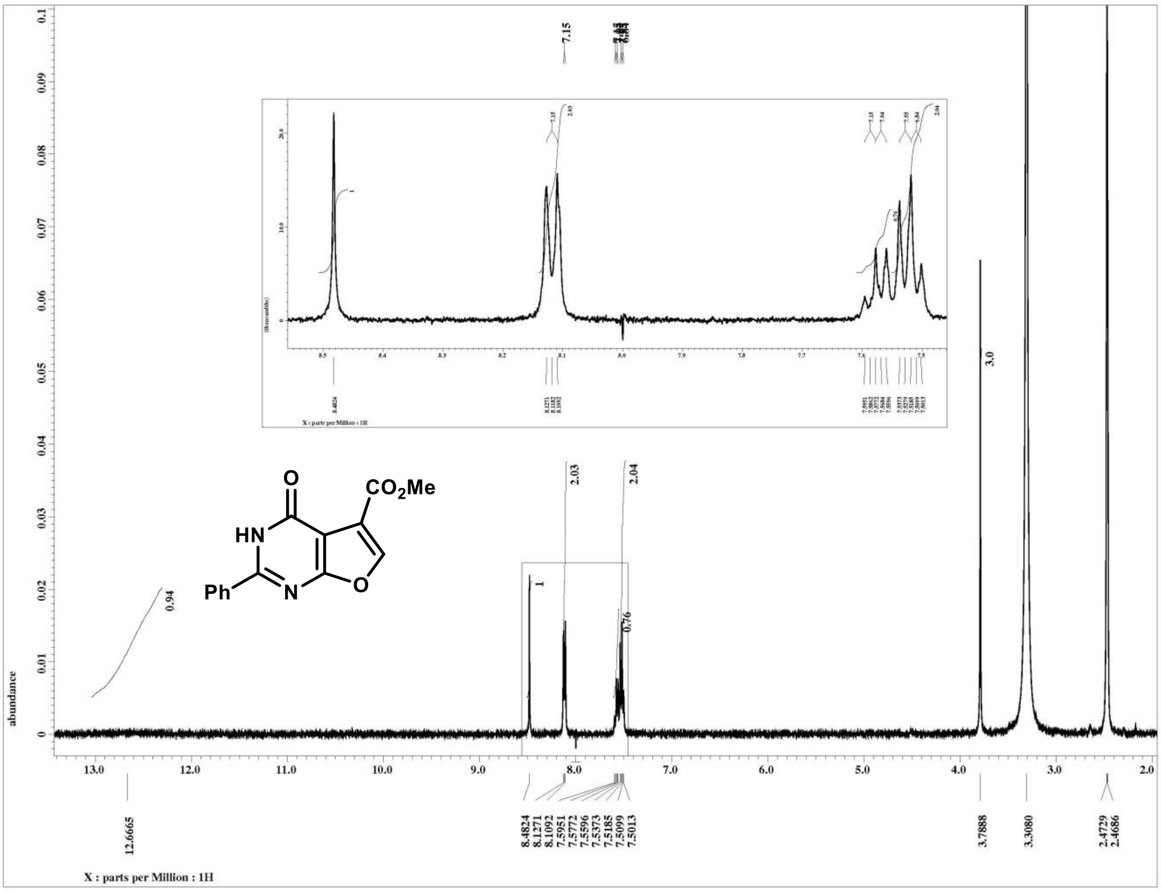

Figure S97. ^{1}H NMR spectrum of methyl 4-oxo-2-phenyl-3,4-dihydrofuro[2,3-*d*]pyrimidine-5-carboxylate (**7e**) in DMSO-d_6

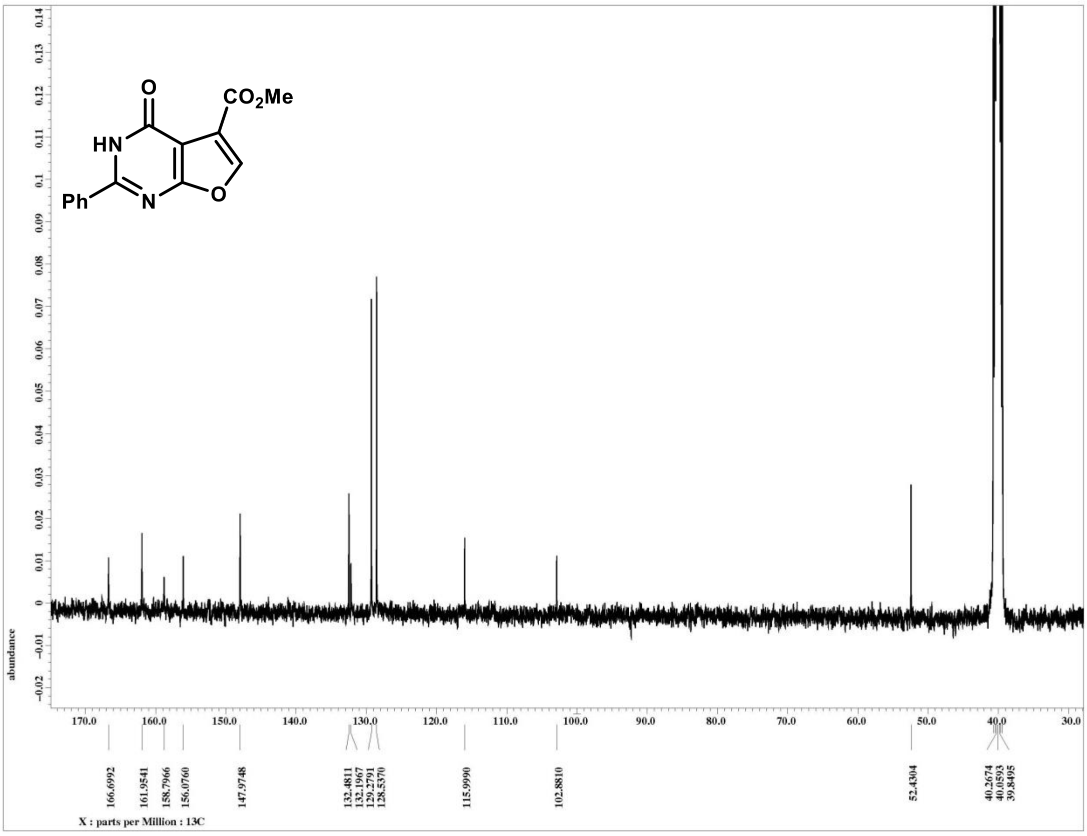

Figure S98. ^{13}C{^{1}H} NMR spectrum of methyl 4-oxo-2-phenyl-3,4-dihydrofuro[2,3-*d*]pyrimidine-5-carboxylate (**7e**) in DMSO-d_6

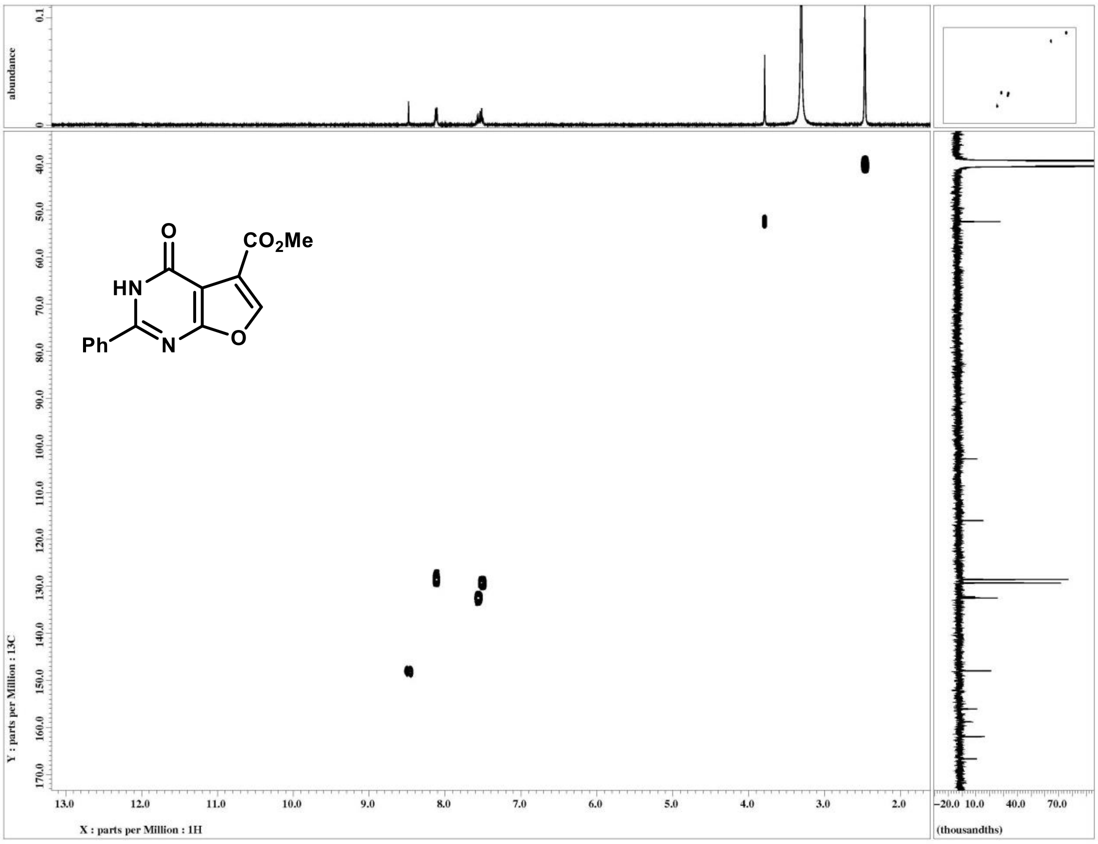

Figure S99. ^{1}H-^{13}C HMQC spectrum of methyl 4-oxo-2-phenyl-3,4-dihydrofuro[2,3-*d*]pyrimidine-5-carboxylate (**7e**) in DMSO-d_6

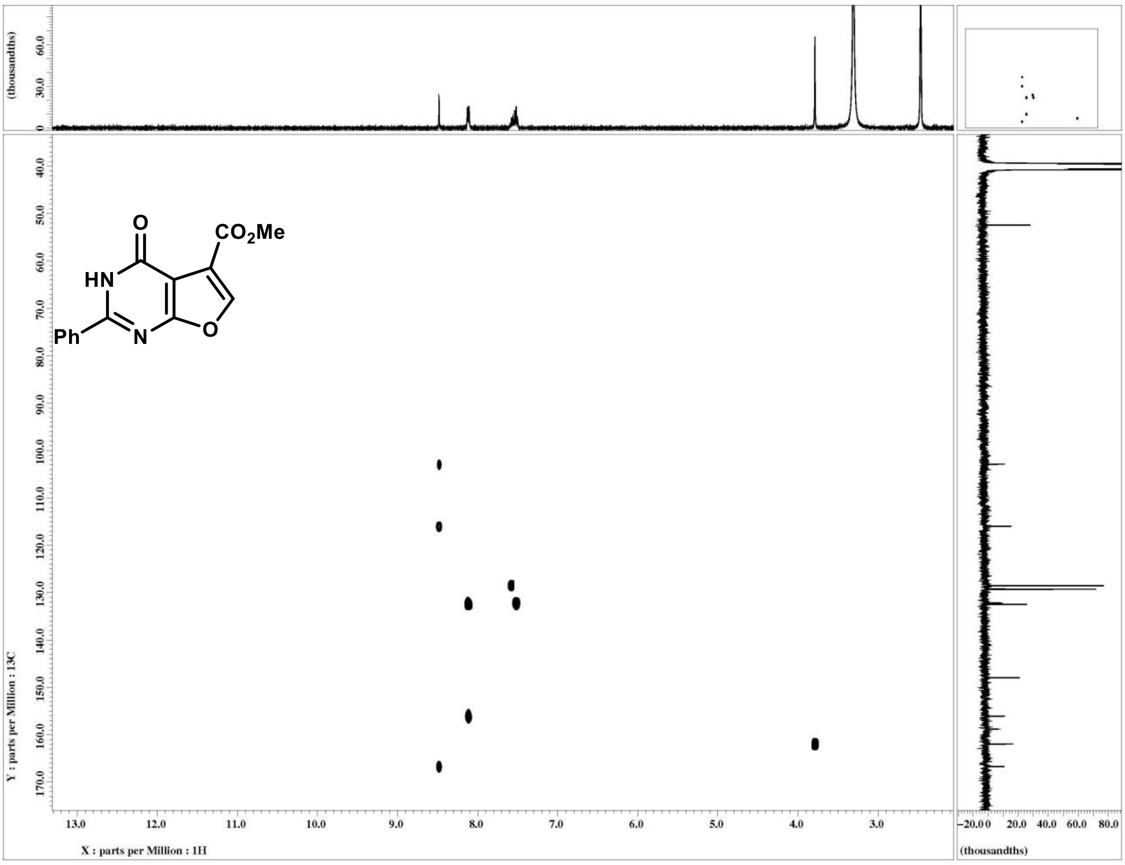

Figure S100. ^{1}H-^{13}C HMBC spectrum of methyl 4-oxo-2-phenyl-3,4-dihydrofuro[2,3-*d*]pyrimidine-5-carboxylate (**7e**) in DMSO-d_6

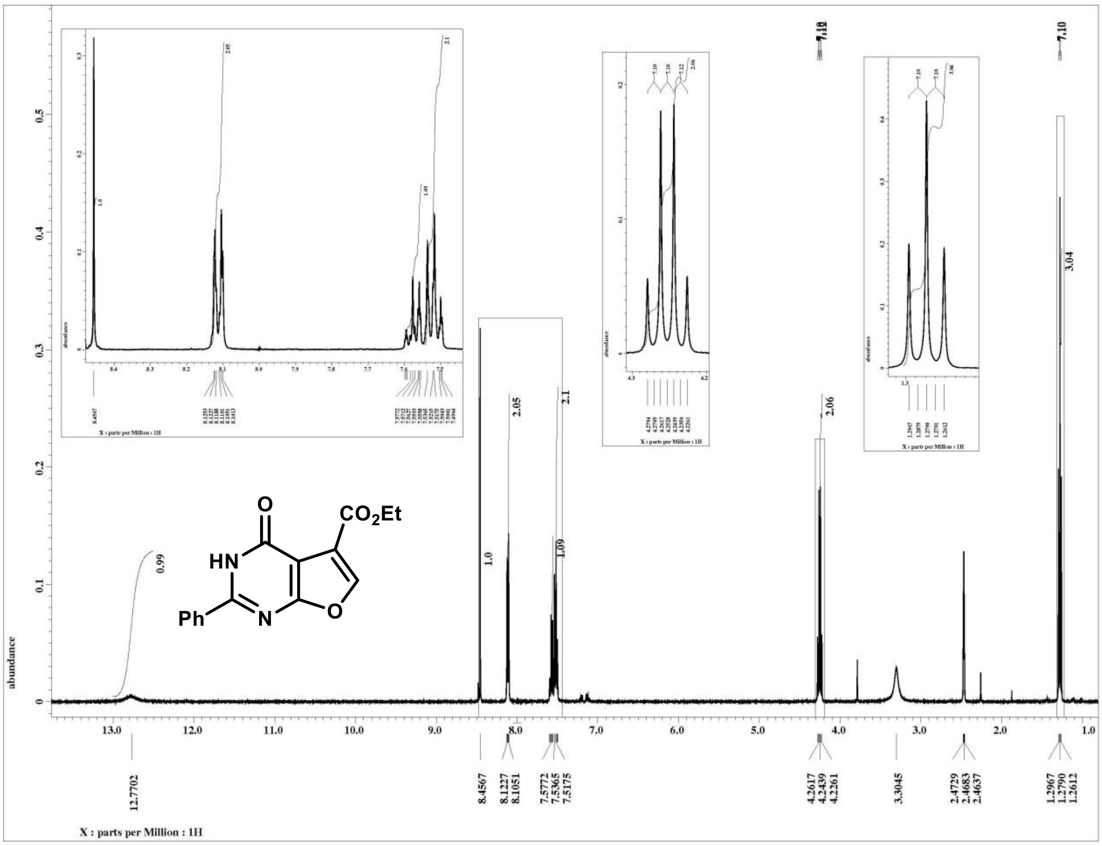

Figure S101. ^{1}H NMR spectrum of ethyl 4-oxo-2-phenyl-3,4-dihydrofuro[2,3-*d*]pyrimidine-5-carboxylate (**7f**) in DMSO-d_6

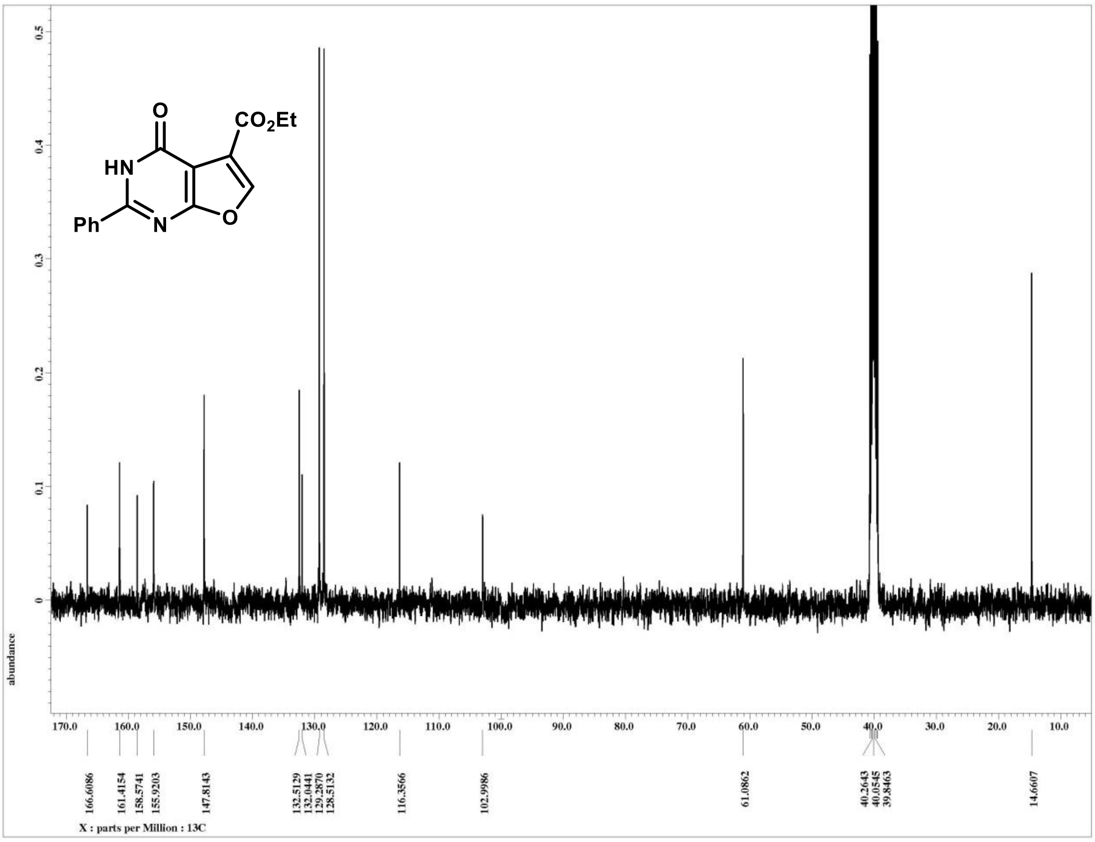

Figure S102. $^{13}C\{^{1}H\}$ NMR spectrum of ethyl 4-oxo-2-phenyl-3,4-dihydrofuro[2,3-*d*]pyrimidine-5-carboxylate (**7f**) in DMSO-d_6

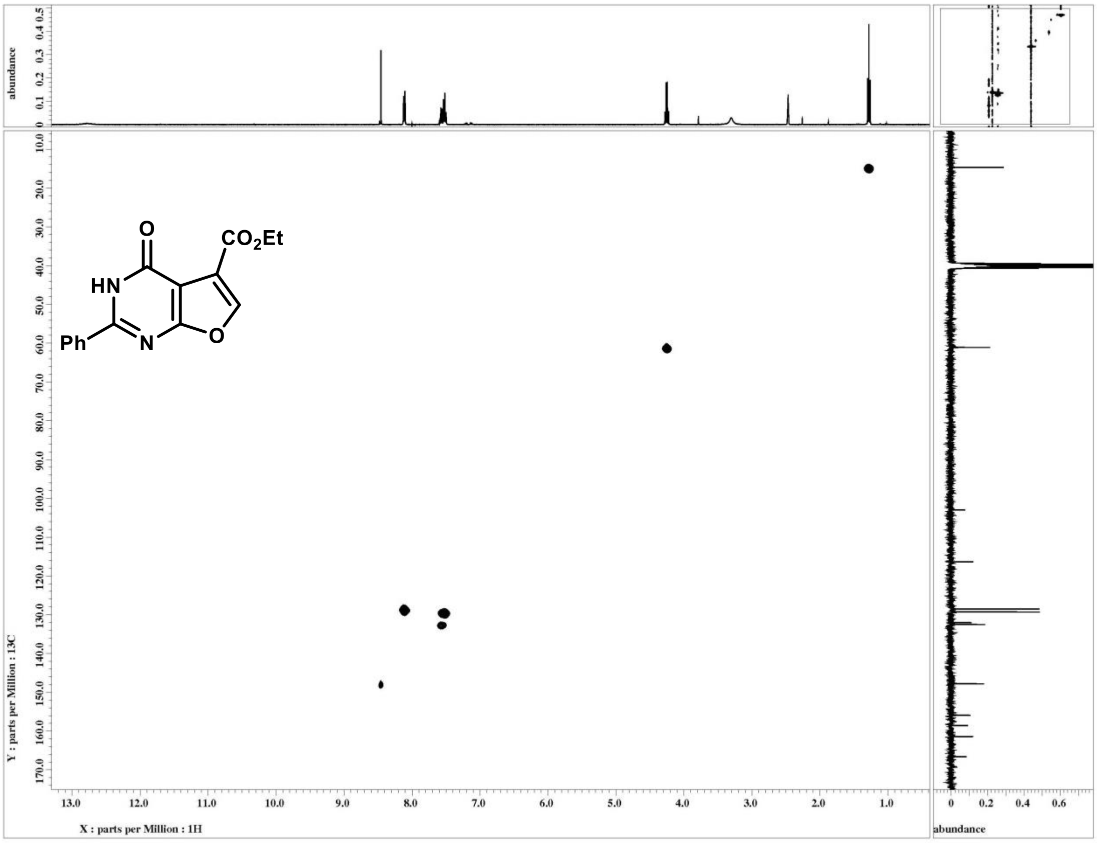

Figure S103. ^{1}H-^{13}C HMQC spectrum of ethyl 4-oxo-2-phenyl-3,4-dihydrofuro[2,3-*d*]pyrimidine-5-carboxylate (**7f**) in DMSO-d_6

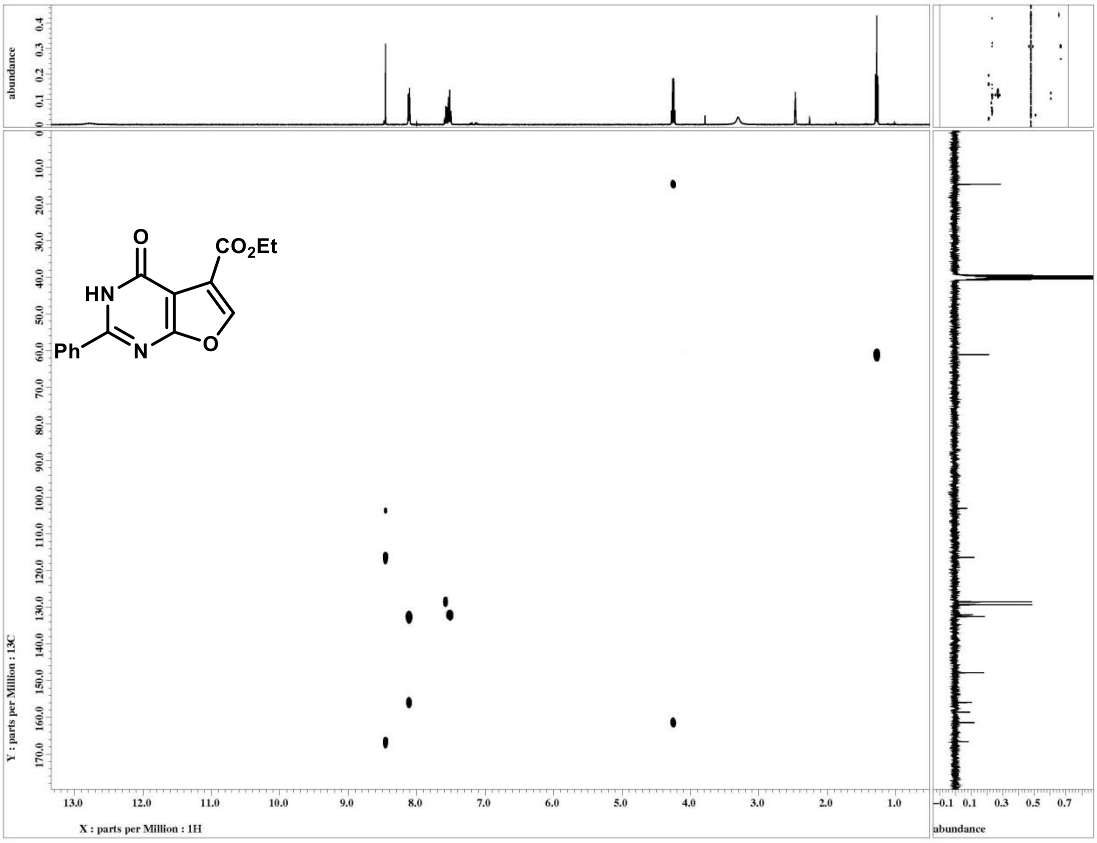

Figure S104. ^{1}H-^{13}C HMBC spectrum of ethyl 4-oxo-2-phenyl-3,4-dihydrofuro[2,3-*d*]pyrimidine-5-carboxylate (**7f**) in DMSO-d_6

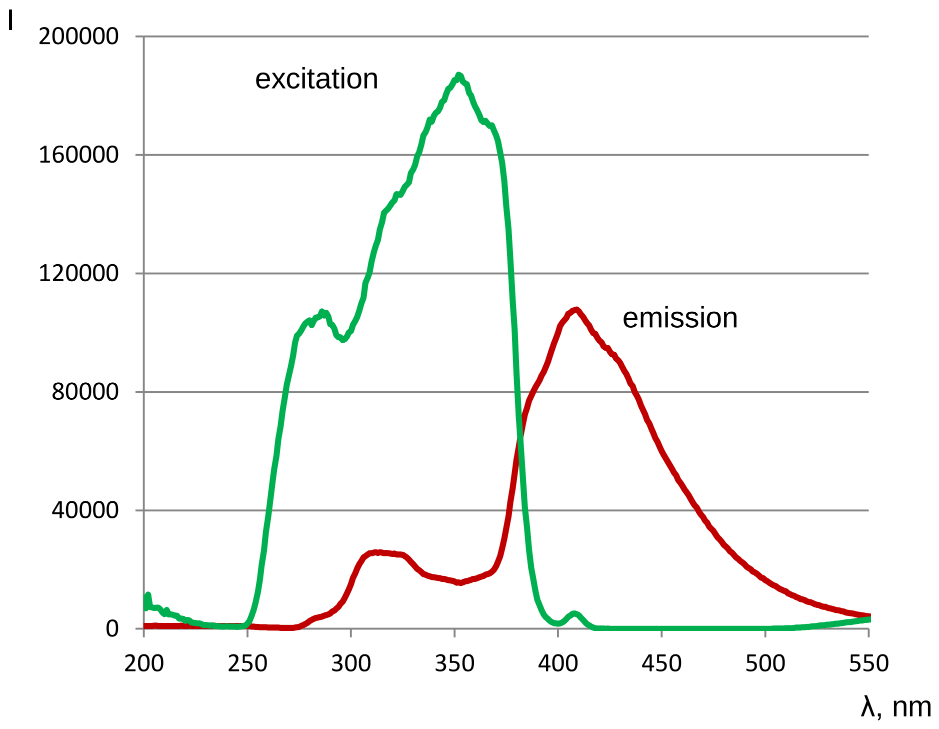

Figure S105. Luminescence excitation spectrum of compound **6c** in DMSO solution (*C*m = 5.23 · 10^{-5} mol/L)

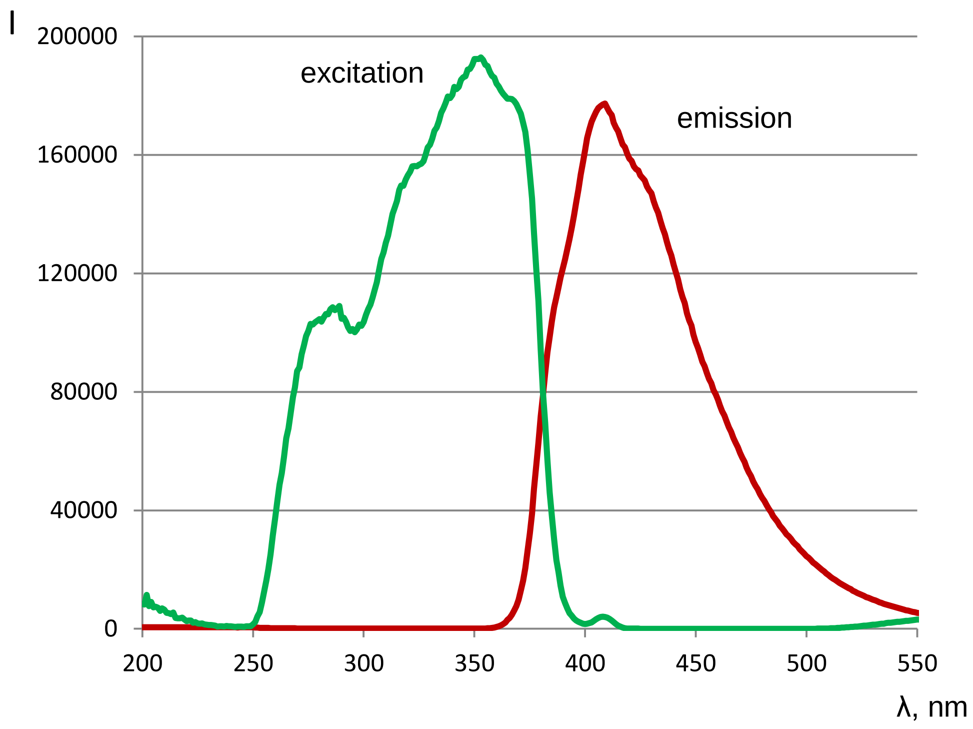

Figure S106. Luminescence excitation spectrum of compound **6d** in DMSO solution (*C*m = 5.46 · 10^{-5} mol/L)

Table S1. Principal crystallographic parameters of compound **5a**, **5b**, **6b**, **6c**, **and 7a** based on X-ray diffraction data

	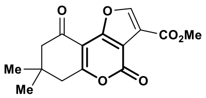	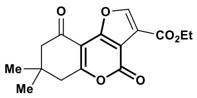	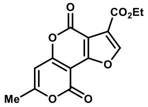	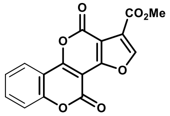	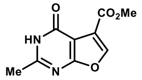
Parameter	**5a**	**5b**	**6b**	**6c**	**7a**
Molecular formula	$C_{15}H_{14}O_6$	$C_{16}H_{16}O_6$	$C_{14}H_{10}O_7$	$C_{16}H_8O_7$	$C_9H_8N_2O_4$
Molecular weight	290.26	304.29	290.22	312.22	208.17
Crystal system	monoclinic	triclinic	triclinic	monoclinic	triclinic
Space group	$P2_1/c$ (14)	P-1 (2)	P-1 (2)	Pc (7)	P-1 (2)
Z	4	6 (3 independent molecules)	2	4 (two independent molecules)	4 (two independent molecules)
Unit cell parameters					
a/Å	6.6178(6)	11.2612(5)	7.4218(7)	5.3339(7)	9.0785(11)
b/Å	7.5386(6)	14.1141(6)	7.6463(7)	11.6680(16)	9.2773(11)
c/Å	26.181(2)	14.7523(7)	11.2353(11)	20.098(3)	10.6926(13)
α/deg	90	98.448(2)	82.512(4)	90	78.850(4)
β/deg	95.796(4)	91.895(2)	88.594(4)	93.622(5)	81.164(4)
γ/deg	90	112.426(2)	69.106(3)	90	85.869(4)
$V/Å^3$	1299.47(19)	2133.70(17)	590.42(10)	1248.3(3)	872.26(18)
$d_{calc}/g/cm^3$	1.484	1.421	1.632	1.661	1.585
Absorption coefficient, μ/mm^{-1}	0.116	0.109	0.134	0.133	0.127
F(000)	608.0	960.0	300	640	432.0
2Θ range for data collection /deg	5.626 to 55.082	2.804 to 64.0	5.752 to 64.0	3.492 to 58.2	4.48 to 63.994

Ranges of indices					
h	-8 ≤ *h* ≤ 8	-16 ≤ *h* ≤ 16	-11 ≤ *h* ≤ 11	-7 ≤ *h* ≤ 7	-13 ≤ *h* ≤ 13
k	-9 ≤ *k* ≤ 9	-21 ≤ *k* ≤ 21	-11 ≤ *k* ≤ 11	-15 ≤ *k* ≤ 15	-13 ≤ *k* ≤ 13
l	-34 ≤ *l* ≤ 34	-21 ≤ *l* ≤ 21	-16 ≤ *l* ≤ 16	-27 ≤ *l* ≤ 27	-15 ≤ *l* ≤ 15
Number of reflections					
total	48605	93134	25013	30457	79507
independent	2981	14780	4078	6673	6043
R_{int}	0.1129	0.0734	0.0545	0.085	0.0640
Observed [$I > 2\sigma(I)$]	2354	11195	3251	4916	5001
Number of reflections/of contraints/number of parameters	2981/0/193	14780/0/604	4078/0/192	6673/38/411	6043/0/278
GOOF	1.039	1.034	1.013	1.014	1.017
R [$I > 2\sigma(I)$]					
R_1	0.0372	0.0448	0.0422	0.1006	0.0423
wR_2	0.0987	0.1153	0.1136	0.2433	0.1164
R (based on all reflections)					
R_1	0.0500	0.0641	0.0550	0.1277	0.0508
wR_2	0.1034	0.1245	0.1182	0.2643	0.1224
Residual electron density (ρ_{max}/ρ_{min})/e Å^{-3}	0.29/-0.29	0.50/-0.39	0.55/-0.29	1.68/-0.47	0.55/-0.53
CCDC	2403284	2403285	2403316	2403286	2403287

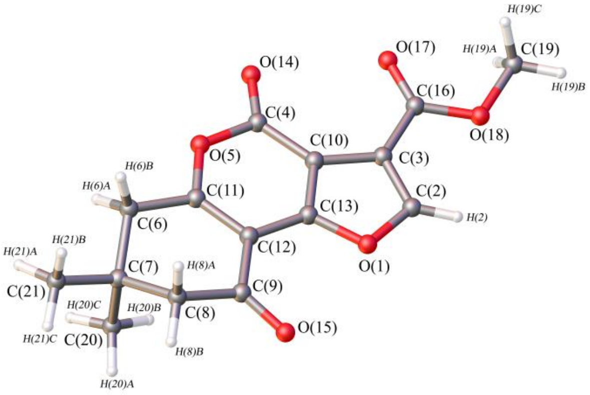

Figure S107. Perspective views of methyl 7,7-dimethyl-4,9-dioxo-6,7,8,9-tetrahydro-4*H*-furo[3,2-*c*][1]benzopyran-3-carboxylate (**5a**) (X-ray data).

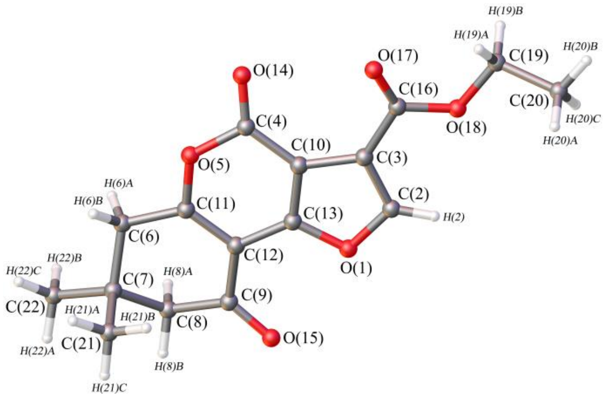

Figure S108. Perspective views of ethyl 7,7-dimethyl-4,9-dioxo-6,7,8,9-tetrahydro-4*H*-furo[3,2-*c*][1]benzopyran-3-carboxylate (**5b**) (X-ray data).

Table S2. Torsion angles (τ) in the molecule of compounds **5a** and **5b**

Angle	τ/deg	
	**5a**	**5b**
	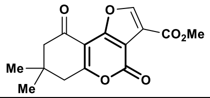	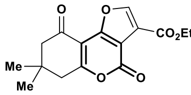
H2–C2–C3–C10	-179.8	-179.7
H2–C2–C3–C16	-1.7	4.1
O1–C2–C3–C10	0.2(1)	0.3(1)
O1–C2–C3–C16	178.4(1)	-175.87(9)
H2–C2–O1–C13	179.8	179.0
C3–C2–O1–C13	-0.2(1)	-1.0(1)
C2–C3–C10–C4	179.6(1)	177.2(1)
C2–C3–C10–C13	-0.2(1)	0.5(1)
C16–C3–C10–C4	1.5(2)	-6.9(2)
C16–C3–C10–C13	-178.3(1)	176.3(1)
C2–C3–C16–O17	-178.5(1)	159.6(1)
C2–C3–C16–O18	0.3(2)	-18.2(1)
C10–C3–C16–O17	-0.8(2)	-15.6(2)
C10–C3–C16–O18	178.1(1)	166.58(9)
O5–C4–C10–C3	-177.0(1)	-174.6(1)
O5–C4–C10–C13	2.8(2)	1.8(1)
O14–C4–C10–C3	2.2(3)	4.5(2)
O14–C4–C10–C13	-178.0(1)	-179.1(1)
C10–C4–O5–C11	-2.7(2)	2.9(1)
O14–C4–O5–C11	178.0(1)	-176.40(9)
H6A–C6–C7–C8	167.7	69.9
H6A–C6–C7–C20	46.7	–
H6A–C6–C7–C21	-73.7	-170.11
H6A–C6–C7–C22	–	-49.6
H6B–C6–C7–C8	-74.7	-172.08
H6B–C6–C7–C20	164.3	–
H6B–C6–C7–C21	43.9	-52.1
H6B–C6–C7–C22	–	68.4
C11–C6–C7–C8	46.4(1)	-51.1(1)
C11–C6–C7–C20	-74.6(1)	–
C11–C6–C7–C21	165.1(1)	68.9(1)

C11A–C6A–C7A–C22A	–	-170.65(9)
H6A–C6–C11–C12	-140.8	-97.8
H6A–C6–C11–O5	40.9	84.2
H6B–C6–C11–C12	101.5	144.2
H6B–C6–C11–O5	-76.7	-33.9
C7–C6–C11–C12	-19.6(2)	23.2(1)
C7–C6–C11–O5	162.1(1)	-154.85(8)
C6–C7–C8–H8A	62.9	-64.0
C6–C7–C8–H8B	179.9	178.75
C6–C7–C8–C9	-58.6(1)	57.4(1)
C20–C7–C8–H8A	-176.6	–
C20–C7–C8–H8B	-59.5	–
C20–C7–C8–C9	62.0(1)	–
C21–C7–C8–H8A	-55.8	175.37
C21–C7–C8–H8B	61.3	58.2
C21–C7–C8–C9	-177.3(1)	-63.2(1)
C22–C7–C8–H8B	–	-62.4
C22–C7–C8–H8A	–	54.9
C22–CA–C8–C9A	–	176.25(9)
C6–C7–C20–H20A	171.0	–
C6–C7–C20–H20B	51.0	–
C6–C7–C20–H20C	-69.0	–
C6–C7–C21–H21A	–	58.1
C6–C7–C21–H21B	–	-61.9
C6–C7–C21–H21C	–	178.07
C8–C7–C20–H20A	52.0	–
C8–C7–C20–H20B	-68.0	–
C8–C7–C20–H20C	172.0	–
C8–C7–C21–H21A	–	176.94
C8–C7–C21–H21B	–	56.9
C8–C7–C21–H21C	–	-63.1
C21–C7–C20–H20A	-68.7	–
C21–C7–C20–H20B	171.3	–
C21–C7–C20–H20C	51.3	–
C22–C7–C21–H21A	–	-62.2
C22–C7–C21–H21B	–	177.84
C22–C7–C21–H21C	–	57.8
C6–C7–C21–H21A	56.4	–
C6–C7–C21–H21B	-63.6	–
C6–C7–C21–H21C	176.4	–
C6–C7–C22–H22A	–	-176.4
C6–C7–C22–H22B	–	63.6
C6–C7–C22–H22C	–	-56.4
C8–C7–C21–H21A	174.0	–
C8–C7–C21–H21B	54.0	–
C8–C7–C21–H21C	-66.0	–
C8–C7–C22–H22A	–	65.4
C8–C7–C22–H22B	–	-54.6
C8–C7–C22–H22C	–	-174.6
C20–C7–C21–H21A	-64.4	–
C20–C7–C21–H21B	175.6	–
C20–C7–C21–H21C	55.6	–
C21–C7–C22–H22A	–	-55.2
C21–C7–C22–H22B	–	-175.2
C21–C7–C22–H22C	–	64.8

C7–C8–C9–C12	40.8(2)	-33.2(1)
C7–C8–C9–O15	-140.8(1)	147.5(1)
H8A–C8–C9–C12	-80.6	88.2
H8A–C8–C9–O15	97.8	-91.1
H8B–C8–C9–C12	162.3	-154.59
H8B–C8–C9–O15	-19.3	26.1
C8–C9–C12–C11	-10.2(2)	1.9(1)
C8–C9–C12–C13	167.8(1)	177.39(9)
O15–C9–C12–C11	171.4(1)	-178.8(1)
O15–C9–C12–C13	-10.6(2)	-3.3(2)
C3–C10–C13–C12	177.9(1)	174.25(9)
C3–C10–C13–O1	0.0(1)	-1.1(1)
C4–C10–C13–C12	-1.9(2)	-3.1(1)
C4–C10–C13–O1	-179.8(1)	-178.43(8)
C6–C11–C12–C9	-0.2(2)	3.0(1)
C6–C11–C12–C13	-178.4(1)	-172.89(9)
O5–C11–C12–C9	177.9(1)	-179.15(9)
O5–C11–C12–C13	-0.4(2)	4.9(1)
C6–C11–O5–C4	179.9(1)	171.48(9)
C12–C11–O5–C4	1.5(2)	-6.6(1)
C9–C12–C13–C10	-177.5(1)	-175.92(9)
C9–C12–C13–O1	0.0(2)	-1.2(2)
C11–C12–C13–C10	0.6(2)	-0.3(1)
C11–C12–C13–O1	178.2(1)	174.46(9)
C10–C13–O1–C2	0.1(1)	1.3(1)
C12–C13–O1–C2	-177.7(1)	-174.05(9)
C3–C16–O18–C19	-175.4(1)	175.70(9)
O17–C16–O18–C19	3.5(2)	-2.1(2)
H19A–C19–O18–C16	-62.7	–
H19B–C19–O18–C16	177.3	–
H19C–C19–O18–C16	57.3	–
H19A–C19–C20–H20A	–	60.4
H19A–C19–C20–H20B	–	-59.6
H19A–C19–C20–H20C	–	-179.6
H19B–C19–C20–H20A	–	-179.6
H19B–C19–C20–H20B	–	60.4
H19B–C19–C20–H20C	–	-59.6
O18–C19–C20–H20A	–	-59.6
O18–C19–C20–H20B	–	-179.6
O18–C19–C20–H20C	–	60.4
H19A–C19–O18–C16	–	75.0
H19B–C19–O18–C16	–	-45.0
C20–C19–O18–C16	–	-164.96(9)

Table S3. Angles (τ) in the molecule of compounds **5a** and **5b**

**Angle**	**τ/deg**	
	**5a**	**5b**
	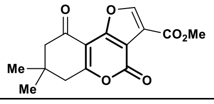	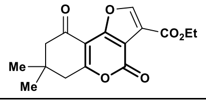
H2–C2–C3	124.3	124.5
H2–C2–O1	124.4	124.5
C3–C2–O1	111.3(1)	110.98(9)
C2–C3–C10	105.7(1)	105.67(8)

C2–C3–C16	126.2(1)	124.05(9)
C10–C3–C16	128.0(1)	130.14(9)
C10–C4–O5	114.0(1)	113.66(9)
C10–C4–O14	130.9(1)	130.5(1)
O5–C4–O14	115.1(1)	115.85(9)
H6A–C6–H6B	107.8	107.97
H6A–C6–C7	109.1	109.33
H6A–C6–C11	109.1	109.33
H6B–C6–C7	109.1	109.33
H6B–C6–C11	109.1	109.33
C7–C6–C11	112.4(1)	111.48(8)
C6–C7–C8	107.6(1)	107.85(8)
C6–C7–C20	110.2(1)	–
C6–C7–C21	109.4(1)	110.66(8)
C6–C7–C22	–	109.06(9)
C8–C7–C20	110.9(1)	–
C8–C7–C21	109.3(1)	109.69(9)
C8–C7–C22	–	110.10(9)
C20–C7–C21	109.4(1)	–
C21–C7–C22	–	109.46(9)
C7–C8–H8A	108.8	108.89
C7–C8–H8B	108.8	108.90
C7–C8–C9	113.7(1)	113.38(9)
H8A–C8–H8B	107.7	107.7
H8A–C8–C9	108.8	108.89
H8B–C8–C9	108.8	108.89
C8–C9–C12	115.0(1)	116.46(9)
C8–C9–O15	123.0(1)	121.96(9)
C12–C9–O15	122.0(1)	121.57(9)
C3–C10–C4	134.4(1)	134.24(9)
C3–C10–C13	106.1(1)	106.10(8)
C4–C10–C13	119.5(1)	119.59(9)
C6–C11–C12	125.6(1)	124.50(9)
C6–C11–O5	112.1(1)	113.20(8)
C12–C11–O5	122.3(1)	122.27(8)
C9–C12–C11	119.5(1)	119.89(9)
C9–C12–C13	125.6(1)	125.27(8)
C11–C12–C13	114.9(1)	114.70(8)
C10–C13–C12	124.5(1)	124.54(9)
C10–C13–O1	110.5(1)	110.50(8)
C12–C13–O1	125.0(1)	124.78(8)
C3–C16–O17	124.3(1)	124.6(1)
C3–C16–O18	112.1(1)	110.86(9)
O17–C16–O18	123.5(1)	124.5(1)
H19A–C19–H19B	109.5	108.6
H19A–C19–H19C	109.5	–
H19A–C19–C20	–	110.3
H19A–C19–O18	109.5	110.4
H19B–C19–H19C	109.5	–
H19B–C19–C20	–	110.4
H19B–C19–O18	109.5	110.4
H19C–C19–O18	109.5	
C20–C19–O18	–	106.86(9)
C7–C20–H20A	109.5	–
C7–C20–H20B	109.5	–

C7–C20–H20C	109.5	–
C19–C20–H20A	–	109.5
C19–C20–H20B	–	109.5
C19–C20–H20C	–	109.5
H20A–C20–H20B	109.5	109.5
H20A–C20–H20C	109.5	109.5
H20B–C20–H20C	109.5	109.5
C7–C21–H21A	109.5	109.47
C7–C21–H21B	109.5	109.47
C7–C21–H21C	109.5	109.48
H21A–C21–H21B	109.5	109.5
H21A–C21–H21C	109.5	109.5
H21B–C21–H21C	109.5	109.5
C2–O1–C13	106.36(9)	106.73(8)
C4–O5–C11	124.8(1)	124.95(8)
C16–O18–C19	113.1(1)	116.49(9)
C7–C22–H22A	–	109.5
C7–C22–H22B	–	109.5
C7–C22–H22C	–	109.5
H22A–C22–H22B	–	109.5
H22A–C22–H22C	–	109.5
H22B–C22–H22C	–	109.5

Table S4. Bond lengths (*d*) in the molecule of compounds **5a** and **5b**

**Bond**	***d*/Å**	
	**5a**	**5b**
	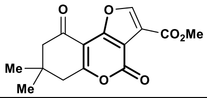	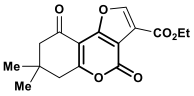
C2–H2	0.950	0.950
C2–C3	1.352(2)	1.359(2)
C2–O1	1.374(1)	1.375(1)
C3–C10	1.443(2)	1.444(1)
C3–C16	1.471(2)	1.475(2)
C4–C10	1.439(2)	1.440(1)
C4–O5	1.410(1)	1.414(1)
C4–O14	1.202(2)	1.199(2)
C6–H6A	0.990	0.990
C6–H6B	0.990	0.990
C6–C7	1.538(2)	1.538(2)
C6–C11	1.485(2)	1.488(1)
C7–C8	1.539(2)	1.537(2)
C7–C20	1.529(2)	–
C7–C21	1.527(2)	1.533(1)
C7–C22	–	1.528(2)
C8–H8A	0.990	0.990
C8–H8B	0.990	0.990
C8–C9	1.506(2)	1.509(2)
C9–C12	1.480(2)	1.477(2)
C9–O15	1.222(2)	1.220(1)
C10–C13	1.371(2)	1.373(2)
C11–C12	1.364(2)	1.365(1)
C11–O5	1.352(1)	1.352(1)
C12–C13	1.422(2)	1.424(1)

C13–O1	1.360(1)	1.357(1)
C16–O17	1.202(2)	1.201(2)
C16–O18	1.343(2)	1.333(1)
C19–H19A	0.980	0.990
C19–H19B	0.980	0.990
C19–H19C	0.980	–
C19–C20	–	1.502(2)
C19–O18	1.444(1)	1.456(2)
C20–H20A	0.980	0.980
C20–H20B	0.980	0.980
C20–H20C	0.980	0.980
C21–H21A	0.980	0.980
C21–H21B	0.980	0.980
C21–H21C	0.980	0.980
C22–H22A	–	0.980
C22–H22B	–	0.980
C22–H22C	–	0.980

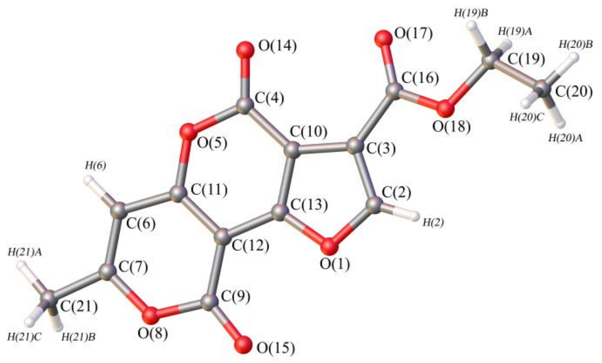

Figure S109. Perspective views of ethyl 7-methyl-4,9-dioxo-4*H*,9*H*-furo[2,3-*d*]pyrano[4,3-*b*]pyran-3-carboxylate (**6b**) (X-ray data).

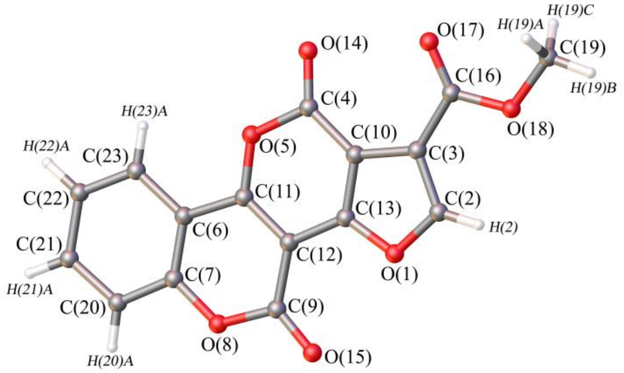

Figure S110. Perspective views of methyl 4,11-dioxo-4*H*,11*H*-furo[2',3':4,5]pyrano[3,2-*c*]chromene-1-carboxylate (**6c**) (X-ray data).

Table S5. Torsion angles (τ) in the molecule of compounds **6b** and **6c**

**Angle**	**τ/deg**	
	**6b**	**6c**
	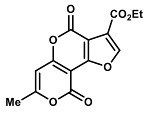	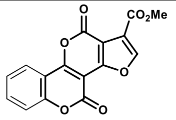
C2–O1–C13–C10	-0.1(1)	1.9(9)
C2–O1–C13–C12	-179.9(1)	-177.8(8)
C13–O1–C2–H2	179.8	178.2
C13–O1–C2–C3	-0.2(1)	-1.9(9)
C19–O18–C16–O17	-3.4(2)	1(1)
C19–O18–C16–C3	176.16(9)	-176.9(8)
C16–O18–C19–H19A	57.4	-42
C16–O18–C19–H19B	-62.4	-162.3
H19C–C19–O18A–C16	–	78
C16–O18–C19–C20	177.5(1)	–
C4–O5–C11–C12	-0.5(2)	4(1)
C4–O5–C11–C6	-179.8(1)	-178.0(7)
C11–O5–C4–O14	-178.1(1)	174.5(7)

C11–O5–C4–C10	2.3(2)	-4(1)
C7–O8–C9–O15	178.6(1)	179.4(8)
C7–O8–C9–C12	-0.8(2)	-0(1)
C9–O8–C7–C6	-0.3(2)	-2(1)
C9–O8–C7–C21	-179.6(1)	–
O1–C13–C10–C3	0.3(1)	-1.2(9)
O1–C13–C10–C4	-176.5(1)	180.0(7)
C12–C13–C10–C3	-179.9(1)	178.4(8)
C12–C13–C10–C4	3.3(2)	-0(1)
O1–C13–C12–C9	-2.2(2)	-0(1)
O1–C13–C12–C11	178.5(1)	-179.8(8)
C10–C13–C12–C9	178.0(1)	-179.8(8)
C10–C13–C12–C11	-1.3(2)	1(1)
C13–C10–C3–C2	-0.4(1)	0(1)
C13–C10–C3–C16	176.6(1)	-178.0(8)
C4–C10–C3–C2	175.7(1)	179(1)
C4–C10–C3–C16	-7.3(2)	1(2)
C13–C10–C4–O5	-3.6(2)	2(1)
C13–C10–C4–O14	176.9(1)	-176.0(9)
C3–C10–C4–O5	-179.3(1)	-176.7(9)
C3–C10–C4–O14	1.2(2)	6(2)
O1–C2–C3–C10	0.4(1)	1(1)
O1–C2–C3–C16	-176.9(1)	179.3(8)
H2–C2–C3–C10	-179.6	-178.9
H2–C2–C3–C16	3.1	-1
C10–C3–C16–O18	169.0(1)	-177.7(8)
C10–C3–C16–O17	-11.5(2)	5(2)
C2–C3–C16–O18	-14.5(2)	5(1)
C2–C3–C16–O17	165.1(1)	-173.0(9)
O15–C9–C12–C13	2.5(2)	2(1)
O15–C9–C12–C11	-178.3(1)	-178.4(8)
O8–C9–C12–C13	-178.2(1)	-178.1(8)
O8–C9–C12–C11	1.0(2)	1(1)
C13–C12–C11–O5	-0.2(2)	-2(1)
C13–C12–C11–C6	179.0(1)	179.8(8)
C9–C12–C11–O5	-179.5(1)	178.1(7)
C9–C12–C11–C6	-0.3(2)	0(1)
O5–C11–C6–C7	178.5(1)	179.2(7)
O5–C11–C6–H6	-1.5	–
C12–C11–C6–C7	-0.7(2)	-3(1)
C12–C11–C6–H6	179.3	–
O8–C7–C6–C11	1.0(2)	4(1)
O8–C7–C6–H6	-179.0	–
C21–C7–C6–C11	-179.7(1)	–
C21–C7–C6–H6	0.3	–
O8–C7–C21–H21A	-173.6	–
O8–C7–C21–H21B	66.4	–
O8–C7–C21–H21C	-53.6	–
C6–C7–C21–H21A	7.1	–
C6–C7–C21–H21B	-112.9	–
C6–C7–C21–H21C	127.1	–
O18–C19–C20–H20A	-60.9	–
O18–C19–C20–H20B	179.1	–
O18–C19–C20–H20C	59.1	–
H19A–C19–C20–H20A	59.2	–

H19A–C19–C20–H20B	-60.8	–
H19A–C19–C20–H20C	179.2	–
H19B–C19–C20–H20A	179.0	–
H19B–C19–C20–H20B	59.0	–
H19B–C19–C20–H20C	-61.0	–
C11–C6–C7–C20	–	-180.0(8)
C23–C6–C7–C20	–	-3(1)
C23–C6–C7–O8	–	-178.7(8)
C23–C6–C11–C12	–	-180.0(8)
C23–C6–C11–O5	–	2(1)
C7–C6–C23–C22	–	1(1)
C7–C6–C23–H23A	–	-179.0
C11–C6–C23–C22	–	178.0(8)
C11–C6–C23–H23A	–	-2
C6–C7–C20–H20A	–	-176.0
C6–C7–C20–C21	–	4(1)
O8–C7–C20–H20A	–	0
O8–C7–C20–C21	–	-179.6(8)
C20–C7–O8–C9	–	-178.6(7)
C7–C20–C21–H21A	–	176.4
C7–C20–C21–C22	–	-4(1)
H20A–C20–C21–H21A	–	-3
H20A–C20–C21–C22	–	176.4
C20–C21–C22–H22A	–	-178.1
C20–C21–C22–C23	–	2(1)
H21A–C21–C22–H22A	–	2
H21A–C21–C22–C23	–	-178.1
C21–C22–C23–C6	–	-1(1)
C21–C22–C23–H23A	–	179.3
H22A–C22–C23–C6	–	179.5
H22A–C22–C23–H23A	–	-1

Table S6. Angles (τ) in the molecule of compounds **6b** and **6c**

**Angle**	**τ/deg**	
	**6b**	**6c**
	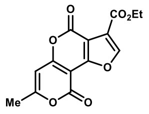	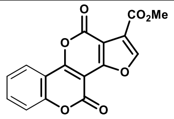
C13–O1–C2	105.99(9)	105.8(6)
C16–O18–C19	115.58(9)	116.6(7)
C11–O5–C4	124.46(9)	124.5(7)
C9–O8–C7	123.66(9)	121.2(7)
O1–C13–C10	110.9(1)	109.0(7)
O1–C13–C12	124.7(1)	125.3(7)
C10–C13–C12	124.5(1)	125.7(8)
C13–C10–C3	106.1(1)	106.2(7)
C13–C10–C4	119.8(1)	117.1(8)
C3–C10–C4	134.0(1)	136.6(8)
O1–C2–H2	124.3	124.6
O1–C2–C3	111.5(1)	110.7(7)
H2–C2–C3	124.3	124.7
C10–C3–C2	105.6(1)	108.2(8)
C10–C3–C16	130.5(1)	127.5(8)

C2–C3–C16	123.8(1)	124.3(8)
O18–C16–O17	125.0(1)	125.0(8)
O18–C16–C3	109.3(1)	109.5(7)
O17–C16–C3	125.7(1)	125.4(8)
O15–C9–O8	117.7(1)	117.5(7)
O15–C9–C12	127.0(1)	125.8(8)
O8–C9–C12	115.3(1)	116.7(7)
C13–C12–C9	125.1(1)	124.2(8)
C13–C12–C11	115.0(1)	115.9(8)
C9–C12–C11	119.9(1)	119.9(8)
O5–C11–C12	122.3(1)	–
O5–C11–C6	115.9(1)	117.3(7)
C12–C11–C6	121.8(1)	121.9(8)
C12–C11–O5	–	120.8(8)
O8–C7–C6	121.5(1)	122.7(8)
O8–C7–C20	–	115.3(7)
O8–C7–C21	112.5(1)	–
C6–C7–C20	–	121.9(8)
C6–C7–C21	126.0(1)	–
C11–C6–C7	117.8(1)	117.6(8)
C11–C6–H6	121.1	–
C11–C6–C23	–	124.7(8)
C7–C6–H6	121.1	–
C7–C6–C23	–	117.6(8)
O5–C4–O14	115.4(1)	114.7(7)
O5–C4–C10	113.9(1)	115.9(7)
O14–C4–C10	130.7(1)	129.4(8)
O18–C19–H19A	110.3	109
O18–C19–H19B	110.3	109
O18–C19–H19C	–	109
O18–C19–C20	107.3(1)	–
H19A–C19–H19B	108.5	110
H19A–C19–H19C	–	110
H19B–C19–C19C	–	109
H19A–C19–C20	110.3	–
H19B–C19–C20	110.3	–
C7–C20–C21	–	118.1(8)
C7–C20–H20A	–	121.0
C7–C21–H21A	109.5	–
C7–C21–H21B	109.5	–
C7–C21–H21C	109.5	–
H20A–C20–C21	–	120.9
C20–C21–H21A	–	118.6
C20–C21–C22	–	122.9(9)
H21A–C21–H21B	109.5	–
H21A–C21–H21C	109.5	–
H21B–C21–H21C	109.5	–
H21A–C21–C22	–	118.5
C21–C22–H22A	–	120.8
C21–C22–C23A	–	118.3(8)
C19–C20–H20A	109.5	–
C19–C20–H20B	109.5	–
C19–C20–H20C	109.5	–
H20A–C20–H20B	109.5	–
H20A–C20–H20C	109.5	–

H20B–C20–H20C	109.5	–
H22A–C22–C23	–	120.9
C6–C23–C22	–	121.1(8)
C6–C23–H23A	–	119.5
C22–C23–H23A	–	119.4

Table S7. Bond lengths (*d*) in the molecule of compounds **6b** and **6c**

**Bond**	***d*/Å**	
	**6b**	**6c**
	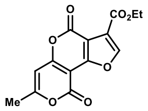	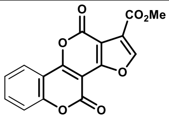
O1–C13	1.356(1)	1.38(1)
O1–C2	1.379(1)	1.38(1)
O18–C16	1.345(2)	1.36(1)
O18–C19	1.453(1)	1.43(1)
O17–C16	1.205(1)	1.18(1)
O5–C11	1.354(1)	1.36(1)
O5–C4	1.428(1)	1.42(1)
O15–C9	1.207(2)	1.22(1)
O8–C9	1.392(2)	1.40(1)
O8–C7	1.371(2)	1.37(1)
O14–C4	1.198(1)	1.21(1)
C13–C10	1.374(1)	1.39(1)
C13–C12	1.424(2)	1.40(1)
C10–C3	1.439(2)	1.40(1)
C10–C4	1.442(2)	1.44(1)
C2–H2	0.950	0.950
C2–C3	1.354(2)	1.33(1)
C3–C16	1.482(1)	1.50(1)
C9–C12	1.449(1)	1.46(1)
C12–C11	1.378(2)	1.38(1)
C11–C6	1.419(2)	1.40(1)
C7–C6	1.352(2)	1.38(1)
C7–C20	–	1.42(1)
C7–C21	1.480(2)	–
C6–H6	0.950	–
C19–H19A	0.990	0.98
C19–H19B	0.990	0.98
C19–H19C	–	0.98
C19–C20	1.509(2)	–
C20–C21	–	1.33(1)
C21–C22	–	1.41(1)
C21–H21A	0.980	0.95
C21–H21B	0.980	–
C21–H21C	0.980	–
C20–H20A	0.980	0.951
C20–H20B	0.980	–
C20–H20C	0.980	–
C22–H22	–	0.950
C22–C23	–	1.38(1)
C23–H23A	–	0.950
C23–C6	–	1.41(1)

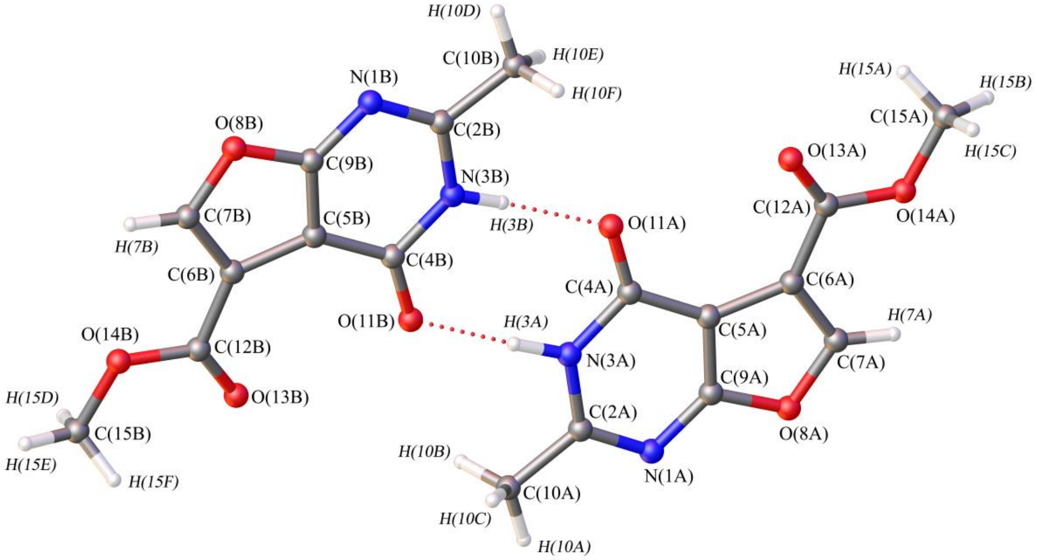

Figure S111. Perspective views of methyl 2-methyl-4-oxo-3,4-dihydrofuro[2,3-*d*]pyrimidine-5-carboxylate (**7a**) (X-ray data).

Table S8. Torsion angles (τ) in the molecule of compound **7a**

Angle	τ/deg	Angle	τ/deg
N1A–C2A–C10A–H10A	-0.4	N1B–C2B–C10B–H10D	-10.8
N1A–C2A–C10A–H10B	-120.37	N1B–C2B–C10B–H10E	-130.78
N1A–C2A–C10A–H10C	119.63	N1B–C2B–C10B–H10F	109.2
N3A–C2A–C10A–H10A	-179.46	N3B–C2B–C10B–H10D	170.47
N3A–C2A–C10A–H10B	60.5	N3B–C2B–C10B–H10E	50.5
N3A–C2A–C10A–H10C	-59.5	N3B–C2B–C10B–H10F	-69.5
C10A–C2A–N1A–C9A	-176.73(8)	C10B–C2B–N1B–C9B	-179.50(8)
N3A–C2A–N1A–C9A	2.3(1)	N3B–C2B–N1B–C9B	-0.8(1)
C10A–C2A–N3A–C4A	176.70(8)	C10B–C2B–N3B–C4B	-179.58(8)
C10A–C2A–N3A–H3A	-2(1)	C10B–C2B–N3B–H3B	-4(1)
N1A–C2A–N3A–C4A	-2.4(1)	N1B–C2B–N3B–C4B	1.7(1)
N1A–C2A–N3A–H3A	179(1)	N1B–C2B–N3B–H3B	178(1)
O11A–C4A–C5A–C6A	2.3(2)	N3B–C4B–C5B–C6B	-179.9(1)
O11A–C4A–C5A–C9A	-176.95(9)	N3B–C4B–C5B–C9B	-0.2(1)
N3A–C4A–C5A–C6A	-178.13(9)	O11B–C4B–C5B–C6B	-0.8(2)
N3A–C4A–C5A–C9A	2.6(1)	O11B–C4B–C5B–C9B	178.93(9)
C5A–C4A–N3A–C2A	-0.4(1)	C5B–C4B–N3B–C2B	-1.0(1)
C5A–C4A–N3A–H3A	179(1)	C5B–C4B–N3B–H3B	-177(1)
O11A–C4A–N3A–C2A	179.24(8)	O11B–C4B–N3B–C2B	179.73(8)
O11A–C4A–N3A–H3A	-2(1)	O11B–C4B–N3B–H3B	4(1)
C4A–C5A–C6A–C7A	-178.8(1)	C4B–C5B–C6B–C7B	179.6(1)
C4A–C5A–C6A–C12A	4.1(2)	C4B–C5B–C6B–C12B	-3.7(2)
C9A–C5A–C6A–C7A	0.52(9)	C9B–C5B–C6B–C7B	-0.16(9)
C9A–C5A–C6A–C12A	-176.58(8)	C9B–C5B–C6B–C12B	176.58(8)
C4A–C5A–C9A–N1A	-2.9(1)	C4B–C5B–C9B–N1B	1.1(1)
C4A–C5A–C9A–O8A	178.92(7)	C4B–C5B–C9B–O8B	-179.58(7)
C6A–C5A–C9A–N1A	177.60(9)	C6B–C5B–C9B–N1B	-179.17(9)
C6A–C5A–C9A–O8A	-0.57(9)	C6B–C5B–C9B–O8B	0.20(9)
C5A–C6A–C7A–H7A	179.69	C5B–C6B–C7B–H7B	-179.93
C5A–C6A–C7A–O8A	-0.3(1)	C5B–C6B–C7B–O8B	0.1(1)
C12A–C6A–C7A–H7A	-3.0	C12B–C6B–C7B–H7B	3.1
C12A–C6A–C7A–O8A	176.97(8)	C12B–C6B–C7B–O8B	-176.87(8)
C5A–C6A–C12A–O13A	7.6(2)	C5B–C6B–C12B–O13B	-1.4(1)

C5A–C6A–C12A–O14A	-172.27(8)	C5B–C6B–C12B–O14B	179.27(8)
C7A–C6A–C12A–O13A	-168.99(9)	C7B–C6B–C12B–O13B	174.81(9)
C7A–C6A–C12A–O14A	11.1(1)	C7B–C6B–C12B–O14B	-4.6(1)
C6A–C7A–O8A–C9A	-0.04(9)	C6B–C7B–O8B–C9B	0.1(1)
H7A–C7A–O8A–C9A	179.97	H7B–C7B–O8B–C9B	-179.95
C5A–C9A–N1A–C2A	0.3(1)	C5B–C9B–N1B–C2B	-0.5(1)
O8A–C9A–N1A–C2A	178.37(7)	O8B–C9B–N1B–C2B	-179.84(7)
C5A–C9A–O8A–C7A	0.39(9)	C5B–C9B–O8B–C7B	-0.16(9)
N1A–C9A–O8A–C7A	-178.02(7)	N1B–C9B–O8B–C7B	179.29(8)
C6A–C12A–O14A–C15A	-178.52(7)	C6B–C12B–O14B–C15B	173.60(7)
O13A–C12A–O14A–C15A	1.6(1)	O13B–C12B–O14B–C15B	-5.8(1)
H15A–C15A–O14A–C12A	-34.9	H15D–C15B–O14B–C12B	-73.8
H15B–C15A–O14A–C12A	-154.88	H15E–C15B–O14B–C12B	166.19
H15C–C15A–O14A–C12A	85.1	H15F–C15B–O14B–C12B	46.2

Table S9. Angles (τ) in the molecule of compound **7a**

**Angle**	**τ/deg**	**Angle**	**τ/deg**
C10A–C2A–N1A	120.38(8)	C10B–C2B–N1B	120.71(8)
C10A–C2A–N3A	116.84(7)	C10B–C2B–N3B	116.74(7)
N1A–C2A–N3A	122.77(8)	N1B–C2B–N3B	122.54(8)
C5A–C4A–O11A	128.40(8)	C5B–C4B–N3B	111.85(7)
C5A–C4A–N3A	111.77(7)	C5B–C4B–O11B	128.06(8)
O11A–C4A–N3A	119.82(8)	N3B–C4B–O11B	120.09(8)
C4A–C5A–C6A	137.80(8)	C4B–C5B–C6B	137.86(8)
C4A–C5A–C9A	116.39(8)	C4B–C5B–C9B	116.23(8)
C6A–C5A–C9A	105.81(7)	C6B–C5B–C9B	105.90(7)
C5A–C6A–C7A	105.44(7)	C5B–C6B–C7B	105.47(7)
C5A–C6A–C12A	129.64(8)	C5B–C6B–C12B	129.59(8)
C7A–C6A–C12A	124.86(8)	C7B–C6B–C12B	124.85(8)
C6A–C7A–H7A	124.03	C6B–C7B–H7B	124.11
C6A–C7A–O8A	111.94(7)	C6B–C7B–O8B	111.79(8)
H7A–C7A–O8A	124.03	H7B–C7B–O8B	124.11
C5A–C9A–N1A	130.33(8)	C5B–C9B–N1B	130.27(8)
C5A–C9A–O8A	110.76(7)	C5B–C9B–O8B	110.66(7)
N1A–C9A–O8A	118.89(7)	N1B–C9B–O8B	119.07(7)
C2A–C10A–H10A	109.47	C2B–C10B–H10D	109.48
C2A–C10A–H10B	109.48	C2B–C10B–H10E	109.47
C2A–C10A–H10C	109.47	C2B–C10B–H10F	109.47
H10A–C10A–H10B	109.47	H10D–C10B–H10E	109.47
H10A–C10A–H10C	109.47	H10D–C10B–H10F	109.47
H10B–C10A–H10C	109.47	H10E–C10B–H10F	109.47
C6A–C12A–O13A	125.04(8)	C6B–C12B–O13B	125.02(8)
C6A–C12A–O14A	110.74(7)	C6B–C12B–O14B	110.85(7)
O13A–C12A–O14A	124.23(8)	O13B–C12B–O14B	124.12(8)
H15A–C15A–H15B	109.5	H15D–C15B–H15E	109.5
H15A–C15A–H15C	109.5	H15D–C15B–H15F	109.5
H15A–C15A–O14A	109.47	H15D–C15B–O14B	109.47
H15B–C15A–H15C	109.5	H15E–C15B–H15F	109.5
H15B–C15A–O14A	109.47	H15E–C15B–O14B	109.47
H15C–C15A–O14A	109.47	H15F–C15B–O14B	109.47
C2A–N1A–C9A	112.41(7)	C2B–N1B–C9B	112.61(7)
C7A–O8A–C9A	106.05(7)	C2B–N3B–C4B	126.48(7)
C12A–O14A–C15A	115.72(7)	C2B–N3B–H3B	117(1)
C2A–N3A–C4A	126.22(7)	C4B–N3B–H3B	116(1)

C2A–N3A–H3A	116(1)	C7B–O8B–C9B	106.19(7)
C4A–N3A–H3A	118(1)	C12B–O14B–C15B	114.96(7)

Table S10. Bond lengths (*d*) in the molecule of compound **7a**

Bond	*d*/Å	Bond	*d*/Å
C2A–C10A	1.485(1)	C2B–C10B	1.487(1)
C2A–N1A	1.318(1)	C2B–N1B	1.317(1)
C2A–N3A	1.361(1)	C2B–N3B	1.359(1)
C4A–C5A	1.435(1)	C4B–C5B	1.436(1)
C4A–O11A	1.236(1)	C4B–N3B	1.392(1)
C4A–N3A	1.396(1)	C4B–O11B	1.235(1)
C5A–C6A	1.443(1)	C5B–C6B	1.443(1)
C5A–C9A	1.381(1)	C5B–C9B	1.381(1)
C6A–C7A	1.359(1)	C6B–C7B	1.362(1)
C6A–C12A	1.472(1)	C6B–C12B	1.473(1)
C7A–H7A	0.950	C7B–H7B	0.950
C7A–O8A	1.369(1)	C7B–O8B	1.368(1)
C9A–N1A	1.341(1)	C9B–N1B	1.341(1)
C9A–O8A	1.362(1)	C9B–O8B	1.364(1)
C10A–H10A	0.980	C10B–H10D	0.980
C10A–H10B	0.980	C10B–H10E	0.980
C10A–H10C	0.980	C10B–H10F	0.980
C12A–O13A	1.204(1)	C12B–O13B	1.203(1)
C12A–O14A	1.345(1)	C12B–O14B	1.347(1)
C15A–H15A	0.980	C15B–H15D	0.980
C15A–H15B	0.980	C15B–H15E	0.980
C15A–H15C	0.980	C15B–H15F	0.980
C15A–O14A	1.449(1)	C15B–O14B	1.447(1)
N3A–H3A	0.97(2)	N3B–H3B	0.89(2)

References

1. Kakinuma, S. Method for Producing 2-Hydroxy-1,4-Naphthoquinone. Patent EP 3 339 283 A1, June 27, 2018.
2. Ziegler, E.; Junek, H.; Herzog, U. *Monatsh. Chem.* **1971**, *102*, 1626-1630. doi: 10.1007/BF00917215
3. Davidson, D.; Bernhard, S. A. *J. Am. Chem. Soc.* **1948**, *70*, 3426-3428. doi: 10.1021/ja01190a060
4. Omori, A.; Sonoda, N.; Tsutsumi, S. *J. Org. Chem.* **1969**, *34*, 2480-2482. doi: 10.1021/jo01260a048
5. Kobayashi, K.; Nagase, K.; Morikawa, O.; Konishi, H. *Heterocycles* **2003**, *60*, 939-946.
6. Potapova, A. E.: Kuvaeva, E. V.; Yakovlev, I. P.; Fedorova, E. V.; Shchegolev, A. E. *Butlerov Commun*. **2015**, *44*, 65-68.
7. Hari, K. N.; Poojary, B.; Chandrasehar, G. *Results in Chem.* **2022**, *4*, 100676. doi: 10.1016/j.rechem.2022.100676
